# Evaluating the Relative Importance of Polyfluoroalkyl Substances in AFFF-Impacted Soils

**DOI:** 10.1021/acs.est.5c17749

**Published:** 2026-04-27

**Authors:** Sara L. Jones, Nicholas Gonda, J. Conrad Pritchard, Jaydon Richardson, Matthew C. Bigler, Mark L. Brusseau, Bo Guo, James Hatton, Maxwell Hire, Charles E. Schaefer, Christopher P. Higgins

**Affiliations:** † Department of Civil and Environmental Engineering, 3557 Colorado School of Mines, 1500 Illinois Street, Golden, Colorado 80401, United States; ‡ Department of Environmental Science, 8041 The University of Arizona, 1177 East fourth Street, Tucson, Arizona 85721, United States; § Department of Hydrology and Atmospheric Sciences, The University of Arizona, 1133 James E. Rogers Way, Tucson, Arizona 85721, United States; ∥ 41930 Jacobs Engineering Group Inc., Englewood, Colorado 80112, United States; ⊥ CDM Smith, 110 Fieldcrest Avenue, #8, Sixth Floor, Edison, New Jersey 08837, United States

**Keywords:** PFASs, AFFF, soils, polyfluoroalkyl substances, PFAA precursors, 6:2 FTAB, EPA Method 1633

## Abstract

This multisite study analyzed depth-discrete soil samples in 12 cores (≤2 m from the ground surface) from 10 aqueous film-forming foam (AFFF)-impacted U.S. Department of Defense installations. A broad suite of per- and polyfluoroalkyl substances (PFASs) were analyzed using extraction protocols designed for source-zone soils and liquid chromatography-high resolution mass spectrometry (LC-HRMS) with targeted and semiquantitative workflows. Across all samples, 162 PFASs spanning 50 classes were identified, with both electrochemical fluorination (ECF)- and fluorotelomer (FT)-derived signatures present at nearly all sites. Class-level detection frequency analysis showed that shallow intervals (down to 30 cm below ground surface) captured most class diversity at the studied sites. Regarding total PFAS mass within a soil core, precursors frequently dominated, though several cores were perfluoroalkyl acid (PFAA)-dominated. These data indicate that reliance on the Environmental Protection Agency (EPA) Method 1633 target list alone substantially underestimated precursor mass in all 12 studied cores. Vertical profiles of PFAAs and precursors showed varying trends and correlations in concentration with depth, suggesting site-specific transport and transformation phenomena. The results of this study point toward several polyfluoroalkyl substances that may be considered for prompt investigation while also highlighting a need for detailed characterizations of diverse AFFF-impacted sites.

## Graphical abstract

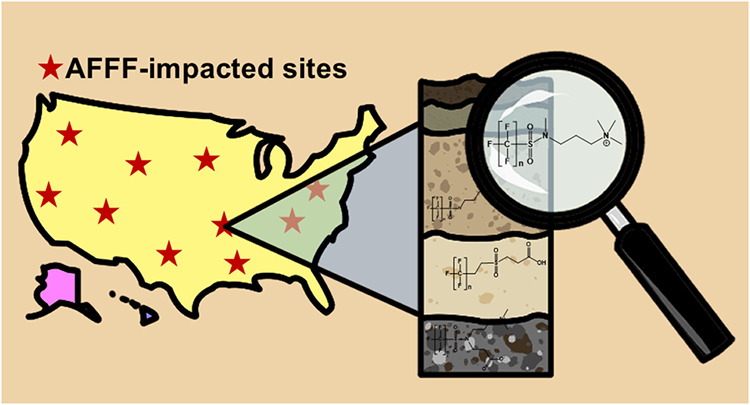

## 1 Introduction

The widespread environmental releases of per- and polyfluoroalkyl substances (PFASs), particularly through the use of aqueous film forming foam (AFFF), has led to concerns due to PFAS toxicity and bioaccumulation.^1–6^ Since the 1960s, AFFF has been extensively employed at military bases, airports, oil refineries, and municipal firefighter training facilities,^1,2,7^ resulting in significant environmental releases of PFASs.^1,8^ Regulatory agencies such as the United States Environmental Protection Agency (U.S. EPA) often focus remediation efforts on a handful of seemingly ubiquitous perfluoroalkyl acids (PFAAs), which are known components of legacy AFFF formulations.^7,9^ However, AFFF characterization studies from the past two decades have revealed that these foams are highly complex mixtures containing diverse classes of PFASs.^5,7,10,11^ Much of this diversity is comprised of polyfluorinated substances, often referred to as PFAA precursors due to their susceptibility to undergo transformation.^5,7,10,12,13^ The rates and means by which these polyfluoroalkyl substances transform into the more persistent PFAAs are the subject of much study,^14–16^ but it remains unclear which are most critical for predicting future PFAA mass loadings to receiving groundwaters and surface waters at AFFF-impacted sites.

Recent studies have provided valuable insight into the processes dictating PFAS migration at AFFF-impacted sites.^17^ In unsaturated soils, PFASs may partition to solid phases via hydrophobic sorption and electrostatic interactions, both of which have been shown to be important mechanisms for PFAS retention.^18–20^ Field-based investigations utilizing depth-discrete sampling techniques at AFFF-impacted sites have also highlighted the role of polyfluoroalkyl substances in the leaching of PFAAs from surface soils to the groundwater table. For example, large fractions of PFAS mass have been observed in shallow soils at or near the source long after AFFF use has ceased.^6,21–24^ Modeling work similarly indicates substantial retention and slow release from unsaturated soils over decadal timeframes, underscoring soils’ likely role as primary sources to aquifers and downgradient receptors.^13,23,25–30^ With improved soil extraction protocols^31,32^ and quantitative liquid chromatography-high resolution mass spectrometry (LC-HRMS) approaches,^31^ a significant fraction of this observably less-mobile PFAS mass has been attributed to cationic and zwitterionic polyfluoroalkyl substances.^6,21,22,24,33^

While these insights are important for AFFF-impacted site assessments, broader trends in PFAA precursor distribution remains an understudied topic. Many field investigations focus on detailed characterizations of singular AFFF-impacted sites,^6,22,24^ though Adamson et al.^21^ did evaluate potential site-specific factors influencing PFAS fate and transport across three sites. Previous analyses were also limited by the availability of relevant analytical-grade PFAS standards crucial for accurate estimations of total PFAS mass. Although some key precursors (e.g., fluorotelomer sulfonates, perfluoroalkyl sulfonamides, etc.) can be quantified with standards,^5^ many identified AFFF-derived PFASs still lack commercial standards, forcing reliance on semiquantitative HRMS workflows and constraining quantitative comparability.^7,10,34^

Considering the role that AFFF-source zone soils play in releasing PFASs to groundwater, an understanding of both the identity and vertical migration of PFAAs and PFAA precursors through soil profiles is needed. Identifying these migration patterns under a variety of climatic and hydrogeologic conditions would also help guide site investigation efforts. However, because guidance on soil screening^35,36^ often emphasizes surface soils (0–30 cm bgs),^37^ investigations risk incomplete capture of PFAS diversity. Depth-resolved, multisite data sets are needed to quantify all PFASs across surficial and vadose zone intervals, as it remains unclear as to whether analysis of the first 30 cm of a source zone soil provides sufficient information on the amount and types of PFASs present. As only a few sites have been subjected to detailed characterization, it is also unclear whether the presence of specific PFASs can be predictive of the presence of other PFASs. Such information can be used to help guide site investigation and remediation efforts.

Addressing these gaps is essential for effective site characterization, risk assessment, and development of remediation strategies at AFFF-contaminated sites. The objective of this study was to evaluate the prevalence and vertical distribution of AFFF-derived PFASs in shallow soil cores collected from 12 different AFFF-impacted source zones at 10 different U.S. Department of Defense (U.S. DoD) sites. Soil cores were collected within the top 2 m of the vadose zone at sites representing six different climatic regions. Using soil extraction methods intended to capture the broad diversity of PFASs present in AFFF-impacted soils,^25,31,32^ depth-resolved soil samples were extracted and analyzed via LC-HRMS using 63 PFAS standards (including 10 cationic/zwitterionic PFASs) with targeted and semiquantitative analysis. Correlational analyses between individual PFASs across all samples were also conducted. This work is intended to inform prioritizations of PFAA precursors for future investigations and ultimately guide more targeted assessment and remediation efforts at AFFF-impacted sites.

## 2 Materials and Methods

### 2.1 Materials

Analytical standards were obtained from Wellington Laboratories (Guelph, Ontario, Canada), SynQuest Laboratories (Alachua, Florida, U.S.), and Chiron (Trondheim, Trøndelag, Norway). Isotopically labeled standards were obtained from Wellington Laboratories (Guelph, Ontario, Canada). Details of analytical and isotopically labeled standards are provided in Table S2. All solvents used were Optima LC/MS grade and purchased from Fisher Scientific (Hampton, NH), unless otherwise stated. All reagents used were purchased from Fisher Scientific; these include acetic acid (Optima LC/MS grade, *aq*), formic acid (Optima LC/MS grade, *aq*), ammonium acetate (Optima LC/MS grade, *aq*), ammonium hydroxide (Optima LC/MS grade, *aq*), hydrochloric acid (Trace Metal grade, *aq*), and sodium acetate anhydrous (ACS grade, *s*).

### 2.2 Site and Core Information

The ten U.S. DoD sites (S01–S10) evaluated in this study are described in detail in Text S1, and key information is summarized in Table 1. Sites were selected based on previous screening investigations confirming historical releases of AFFF and evidence of PFASs in soil and/or groundwater. All sites except S08 and S09 are current or former fire training areas (FTAs). S08 is a fire station where testing and repair activities led to AFFF discharges, and S09 is adjacent to buildings that contained aboveground (potentially leaky) AFFF storage tanks for fire suppression systems. Full descriptions of S01 and S10 are provided in Bigler et al.^38^ and Pritchard et al.,^22^ respectively.

**Table 1:** Summary of Relevant Site and Core Information

site	core	location in the U.S.	depth-averaged composite soil texture of core	depth to groundwater, m bgs	average annual precipitation, cm	groundwater recharge rate, cm/yr
S01	C01	Mountain Southwest	sandy clay loam	106	30	3
S02	C02	Mountain West	fine sand	11	48	4
S03	C03	West South Central	sandy clay loam	16	82	4
S04	C04A	Pacific Southwest	very fine sand	18	51	1
	C04B		silty clay			
S05	C05	South Atlantic	very fine sand	1.5	129	21
S06	C06	South Mid-Atlantic	*not measured*	2	114	36
S07	C07	South Mid-Atlantic	clayey, silty, very fine sand	2	119	26
S08	C08	Pacific Northwest	silty, very fine sand	2	37	3
S09	C09A	East North Central	very fine sand	4.5	79	25
	C09B		very fine sand			
S10	C10	South Atlantic	sandy clay	2	125	23

### 2.3 Soil Collection

One or two cores were collected from each site in this study (Table 1) using commonly employed and equivalent depth-discrete sampling techniques. At S01, a 1.5 m soil boring was collected following Bigler et al.^38^ Briefly, a 15.2 cm diameter steel casing was advanced to 1.5 m below ground surface (bgs). The core was then split into 7.6 cm intervals which were then homogenized to produce 19 subsamples across its entire length, except for the second-from-top segment which was lost during processing. At the remaining sites, 11 cores of varying lengths were collected (Text S1; Pritchard et al.^22^) using 5.7 cm steel casings advanced by direct push technology (DPT). Cores from S02, S03, S05, and S09 were advanced to 1.5 m bgs and split into ten 15 cm homogenized intervals. Two cores from S04 were advanced to ∼1.5 m bgs and split into nine or ten 15 cm homogenized intervals, respectively. Cores from S06 and S07 were advanced to 0.6 m bgs or 1.2 m bgs and split into six or 12 homogenized intervals of varying length, respectively. The core from S08 was advanced to 0.9 m bgs and split into six 15 cm homogenized intervals. The core from S10 was advanced to 1.8 m bgs and split into six 31 cm homogenized intervals. Soil samples were shipped on ice to the Colorado School of Mines and stored at −20 °C until processing. Soils were air-dried, homogenized using a mortar and pestle, and sieved to <2 mm. Water content was separately determined by drying additional aliquots of the soils for 24 h at 105 °C.

Site metadata was collected from various online databases [EPA EnviroAtlas; Air Force Civil Engineer Center (AFCEC) Comprehensive Environmental Response, Compensation, and Liability Act (CERCLA) Administrative Record] and included the depth to groundwater, lithology, average annual precipitation, and average groundwater recharge rate. Due to a limited soil mass for many intervals, soil pH (Table S1) was measured only for select shallow segments, whereas total organic carbon (TOC) was measured for all shallow samples.

### 2.4 PFAS Analysis

Soil samples collected from C01 were extracted via the method described in Nickerson et al.^31^ Briefly, 0.5 g aliquots of soil were added to 50 mL polypropylene centrifuge tubes, spiked with 4 ng of extracted internal standards, and extracted twice with 4 mL of 0.1% (v/v) ammonium hydroxide in methanol and twice with 4 mL of 0.5 M hydrochloric acid in methanol. Each extraction consisted of 30 s of vortexing, 15 min of sonication, and 20 min of centrifugation at 2470 rcf. Basic and acidic extracts were cleaned separately using ENVI-Carb solid-phase extraction (SPE) cartridges (Supelclean ENVI-Carb, 250 mg/6 mL; MilliporeSigma) using an elution procedure described previously.^31^ Acidic extracts were neutralized with a 1:1 (v/v) ammonium hydroxide to methanol solution. Both extracts were evaporated to dryness under nitrogen at 30 °C, then reconstituted and combined using 1.5 mL of 1% (v/v) acetic acid in methanol. Reconstituted extracts were stored at −20 °C overnight to promote salt precipitation and were then centrifuged at 17,000 rcf for 10 min. Final vials contained 80:20 (v/v) methanol to water and 750 ng/L of nonextracted internal standard. Unlike the prior characterization work on samples from C01,^38^ the soil extracts in this study were analyzed by LC-HRMS, as described below.

Soil samples collected from C02–C10 were extracted via the BAMBINO method described in Gonda et al.,^32^ which also includes a detailed analysis of the PFASs missed when employing the existing EPA Method 1633 (both its extraction and cleanup conditions as well as its specified analytes). The BAMBINO method was developed to provide a broad analytical window comparable to the method described above while using a cleanup procedure aligned with EPA Method 1633; resultant data from the two extraction methods are essentially equivalent for the EPA Method 1633 analytes.^32^ Briefly, 0.5 g aliquots of soil were added to 50 mL polypropylene centrifuge tubes and spiked with 42 ng of extracted internal standards. They were extracted three times with 0.3% (v OH^–^/v) ammonium hydroxide in methanol (2.5 mL, 3.75 mL, and 1.25 mL) and three times with 0.5 M hydrochloric acid (2.5 mL, 3.75 mL, and 1.25 mL). Extractions involved 30 s of vortexing followed by centrifugation at 2470 rcf for 10 min. Acidic extracts were neutralized with a 1:1 (v/v) ammonium hydroxide to methanol solution and centrifuged again at 2470 rcf for 10 min. Basic and acidic extracts were combined in equal volumes and cleaned using Strata PFAS SPE cartridges (WAX/GCB, 200 mg/50 mg/6 mL; Phenomenex) following previously described elution procedures.^32^ SPE extracts were neutralized with 25 μL acetic acid and final sample vials contained 1:1 (v/v) methanol to 20 mM ammonium acetate in water and 667 ng/L of nonextracted internal standard.

Both extraction methods use strong acids and bases, which enhances the recovery of zwitterionic and cationic PFASs.^31^ However, limited interconversion of certain PFASs may occur. In composite soil experiments using the second extraction and cleanup method described above, Gonda et al.^32^ appreciable conversion of only one anionic suspect PFAS was observed, suggesting any interconversion would be limited and outweighed by the enhanced recovery of the zwitterionic and cationic PFASs.

Each extract was analyzed for 63 targeted PFASs (with reference standards) and 5324 semiquantifiable suspect PFASs from a custom extracted ion chromatogram (XIC) list derived from the National Institute of Standards and Technology (NIST) PFAS list.^39^ Analysis was performed via liquid chromatography quadrupole time-of-flight mass spectrometry (LC-QToF-MS).^31,40^ Briefly, samples were injected onto a SCIEX ExionLC high-performance liquid chromatography (HPLC) system and separated using a 20 mM ammonium acetate/methanol gradient. Mass spectra were collected using a SCIEX X500R QToF-MS (Framingham, Massachusetts, U.S.) operated in both electrospray negative (ESI−) and electrospray positive (ESI+) modes. Data for target, suspect screening, and semiquantification analyses were acquired and processed in SCIEX OS 3.4.5 with in-house MATLAB scripts (R2024b Update 5).

Target analytes were confirmed by retention time (RT) and accurate mass as compared to analytical standards and assigned a PFAS Confidence in Identification (PCI) level of 1a/1b based on Charbonnet et al.^34^ Unknown PFASs meeting molecular ion mass accuracy, isotopic pattern-match, and library-match criteria were also identified. Reported suspect features had confidence levels between 2a–3d,^34^ therefore some PFASs with lower quality MS data may have been be missed. For the semiquantitative analysis of suspect analytes, concentrations were estimated with a target calibrant-internal standard pair selected based on ionizable functional group and fluoroalkyl chain length.^22^ Additional analytical details and calculation procedures are provided in Texts S3 and S4.

## 3 Results and Discussion

### 3.1 Detection Frequency of PFAS Classes

Across all cores and samples, a total of 162 different PFASs representing 50 classes were identified (PCI levels 3d or higher) in at least one soil sample. Class-level detection frequency was visualized by grouping all target and suspect identifications into PFAS classes (Figure 1). C01 was excluded because its depth segmentation, specifically for the shallow depth, was not directly comparable to the other cores. This analysis was intended to identify PFAS classes that would have been missed if only the shallowest soil sample was analyzed. Here, the “shallowest” segment refers to the first 10–30 cm of depth from the surface (core-specific lengths are provided in Table S1). Figure 1 shows broad PFAS diversity across sites, with notable exceptions being C09A and C09B. Classes detected across nearly all sites include perfluoroalkyl carboxylic acids (PFCAs), perfluoroalkyl sulfonic acids (PFSAs), and perfluoroalkyl sulfonamides (FASAs). This is important, as while many PFCAs and PFSAs are included in EPA Method 1633, only the C8 FASA (FOSA) is additionally included, despite frequent detection of the C3–C7 FASAs across cores. While this pattern is consistent with electrochemical fluorination (ECF)-based AFFF inputs, X:2 fluorotelomer sulfonates (X:2 FTSs) – indicative of use of fluorotelomer (FT)-based AFFFs – were also detected at nearly all sites. Related fluorotelomer-based classes such as X:2 fluorotelomer sulfinates (X:2 FTSi’s) and X:2 unsaturated fluorotelomer sulfonates (X:2 UFTSs) were also frequently detected. Taken together, these observations suggest that both FT-based and ECF-based AFFFs were used at nearly all sites.

**Figure 1:**
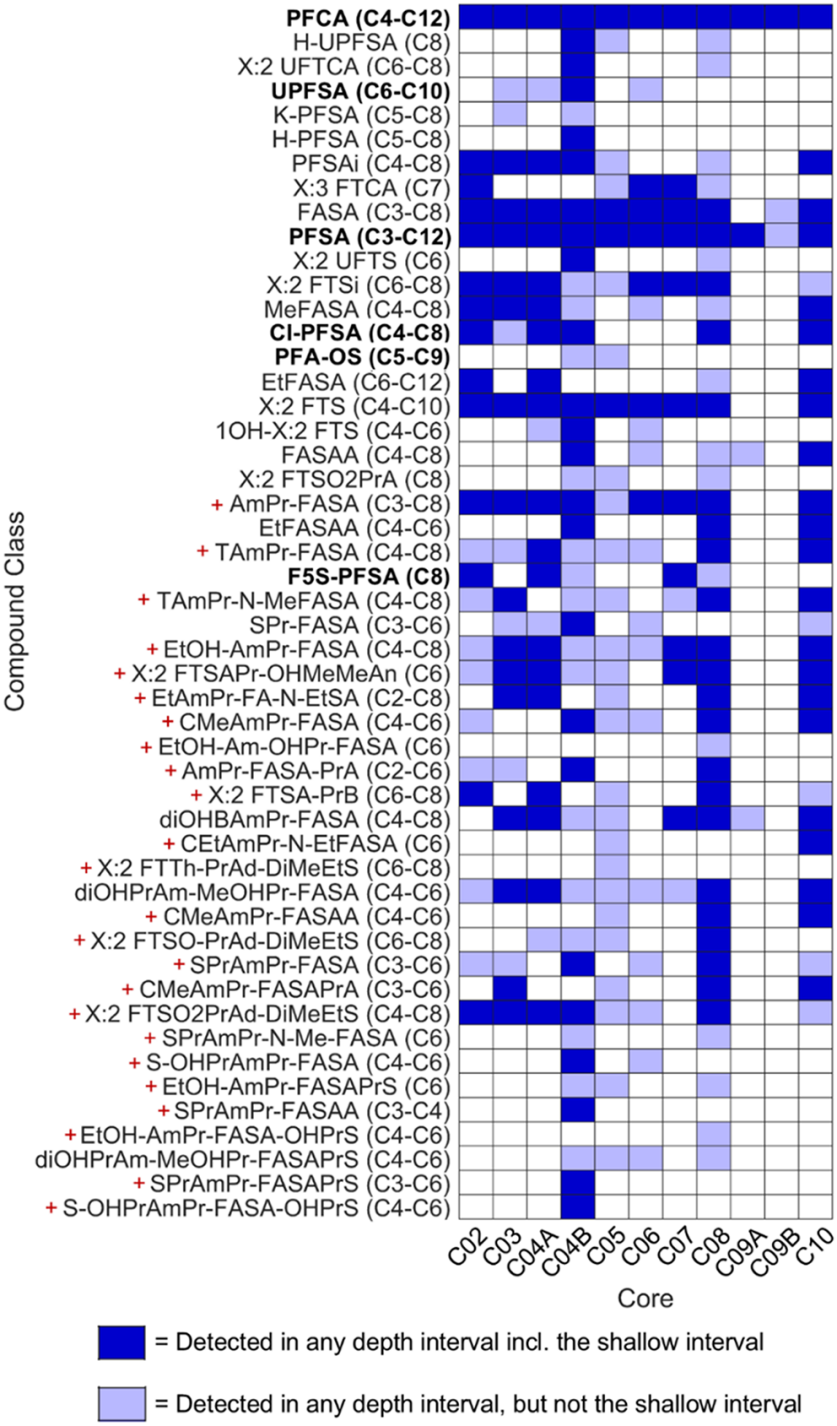
Detection frequency heatmap of PFAS classes found across C02—C10. Only concentrations above reporting limits were used. Nonshallow detections, meaning a member of the PFAS class exceeded its reporting limit in at least one depth interval *except* the shallowest of the core, are represented with light-blue squares. Shallowest-depth detections, meaning a member of the PFAS class exceeded its reporting limit in at least one depth interval *including* the shallowest of the core, are represented with dark-blue squares. Each class label was annotated with the observed chain-length range present in the data set. Classes in bold indicate PFAA classes. Classes with a red “+” symbol indicate cationic/zwitterionic species. A listing of all acronyms for all classes is provided in Tables S2 and S3.

The extent to which PFAS class diversity was captured in the shallowest interval is summarized further in Table 2. For eight of the eleven cores included in this analysis, shallow detections represent the majority (≥50%) of class-level diversity observed across the full core, whereas for the remaining three cores only ∼14–35% of the measured classes were present in the shallowest interval. Interestingly, two of these three cores (C05 and C06) were from sites with moderate to high average annual precipitation (>100 cm/year) and higher groundwater recharge rates, which could explain a greater depth penetration for a larger variety of PFASs. However, C07 and C10 were also from sites with moderately high average annual rainfall and recharge, yet shallow intervals captured >80% of the class-level diversity. This suggests that rainfall and recharge rate are not the sole factors determining PFAS transport in shallower soil depths. Other contributing factors likely include TOC of the shallow soils as well as pH. Shallow-interval TOC measurements ranged from <0.2–4.9% (see Table S1), with those in C05 and C06 falling in the middle to lower end of this range (0.36 and 0.59%, respectively). While the greater shallow-segment depth and clay content of C10 may help explain its higher apparent shallow retention of PFASs (its pH and TOC were similar to those of C06), C07′s shallowest interval exhibited higher TOC (1.3%) and a lower pH (4.8 versus ∼7.5 at many sites): both higher TOC and lower pH have been associated with greater PFAS retention on soils.^18,41^ Collectively, these data suggest that in the *absence* of higher organic carbon or clay and/or lower pH in the shallow soils, higher average annual precipitation may be associated with deeper penetration of PFASs through the soil profile, though it is clear that a variety of other factors likely impact PFAS retention in soils.^22,24^

**Table 2:** Total Class and Cationic/Zwitterionic Class Detection Frequencies across Cores C02 through C10

site	core	total # classes detected	% of all classes detected in shallowest-depth^a^	total # cationic/zwitterionic classes detected	% of cationic/zwitterionic classes detected in shallowest-depth^a^
S02	C02	22	63.6	11	27.3
S03	C03	23	69.6	10	70.0
S04	C04A	23	82.6	8	87.5
	C04B	40	60.0	16	56.3
S05	C05	28	14.3	15	0.0
S06	C06	20	35.0	7	14.3
S07	C07	13	84.6	4	75.0
S08	C08	38	60.5	18	77.8
S09	C09A	4	50.0	0	n/a
	C09B	3	33.3	0	n/a
S10	C10	27	81.5	13	76.9

a Lengths and diameters associated with each cores’ “shallowest-depth” may be found in Table S1.

\Regardless, for nine of the eleven cores, PFAA precursor classes account for at least 50% of all shallowest depth detections for each core (ranging from ∼50 to 88%), indicating that precursors generally dominate PFAS class diversity in shallow soils. This analysis did not assess whether these precursors were originally present in the AFFF formulations or were formed as a result of other precursor transformation, the latter of which would be consistent with a subsurface presence but absence from the shallowest interval. More importantly, these data suggest that sampling the first ∼30 cm of soil should capture the widest range of PFASs for most sites, consistent with common soil sampling guidance.^37^ Further, correlational analyses of PFASs within a class across all samples (Figures S164–S190) generally indicate that chain-length variants within a class are generally correlated with one another, suggesting that detection of one member of a class increases the likelihood that other chain lengths are also present.

### 3.2 Cross-Core Comparison of PFAS Contributions

In two previous studies, Nickerson et al.^6^ and Pritchard et al.^22^ reported very high proportions (up to 97%) of cationic/zwitterionic PFAA precursor mass found within the shallowest depth interval of AFFF-impacted soil cores. The diversity assessment above is consistent with these findings in terms of detection frequency: of the nine cores where cationic/zwitterionic precursors were detected, six have a majority (>50%) of them in the shallowest interval. However, these prior assessments emphasize that it is not only the chemical diversity that matters, but also which PFASs constitute the majority of PFAS mass across a soil profile. Herein, the relative contribution of individual PFASs to total detected PFAS mass (measured and/or estimated by HRMS) was calculated. For each core, the top five PFASs by relative mass contribution were identified and the combined set of these PFASs across all cores is provided in Figure 2. PFASs not associated in any cores’ top five were grouped into a separate “other” category, which constituted between 3% and 41% across the cores shown. C09A and C09B were excluded due to low PFAS mass per unit core (<10^3^ ng/cm^3^) and limited chemical diversity (<10 unique PFAS classes). Each PFAS contribution is annotated by chemical type: compounds are identified as precursor vs PFAA, and precursors are further classified as ECF- or FT-based. Most importantly, Figure 2 highlights which PFASs are included in EPA Method 1633 and which of those not in EPA Method 1633 currently have commercially available analytical standards.

**Figure 2:**
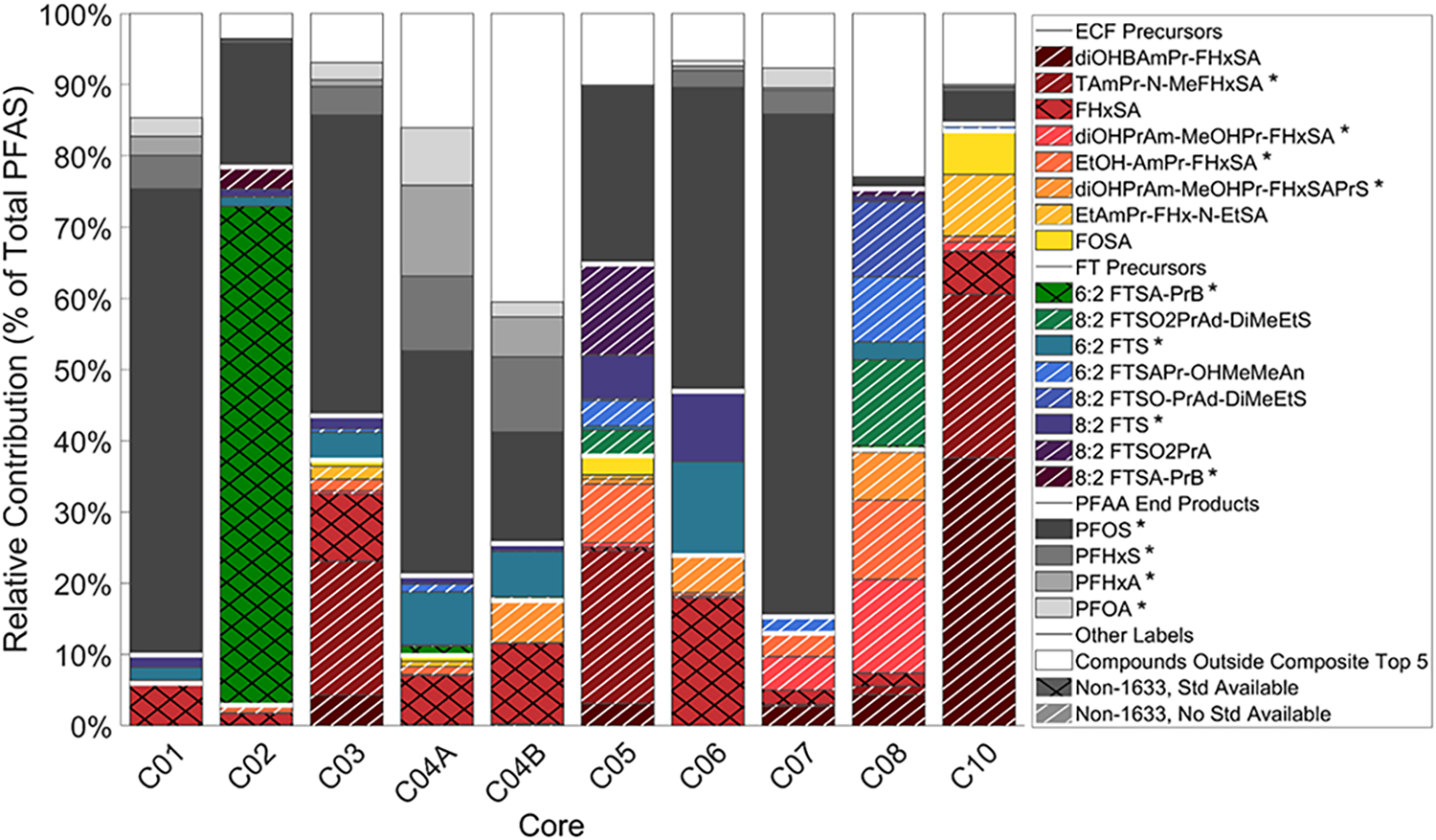
Stacked bar chart representing relative contributions of abundant PFASs across each core, excluding C09A and C09B. Black, cross-hatched bars indicate non-EPA Method 1633 analytes with commercially available standards whereas white, diagonal-hatched bars indicate non-EPA Method 1633 analytes currently *without* commercially available standards. Asterisked compounds in the legend indicate PFASs found in AFFF formulations.^5,7,10,11,13,42^

Figure 2 highlights several important site-specific differences. While the composite top five PFAS list is dominated by precursors, not all cores exhibit majority (>50%) precursor contributions (see also Table 3). For instance, C01 and C07 are strongly PFAA-dominant, with over 80% of PFAS mass comprised of PFAAs. Among cores with >70% precursor contributions, precursors are predominantly ECF-based (i.e., C10), but C02 (from a current FTA, Text S1) is almost entirely FT-derived. Other cores exhibit a mixture of both precursor families, such as C08, which shows roughly equal contributions from FT and ECF precursors. An interesting observation in PFAS mass distributions can be seen in C04A and C04B, which are two cores collected from different AFFF sources zones at the same installation (Text S1). Their overall PFAS distributions are similar relative to those of all other cores. However, slight differences are apparent in the contributions of certain PFASs. For example, the PFOS contribution to the total PFAS mass is 31% in C04A versus only 16% in C04B. C04B exhibits a larger fraction of its PFAS mass coming from less frequently detected PFASs (i.e., compounds outside of the composite top five is 41% for C04B versus 16% for C04A). Collectively, these patterns likely reflect differences in historical AFFF formulations or even differences in application frequency and/or aging.

**Table 3:** Summary of PFAS Mass Contributions in Each Core

core	total PFAS mass per unit core, ng/cm^3^	PFASs not quantifiable by EPA Method 1633 (%)	ECF-based precursor contribution (%)	FT-based precursor contribution (%)	PFAA contribution (%)
C01	4.16 × 10^5^	10.9	8.90	4.40	87.1
C02	7.45 × 10^4^	78.2	4.70	76.2	19.2
C03	3.03 × 10^4^	41.4	41.5	6.40	52.1
C04A	1.97 × 10^4^	18.9	12.7	15.4	72.1
C04B	2.92 × 10^5^	47.7	42.7	11.3	46.1
C05	3.77 × 10^4^	64.6	46.2	27.7	26.1
C06	3.31 × 10^5^	28.8	28.6	23.4	48.0
C07	3.17 × 10^4^	16.7	13.8	3.27	83.0
C08	2.41 × 10^5^	92.9	56.2	40.2	3.60
C09A	2.33 × 10^2^	20.2	20.2	0.00	79.8
C09B	2.30 × 10^2^	0.60	1.00	0.00	99.0
C10	3.92 × 10^5^	87.7	93.3	1.00	5.70

This analysis also indicates that reliance on EPA Method 1633-target analytes alone would substantially underestimate precursor mass contributions, potentially obscuring their importance relative to PFAAs (see Figure S192). C02 stands out as an extreme case: nearly all precursor mass would go unrecognized if only EPA Method 1633-listed compounds were measured. More broadly, across all 12 studied cores, at least half of the precursor burden (within the sampled depths) would not be captured via target analysis alone. While semiquantitation can provide estimates, these results reinforce the importance of expanding standard availability for abundant AFFF-derived precursors frequently observed in soils. Together, these findings have two implications. First, the precursor dominance at many sites supports previous reports of precursors constituting a substantial fraction of PFAS source-zone mass, which have the potential to drive long-term PFAA releases (upon their transformation to PFAAs) to groundwater.^6,22,43,44^ Second, the substantial gap between target-only and estimated precursor contributions highlights a critical challenge for site assessments relying on EPA Method 1633 alone, a sentiment shared with previous field-scale characterizations utilizing depth-discrete soil sampling at AFFF-impacted sites.^6,22,23^

Going beyond EPA Method 1633 is important in supporting decision-making for risk assessors at these types of sites. Specifically, one may want to consider the PFASs identified in Figure 2 which are not included on the EPA Method 1633 analyte list (Table 4). Site investigations using EPA Method 1633 could easily improve the scope of their assessment by including compounds such as FHxSA and 6:2 FTSA-PrB (6:2 FTAB) on their target analyte list since standards for these are commercially available. Though not present in great abundance compared to other precursors in Figure 2, FHxSA is present across all studied cores, which supports the findings of previous AFFF-impacted site characterizations.^6,21,22,43^ In contrast, 6:2 FTSA-PrB is not found across all studied cores, though it may be present in great abundance at sites with more recent FT-based AFFF use. Given that both FHxSA and 6:2 FTSA-PrB can both be detected via ESI- mass spectrometry, their addition to the ESI- EPA Method 1633 list should require minimal modifications. Finally, compounds listed in Table 4 without commercial standards represent priorities for standards development to support structural confirmation and improved quantitation.

**Table 4:** Suggested Prioritization List of AFFF-Derived PFAA Precursors for Further Inquiry^a^

compound acronym	neutral molecular formula	charge	manufacturing process	commercial standard currently available?
diOHPrAm-MeOHPr-FHxSAPrS	C_18_H_27_F_13_N_2_O_8_S_2_	anionic	ECF	no
diOHPrAm-MeOHPr-FHxSA	C_15_H_21_F_13_N_2_O_5_S	anionic	ECF	no
diOHBAmPr-FHxSA	C_15_H_21_F_13_N_2_O_4_S	anionic	ECF	no
TAmPr-N-MeFHxSA	C_13_H_17_F_13_N_2_O_2_S	cationic	ECF	no
EtAmPr-FHx-N-EtSA	C_15_H_21_F_13_N_2_O_2_S	cationic	ECF	no
EtOH-AmPr-FHxSA	C_13_H_17_F_13_N_2_O_3_S	zwitterionic	ECF	no
FHxSA	C_6_H_2_F_13_NO_2_S	anionic	ECF	yes
8:2 FTSO2PrAd-DiMeEtS	C_17_H_18_F_17_NO_6_S_2_	zwitterionic	FT	no
8:2 FTSO-PrAd-DiMeEtS	C_17_H_18_O_5_S_2_NF_17_	zwitterionic	FT	no
8:2 FTSO2PrA	C_13_H_9_F_17_O_4_S	anionic	FT	no
8:2 FTSA-PrB	C_17_H_19_F_17_N_2_O_4_S	zwitterionic	FT	no
6:2 FTSA-PrB	C_15_H_19_F_13_N_2_O_4_S	zwitterionic	FT	yes
6:2 FTSAPr–OHMeMeAn	C_13_H_17_F_13_N_2_O_3_S	cationic	FT	no

a A list of all acronyms is provided in Tables S2 and S3.

### 3.3 Vertical Distribution of Select PFASs across Cores

At most AFFF-impacted sites, the discharge of PFASs from the vadose zone to groundwater is of primary concern. Thus, understanding how the concentrations of AFFF-derived precursors change with depth can provide insight into PFAS transport and potential long-term PFAA loading. Vertical distribution profiles of all PFASs were generated as part of this analysis (Figures S3–S163). Figure 3 presents representative profiles of C6 and C8 PFCAs (PFHxA, PFOA), PFSAs (PFHxS, PFOS), X:2 FTSs (6:2 FTS, 8:2 FTS), and FASAs (FHxSA, FOSA) from select cores spanning an array of geographic locations at sites with differing depths to groundwater, annual average rainfall, etc. (Table 1). Across all studied cores, no single consistent trend was apparent in the vertical distribution of the concentrations of the PFCAs, PFSAs, X:2 FTSs, and FASAs. However, these PFASs were consistently detected throughout each studied core, with the notable exception of C07 and the low-concentration cores C09A and C09B.

**Figure 3:**
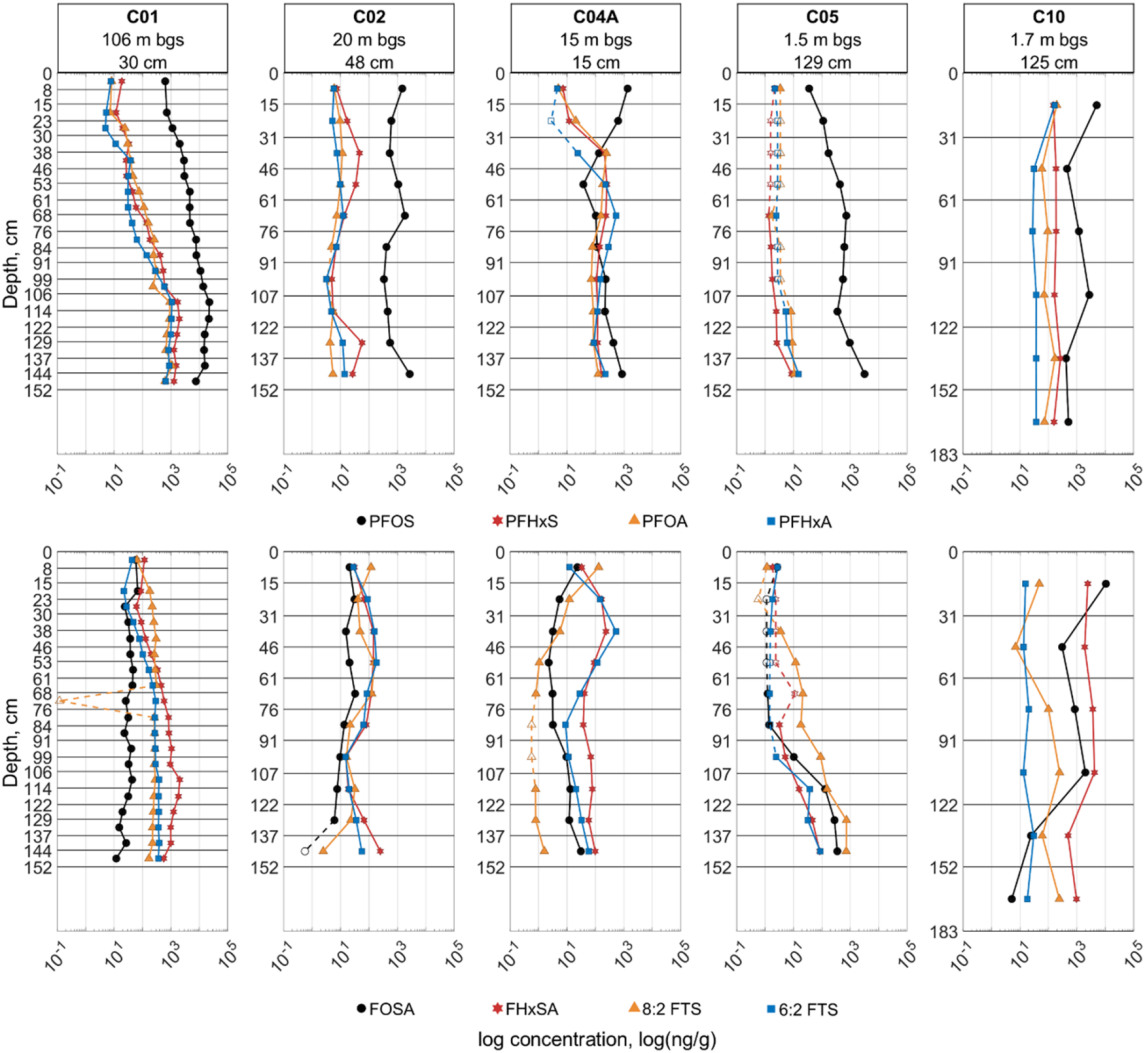
Vertical distribution profiles of select PFAAs (top row) and PFAA precursors (bottom row) in select cores. Concentration is shown on a log-base-10 scale. Headings for each column include estimated depth to groundwater (m below ground surface) and average annual precipitation (cm) at each site. For any given plot, open markers with dashed-line connectors represent sampled depth intervals where the compounds’ concentration was below the reporting limit – the location of the open marker along the *x*-axis is representative of those reporting limits.

Though the PFAA profiles in C01 appear to show that PFAA concentrations are strongly and positively correlated with depth (illustrated in Figure 3), the profiles in C02, C04A, and C10 do not indicate strong positive or negative trends. In addition, precursor profiles generally do not match those of the PFAAs within a given core, which may suggest differences in transport and/or transformation dynamics between these broad classes. It is important to clarify that these profiles only represent the top ∼2 m of soil – the distribution of PFASs below these depths was not explored in this study.

The profile for C04A is notably different from the other cores, possibly because S04 was the most arid site sampled. In this core, several compounds display abrupt increases or decreases over depth intervals in the upper tens of centimeters followed by more gradual increases with depth. This profile might be reflective of low annual precipitation and groundwater recharge rate at this site, which may produce infrequent and short infiltration events relative to the timing of AFFF application. A more detailed assessment of the transport processes at these sites would be needed to more definitively explain the observed profiles.

To more closely examine precursor-product relationships, Figures 4 and 5 show distributions of PFHxA and PFHxS alongside their measured precursors. Though these PFASs are present in other cores, these featured cores generally contain a broader array of precursors to these PFAAs. For example, C01 contained PFHxA and five precursors to PFHxA, including 6:2 FTSA-PrB, though it is only present in the first 23 cm of the core (and at a higher concentration than the other PFASs shown). C02 also shows elevated shallow 6:2 FTSA-PrB concentrations, but its concentration decreases less rapidly with depth than in C01. This may indicate that an AFFF formulation high in 6:2 FTSA-PrB was applied with greater frequency or released more recently at C02 as compared to at C01. In the correlational analysis (Figure 6), the concentrations of 6:2 FTSA-PrB did not show strong positive or negative correlations with those of any other PFASs in the panel. In contrast, among the FT precursors, PFHxA shows its strongest correlation with 6:2 FTS (Figure 6), despite these compounds being separated by multiple transformation steps (with 6:2 FTS often considered a semistable intermediate).^12,46,47^ A similar matrix featuring PFOA and its measured precursors (Figure S191) also suggests strong relationships between PFOA and 8:2 FTS. The fact that 6:2 FTS is a semistable intermediate may also help explain its strong correlation with 6:2 FTSO2PrAd-DiMeEtS, as the 6:2 FTS would likely be a semistable marker if the latter was formed from 6:2 FTSAS (6:2 FtTAoS, 6:2 FTTh-PrAd-DiMeEtS). Though the strong vertical gradient for 1OH-6:2 FTS in C04B (Figure 4) might suggest a negative correlation with 6:2 FTS (as the 1OH-6:2 FTS is likely formed from the oxidation of 6:2 FTS, Scheme S1), relatively strong positive correlations are observed for these two compounds (Figure 6).

**Figure 4:**
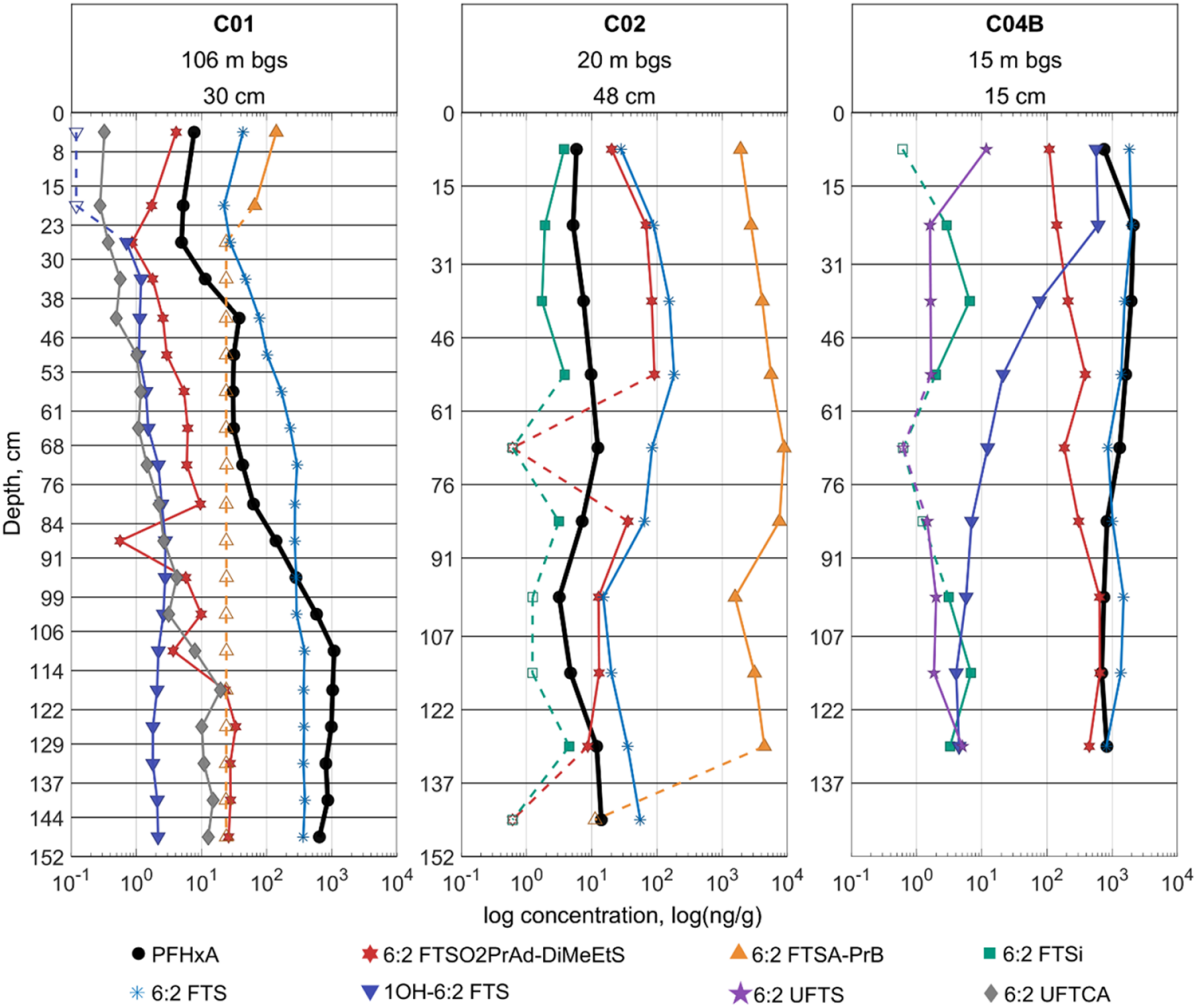
Vertical distribution profiles of PFHxA and a few of its precursors. Concentration is shown on a log-base-10 scale. Headings for each column include estimated depth to groundwater (m below ground surface) and average annual precipitation (cm) at each site. A global legend is used for convenience, but not all labeled compounds were detected in each of the plotted cores. C01 did not contain 6:2 FTSi nor 6:2 UFTS. C02 did not contain 1OH-6:2 FTS, 6:2 UFTS, nor 6:2 UFTCA, while C04B did not contain 6:2 FTSA-PrB (6:2 FTAB), nor 6:2 UFTCA. For any given plot, open markers with dashed-line connectors represent sampled depth intervals where the compounds’ concentration was below the reporting limit–the location of the open marker along the *x*-axis is representative of those reporting limits.

**Figure 5:**
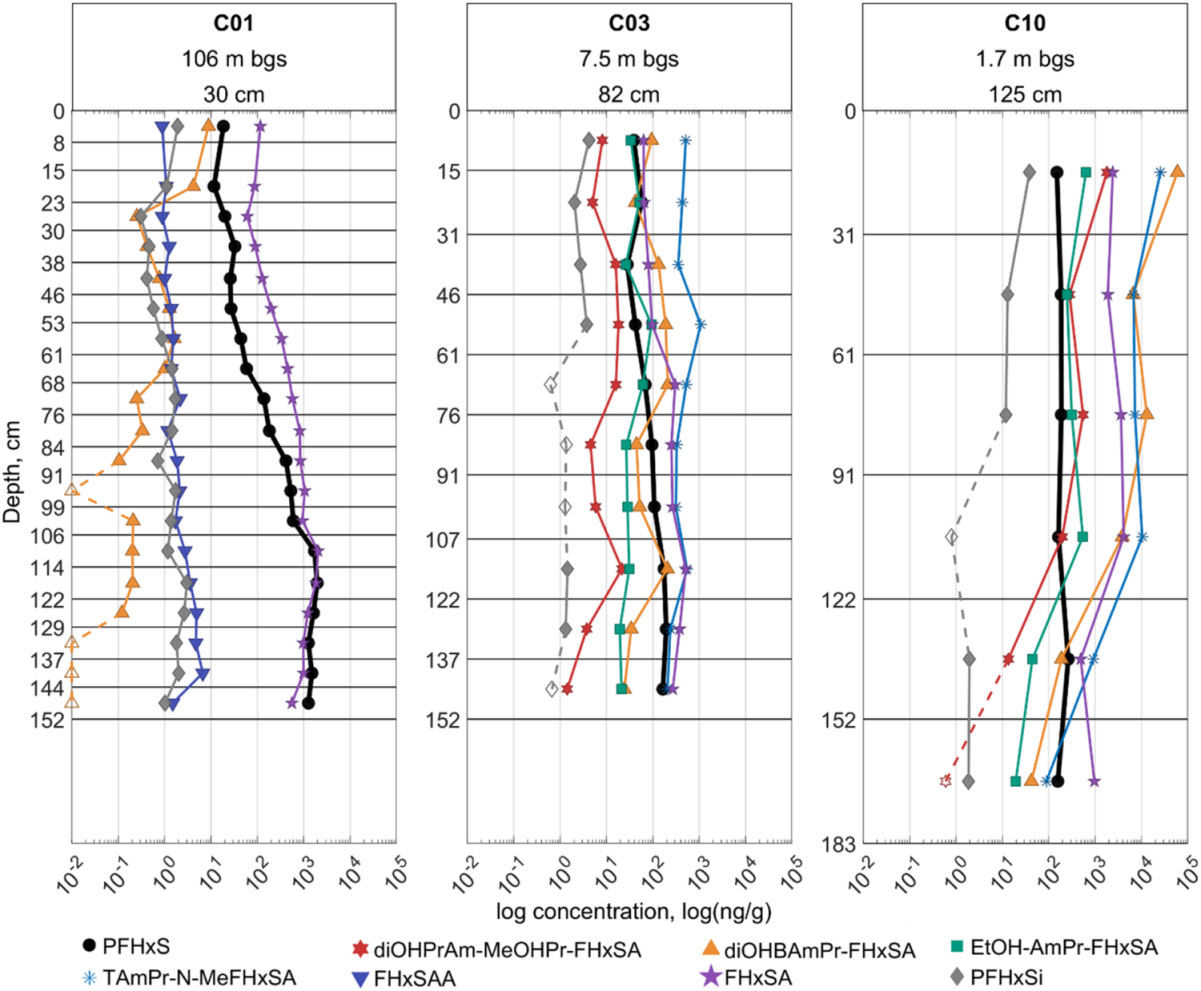
Vertical distribution profiles of PFHxS and a few of its precursors. Concentration is shown on a log-base-10 scale. Headings for each column include estimated depth to groundwater (m below ground surface) and average annual precipitation (cm) at each site. A global legend is used for convenience, but not all labeled compounds were detected in each of the plotted cores. C01 did not contain diOHPrAm-MeOHPr-FHxSA, EtOH-AmPr-FHxSA, nor TAmPr-N-MeFHxSA. FHxSAA was not measured in C03 and C10. For any given plot, open markers with dashed-line connectors represent sampled depth intervals where the compounds’ concentration was below the reporting limit–the location of the open marker along the *x*-axis is representative of those reporting limits.

**Figure 6:**
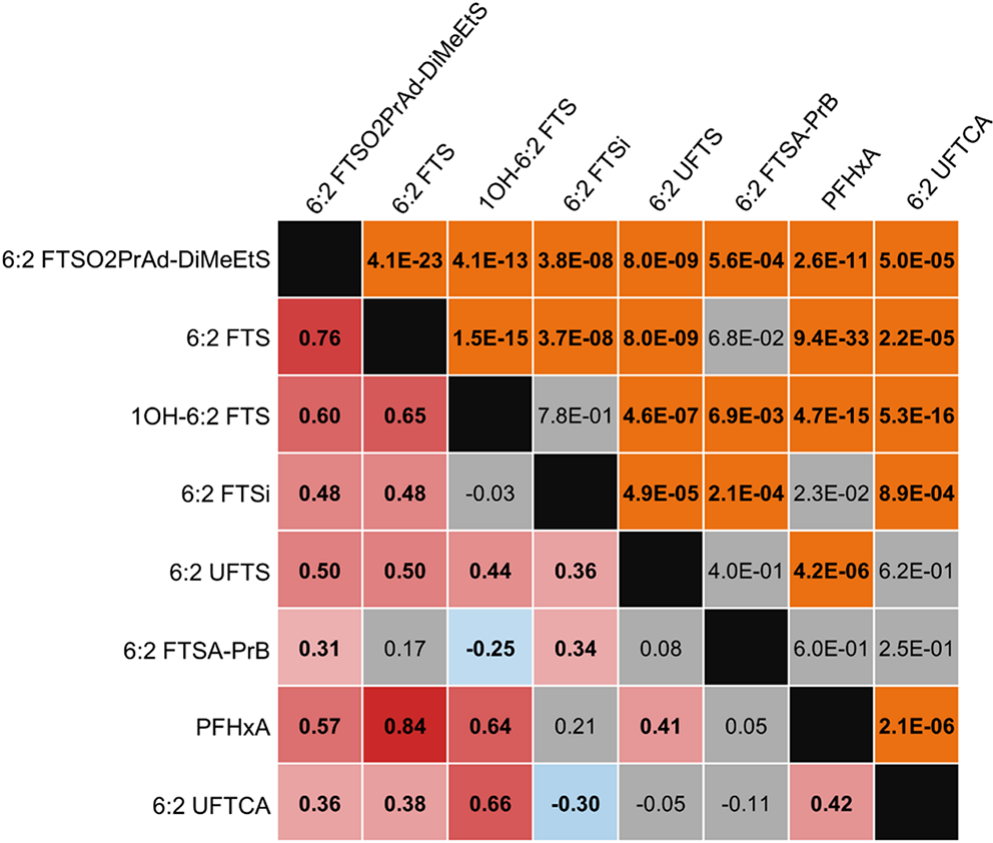
Correlation matrix of the compounds featured in Figure 4–PFHxA and select precursors. The analysis was conducted using data from all sampled intervals across all cores. Below the diagonal are Spearman’s rank correlation coefficients, ranging from 1 (red) to −1 (blue). Above the diagonal are corresponding *p*-values, where *p*-values above 0.01 are shaded gray (statistically insignificant) and those below 0.01 are shaded orange (strong statistical significance). Cells of correlation coefficients with insignificant *p*-values are also shaded gray.

For the ECF precursors shown in Figure 5, C01 shows a gradual increase in FHxSA concentration with depth to ∼106 cm bgs, followed by a decrease from ∼114 cm bgs to the bottom of the core. A similar trend in vertical distribution is evident for PFHxS in the same core. In contrast, the concentrations of FHxSAA and PFHxSi seem to remain relatively constant with depth, whereas those of diOHBAmPr-FHxSA gradually decreases with depth. C03 and C10 show similar overall behavior, where the concentrations of PFHxS and its precursors remain relatively constant throughout the entire length of the cores, though PFHxS does tend toward a slight increase with depth while the precursors tend toward a slight decrease. Across cores, concentrations of diOHBAmPr-FHxSA and diOHPrAm-MeOHPr-FHxSA are strongly correlated from interval to interval (Figure 7). The concentration of PFHxSi also appears to go below the reporting limit in the middle of C03 and C10 and remains at a low concentration further down. In C10, all shown precursors experience a decrease in concentration with depth to ∼122 cm. After 122 cm, concentrations of FHxSA increase slightly while those of all other precursors decrease to the bottom of the core. Concurrently, concentrations of PFHxS remain relatively stable before slightly decreasing with depth to the bottom of the core. The lines of evidence shown from Figures 3, 4, and 5 clearly suggest that transport alone may not be the sole factor determining PFAA or PFAA precursor concentration with depth – it is also largely important to consider potential transformation pathways and site-specific factors.

**Figure 7:**
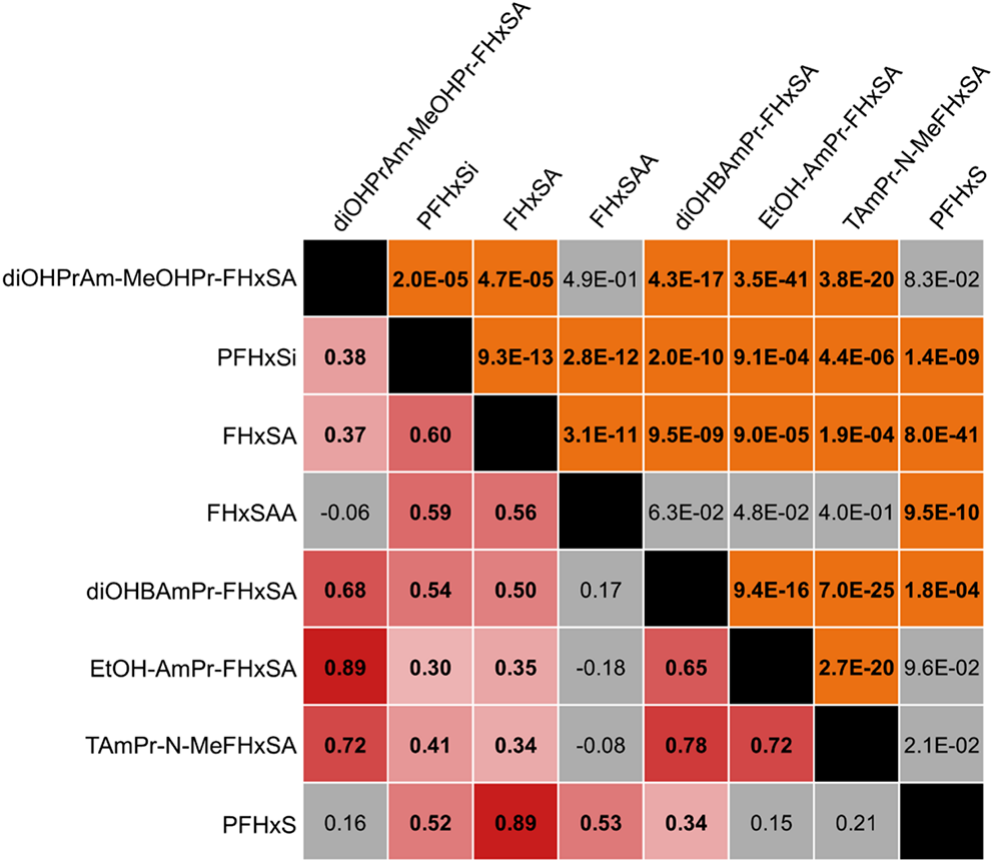
Correlation matrix of the compounds featured in Figure 5–PFHxS and select precursors. The analysis was conducted using data from all sampled intervals across all cores. Below the diagonal are Spearman’s rank correlation coefficients, ranging from 1 (red) to −1 (blue). Above the diagonal are corresponding p-values, where p-values above 0.01 are shaded gray (statistically insignificant) and those below 0.01 are shaded orange (strong statistical significance). Cells of correlation coefficients with insignificant p-values are also shaded gray.

Importantly, not all the precursors featured in Figures 4 through 7 can be directly traced back as components in AFFF formulations: only 6:2 FTS and 6:2 FTSA-PrB have been previously reported as components in FT-based AFFFs,^7,47,48^ while only diOHPrAm-MeOHPr-FHxSA, EtOH-AmPr-FHxSA, and TAmPr-N-MeFHxSA have been previously reported as components in ECF-based AFFFs.^49^ All other precursors reported herein have been tentatively identified as transformation intermediates from other “parent” precursors. For the FT precursors, several studies indicate that 6:2 FTSA-PrB (a component of Forafac 1157) will form PFHxA through various intermediates, including 6:2 FTS.^10,11,46^ Although 6:2 FTSAS, a parent precursor found in Ansul and Angus Fire AFFFs,^10,11^ was not detected in great abundance across the studied cores, one of its oxidation products, 6:2 FTSO2PrAd-DiMeEtS was detected frequently across cores. For the ECF precursors, the pathways leading to PFHxS formation (Scheme S2) are not as well documented.

The correlation matrices (Figures 6 and 7) nevertheless suggest systematic differences between FT and ECF precursor behavior. FT precursors of any one chain length (i.e., 6:2-based chemicals) may form multiple chain lengths of PFCAs,^11,47,50^ whereas ECF precursors of a given chain length only transform into PFSAs of the same chain length.^12,33,51^ These phenomena may be reflected in the differences in magnitude of the correlation coefficients. In general, Figures 6 and 7 show that correlations of PFHxA and its FT precursors are weaker than those between PFHxS and its ECF precursors. Additionally, one could expect correlations between PFASs that are one transformation step removed from each other would be stronger than those between PFASs that are multiple steps away. Though there are some examples where this is the case, there are also examples where the opposite is true. For example, PFHxS is more strongly correlated with FHxSA (ρ = 0.89) than with PFHxSi (ρ = 0.52; Figure 7), despite PFHxSi being an immediate precursor to PFHxS. This could be consistent with the notion that multiple transformation pathways may generate both PFHxS and FHxSA, whereas fewer pathways generate PFHxSi. In other words, the hypothesis that compounds that are one transformation step away from each other would exhibit stronger correlations would hold if single precursors each possessed their own pathways that generated a single, unique product. The absence of such correlations may indicate multiple precursors generating any one product and/or significant branching in pathways as transformations proceed.

## 4 Implications

To the authors’ knowledge, this is the first study to compare the prevalence, contributions, and vertical distributions of PFAAs and PFAA precursors in soils across multiple AFFF-impacted sites with a focus on polyfluoroalkyl species. These findings indicate that shallow-only soil sampling strategies, particularly if EPA Method 1633 is the selected analysis method, can miss important fractions of precursor diversity and PFAS mass at AFFF-impacted sites. When looking at a shallow soil core, precursor classes—often from both ECF and FT lineages—frequently dominate source-zone composition. Most importantly, monitoring programs that rely solely on EPA Method 1633 PFASs are at risk of under-estimating precursor mass at many AFFF-impacted locations. Accordingly, site characterization and risk assessments should, when possible, pair depth-resolved soil sampling with target and semiquantitative workflows to capture abundant AFFF-derived precursors, such as those identified here. Because PFAA and precursor concentrations exhibit site-specific vertical distribution behavior, the potential roles precursor transformation and other factors may play in PFAS transport and subsequent leaching to groundwater warrant further investigation.

One could posit that to better understand the impacts of PFAA precursors at AFFF-impacted sites, meticulous characterization of AFFF formulations is all that is needed. The results of this study clearly indicate that characterization of the actual PFASs present in AFFF-impacted media is perhaps equally important: though many polyfluoroalkyl substances present in AFFF are also abundant in AFFF-impacted soils, there are also many polyfluoroalkyl substances in these soils that most likely have formed from the AFFF components. A handful of studies have laid the groundwork in this effort by linking AFFF components to specific formulations, manufacturers, and timeframes,^5,7,10,11,13,42,49^ though additional transformation rate and pathway studies on these abundant precursors are clearly needed. Importantly, it remains unclear as to whether vertical distributions of PFASs in soil profiles can be linked to site-specific factors such as soil organic carbon, soil texture, porewater ionic strength, recharge rate, water table variability, and many other site-specific factors.^23,25,26,29,30,38,52^ Characterization and confirmation of polyfluorinated, AFFF-derived transformation intermediates can also help remedial investigations draw more concrete conclusions on potential sources of contamination. Collectively, the data from this study point toward a need for systematic evaluations of precursors that appear to be abundant at AFFF-impacted sites.

## Supplementary Material



## Notes

Additional details on site locations, studied cores, PFAS analysis, analyte lists, vertical profiles of all PFASs, correlation matrices, and schemes (PDF)

## References

[1] Moody C. A., Field J. A. (2000). Perfluorinated Surfactants and the Environmental Implications
of Their Use in Fire-Fighting Foams. Environ.
Sci. Technol..

[2] Moody C. A., Hebert G. N., Strauss S. H., Field J. A. (2003). Occurrence and Persistence
of Perfluorooctanesulfonate and Other Perfluorinated Surfactants in
Groundwater at a Fire-Training Area at Wurtsmith Air Force Base, Michigan,
USA. J. Environ. Monit..

[3] Guelfo J. L., Adamson D. T. (2018). Evaluation of a
National Data Set for Insights into
Sources, Composition, and Concentrations of per- and Polyfluoroalkyl
Substances (PFASs) in U.S. Drinking Water. Environ.
Pollut..

[4] Hu X. C., Andrews D. Q., Lindstrom A. B., Bruton T. A., Schaider L. A., Grandjean P., Lohmann R., Carignan C. C., Blum A., Balan S. A., Higgins C. P., Sunderland E. M. (2016). Detection
of Poly- and Perfluoroalkyl Substances (PFASs) in U.S. Drinking Water
Linked to Industrial Sites, Military Fire Training Areas, and Wastewater
Treatment Plants. Environ. Sci. Technol. Lett..

[5] Schultz M. M., Barofsky D. F., Field J. A. (2004). Quantitative Determination of Fluorotelomer
Sulfonates in Groundwater by LC MS/MS. Environ.
Sci. Technol..

[6] Nickerson A., Rodowa A. E., Adamson D. T., Field J. A., Kulkarni P. R., Kornuc J. J., Higgins C. P. (2021). Spatial Trends of Anionic, Zwitterionic,
and Cationic PFASs at an AFFF-Impacted Site. Environ. Sci. Technol..

[7] Place B. J., Field J. A. (2012). Identification of
Novel Fluorochemicals in Aqueous
Film-Forming Foams Used by the US Military. Environ. Sci. Technol..

[8] Anderson R. H., Long G. C., Porter R. C., Anderson J. K. (2016). Occurrence of Select
Perfluoroalkyl Substances at U.S. Air Force Aqueous Film-Forming Foam
Release Sites Other than Fire-Training Areas: Field-Validation of
Critical Fate and Transport Properties. Chemosphere.

[9] PFAS National Primary Drinking Water Regulation; Correction, 89 F.R. 49101. (proposed June 11, 2024). https://www.federalregister.gov/documents/2024/06/11/2024-12645/pfas-national-primary-drinking-water-regulation-correction. (accessed February 05, 2026).

[10] D’Agostino L. A., Mabury S. A. (2014). Identification of Novel Fluorinated Surfactants in
Aqueous Film Forming Foams and Commercial Surfactant Concentrates. Environ. Sci. Technol..

[11] Weiner B., Yeung L. W. Y., Marchington E. B., D’Agostino L. A., Mabury S. A. (2013). Organic Fluorine Content in Aqueous
Film Forming Foams
(AFFFs) and Biodegradation of the Foam Component 6 : 2 Fluorotelomermercaptoalkylamido
Sulfonate (6 : 2 FTSAS). Environ. Chem..

[12] Yan P.-F., Dong S., Pennell K. D., Cápiro N. L. (2024). A Review
of the Occurrence and Microbial Transformation of Per- and Polyfluoroalkyl
Substances (PFAS) in Aqueous Film-Forming Foam (AFFF)-Impacted Environments. Sci. Total Environ..

[13] Houtz E. F., Higgins C. P., Field J. A., Sedlak D. L. (2013). Persistence of Perfluoroalkyl
Acid Precursors in AFFF-Impacted Groundwater and Soil. Environ. Sci. Technol..

[14] Gonda N., Choyke S., Schaefer C., Higgins C. P., Voelker B. (2023). Hydroxyl Radical
Transformations of Perfluoroalkyl Acid (PFAA) Precursors in Aqueous
Film Forming Foams (AFFFs). Environ. Sci. Technol..

[15] LaFond J. A., Rezes R., Shojaei M., Anderson T., Jackson W. A., Guelfo J. L., Hatzinger P. B. (2024). Biotransformation of PFAA Precursors
by Oxygenase-Expressing Bacteria in AFFF-Impacted Groundwater and
in Pure-Compound Studies with 6:2 FTS and EtFOSE. Environ. Sci. Technol..

[16] Schaefer C. E., Choyke S., Ferguson P. L., Andaya C., Burant A., Maizel A., Strathmann T. J., Higgins C. P. (2018). Electrochemical
Transformations of Perfluoroalkyl Acid (PFAA) Precursors and PFAAs
in Groundwater Impacted with Aqueous Film Forming Foams. Environ. Sci. Technol..

[17] Guelfo J. L., Korzeniowski S., Mills M. A., Anderson J., Anderson R. H., Arblaster J. A., Conder J. M., Cousins I. T., Dasu K., Henry B. J., Lee L. S., Liu J., McKenzie E. R., Willey J. (2021). Environmental
Sources, Chemistry, Fate, and Transport
of Per- and Polyfluoroalkyl Substances: State of the Science, Key
Knowledge Gaps, and Recommendations Presented at the August 2019 SETAC
Focus Topic Meeting. Environ. Toxicol. Chem..

[18] Higgins C.
P., Luthy R. G. (2006). Sorption
of Perfluorinated Surfactants on Sediments. Environ. Sci. Technol..

[19] Guelfo J. L., Higgins C. P. (2013). Subsurface Transport
Potential of Perfluoroalkyl Acids
at Aqueous Film-Forming Foam (AFFF)-Impacted Sites. Environ. Sci. Technol..

[20] Du Z., Deng S., Bei Y., Huang Q., Wang B., Huang J., Yu G. (2014). Adsorption
Behavior and Mechanism
of Perfluorinated Compounds on Various Adsorbents—A Review. J. Hazard. Mater..

[21] Adamson D. T., Kulkarni P. R., Nickerson A., Higgins C. P., Field J., Schwichtenberg T., Newell C., Kornuc J. J. (2022). Characterization
of Relevant Site-Specific PFAS Fate and Transport Processes at Multiple
AFFF Sites. Environ. Adv..

[22] Pritchard J. C., Hire M., McDonough J., Higgins C. P., Schaefer C. E. (2026). PFAS Conceptual
Site Model of an AFFF-Impacted Firefighting Training Area Informed
by High Resolution Soil, Porewater, and Groundwater Sampling. J. Contam. Hydrol..

[23] Dauchy X., Boiteux V., Colin A., Hémard J., Bach C., Rosin C., Munoz J.-F. (2019). Deep Seepage of
Per- and Polyfluoroalkyl Substances through the Soil of a Firefighter
Training Site and Subsequent Groundwater Contamination. Chemosphere.

[24] Adamson D. T., Nickerson A., Kulkarni P. R., Higgins C. P., Popovic J., Field J., Rodowa A., Newell C., DeBlanc P., Kornuc J. J. (2020). Mass-Based,
Field-Scale Demonstration of PFAS Retention
within AFFF-Associated Source Areas. Environ.
Sci. Technol..

[25] Guo B., Zeng J., Brusseau M. L. (2020). A Mathematical Model for the Release,
Transport, and Retention of Per- and Polyfluoroalkyl Substances (PFAS)
in the Vadose Zone. Water Resour. Res..

[26] Anderson R. H., Adamson D. T., Stroo H. F. (2019). Partitioning of Poly- and Perfluoroalkyl
Substances from Soil to Groundwater within Aqueous Film-Forming Foam
Source Zones. J. Contam. Hydrol..

[27] Filipovic M., Woldegiorgis A., Norström K., Bibi M., Lindberg M., Österås A.-H. (2015). Historical
Usage of Aqueous Film
Forming Foam: A Case Study of the Widespread Distribution of Perfluoroalkyl
Acids from a Military Airport to Groundwater, Lakes, Soils and Fish. Chemosphere.

[28] Xiao F., Simcik M. F., Halbach T. R., Gulliver J. S. (2015). Perfluorooctane
Sulfonate (PFOS) and Perfluorooctanoate (PFOA) in Soils and Groundwater
of a U.S. Metropolitan Area: Migration and Implications for Human
Exposure. Water Res..

[29] Weber A. K., Barber L. B., LeBlanc D. R., Sunderland E. M., Vecitis C. D. (2017). Geochemical and Hydrologic Factors
Controlling Subsurface
Transport of Poly- and Perfluoroalkyl Substances, Cape Cod, Massachusetts. Environ. Sci. Technol..

[30] Høisæter Å., Pfaff A., Breedveld G. D. (2019). Leaching
and Transport of PFAS from
Aqueous Film-Forming Foam (AFFF) in the Unsaturated Soil at a Firefighting
Training Facility under Cold Climatic Conditions. J. Contam. Hydrol..

[31] Nickerson A., Maizel A. C., Kulkarni P. R., Adamson D. T., Kornuc J. J., Higgins C. P. (2020). Enhanced Extraction of AFFF-Associated
PFASs from Source
Zone Soils. Environ. Sci. Technol..

[32] Gonda N., Zhang C., Tepedelen D., Smith A., Schaefer C., Higgins C. P. (2024). Quantitative Assessment of Poly- and Perfluoroalkyl
Substances (PFASs) in Aqueous Film Forming Foam (AFFF)–Impacted
Soils: A Comparison of Analytical Protocols. Anal. Bioanal. Chem..

[33] Dong S., Yan P.-F., Manz K. E., Abriola L. M., Pennell K. D., Cápiro N. L. (2024). Fate and
Transformation of 15 Classes of Per- and Polyfluoroalkyl
Substances in Aqueous Film-Forming Foam (AFFF)-Amended Soil Microcosms. Environ. Sci. Technol..

[34] Charbonnet J. A., McDonough C. A., Xiao F., Schwichtenberg T., Cao D., Kaserzon S., Thomas K. V., Dewapriya P., Place B. J., Schymanski E. L., Field J. A., Helbling D. E., Higgins C. P. (2022). Communicating Confidence
of Per- and Polyfluoroalkyl
Substance Identification via High-Resolution Mass Spectrometry. Environ. Sci. Technol. Lett..

[35] U.S. Environmental Protection Agency Soil Screening Guidance: User’s Guide 1996 https://semspub.epa.gov/work/HQ/175238.pdf. (accessed February 05, 2026).

[36] U.S. Environmental Protection Agency Supplemental guidance for developing soil screening levels for superfund sites 2002 https://semspub.epa.gov/work/HQ/175878.pdf. (accessed February 05, 2026).

[37] Interstate Technology & Regulatory Council Examination of Risk-Based Screening Values and Approaches of Selected States 2005 https://itrcweb.org/wp-content/uploads/2024/09/RISK-1.pdf. (accessed February 05, 2026).

[38] Bigler M. C., Brusseau M. L., Guo B., Jones S. L., Pritchard J. C., Higgins C. P., Hatton J. (2024). High-Resolution Depth-Discrete Analysis
of PFAS Distribution and Leaching for a Vadose-Zone Source at an AFFF-Impacted
Site. Environ. Sci. Technol..

[39] Place, B. Suspect List of Possible Per- and Polyfluoroalkyl Substances (PFAS), National Institute of Standards and Technology 2021 10.18434/mds2-2387.

[40] Hao S., Choi Y.-J., Wu B., Higgins C. P., Deeb R., Strathmann T. J. (2021). Hydrothermal Alkaline Treatment for Destruction of
Per- and Polyfluoroalkyl Substances in Aqueous Film-Forming Foam. Environ. Sci. Technol..

[41] Nguyen T. M. H., Bräunig J., Thompson K., Thompson J., Kabiri S., Navarro D. A., Kookana R. S., Grimison C., Barnes C. M., Higgins C. P., McLaughlin M. J., Mueller J. F. (2020). Influences of Chemical Properties,
Soil Properties,
and Solution pH on Soil–Water Partitioning Coefficients of
Per- and Polyfluoroalkyl Substances (PFASs). Environ. Sci. Technol..

[42] Backe W. J., Day T. C., Field J. A. (2013). Zwitterionic,
Cationic, and Anionic
Fluorinated Chemicals in Aqueous Film Forming Foam Formulations and
Groundwater from U.S. Military Bases by Nonaqueous Large-Volume Injection
HPLC-MS/MS. Environ. Sci. Technol..

[43] Schaefer C.
E., Nguyen D., Fang Y., Gonda N., Zhang C., Shea S., Higgins C. P. (2024). PFAS Porewater Concentrations in
Unsaturated Soil: Field and Laboratory Comparisons Inform on PFAS
Accumulation at Air-Water Interfaces. J. Contam.
Hydrol..

[44] Maizel A. C., Shea S., Nickerson A., Schaefer C., Higgins C. P. (2021). Release
of Per- and Polyfluoroalkyl Substances from Aqueous Film-Forming Foam
Impacted Soils. Environ. Sci. Technol..

[46] Fang B., Zhang Y., Chen H., Qiao B., Yu H., Zhao M., Gao M., Li X., Yao Y., Zhu L., Sun H. (2024). Stability and Biotransformation of 6:2 Fluorotelomer
Sulfonic Acid, Sulfonamide Amine Oxide, and Sulfonamide Alkylbetaine
in Aerobic Sludge. Environ. Sci. Technol..

[47] Wang N., Liu J., Buck R. C., Korzeniowski S. H., Wolstenholme B. W., Folsom P. W., Sulecki L. M. (2011). 6:2 Fluorotelomer Sulfonate Aerobic
Biotransformation in Activated Sludge of Waste Water Treatment Plants. Chemosphere.

[48] Moe M. K., Huber S., Svenson J., Hagenaars A., Pabon M., Trümper M., Berger U., Knapen D., Herzke D. (2012). The Structure of the
Fire Fighting Foam Surfactant
Forafac 1157 and Its Biological and Photolytic Transformation Products. Chemosphere.

[49] Barzen-Hanson K. A., Roberts S. C., Choyke S., Oetjen K., McAlees A., Riddell N., McCrindle R., Ferguson P. L., Higgins C. P., Field J. A. (2017). Discovery of 40
Classes of Per- and Polyfluoroalkyl
Substances in Historical Aqueous Film-Forming Foams (AFFFs) and AFFF-Impacted
Groundwater. Environ. Sci. Technol..

[50] Shaw D. M. J., Munoz G., Bottos E. M., Duy S. V., Sauvé S., Liu J., Van Hamme J. D. (2019). Degradation and Defluorination of 6:2 Fluorotelomer
Sulfonamidoalkyl Betaine and 6:2 Fluorotelomer Sulfonate by *Gordonia* Sp. Strain NB4–1Y under Sulfur-Limiting
Conditions. Sci. Total Environ..

[51] Liu M., Munoz G., Vo Duy S., Sauvé S., Liu J. (2021). Stability of Nitrogen-Containing
Polyfluoroalkyl Substances in Aerobic
Soils. Environ. Sci. Technol..

[52] Ruyle B. J., Thackray C. P., Butt C. M., LeBlanc D. R., Tokranov A. K., Vecitis C. D., Sunderland E. M. (2023). Centurial Persistence of Forever
Chemicals at Military Fire Training Sites. Environ.
Sci. Technol..

