# Supplementary material for: Evaluating the Relative Importance of Polyfluoroalkyl Substances in AFFF-Impacted Soils
Source: Environ Sci Technol. 2026 Apr 27;60(18):13610–21. doi: 10.1021/acs.est.5c17749 (PMC13173642; doi:10.1021/acs.est.5c17749)
Supplement: Supplementary file 1 [file es5c17749_si_001.pdf]

Supporting Information

Evaluating the Relative Importance of Polyfluoroalkyl Substances in AFFF-impacted Soils

*Sara L. Jones[1], Nicholas Gonda[1], J. Conrad Pritchard[1], Jaydon Richardson[1], Matthew C. Bigler[2], Mark L. Brusseau[2,3], Bo Guo[3], James Hatton[4], Maxwell Hire[5], Charles E. Schaefer[5], Christopher P. Higgins[1,]**

[1]Department of Civil and Environmental Engineering, Colorado School of Mines, 1500 Illinois Street, Golden, Colorado 80401, United States

[2]Department of Environmental Science, The University of Arizona, 1177 East 4th Street, Tucson, Arizona 85721, United States

[3]Department of Hydrology and Atmospheric Sciences, The University of Arizona, 1133 James E. Rogers Way, Tucson, Arizona 85721, United States

[4]Jacobs Engineering Group Inc Englewood, Englewood, CO, 80112, USA

[5]CDM Smith, 110 Fieldcrest Avenue, #8, Sixth Floor, Edison, New Jersey, 08837, United States

*Corresponding Author: chiggins@mines.edu

Pages: 209

Figures: 192

Schemes: 2

Tables: 3

Contents

Text S1. Descriptions of ten U.S. DoD sites analyzed in the study ... 9
Text S2. Additional details on soil sample preparation for PFAS Analysis ... 13
Text S3. Details on LC-QToF-MS Analysis ... 13
Text S4. Data acquisition parameters, qualitative, and quantitative analysis ... 14
Text S4.1. Target analysis ... 14
Text S4.2. Suspect screening analysis ... 15
Text S4.3. Semiquantitative analysis ... 15

Table S1. Additional, tabulated information on cores used in this study ... 17
Table S2. List of Targeted PFAS analytes and internal standards ... 18
Table S3. List of Suspect Hit PFASs and their target calibrants ... 21

Figure S1. Detection frequency heatmap of all PFAAs across cores C02 through C10 ... 26
Figure S2. Detection frequency heatmap of all PFAA precursors across cores C02 through C10 ... 27
Figure S3. Vertical distribution profile of SPr-FPrSA across the twelve studied cores ... 29
Figure S4. Vertical distribution profile of SPr-FBSA across the twelve studied cores ... 30
Figure S5. Vertical distribution profile of SPr-FPeSA across the twelve studied cores ... 31
Figure S6. Vertical distribution profile of SPr-FHxSA across the twelve studied cores ... 32
Figure S7. Vertical distribution profile of CMeAmPr-FBSA across the twelve studied cores ... 33
Figure S8. Vertical distribution profile of CMeAmPr-FHxSA across the twelve studied cores ... 34
Figure S9. Vertical distribution profile of SPrAmPr-FPrSA across the twelve studied cores ... 35
Figure S10. Vertical distribution profile of SPrAmPr-FBSA across the twelve studied cores ... 36
Figure S11. Vertical distribution profile of SPrAmPr-FPeSA across the twelve studied cores ... 37
Figure S12. Vertical distribution profile of SPrAmPr-FHxSA across the twelve studied cores ... 38
Figure S13. Vertical distribution profile of F5S-PFOS across the twelve studied cores ... 39
Figure S14. Vertical distribution profile of H-UPFOS across the twelve studied cores ... 40
Figure S15. Vertical distribution profile of H-PFPeS across the twelve studied cores ... 41
Figure S16. Vertical distribution profile of H-PFHxS across the twelve studied cores ... 42
Figure S17. Vertical distribution profile of H-PFOS across the twelve studied cores ... 43
Figure S18. Vertical distribution profile of S-OHPrAmPr-FBSA-OHPrS across the twelve studied cores ... 44
Figure S19. Vertical distribution profile of S-OHPrAmPr-FHxSA-OHPrS across the twelve studied cores ... 45

Figure S20. Vertical distribution profile of S-OHPrAmPr-FBSA across the twelve studied cores 46
Figure S21. Vertical distribution profile of S-OHPrAmPr-FPeSA across the twelve studied cores 47
Figure S22. Vertical distribution profile of S-OHPrAmPr-FHxSA across the twelve studied cores 48
Figure S23. Vertical distribution profile of SPrAmPr-FPrSAA across the twelve studied cores 49
Figure S24. Vertical distribution profile of SPrAmPr-FBSAA across the twelve studied cores 50
Figure S25. Vertical distribution profile of 8:2 FTSO2PrA across the twelve studied cores 51
Figure S26. Vertical distribution profile of SPrAmPr-FPrSAPrS across the twelve studied cores 52
Figure S27. Vertical distribution profile of SPrAmPr-FBSAPrS across the twelve studied cores 53
Figure S28. Vertical distribution profile of SPrAmPr-FPeSAPrS across the twelve studied cores 54
Figure S29. Vertical distribution profile of SPrAmPr-FHxSAPrS across the twelve studied cores 55
Figure S30. Vertical distribution profile of diOHPrAm-MeOHPr-FBSA across the twelve studied cores 56
Figure S31. Vertical distribution profile of diOHPrAm-MeOHPr-FPeSA across the twelve studied cores 57
Figure S32. Vertical distribution profile of diOHPrAm-MeOHPr-FHxSA across the twelve studied cores 58
Figure S33. Vertical distribution profile of diOHPrAm-MeOHPr-FBSAPrS across the twelve studied cores 59
Figure S34. Vertical distribution profile of diOHPrAm-MeOHPr-FPeSAPrS across the twelve studied cores 60
Figure S35. Vertical distribution profile of diOHPrAm-MeOHPr-FHxSAPrS across the twelve studied cores 61
Figure S36. Vertical distribution profile of Cl-PFBS across the twelve studied cores 62
Figure S37. Vertical distribution profile of Cl-PFHxS across the twelve studied cores 63
Figure S38. Vertical distribution profile of Cl-PFOS across the twelve studied cores 64
Figure S39. Vertical distribution profile of PFBSi across the twelve studied cores 65
Figure S40. Vertical distribution profile of PFPeSi across the twelve studied cores 66
Figure S41. Vertical distribution profile of PFHxSi across the twelve studied cores 67
Figure S42. Vertical distribution profile of PFOSi across the twelve studied cores 68
Figure S43. Vertical distribution profile of 4:2 FTSO2PrAd-DiMeEtS across the twelve studied cores ... 69
Figure S44. Vertical distribution profile of 6:2 FTSO2PrAd-DiMeEtS across the twelve studied cores ... 70
Figure S45. Vertical distribution profile of 8:2 FTSO2PrAd-DiMeEtS across the twelve studied cores ... 71
Figure S46. Vertical distribution profile of 4:2 FTS across the twelve studied cores 72
Figure S47. Vertical distribution profile of 6:2 FTS across the twelve studied cores 73
Figure S48. Vertical distribution profile of 8:2 FTS across the twelve studied cores 74
Figure S49. Vertical distribution profile of 10:2 FTS across the twelve studied cores 75
Figure S50. Vertical distribution profile of 6:2 FTTh-PrAd-DiMeEtS across the twelve studied cores 76

Figure S51. Vertical distribution profile of 8:2 FTTh-PrAd-DiMeEtS across the twelve studied cores 77
Figure S52. Vertical distribution profile of K-PFPeS across the twelve studied cores 78
Figure S53. Vertical distribution profile of K-PFHxS across the twelve studied cores 79
Figure S54. Vertical distribution profile of K-PFHpS across the twelve studied cores 80
Figure S55. Vertical distribution profile of K-PFOS across the twelve studied cores 81
Figure S56. Vertical distribution profile of FPrSA across the twelve studied cores 82
Figure S57. Vertical distribution profile of FBSA across the twelve studied cores 83
Figure S58. Vertical distribution profile of FPeSA across the twelve studied cores 84
Figure S59. Vertical distribution profile of FHxSA across the twelve studied cores 85
Figure S60. Vertical distribution profile of FHpSA across the twelve studied cores 86
Figure S61. Vertical distribution profile of FOSA across the twelve studied cores 87
Figure S62. Vertical distribution profile of EtFBSAA across the twelve studied cores 88
Figure S63. Vertical distribution profile of EtFPeSAA across the twelve studied cores 89
Figure S64. Vertical distribution profile of EtFHxSAA across the twelve studied cores 90
Figure S65. Vertical distribution profile of FBSAA across the twelve studied cores 91
Figure S66. Vertical distribution profile of FPeSAA across the twelve studied cores 92
Figure S67. Vertical distribution profile of FHxSAA across the twelve studied cores 93
Figure S68. Vertical distribution profile of FOSAA across the twelve studied cores 94
Figure S69. Vertical distribution profile of AmPr-FEtSA-PrA across the twelve studied cores 95
Figure S70. Vertical distribution profile of AmPr-FPrSA-PrA across the twelve studied cores 96
Figure S71. Vertical distribution profile of AmPr-FBSA-PrA across the twelve studied cores 97
Figure S72. Vertical distribution profile of AmPr-FHxSA-PrA across the twelve studied cores 98
Figure S73. Vertical distribution profile of AmPr-FPrSA across the twelve studied cores 99
Figure S74. Vertical distribution profile of AmPr-FBSA across the twelve studied cores 100
Figure S75. Vertical distribution profile of AmPr-FPeSA across the twelve studied cores 101
Figure S76. Vertical distribution profile of AmPr-FHxSA across the twelve studied cores 102
Figure S77. Vertical distribution profile of AmPr-FHpSA across the twelve studied cores 103
Figure S78. Vertical distribution profile of AmPr-FOSA across the twelve studied cores 104
Figure S79. Vertical distribution profile of UPFHxS across the twelve studied cores 105
Figure S80. Vertical distribution profile of UPFHpS across the twelve studied cores 106
Figure S81. Vertical distribution profile of UPFOS across the twelve studied cores 107
Figure S82. Vertical distribution profile of UPFNS across the twelve studied cores 108
Figure S83. Vertical distribution profile of UPFDS across the twelve studied cores 109
Figure S84. Vertical distribution profile of PFPe-OS across the twelve studied cores 110
Figure S85. Vertical distribution profile of PFHx-OS across the twelve studied cores 111

Figure S86. Vertical distribution profile of PFHp-OS across the twelve studied cores............................112
Figure S87. Vertical distribution profile of PFO-OS across the twelve studied cores.............................113
Figure S88. Vertical distribution profile of PFN-OS across the twelve studied cores.............................114
Figure S89. Vertical distribution profile of 1OH-4:2 FTS across the twelve studied cores 115
Figure S90. Vertical distribution profile of 1OH-6:2 FTS across the twelve studied cores 116
Figure S91. Vertical distribution profile of 6:2 FTSi across the twelve studied cores 117
Figure S92. Vertical distribution profile of 8:2 FTSi across the twelve studied cores 118
Figure S93. Vertical distribution profile of EtFHxSA across the twelve studied cores............................119
Figure S94. Vertical distribution profile of EtFDSA across the twelve studied cores.............................120
Figure S95. Vertical distribution profile of EtFDoSA across the twelve studied cores............................121
Figure S96. Vertical distribution profile of MeFBSA across the twelve studied cores 122
Figure S97. Vertical distribution profile of MeFPeSA across the twelve studied cores...........................123
Figure S98. Vertical distribution profile of MeFHxSA across the twelve studied cores..........................124
Figure S99. Vertical distribution profile of MeFOSA across the twelve studied cores 125
Figure S100. Vertical distribution profile of CMeAmPr-FPrSAPrA across the twelve studied cores 126
Figure S101. Vertical distribution profile of CMeAmPr-FBSAPrA across the twelve studied cores 127
Figure S102. Vertical distribution profile of CMeAmPr-FHxSAPrA across the twelve studied cores....128
Figure S103. Vertical distribution profile of CMeAmPr-FBSAA across the twelve studied cores..........129
Figure S104. Vertical distribution profile of CMeAmPr-FHxSAA across the twelve studied cores 130
Figure S105. Vertical distribution profile of CEtAmPr-N-EtFHxSA across the twelve studied cores 131
Figure S106. Vertical distribution profile of diOHBAmPr-FBSA across the twelve studied cores.........132
Figure S107. Vertical distribution profile of diOHBAmPr-FPeSA across the twelve studied cores........133
Figure S108. Vertical distribution profile of diOHBAmPr-FHxSA across the twelve studied cores.......134
Figure S109. Vertical distribution profile of diOHBAmPr-FHpSA across the twelve studied cores.......135
Figure S110. Vertical distribution profile of diOHBAmPr-FOSA across the twelve studied cores.........136
Figure S111. Vertical distribution profile of 6:2 UFTS across the twelve studied cores 137
Figure S112. Vertical distribution profile of 6:2 FTSO-PrAd-DiMeEtS across the twelve studied cores138
Figure S113. Vertical distribution profile of 8:2 FTSO-PrAd-DiMeEtS across the twelve studied cores139
Figure S114. Vertical distribution profile of 6:2 FTSAPr-OHMeMeAn across the twelve studied cores140
Figure S115. Vertical distribution profile of EtOH-Am-OHPr-FHxSA across the twelve studied cores. 141
Figure S116. Vertical distribution profile of EtOH-AmPr-FBSA across the twelve studied cores..........142
Figure S117. Vertical distribution profile of EtOH-AmPr-FPeSA across the twelve studied cores.........143
Figure S118. Vertical distribution profile of EtOH-AmPr-FHxSA across the twelve studied cores........144
Figure S119. Vertical distribution profile of EtOH-AmPr-FHpSA across the twelve studied cores........145
Figure S120. Vertical distribution profile of EtOH-AmPr-FOSA across the twelve studied cores..........146

Figure S121. Vertical distribution profile of EtOH-AmPr-FBSA-OHPrS across the twelve studied cores 147
Figure S122. Vertical distribution profile of EtOH-AmPr-FPeSA-OHPrS across the twelve studied cores 148
Figure S123. Vertical distribution profile of EtOH-AmPr-FHxSA-OHPrS across the twelve studied cores 149
Figure S124. Vertical distribution profile of SPrAmPr-N-Me-FHxSA across the twelve studied cores .. 150
Figure S125. Vertical distribution profile of EtOH-AmPr-FHxSAPrS across the twelve studied cores .. 151
Figure S126. Vertical distribution profile of 6:2 FTSA-PrB (6:2 FTAB) across the twelve studied cores 152
Figure S127. Vertical distribution profile of 8:2 FTSA-PrB (8:2 FTAB) across the twelve studied cores 153
Figure S128. Vertical distribution profile of TAmPr-N-MeFBSA across the twelve studied cores 154
Figure S129. Vertical distribution profile of TAmPr-N-MeFPeSA across the twelve studied cores 155
Figure S130. Vertical distribution profile of TAmPr-N-MeFHxSA across the twelve studied cores 156
Figure S131. Vertical distribution profile of TAmPr-N-MeFOSA across the twelve studied cores 157
Figure S132. Vertical distribution profile of TAmPr-FBSA across the twelve studied cores 158
Figure S133. Vertical distribution profile of TAmPr-FPeSA across the twelve studied cores 159
Figure S134. Vertical distribution profile of TAmPr-FHxSA across the twelve studied cores 160
Figure S135. Vertical distribution profile of TAmPr-FHpSA across the twelve studied cores 161
Figure S136. Vertical distribution profile of TAmPr-FOSA across the twelve studied cores 162
Figure S137. Vertical distribution profile of EtAmPr-FEt-N-EtSA across the twelve studied cores 163
Figure S138. Vertical distribution profile of EtAmPr-FPr-N-EtSA across the twelve studied cores 164
Figure S139. Vertical distribution profile of EtAmPr-FB-N-EtSA across the twelve studied cores 165
Figure S140. Vertical distribution profile of EtAmPr-FPe-N-EtSA across the twelve studied cores....... 166
Figure S141. Vertical distribution profile of EtAmPr-FHx-N-EtSA across the twelve studied cores...... 167
Figure S142. Vertical distribution profile of EtAmPr-FHp-N-EtSA across the twelve studied cores...... 168
Figure S143. Vertical distribution profile of EtAmPr-FO-N-EtSA across the twelve studied cores........ 169
Figure S144. Vertical distribution profile of 7:3 FTCA across the twelve studied cores 170
Figure S145. Vertical distribution profile of PFBA across the twelve studied cores 171
Figure S146. Vertical distribution profile of PFPeA across the twelve studied cores 172
Figure S147. Vertical distribution profile of PFHxA across the twelve studied cores 173
Figure S148. Vertical distribution profile of PFHpA across the twelve studied cores 174
Figure S149. Vertical distribution profile of PFOA across the twelve studied cores 175
Figure S150. Vertical distribution profile of PFNA across the twelve studied cores 176
Figure S151. Vertical distribution profile of PFDA across the twelve studied cores 177

Figure S152. Vertical distribution profile of PFUdA across the twelve studied cores 178
Figure S153. Vertical distribution profile of PFDoA across the twelve studied cores 179
Figure S154. Vertical distribution profile of 6:2 UFTCA across the twelve studied cores 180
Figure S155. Vertical distribution profile of 8:2 UFTCA across the twelve studied cores 181
Figure S156. Vertical distribution profile of PFPrS across the twelve studied cores 182
Figure S157. Vertical distribution profile of PFBS across the twelve studied cores 183
Figure S158. Vertical distribution profile of PFPeS across the twelve studied cores 184
Figure S159. Vertical distribution profile of PFHxS across the twelve studied cores 185
Figure S160. Vertical distribution profile of PFHpS across the twelve studied cores 186
Figure S161. Vertical distribution profile of PFOS across the twelve studied cores 187
Figure S162. Vertical distribution profile of PFNS across the twelve studied cores 188
Figure S163. Vertical distribution profile of PFDS across the twelve studied cores 189
Figure S163. Vertical distribution profile of PFDoS across the twelve studied cores 190
Figure S164. Spearman's rank correlation matrices and corresponding p-values of PFASs within the SPr-FASA class 191
Figure S165. Spearman's rank correlation matrices and corresponding p-values of PFASs within the SPrAmPr-FASA class 191
Figure S166. Spearman's rank correlation matrices and corresponding p-values of PFASs within the H-PFSA class 192
Figure S167. Spearman's rank correlation matrices and corresponding p-values of PFASs within the S-OHPrAmPr-FASA class 192
Figure S168. Spearman's rank correlation matrices and corresponding p-values of PFASs within the diOHPrAm-MeOHPr-FASA class 193
Figure S169. Spearman's rank correlation matrices and corresponding p-values of PFASs within the diOHPrAm-MeOHPr-FASAPrS class 193
Figure S170. Spearman's rank correlation matrices and corresponding p-values of PFASs within the Cl-PFSA class 194
Figure S171. Spearman's rank correlation matrices and corresponding p-values of PFASs within the PFSAi class 194
Figure S172. Spearman's rank correlation matrices and corresponding p-values of PFASs within the X:2 FTSO2PrAd-DiMeEtS class 195
Figure S173. Spearman's rank correlation matrices and corresponding p-values of PFASs within the X:2 FTS class 195
Figure S174. Spearman's rank correlation matrices and corresponding p-values of PFASs within the K-PFSA class 196
Figure S175. Spearman's rank correlation matrices and corresponding p-values of PFASs within the FASA class 196

Figure S176. Spearman's rank correlation matrices and corresponding p-values of PFASs within the FASAA class 197

Figure S177. Spearman's rank correlation matrices and corresponding p-values of PFASs within the AmPr-FASA-PrA class 197

Figure S178. Spearman's rank correlation matrices and corresponding p-values of PFASs within the AmPr-FASA class 198

Figure S179. Spearman's rank correlation matrices and corresponding p-values of PFASs within the UPFSA class 198

Figure S180. Spearman's rank correlation matrices and corresponding p-values of PFASs within the PFA-OS class 199

Figure S181. Spearman's rank correlation matrices and corresponding p-values of PFASs within the EtFASA class 199

Figure S182. Spearman's rank correlation matrices and corresponding p-values of PFASs within the MeFASA class 200

Figure S183. Spearman's rank correlation matrices and corresponding p-values of PFASs within the CMeAmPr-FASAPrA class 200

Figure S184. Spearman's rank correlation matrices and corresponding p-values of PFASs within the diOHBAmPr-FASA class 201

Figure S185. Spearman's rank correlation matrices and corresponding p-values of PFASs within the EtOH-AmPr-FASA class 201

Figure S186. Spearman's rank correlation matrices and corresponding p-values of PFASs within the TAmPr-N-MeFASA class 202

Figure S187. Spearman's rank correlation matrices and corresponding p-values of PFASs within the TAmPr-FASA class 202

Figure S188. Spearman's rank correlation matrices and corresponding p-values of PFASs within the EtAmPr-FA-N-EtSA class 203

Figure S189. Spearman's rank correlation matrices and corresponding p-values of PFASs within the PFCA class 203

Figure S190. Spearman's rank correlation matrices and corresponding p-values of PFASs within the PFSA class 204

Figure S191. Spearman's rank correlation matrices and corresponding p-values of PFOA and its FT precursors 205

Figure S192. Side-by-side comparison of PFAS contributions per core when using only EPA Method 1633 analyte list vs extended 1633 + NTA analyte list 206

Scheme S1. Transformation scheme of the PFHxA precursors features in Figure 4 based on those proposed in literature 207

Scheme S2. Transformation scheme of the PFHxS precursors featured in Figure 5 based on those proposed in literature 208

References......209

Text S1. Descriptions of ten U.S. DoD sites analyzed in the study

Site S01: Located in the mountainous, southwestern U.S., the source zone at site S01 (where *core C01* was collected) is a historic firefighting training area that consisted of unlined earthen berms and a lined burn pit. AFFF was applied weekly from 1968 to 1972 and monthly from 1972 to 1989 for training purposes, though the amount used during these trainings is unknown. The climate for this region is defined as hot, semi-arid, and it sees on average 27 cm of precipitation annually. The soil at the site is classified as the Mohave series (Fine-loamy, mixed, superactive, thermic Typic Calciargids). This series has a predominantly sand clay loam texture with a B horizon averaging 27 – 40% clay and a C horizon averaging 3 – 30% clay. The upper 30 cm of soil has low organic matter content (<1%) and a calcic horizon ranges from 100 – 140 cm bgs. The average groundwater recharge rate is 3 cm per year.

Site S02: Located in the mountainous, western U.S., the source zone at site S02 (where *core C02* was collected) is a current firefighting training area that includes a burn pit, a dual high-density polyethylene liner, and a mock aircraft. The training area has been in operation since 1989 and AFFFs were used frequently since this time, but the exact frequency and amount is unknown. The climate for this region is defined as cold, semi-arid and it sees on average 54 cm of precipitation annually. The soil at the site is classified as the Truckton series (Coarse-loamy, mixed, superactive, mesic Aridic Argiustolls). This series has a predominantly coarse sandy loam texture from the surface down to about 1.5 m bgs averaging 50 – 80% sand. The average groundwater recharge rate is 4 cm per year.

Site S03: Located in west south central U.S., the source zone at site S03 (where *core C03* was collected) is a historic firefighting training area that consisted of an unlined earthen berm and a concrete-lined burn pit. AFFF for training was applied monthly from the early 1970s through 1984 at the unlined berm. Monthly use of AFFF for training continued at the concrete-lined pit from 1984 to an unknown end date – concurrently, firefighting vehicles tested their systems by releasing AFFF into the unlined berm at an unknown frequency. The amount of AFFF used during these trainings/system tests is unknown. The climate for this region is defined as humid subtropical, and it sees on average 28 cm of precipitation annually. The soil at the site is classified as the Lewisville series (Fine-silty, mixed, active, thermic Udic Calciustolls). This series has a predominantly silty clay texture averaging 24 – 35% clay. A calcic horizon ranges from 41 to 157 cm bgs which overlaps a region containing up to 25% secondary carbonates starting at the surface. The average groundwater recharge rate is 4 cm per year.

Site S04: Located in the Pacific southwestern U.S., two source zones were studied at site S04. The source zone where *core C04A* was collected is a current firefighting training area that includes a gravel lot with a crash truck and a cement pad outside of a fire station. Nozzle spray tests, training exercises, and responses occur on the gravel lot, after which the fire engines are rinsed on the concrete pad. The time of first AFFF use, frequency of exercises, and amount of AFFF used during these exercises is unknown. The source zone where *core C04B* was collected is a current/historic firefighting training area that consists of unlined earthen berms (historic) and a lined fire training facility (current). The historic training areas were constructed around 1960, and the current training area was constructed in 1988 following the destruction of the historic FTAs earlier the same year. Prior to 1992, AFFFs or water was used for fire extinguishing training at an unknown frequency. After 1992, approximately 50 gallons of AFFF was used monthly during firefighting training. It is known that AFFF oftentimes sprayed outside the berm area due to high winds, and following training the nozzles were washed out at the training area. The climate for this region is defined as cold desert, and it sees on average 1 cm of precipitation annually. The soil at the site is classified as the Cajon/Helendale Series (Mixed, thermic Typic Torripsamments/ Coarse-loamy, mixed, superactive, thermic Typic Haplargids). These series have a predominantly sandy texture with sandy loam also present in many horizons. These series are known for their high saturated hydraulic conductivity. The average groundwater recharge rate is 1 cm per year.

Site S05: Located in the south Atlantic U.S., the source zone at site S05 (where *core C05* was collected) is a historic firefighting training area that includes a shallow, unlined burning pit. AFFF was applied at an unknown frequency from 1966 to 1991 for training purposes. The climate for this region is defined as humid subtropical, and it sees on average 68 cm of precipitation annually. The soil at the site is classified as the Sapelo series (Sandy, siliceous, thermic Ultic Alaquods). The predominant soil texture found in this series of soil is fine sand, though clay lenses are present in deeper horizons. This series is remarked to be a poorly draining soil series, though surface runoff is negligible. The average groundwater recharge rate is 21 cm per year.

Site S06: Located in the south mid-Atlantic U.S., the source zone at site S06 (where *core C06* was collected) is a historic firefighting training area. From the 1960s to mid-1970s, fire training took place twice per week on an abandoned runway where ignited waste fuel was extinguished using AFFF. The annual usage of AFFF in this timeframe was estimated to be 2,000 gallons. In the mid-1970s, a fire training ring with an earthen berm was installed to help contain runoff, though it was observed to still flow into surrounding soils. In the early 1980s, a second fire training ring was installed on a concrete pad with a concrete berm, though the twice-a-week training ceased sometime later in the decade. In the 1990s to 2001, fire trucks were tested quarterly near this area by spraying AFFF onto the grass. The climate for this site is defined as humid subtropical, and it sees on average 191 cm of precipitation annually. The soil at the site is classified as the Acredale series (Fine-silty, mixed, active, thermic Typic Endoaqualfs). The predominant soil texture found in this series is silt loam – iron accumulations are found starting at 18 cm bgs and deeper whereas

pockets of iron depletions are found starting at 89 cm bgs and deeper. The average groundwater recharge rate is 36 cm per year.

Site S07: Located in the south mid-Atlantic U.S., the source zone at site S07 (where *core C07* was collected) is a historic firefighting training area consisting of an earthen berm where ignited fuel was extinguished using AFFF, though the time of first application and frequency of trainings are unknown. The climate for this site is defined as humid subtropical, and it sees on average 106 cm of precipitation annually. The soil at the site is classified as the Acredale series, of which a further description may be found in the description of site S06 above. The average groundwater recharge rate is 26 cm per year.

Site S08: Located in the Pacific northwestern U.S., the source zone at site S08 (where *core C08* was collected) is a current and former fire station where AFFF is stored and used. The former fire station was in operation from the mid-1940s to 1995, when it was demolished and the current fire station was built in its place. Currently, approximately 2,000 gallons of AFFF (Chemguard and potentially other brands) are stored in trucks, 250-gallon poly storage tanks, and 5-gallon totes. Since 2015, monthly foam testing is conducted in a grassy area north of the fire station, though the quantities of foam used are reported to be small. The climate for this site is defined as Mediterranean-influenced warm-summer humid continental, and it sees on average 5 cm of precipitation per year. The soil at the site is classified as the Henley series (Coarse-loamy, mixed, superactive, mesic Aquic Haplodurids). The predominant soil texture is loam with roughly 10 to 18% clay across its horizons. This soil series sees a seasonally high water table, where soils around 60 cm bgs may be found fully saturated between the months of March and August. The average groundwater recharge rate is 3 cm per year.

Site S09: Located in east north central U.S., two source zones were studied at site S09. The source zone where *core C09A* was collected is an area near a building that formerly housed a jet engine test cell. This building was equipped with a fire suppression system which had an aboveground AFFF storage tank, though the volume and usage of the tank are unknown. The source zone where *core C09B* was collected is an area near a building that was formerly used as a maintenance hangar. This building was also equipped with a fire suppression system which has a 5,000-gallon, aboveground AFFF storage tank. The usage of this tank is unknown, but there is evidence that it was leaking. The climate for this site is defined as warm-summer humid continental, and it sees on average 81 cm of precipitation per year. The soil at the site is predominantly of the Grayling series (Isotic, frigid Typic Udipsamments), though urban soils have been mixed at the site. The predominant soil texture is sand –deeper horizons have coarser, almost gravelly sands. The average groundwater recharge rate is 25 cm per year.

Site S10: Located in the south Atlantic U.S., the source zone at site S10 (where *core C10* was collected) is a former fire training area consisting of an earthen berm. Fire trainings occurred from 1971 to 1983, though the frequency of the trainings and volumes of AFFF used are unknown. The

climate of this site is defined as humid subtropical, and it sees on average 75 cm of precipitation annually. The soil at the site is urbanized, though surveys show it contains fractions of soils from the Yauhannah, Yemassee, and Ogeechee series. The predominant soil texture is loamy fine sand. The average groundwater recharge rate is 23 cm per year.

Text S2. Additional details on soil sample preparation for PFAS Analysis

As described in the main text, soil samples were extracted using two main methods described in Nickerson et al.[1] and Gonda et al.[2], which are referred to as "Nickerson method" and "Bambino method" hereon, respectively. Samples from core C01 were extracted using the Nickerson method whereas all other cores (cores C02 through C10) were extracted using the Bambino method.

For LC-QToF-MS analysis, samples prepared using the Nickerson method were diluted to ensure that concentrations fell between a PFAS calibration range of 0.2- 20000 ng/L for both ESI- and ESI+ analytes. A 400 µL solution containing 20% water, 25% soil extract in methanol, 49.3% methanol, and 0.6% ammonium hydroxide was prepared. A final concentration of 750 ng/L of injection standard was also achieved in this solution (M2PFOA, Wellington Laboratories).

Similar to above, samples prepared using the Bambino method were diluted to ensure that concentrations fell between a PFAS calibration range of 5.05 – 12,500 ng/L for ESI- analytes and 10.13 – 25,070 ng/L for ESI+ analytes. A 750 µL solution containing 50% soil extract in methanol, 50% 20 mM ammonium acetate in water was prepared. A final concentration of 664.45 ng/L of injection standard was also achieved in this solution (M2PFOA, Wellington Laboratories).

Text S3. Details on LC-QToF-MS Analysis

100 µL of each sample was injected into a SCIEX Exion LC high-pressure liquid chromatography (HPLC) system with a Gemini C18 analytical column (3 mm × 100 mm, 5 µm; Phenomenex, Torrance, CA) with one SecurityGuardTM C18 Guard Cartridge (4 mm × 2 mm I.D.; Phenomenex) and two Zorbax DIOL guard cartridges (4.6 mm × 12.5 mm, 6 µm; Agilent, Santa Clara, CA) for ESI- mode or no Zorbax DIOLs for ESI+ mode. The column oven temperature was set to 40 °C. The aqueous mobile phase included two eluents: (A) 20 mM ammonium acetate (Fisher Scientific) in Optima™ LC/MS Grade water, and (B) 100% Optima™ LC/MS Grade methanol. Eluent flow rate was 0.60 mL/min, and the gradient can be found in the table below:

Time (min)	%A	%B	Curve
0.00	90	10	0
0.50	50	50	0
8.00	1	99	0
13.00	1	99	0
13.50	90	10	0
20.00	90	10	0

A SCIEX X500R QToF-MS system (Framingham, MA) was used to measure PFASs following electrospray ionization in both ESI+ and ESI- modes with SWATH® Data-Independent Acquisition. Precursor ion data for m/z 100-1200 was collected for 1283 cycles with a total scan time of 842 ms and accumulation time = 20 ms. The ion spray voltage was set at –4500 V (ESI-) or +4500 V (ESI+) and temperature was set to 550 °C with an ion source gas pressure of 60 psi, a curtain gas pressure of 35 psi, and a collision (CAD) gas pressure of 10 psi. For QToF scanning,

the collision energy was set to -5 V and the declustering potential was set to -20 V, both with a spread of 0 V. Product ion (MS/MS) scanning for m/z 50-1200 Da was conducted in the following mass m/z windows (Da): 100 − 150, 149 − 200, 199 − 250, 249 − 300, 349 − 400, 399 − 450, 449 − 550, 549 −650, 649 − 800, 799 − 1200. For each SWATH window, the accumulation was 50 ms and collision energy was –35 V with spread of 30 V.

Text S4. Data acquisition parameters, qualitative, and quantitative analysis

Text S4.1. Target analysis

Quantitative data acquisition and processing were performed using SCIEX OS Version 3.4.5 for targeted analytes. The compounds (signal-to-noise ratio >10) were confirmed based on chromatographic retention time and accurate mass (XIC window 0.01 Da) against analytical standards (<10 ppm difference). Initial integration parameters included: 90% of lowest-intensity peaks as noise, 2 min of a baseline-subtract window, minimum peak intensity =100, and peak width = 10 points.

Soil concentrations of targeted PFAS analytes were calculated using three different calibration approaches depending on the ionization mode. This is due to the availability of native and isotopically-labeled standards for analyte PFASs, though the calculations are consistent with previously published studies.[1–3] Each analyte was matched with an isotopically-labeled extracted internal standard (EIS) or non-extracted internal standard (NIS) for ESI- and ESI+ modes, respectively. The list of these target analyte-internal standard pairs can be found in Table S2. For ESI- target analytes, natives and exact or closely matching isotopically-labeled standards were commercially available during the time of this study, so isotopic dilution and internal standard calibration methods were utilized for these analytes. In-vial concentrations were calculated as a function of the response factor of the target analyte-internal standard pair, the observed peak areas of the analyte and the EIS, and the in-vial concentration of the spiked EIS (Equation S1).

$$\frac{C_{s,Tnative}}{C_{s,EIS}} = \frac{A_{s,Tnative}}{A_{s,EIS}} / \frac{A_{cal,Tnative} * C_{cal,EIS}}{C_{cal,Tnative} * A_{cal,EIS}} \qquad \text{Equation S1.}$$

Where,

$\frac{A_{cal,Tnative} * C_{cal,EIS}}{C_{cal,Tnative} * A_{cal,EIS}}$ = a term that represents the response factor (RF) of the analyte-internal standard pair, derived from the slope of the calibration curve ($1/x^2$ weight) of the native.

$A_{s,Tnative}$ = the observed peak area of the analyte

$A_{s,EIS}$ = the observed peak area of the internal standard

$C_{s,EIS}$ = the spiked concentration of internal standard

$C_{s,Tnative}$ = the calculated concentration of analyte in-vial

For ESI+ target analytes, only native standards were commercially available during the time of this study, so external standard calibration was utilized for these analytes. In-vial concentrations were calculated as a function of the slope of the native standard calibration curve and observed peak area of the analyte. Analyte and native peak areas were normalized to the peak area of an ESI+ NIS, Atrazine-d_5, due to its similarity in retention time and to account for instrument variability (Equation S2).

$$C_{s,Tnative} = \frac{A_{s,Tnative}}{A_{s,ESI+NIS}} / \frac{A_{cal,Tnative}}{A_{cal,ESI+NIS} * C_{cal,Tnative}} \quad \text{Equation S2.}$$

Where,

$\frac{A_{cal,Tnative}}{A_{cal,ESI+NIS} * C_{cal,Tnative}}$ = a term that represents the slope of the calibration curve ($1/x^2$ weight) of the native. The peak area of the native in each calibration point was normalized to the peak area of Atrazine-d_5 in the respective point.

$\frac{A_{s,Tnative}}{A_{s,ESI+NIS}}$ = the peak area of the analyte in the sample normalized to the peak area of Atrazine-d_5 in the respective sample.

$C_{s,Tnative}$ = the calculated concentration of analyte in the sample

Text S4.2. Suspect screening analysis

Qualitative suspect screening involved the comparison of identified mass spectral features against an in-house custom spectral library and a custom extracted ion chromatogram (XIC) list.[4] Together, the library and XIC list contains spectra for >300 AFFF-associated PFASs and molecular formulas/neutral mass for >1,400 PFASs, including a wide variety of well-characterized PFASs, PFASs reported in literature, and in-silico-predicted homologues.[4–6] The suspect screening method used in this study was adopted from previous studies.[1,7] In brief, unknown PFASs were identified based on the molecular ion, isotopic pattern, and the library purity score. Samples were screened by searching for the deprotonated molecular ion $[M-H]^-$ for ESI- analysis and the protonated molecular ion $[M+H]^+$ in ESI+ analysis with parameters: XIC window = 0.01 Da, signal-to-noise threshold = 10:1, minimum peak intensity = 100, and baseline subtraction > 2 min. A MATLAB script (MATLAB Version 24.2.0.2863752 (R2024b) Update 5) was used to process data exported from SCIEX OS and screen suspected hits, including library and XIC list matches. Features matching <10 ppm mass error, <20% isotope ratio difference, and >70% spectral library match based on the SCIEX OS algorithm were considered library matches. Features matching <5 ppm mass error, <10% isotope ratio difference, and <70 spectral library match were considered XIC matches. Both library and XIC matches were considered suspected hits in this study.

Text S4.3. Semiquantitative analysis

A similar semiquantitative analysis approach described previously by Nickerson et al.[1], Hao et al.[7], and Pritchard et al.[3] was performed in this study. Based on suspect screening results, the concentration of suspect hits was estimated using a target "calibrant"-internal standard pair from

those in Table S2. The calibrant-internal standard pair were selected for each suspect hit based on common structural features, such as ionizable functional group and perfluorinated chain length – the target calibrant associated with each suspect hit are shown in Table S3. The estimated, in-vial concentrations of suspect hit compounds were then calculated as a function of the response factor or normalized calibration curve slope of the assigned calibrant-internal standard pair (for ESI- compounds or ESI+ compounds, respectively), the molar masses of both the suspect hit and the calibrant, and the observed peak areas of the internal standard and suspect hit, and the in-vial concentration of the EIS (for ESI- compounds). The semiquantitative approach used for ESI- suspect hits is given by Equation S3 and that used for ESI+ suspect hits is given by Equation S4.

$$\frac{C_{s,\,SQnative}}{C_{s,\,EIS}} = \frac{molar\ mass_{SQnative}}{molar\ mass_{Tnative}} * \frac{A_{s,\,SQnative}}{A_{s,\,EIS}} / \frac{A_{cal,Tnative} * C_{cal,EIS}}{C_{cal,Tnative} * A_{cal,EIS}} \quad \text{Equation S3.}$$

$$C_{s,\,SQnative} = \frac{molar\ mass_{SQnative}}{molar\ mass_{Tnative}} * \frac{A_{s,SQnative}}{A_{sESI+NIS}} / \frac{A_{cal,Tnative}}{A_{cal,ESI+NIS} * C_{cal,Tnative}} \quad \text{Equation S4.}$$

Where,

$A_{s,\,SQnative}$ = the observed peak area of the suspect hit

$molar\ mass_{SQnative}$ = the molar mass of the suspect hit

$molar\ mass_{Tnative}$ = the molar mass of the target calibrant

$C_{s,\,SQnative}$ = the estimated, in-vial concentration of the suspect hit

It should be stated that all semiquantified concentrations are estimates and may be over- or underestimated from their true concentration in the sample. Though both concentrations from both classes of compounds are affected, this is particularly true for the those measured in ESI+ mode due to the limited number of native standards and lack of isotopically-labeled standards.

Table S1. Additional, tabulated information on cores used in this study

Soil pH measurements were conducted by creating a sediment slurry using equal volumes of soil and dilute calcium chloride solution and measuring pH using an electronic probe. Soil pH measurements could not be conducted for all samples due to limited mass of soil.

Core	Total Depth of Core, m bgs	Diameter of core, cm	Number of Segments	Depth of Shallowest Segment, cm bgs	TOC of Shallowest Segment, %	pH of Shallowest Segment
C01	1.5	15.2	19	7.6	0.65	--
C02	1.5	5.7	10	15.2	0.13	7.72
C03	1.5	5.7	10	15.2	0.47	7.73
C04A	1.5	5.7	10	15.2	0.33	7.71
C04B	1.4	5.7	9	15.2	<0.20	--
C05	1.5	5.7	10	15.2	0.36	7.31
C06	0.6	5.7	6	9.1	0.59	7.76
C07	1.2	5.7	12	9.1	1.3	4.81
C08	0.9	5.7	6	15.2	4.9	5.25
C09A	1.5	5.7	10	15.2	1.1	6.29
C09B	1.5	5.7	10	15.2	0.35	7.58
C10	1.8	5.7	6	30.5	0.54	7.67

Table S2. List of Targeted PFAS analytes and internal standards

Component Name	Acronym	Class	Neutral Molecular Formula	Charge	ESI Mode	Internal Standard	Soil LOQ Range, ng/g
perfluorobutane sulfonamide	FBSA	FASA	C4H2F9NO2S	Anionic	(-)	$^{13}C_8$-FOSA	0.55-0.62
perfluoropentane sulfonamide	FPeSA	FASA	C5H2F11NO2S	Anionic	(-)	$^{13}C_8$-FOSA	0.55-0.59
perfluorohexane sulfonamide	FHxSA	FASA	C6H2F13NO2S	Anionic	(-)	$^{13}C_8$-FOSA	0.55-11.12
perfluoroheptane sulfonamide	FHpSA	FASA	C7H2F15NO2S	Anionic	(-)	$^{13}C_8$-FOSA	0.55-571.48
perfluorooctane sulfonamide	FOSA	FASA	C8H2F17NO2S	Anionic	(-)	$^{13}C_8$-FOSA	0.55-1.18
perfluoro-n-butanoic acid	PFBA	PFCA	C4HF7O2	Anionic	(-)	$^{13}C_4$-PFBA	3.01-46.12
perfluoro-n-pentanoic acid	PFPeA	PFCA	C5HF9O2	Anionic	(-)	$^{13}C_5$-PFPeA	2.44-23.70
perfluoro-n-hexanoic acid	PFHxA	PFCA	C6HF11O2	Anionic	(-)	$^{13}C_2$-PFHxA	2.77-3.41
perfluoro-n-heptanoic acid	PFHpA	PFCA	C7HF13O2	Anionic	(-)	$^{13}C_4$-PFHpA	0.56-4.61
perfluoro-n-octanoic acid	PFOA	PFCA	C8HF15O2	Anionic	(-)	$^{13}C_4$-PFOA	3.46-3.50
perfluoro-n-nonanoic acid	PFNA	PFCA	C9HF17O2	Anionic	(-)	$^{13}C_5$-PFNA	0.55-0.62
perfluoro-n-decanoic acid	PFDA	PFCA	C10HF19O2	Anionic	(-)	$^{13}C_2$-PFDA	0.56-1.69
perfluoro-n-undecanoic acid	PFUdA	PFCA	C11HF21O2	Anionic	(-)	$^{13}C_2$-PFUdA	0.55-0.73
perfluoro-n-dodecanoic acid	PFDoA	PFCA	C12HF23O2	Anionic	(-)	$^{13}C_2$-PFDoA	0.01-0.73
perfluoro-n-tridecanoic acid	PFTrDA	PFCA	C13HF25O2	Anionic	(-)	$^{13}C_2$-PFTeDA	0.01-0.73
perfluoro-n-tetradacanoic acid	PFTeDA	PFCA	C14HF27O2	Anionic	(-)	$^{13}C_2$-PFTeDA	0.01-0.73
perfluoro-n-hexadecanoic acid	PFHxDA	PFCA	C16HF31O2	Anionic	(-)	$^{13}C_2$-PFHxDA	0.55-0.73
perfluoro-n-octadecanoic acid	PFODA	PFCA	C18HF35O2	Anionic	(-)	$^{13}C_2$-PFHxDA	3.56-3.69
perfluoropropane sulfonate	PFPrS	PFSA	C3HF7O3S	Anionic	(-)	$^{13}C_3$-PFBS	0.55-0.73
perfluorobutane sulfonate	PFBS	PFSA	C4HF9O3S	Anionic	(-)	$^{13}C_3$-PFBS	0.56-972.73
perfluoropentane sulfonate	PFPeS	PFSA	C5HF11O3S	Anionic	(-)	$^{13}C_2$-PFOS	0.55-0.70
perfluorohexane sulfonate	PFHxS[i]	PFSA	C6HF13O3S	Anionic	(-)	$^{18}O_2$-PFHxS	0.55-1.58
perfluoroheptane sulfonate	PFHpS	PFSA	C7HF15O3S	Anionic	(-)	$^{18}O_2$-PFHxS	0.55-0.59
perfluorooctane sulfonate	PFOS[i]	PFSA	C8HF17O3S	Anionic	(-)	$^{13}C_4$-PFOS	4.19-5.37
perfluorononane sulfonate	PFNS	PFSA	C9HF19O3S	Anionic	(-)	$^{13}C_4$-PFOS	0.55-0.72
perfluorodecane sulfonate	PFDS	PFSA	C10HF21O3S	Anionic	(-)	$^{13}C_4$-PFOS	0.55-0.73
perfluorododecane sulfonate	PFDoS	PFSA	C12HF25O3S	Anionic	(-)	$^{13}C_4$-PFOS	0.01-11.28
chloro-perfluorooctane sulfonate	Cl-PFOS	Cl-PFSA	C8HClF16O3S	Anionic	(-)	$^{13}C_4$-PFOS	0.55-1.44
9-chloro-3-oxa-perfluorononane sulfonate	Cl-O-PFNS	Cl-PFSA	C8HClF16O4S	Anionic	(-)	$^{13}C_4$-PFOS	0.55-1.44
11-chloro-3-oxa-perfluoroundecane sulfonate	Cl-O-PFUdS	Cl-PFSA	C10HClF20O4S	Anionic	(-)	$^{13}C_4$-PFOS	0.55-1.44
2-(N-ethylperfluoro-1-octanesulfonamido)ethanol	EtFOSE	EtFASE	C12H10F17NO3S	Anionic	(-)	d_9-N-EtFOSE-M	2.77-3.41
2-(N-methylperfluoro-1-octanesulfonamido)ethanol	MeFOSE	MeFASE	C11H8F17NO3S	Anionic	(-)	d_7-N-MeFOSE-M	3.46-3.50
N-methyl perfluoro-1-octane sulfonamide	MeFOSA	MeFASA	C9H4F17NO2S	Anionic	(-)	d_3-MeFOSA	0.55-0.72
N-ethylperfluoro-1-octane sulfonamide	EtFOSA	EtFASA	C10H6F17NO2S	Anionic	(-)	d_5-EtFOSA	0.01-0.73
perfluorooctane sulfonamido acetic acid	FOSAA	FASAA	C10H4F17NO4S	Anionic	(-)	d_3-MeFOSAA	0.01-57.26

Component Name	Acronym	Class	Neutral Molecular Formula	Charge	ESI Mode	Internal Standard	Soil LOQ Range, ng/g
N-methylperfluorooctane sulfonamido acetic acid	MeFOSAA[i]	MeFASAA	C11H6F17NO4S	Anionic	(-)	d_3-MeFOSAA	0.55-0.59
N-ethylperfluorooctane sulfonamido acetic acid	EtFOSAA[i]	EtFASAA	C12H8F17NO4S	Anionic	(-)	d_5-EtFOSAA	0.55-0.59
4:2 fluorotelomer sulfonate	4:2 FTS	X:2 FTS	C6H5F9O3S	Anionic	(-)	$^{13}C_2$-4:2 FTS	0.55-1.25
6:2 fluorotelomer sulfonate	6:2 FTS	X:2 FTS	C8H5F13O3S	Anionic	(-)	$^{13}C_2$-6:2 FTS	0.55-1.55
8:2 fluorotelomer sulfonate	8:2 FTS	X:2 FTS	C10H5F17O3S	Anionic	(-)	$^{13}C_2$-8:2 FTS	0.12-0.59
10:2 fluorotelomer sulfonate	10:2 FTS	X:2 FTS	C12H5F21O3S	Anionic	(-)	$^{13}C_2$-8:2 FTS	0.24-5.73
3:3 fluorotelomer carboxylic acid	3:3 FTCA	X:3 FTCA	C6H5F7O2	Anionic	(-)	$^{13}C_2$-6:2 FTCA	0.06-7.33
5:3 fluorotelomer carboxylic acid	5:3 FTCA	X:3 FTCA	C8H5F11O2	Anionic	(-)	$^{13}C_2$-8:2 FTCA	0.06-7.33
7:3 fluorotelomer carboxylic acid	7:3 FTCA	X:3 FTCA	C10H5F15O2	Anionic	(-)	$^{13}C_2$-10:2 FTCA	0.06-7.33
6:2 fluorotelomer carboxylic acid	6:2 FTCA[ii]	X:2 FTCA	C8H3F13O2	Anionic	(-)	$^{13}C_2$-6:2 FTCA	0.55-0.72
8:2 fluorotelomer carboxylic acid	8:2 FTCA[ii]	X:2 FTCA	C10H3F17O2	Anionic	(-)	$^{13}C_2$-8:2 FTCA	0.55-0.72
10:2 fluorotelomer carboxylic acid	10:2 FTCA[ii]	X:2 FTCA	C12H3F21O2	Anionic	(-)	$^{13}C_2$-10:2 FTCA	0.55-0.72
6:2 unsaturated fluorotelomer carboxylic acid	6:2 UFTCA[ii]	X:2 UFTCA	C8H2F12O2	Anionic	(-)	$^{13}C_2$-6:2 UFTCA	0.55-0.62
8:2 unsaturated fluorotelomer carboxylic acid	8:2 UFTCA[ii]	X:2 UFTCA	C10H2F16O2	Anionic	(-)	$^{13}C_2$-8:2 UFTCA	0.55-0.62
10:2 unsaturated fluorotelomer carboxylic acid	10:2 UFTCA[ii]	X:2 UFTCA	C12H2F20O2	Anionic	(-)	$^{13}C_2$-10:2 UFTCA	0.55-0.62
dodecafluoro-3H-4,8-dioxanonanoate	ADONA	PFAA Ethers	C7H2F12O4	Anionic	(-)	$^{13}C_4$-PFOA	3.46-3.50
Perfluoro-4-ethylcyclohexanesulfonate	PFEtCHxS	CHxS	C8HF15O3S	Anionic	(-)	$^{13}C_4$-PFOS	0.55-0.70
tetrafluoro-2-(heptafluoropropoxy)propanoic acid	HFPO-DA	PFAA Ethers	C6HF11O3	Anionic	(-)	$^{13}C_3$-HFPO-DA	0.12-0.59
potassium perfluoro(2-ethoxyethane)sulfonate	PFEESA	PFAA Ethers	C4F9KO4S	Anionic	(-)	$^{13}C_3$-HFPO-DA	0.12-0.59
perfluoro-4-oxapentanoic acid	PFMPA	PFAA Ethers	C4HF7O3	Anionic	(-)	$^{13}C_3$-HFPO-DA	0.12-0.59
perfluoro-5-oxahexanoic acid	PFMBA	PFAA Ethers	C5HF9O3	Anionic	(-)	$^{13}C_3$-HFPO-DA	0.12-0.59
perfluoro-3,6-dioxaheptanoic acid	NFDHA	PFAA Ethers	C5HF9O4	Anionic	(-)	$^{13}C_3$-HFPO-DA	0.12-0.59
N-dimethyl ammonio propyl perfluorohexane sulfonamide	AmPr-FHxSA	AmPr-FASA	C11H13F13N2O2S	Zwitterionic	(+)	Atrazine-d_5	1.12-12.15
N-dimethyl ammonio propyl perfluorooctane sulfonamide	AmPr-FOSA[iv]	AmPr-FASA	C13H13F17N2O2S	Zwitterionic	(+)	Atrazine-d_5	1.12-12.15
N-dimethyl ammonio propyl perfluorohexane sulfonamido propanoic acid	AmPr-FHxSA-PrA[iv]	AmPr-FASA-PrA	C14H17F13N2O4S	Zwitterionic	(-)	Atrazine-d_5	1.12-12.15
N-dimethyl ammonio propyl perfluorooctane sulfonamido propanoic acid	AmPr-FOSA-PrA[iv]	AmPr-FASA-PrA	C16H17F17N2O4S	Zwitterionic	(-)	Atrazine-d_5	1.12-12.15
N-dimethyl ammonio propyl-perfluorohexane amide	PFHxAAm[v]	PFAAAm	C11H13F11N2O	Zwitterionic	(-)	Atrazine-d_5	0.55-0.62
6:2 fluorotelomer sulfonamido propyl betaine	6:2 FTSA-PrB	X:2 FTSA-PrB	C15H19F13N2O4S	Zwitterionic	(+)	Atrazine-d_5	1.13-24.29
N-Trimethylammoniopropyl perfluorobutane sulfonamide	TAmPr-FBSA[v]	TAmPr-FASA	C10H15F9N2O2S	Cationic	(+)	Atrazine-d_5	1.13-12.85
N-Trimethylammoniopropyl perfluorohexane sulfonamide	TAmPr-FHxSA	TAmPr-FASA	C12H15F13N2O2S	Cationic	(+)	Atrazine-d_5	1.13-12.85
5:3 fluorotelomer betaine	5:3 FTB	X:3 FTB	C12H14F11NO2	Cationic	(+)	Atrazine-d_5	1.12-12.15
6:2 hydrido-fluorotelomer betaine	6:2 H-FTB	X:2 H-FTB	C12H13F12NO2	Cationic	(+)	Atrazine-d_5	1.12-12.15

i. Exist in the standard as the linear and branched isomers.

ii. Stored in 100% Optima™ LC/MS Grade 2-isopropanol to limit potential degradation. All other compounds stored in 100% Optima™ LC/MS Grade methanol.
iii. Since no isotopically-labeled internal standards were available for the ESI+ target analytes at the time of this study, Atrazine-d_5 (CDN Isotopes, Pointe-Claire, Quebec, Canada) was used as internal standard due to similar elution time.
iv. Standard acquired from SynQuest Laboratories (Alachua, Florida, United States of America). All other standards were acquired from Wellington Laboratories (Guelph, Ontario, Canada) unless otherwise stated.
v. Standard acquired from Chiron (Trondheim, Trøndelag, Norway). All other standards were acquired from Wellington Laboratories (Guelph, Ontario, Canada) unless otherwise stated.

Table S3. List of Suspect Hit PFASs and their target calibrants

Component Name	Acronym	Class	Neutral Molecular Formula	Charge	ESI Mode	PCI Level	Target Calibrant	LOQ Range, ng/g
N-sulfo propyl perfluoropropane sulfonamide	SPr-FPrSA	SPr-FASA	C6H8F7NO5S2	Anionic	(-)	2a	EtFOSA	0.01-1.57
N-sulfo propyl perfluorobutane sulfonamide	SPr-FBSA	SPr-FASA	C7H8F9NO5S2	Anionic	(-)	2a	EtFOSA	0.01-1.57
N-sulfo propyl perfluoropentane sulfonamide	SPr-FPeSA	SPr-FASA	C8H8F11NO5S2	Anionic	(-)	3b	EtFOSA	0.01-1.57
N-sulfo propyl perfluorohexane sulfonamide	SPr-FHxSA	SPr-FASA	C9H8F13NO5S2	Anionic	(-)	2a	EtFOSA	0.60-1.57
N-Carboxymethyldimethylammoniopropyl-perfluorobutanesulfonamide	CMeAmPr-FBSA	CMeAmPr-FASA	C11H15F9N2O4S	Zwitterionic	(+)	3c	6:2 FTSA-PrB	12.15-24.29
N-Carboxymethyldimethylammoniopropyl-perfluorohexanesulfonamide	CMeAmPr-FHxSA	CMeAmPr-FASA	C13H15F13N2O4S	Zwitterionic	(+)	2b	6:2 FTSA-PrB	12.15-24.29
N-sulfo propyl dimethyl ammonio propyl perfluoropropane sulfonamide	SPrAmPr-FPrSA	SPrAmPr-FASA	C11H19F7N2O5S2	Zwitterionic	(-)	2a	EtFOSA	0.01-1.57
N-sulfo propyl dimethyl ammonio propyl perfluorobutane sulfonamide	SPrAmPr-FBSA	SPrAmPr-FASA	C12H19F9N2O5S2	Zwitterionic	(-)	2a	EtFOSA	0.01-1.57
N-sulfo propyl dimethyl ammonio propyl perfluoropentane sulfonamide	SPrAmPr-FPeSA	SPrAmPr-FASA	C13H19F11N2O5S2	Zwitterionic	(-)	2c	EtFOSA	0.01-1.57
N-sulfo propyl dimethyl ammonio propyl perfluorohexane sulfonamide	SPrAmPr-FHxSA	SPrAmPr-FASA	C14H19F13N2O5S2	Zwitterionic	(-)	2a	EtFOSA	0.01-1.57
PentaFluoroSulfide perfluorooctane sulfonate	F5S-PFOS	F5S-PFSA	C8HF21O3S2	Anionic	(-)	2b	PFOS	0.60-0.80
hydrido-unsaturated perfluorooctane sulfonate	H-UPFOS	H-UPFSA	C8H2F14O3S	Anionic	(-)	2b	PFHpS	0.12-0.80
Hydrido-PerFluoroPentane Sulfonate	H-PFPeS	H-PFSA	C5H2F10O3S	Anionic	(-)	3c	PFPeS	0.01-1.57
Hydrido-PerFluoroHexane Sulfonate	H-PFHxS	H-PFSA	C6H2F12O3S	Anionic	(-)	3c	PFHxS	0.01-1.57
Hydrido-PerFluoroOctane Sulfonate	H-PFOS	H-PFSA	C8H2F16O3S	Anionic	(-)	3c	PFOS	0.12-1.57
N-sulfohydroxypropyl dimethylammonio propyl perfluorobutane sulfonamido hydroxy propyl sulfonate	S-OHPrAmPr-FBSA-OHPrS	S-OHPrAmPr-FASA-OHPrS	C15H25F9N2O10S3	Zwitterionic	(-)	2b	EtFOSAA	0.12-7.96
N-sulfohydroxypropyl dimethylammonio propyl perfluorohexane sulfonamido hydroxy propyl sulfonate	S-OHPrAmPr-FHxSA-OHPrS	S-OHPrAmPr-FASA-OHPrS	C17H25F13N2O10S3	Zwitterionic	(-)	2a	EtFOSAA	0.12-7.96
N-sulfo hydroxypropyl dimethylammonio propyl perfluorobutane sulfonamide	S-OHPrAmPr-FBSA	S-OHPrAmPr-FASA	C12H19F9N2O6S2	Zwitterionic	(-)	2a	EtFOSA	0.01-1.57
N-sulfo hydroxypropyl dimethylammonio propyl perfluoropentane sulfonamide	S-OHPrAmPr-FPeSA	S-OHPrAmPr-FASA	C13H19F11N2O6S2	Zwitterionic	(-)	2c	EtFOSA	0.01-1.57
N-sulfo hydroxypropyl dimethylammonio propyl perfluorohexane sulfonamide	S-OHPrAmPr-FHxSA	S-OHPrAmPr-FASA	C14H19F13N2O6S2	Zwitterionic	(-)	2a	EtFOSA	0.01-6.41
N-sulfopropyldimethylammoniopropyl-perfluoropropane sulfonamido acetic acid	SPrAmPr-FPrSAA	SPrAmPr-FASAA	C13H21F7N2O7S2	Zwitterionic	(-)	2b	EtFOSAA	0.12-7.96
N-sulfopropyldimethylammoniopropyl-perfluorobutane sulfonamido acetic acid	SPrAmPr-FBSAA	SPrAmPr-FASAA	C14H21F9N2O7S2	Zwitterionic	(-)	3c	EtFOSAA	0.12-7.96
8:2 fluorotelomersulfonyl propanoic acid	8:2 FTSO2PrA	X:2 FTSO2PrA	C13H9F17O4S	Anionic	(-)	3c	8:2 FTCA	0.60-7.96
N-sulfopropyl dimethylammoniopropyl perfluoropropane sulfonamido propyl sulfonate	SPrAmPr-FPrSAPrS	SPrAmPr-FASAPrS	C14H25F7N2O8S3	Zwitterionic	(-)	2a	EtFOSAA	0.12-7.96
N-sulfopropyl dimethylammoniopropyl perfluorobutane sulfonamido propyl sulfonate	SPrAmPr-FBSAPrS	SPrAmPr-FASAPrS	C15H25F9N2O8S3	Zwitterionic	(-)	2a	EtFOSAA	0.12-7.96

Component Name	Acronym	Class	Neutral Molecular Formula	Charge	ESI Mode	PCI Level	Target Calibrant	LOQ Range, ng/g
N-sulfopropyl dimethylammoniopropyl perfluoropentane sulfonamido propyl sulfonate	SPrAmPr-FPeSAPrS	SPrAmPr-FASAPrS	C16H25F11N2O8S3	Zwitterionic	(-)	2c	EtFOSAA	0.12-7.96
N-sulfopropyl dimethylammoniopropyl perfluorohexane sulfonamido propyl sulfonate	SPrAmPr-FHxSAPrS	SPrAmPr-FASAPrS	C17H25F13N2O8S3	Zwitterionic	(-)	2a	EtFOSAA	0.12-7.96
N-dihydroxy propyl dimethyl ammonio hydroxymethyl propyl-perfluorobutanesulfonamide	diOHPrAm-MeOHPr-FBSA	diOHPrAm-MeOHPr-FASA	C13H21F9N2O5S	Anionic	(-)	2a	EtFOSA	0.01-1.36
N-dihydroxy propyl dimethyl ammonio hydroxymethyl propyl-perfluoropentanesulfonamide	diOHPrAm-MeOHPr-FPeSA	diOHPrAm-MeOHPr-FASA	C14H21F11N2O5S	Anionic	(-)	2a	EtFOSA	0.01-1.36
N-dihydroxy propyl dimethyl ammonio hydroxymethyl propyl-perfluorohexanesulfonamide	diOHPrAm-MeOHPr-FHxSA	diOHPrAm-MeOHPr-FASA	C15H21F13N2O5S	Anionic	(-)	2a	EtFOSA	0.01-1.28
N-dihydroxy propyldimethyl ammoniohydroxymethyl propyl-perfuorobutane sulfonamido propyl sulfonate	diOHPrAm-MeOHPr-FBSAPrS	diOHPrAm-MeOHPr-FASAPrS	C16H27F9N2O8S2	Anionic	(-)	2a	EtFOSAA	0.12-7.96
N-dihydroxy propyldimethyl ammoniohydroxymethyl propyl-perfluoropentane sulfonamidopropyl sulfonate	diOHPrAm-MeOHPr-FPeSAPrS	diOHPrAm-MeOHPr-FASAPrS	C17H27F11N2O8S2	Anionic	(-)	2a	EtFOSAA	0.12-7.96
N-dihydroxy propyldimethyl ammoniohydroxymethyl propyl-perfluorohexane sulfonamido propyl sulfonate	diOHPrAm-MeOHPr-FHxSAPrS	diOHPrAm-MeOHPr-FASAPrS	C18H27F13N2O8S2	Anionic	(-)	2a	EtFOSAA	0.12-7.96
Chloro-perfluorobutane sulfonate	Cl-PFBS	Cl-PFSA	C4HClF8O3S	Anionic	(-)	3c	PFBS	0.01-0.80
Chloro-perfluorohexane sulfonate	Cl-PFHxS	Cl-PFSA	C6HClF12O3S	Anionic	(-)	3c	PFHxS	0.01-1.57
perfluorobutane sulfinate	PFBSi	PFSAi	C4HF9O2S	Anionic	(-)	2a	PFBS	0.01-0.80
perfluoropentane sulfinate	PFPeSi	PFSAi	C5HF11O2S	Anionic	(-)	3c	PFPeS	0.01-0.80
perfluorohexane sulfinate	PFHxSi	PFSAi	C6HF13O2S	Anionic	(-)	2a	PFHxS	0.60-1.36
perfluorooctane sulfinate	PFOSi	PFSAi	C8HF17O2S	Anionic	(-)	2b	PFOS	0.12-1.28
4:2 fluorotelomer sulfonyl propanoamido-dimethylethyl sulfonate	4:2 FTSO2PrAd-DiMeEtS	X:2 FTSO2PrAd-DiMeEtS	C13H18F9NO6S2	Zwitterionic	(-)	2b	4:2 FTS	0.60-0.80
6:2 fluorotelomer sulfonyl propanoamido-dimethylethyl sulfonate	6:2 FTSO2PrAd-DiMeEtS	X:2 FTSO2PrAd-DiMeEtS	C15H18F13NO6S2	Zwitterionic	(-)	2b	6:2 FTS	0.60-1.36
8:2 fluorotelomer sulfonyl propanoamido-dimethylethyl sulfonate	8:2 FTSO2PrAd-DiMeEtS	X:2 FTSO2PrAd-DiMeEtS	C17H18F17NO6S2	Zwitterionic	(-)	2b	8:2 FTS	0.60-0.80
6:2 fluorotelomer thia propanoamido dimethyl ethyl sulfonate	6:2 FTTh-PrAd-DiMeEtS	X:2 FTTh-PrAd-DiMeEtS	C15H18F13NO4S2	Zwitterionic	(-)	3c	6:2 FTS	0.16-1.57
8:2 fluorotelomer thia propanoamido dimethyl ethyl sulfonate	8:2 FTTh-PrAd-DiMeEtS	X:2 FTTh-PrAd-DiMeEtS	C17H18F17NO4S2	Zwitterionic	(-)	2a	8:2 FTS	0.12-0.80
Keto-perfluoropentane sulfonate	K-PFPeS	K-PFSA	C5HF9O4S	Anionic	(-)	3c	PFBS	0.01-0.80
Keto-perfluorohexane sulfonate	K-PFHxS	K-PFSA	C6HF11O4S	Anionic	(-)	3c	PFPeS	0.01-0.80
Keto-perfluoroheptane sulfonate	K-PFHpS	K-PFSA	C7HF13O4S	Anionic	(-)	2b	PFHxS	0.01-1.57
Keto-perfluorooctane sulfonate	K-PFOS	K-PFSA	C8HF15O4S	Anionic	(-)	2b	PFHpS	0.60-1.36
perfluoropropane sulfonamide	FPrSA	FASA	C3H2F7NO2S	Anionic	(-)	2a	FBSA	0.60-0.78
N-ethylperfluorobutane sulfonamido acetic acid	EtFBSAA	EtFASAA	C8H8F9NO4S	Anionic	(-)	3c	EtFOSAA	0.12-7.96
N-ethylperfluoropentane sulfonamido acetic acid	EtFPeSAA	EtFASAA	C9H8F11NO4S	Anionic	(-)	3c	EtFOSAA	0.12-7.96

Component Name	Acronym	Class	Neutral Molecular Formula	Charge	ESI Mode	PCI Level	Target Calibrant	LOQ Range, ng/g
N-ethylperfluorohexane sulfonamido acetic acid	EtFHxSAA	EtFASAA	C10H8F13NO4S	Anionic	(-)	3c	EtFOSAA	0.12-7.96
Perfluorobutane sulfonamido acetic acid	FBSAA	FASAA	C6H4F9NO4S	Anionic	(-)	2b	FOSAA	0.01-0.80
Perfluoropentane sulfonamido acetic acid	FPeSAA	FASAA	C7H4F11NO4S	Anionic	(-)	3c	FOSAA	0.01-0.80
Perfluorohexane sulfonamido acetic acid	FHxSAA	FASAA	C8H4F13NO4S	Anionic	(-)	2b	FOSAA	0.60-0.80
N-dimethyl ammonio propyl perfluoroethane sulfonamido propanoic acid	AmPr-FEtSA-PrA	AmPr-FASA-PrA	C10H17F5N2O4S	Zwitterionic	(-)	3c	EtFOSAA	0.12-7.96
N-dimethyl ammonio propyl perfluoropropane sulfonamido propanoic acid	AmPr-FPrSA-PrA	AmPr-FASA-PrA	C11H17F7N2O4S	Zwitterionic	(-)	2a	EtFOSAA	0.12-7.96
N-dimethyl ammonio propyl perfluorbutoane sulfonamido propanoic acid	AmPr-FBSA-PrA	AmPr-FASA-PrA	C12H17F9N2O4S	Zwitterionic	(-)	2a	EtFOSAA	0.12-7.96
N-dimethyl ammonio propyl perfluorohexane sulfonamido propanoic acid	AmPr-FHxSA-PrA	AmPr-FASA-PrA	C14H17F13N2O4S	Zwitterionic	(-)	1a	EtFOSAA	0.12-7.96
N-dimethyl ammonio propyl perfluoropropane sulfonamide	AmPr-FPrSA	AmPr-FASA	C8H13F7N2O2S	Zwitterionic	(+)	3c	AmPr-FHxSA	1.14-12.15
N-dimethyl ammonio propyl perfluorobutane sulfonamide	AmPr-FBSA	AmPr-FASA	C9H13F9N2O2S	Zwitterionic	(+)	2a	AmPr-FHxSA	1.21-12.15
N-dimethyl ammonio propyl perfluoropentane sulfonamide	AmPr-FPeSA	AmPr-FASA	C10H13F11N2O2S	Zwitterionic	(+)	2a	AmPr-FHxSA	1.14-12.15
N-dimethyl ammonio propyl perfluoroheptane sulfonamide	AmPr-FHpSA	AmPr-FASA	C12H13F15N2O2S	Zwitterionic	(+)	2a	AmPr-FHxSA	1.21-12.15
Unsaturated perfluorohexane sulfonate	UPFHxS	UPFSA	C6HF11O3S	Anionic	(-)	2c	PFPeS	0.01-0.80
Unsaturated perfluoroheptane sulfonate	UPFHpS	UPFSA	C7HF13O3S	Anionic	(-)	3c	PFHxS	0.12-1.57
Unsaturated perfluorooctane sulfonate	UPFOS	UPFSA	C8HF15O3S	Anionic	(-)	2a	PFHpS	0.12-1.36
Unsaturated perfluorononane sulfonate	UPFNS	UPFSA	C9HF17O3S	Anionic	(-)	2b	PFOS	0.12-1.57
Unsaturated perfluorodecane sulfonate	UPFDS	UPFSA	C10HF19O3S	Anionic	(-)	2a	PFNS	0.60-0.80
perfluoropentane sulfate	PFPe-OS	PFA-OS	C5HF11O4S	Anionic	(-)	3c	PFPeS	0.01-0.80
perfluorohexane sulfate	PFHx-OS	PFA-OS	C6HF13O4S	Anionic	(-)	3c	PFHxS	0.01-1.57
perfluoroheptane sulfate	PFHp-OS	PFA-OS	C7HF15O4S	Anionic	(-)	2b	PFHpS	0.01-0.80
perfluorooctane sulfate	PFO-OS	PFA-OS	C8HF17O4S	Anionic	(-)	3c	PFOS	0.60-1.57
perfluorononane sulfate	PFN-OS	PFA-OS	C9HF19O4S	Anionic	(-)	2b	PFNS	0.60-0.80
1-hydroxy-4:2 fluorotelomer sulfonate	1OH-4:2 FTS	1OH-X:2 FTS	C6H5F9O4S	Anionic	(-)	3b	4:2 FTS	0.60-1.57
1-hydroxy-6:2 fluorotelomer sulfonate	1OH-6:2 FTS	1OH-X:2 FTS	C8H5F13O4S	Anionic	(-)	2b	6:2 FTS	0.12-1.57

Component Name	Acronym	Class	Neutral Molecular Formula	Charge	ESI Mode	PCI Level	Target Calibrant	LOQ Range, ng/g
6:2 fluorotelomer sulfinate	6:2 FTSi	X:2 FTSi	C8H5F13O2S	Anionic	(-)	2b	6:2 FTS	0.12-1.28
8:2 fluorotelomer sulfinate	8:2 FTSi	X:2 FTSi	C10H5F17O2S	Anionic	(-)	3c	8:2 FTS	0.12-1.28
N-ethylperfluoro-1-hexane sulfonamide	EtFHxSA	EtFASA	C8H6F13NO2S	Anionic	(-)	2b	EtFOSA	0.01-0.78
N-ethylperfluoro-1-decane sulfonamide	EtFDSA	EtFASA	C12H6F21NO2S	Anionic	(-)	3c	EtFOSA	0.01-6.20
N-ethylperfluoro-1-dodecane sulfonamide	EtFDoSA	EtFASA	C14H6F25NO2S	Anionic	(-)	3c	EtFOSA	0.01-6.20
N-methyl perfluoro-1-butane sulfonamide	MeFBSA	MeFASA	C5H4F9NO2S	Anionic	(-)	3c	MeFOSA	0.01-0.80
N-methyl perfluoro-1-pentane sulfonamide	MeFPeSA	MeFASA	C6H4F11NO2S	Anionic	(-)	3c	MeFOSA	0.01-0.80
N-methyl perfluoro-1-hexane sulfonamide	MeFHxSA	MeFASA	C7H4F13NO2S	Anionic	(-)	2b	MeFOSA	0.01-0.80
N-carboxymethyldimethyl ammoniopropyl-perfluoropropane sulfonamido propanoic acid	CMeAmPr-FPrSAPrA	CMeAmPr-FASAPrA	C13H19F7N2O6S	Zwitterionic	(+)	2b	6:2 FTSA-PrB	12.15-24.29
N-carboxymethyldimethyl ammoniopropyl-perfluorobutane sulfonamido propanoic acid	CMeAmPr-FBSAPrA	CMeAmPr-FASAPrA	C14H19F9N2O6S	Zwitterionic	(+)	2b	6:2 FTSA-PrB	12.15-24.29
N-carboxymethyldimethyl ammoniopropyl-perfluorohexane sulfonamido propanoic acid	CMeAmPr-FHxSAPrA	CMeAmPr-FASAPrA	C16H19F13N2O6S	Zwitterionic	(+)	2b	6:2 FTSA-PrB	12.15-24.29
N-carboxymethyl dimethylammoniopropyl-perfluorobutane sulfonamido acetic acid	CMeAmPr-FBSAA	CMeAmPr-FASAA	C13H17F9N2O6S	Zwitterionic	(+)	2b	6:2 FTSA-PrB	12.15-24.29
N-carboxymethyl dimethylammoniopropyl-perfluorohexane sulfonamido acetic acid	CMeAmPr-FHxSAA	CMeAmPr-FASAA	C15H17F13N2O6S	Zwitterionic	(+)	2b	6:2 FTSA-PrB	12.15-24.29
N-carboxyethyl dimethylammoniopropyl-N-ethyl perfluorohexane sulfonamide	CEtAmPr-N-EtFHxSA	CEtAmPr-N-EtFASA	C16H21F13N2O4S	Zwitterionic	(+)	2b	6:2 FTSA-PrB	12.15-24.29
N-dihydroxybutyl dimethylammoniopropyl perfluorobutane sulfonamide	diOHBAmPr-FBSA	diOHBAmPr-FASA	C13H21F9N2O4S	Anionic	(-)	2a	EtFOSA	0.01-1.57
N-dihydroxybutyl dimethylammoniopropyl perfluoropentane sulfonamide	diOHBAmPr-FPeSA	diOHBAmPr-FASA	C14H21F11N2O4S	Anionic	(-)	2a	EtFOSA	0.01-1.57
N-dihydroxybutyl dimethylammoniopropyl perfluorohexane sulfonamide	diOHBAmPr-FHxSA	diOHBAmPr-FASA	C15H21F13N2O4S	Anionic	(-)	2a	EtFOSA	0.01-2.78
N-dihydroxybutyl dimethylammoniopropyl perfluoroheptane sulfonamide	diOHBAmPr-FHpSA	diOHBAmPr-FASA	C16H21F15N2O4S	Anionic	(-)	3c	EtFOSA	0.01-1.57
N-dihydroxybutyl dimethylammoniopropyl perfluorooctane sulfonamide	diOHBAmPr-FOSA	diOHBAmPr-FASA	C17H21F17N2O4S	Anionic	(-)	2b	EtFOSA	0.01-7.47
6:2 unsaturated fluorotelomer sulfonate	6:2 UFTS	X:2 UFTS	C8H4F12O3S	Anionic	(-)	2b	6:2 FTS	0.12-1.57
6:2 fluorotelomer sulfinyl propanamido dimethyl ethyl sulfonate	6:2 FTSO-PrAd-DiMeEtS	X:2 FTSO-PrAd-DiMeEtS	C15H18O5S2NF13	Zwitterionic	(-)	2b	6:2 FTS	0.12-1.36
8:2 fluorotelomer sulfinyl propanamido dimethyl ethyl sulfonate	8:2 FTSO-PrAd-DiMeEtS	X:2 FTSO-PrAd-DiMeEtS	C17H18O5S2NF17	Zwitterionic	(-)	2b	8:2 FTS	0.12-1.36
6:2 fluorotelomer sulfonamido propyl hydroxymethyl methyl amine	6:2 FTSAPr-OHMeMeAn	X:2 FTSAPr-OHMeMeAn	C13H17F13N2O3S	Cationic	(+)	3c	6:2 FTSA-PrB	12.30-24.29

Component Name	Acronym	Class	Neutral Molecular Formula	Charge	ESI Mode	PCI Level	Target Calibrant	LOQ Range, ng/g
N-hydroxyethyl dimethylammoniohydroxypropyl perfluorohexanesulfonamide	EtOH-Am-OHPr-FHxSA	EtOH-Am-OHPr-FASA	C13H17F13N2O4S	Zwitterionic	(+)	3c	6:2 FTSA-PrB	12.15-24.29
N-hydroxyethyldimethylammoniopropyl perfluorobutanesulfonamide	EtOH-AmPr-FBSA	EtOH-AmPr-FASA	C11H17F9N2O3S	Zwitterionic	(+)	2a	6:2 FTSA-PrB	12.15-24.29
N-hydroxyethyldimethylammoniopropyl perfluoropentanesulfonamide	EtOH-AmPr-FPeSA	EtOH-AmPr-FASA	C12H17F11N2O3S	Zwitterionic	(+)	2a	6:2 FTSA-PrB	12.15-24.29
N-hydroxyethyldimethylammoniopropyl perfluorohexanesulfonamide	EtOH-AmPr-FHxSA	EtOH-AmPr-FASA	C13H17F13N2O3S	Zwitterionic	(+)	2a	6:2 FTSA-PrB	12.15-24.29
N-hydroxyethyldimethylammoniopropyl perfluoroheptanesulfonamide	EtOH-AmPr-FHpSA	EtOH-AmPr-FASA	C14H17F15N2O3S	Zwitterionic	(+)	2a	6:2 FTSA-PrB	12.15-24.29
N-hydroxyethyldimethylammoniopropyl perfluorooctanesulfonamide	EtOH-AmPr-FOSA	EtOH-AmPr-FASA	C15H17F17N2O3S	Zwitterionic	(+)	2a	6:2 FTSA-PrB	12.15-24.29
N-hydroxyethyl dimethylammoniopropyl perfluorobutane sulfonamido hydroxy propylsulfonate	EtOH-AmPr-FBSA-OHPrS	EtOH-AmPr-FASA-OHPrS	C14H23F9N2O7S2	Zwitterionic	(+)	2a	6:2 FTSA-PrB	12.15-24.29
N-hydroxyethyl dimethylammoniopropyl perfluoropentane sulfonamido hydroxy propylsulfonate	EtOH-AmPr-FPeSA-OHPrS	EtOH-AmPr-FASA-OHPrS	C15H23F11N2O7S2	Zwitterionic	(+)	2a	6:2 FTSA-PrB	12.15-24.29
N-hydroxyethyl dimethylammoniopropyl perfluorohexane sulfonamido hydroxy propylsulfonate	EtOH-AmPr-FHxSA-OHPrS	EtOH-AmPr-FASA-OHPrS	C16H23F13N2O7S2	Zwitterionic	(+)	2a	6:2 FTSA-PrB	12.15-24.29
N-sulfopropyldimethylammoniopropyl N-methyl perfuorohexanesulfonamide	SPrAmPr-N-Me-FHxSA	SPrAmPr-N-Me-FASA	C15H21F13N2O5S2	Zwitterionic	(+)	2b	6:2 FTSA-PrB	12.30-83.27
N-hydroxyethyl dimethylammoniopropyl perfluorohexane sulfonamido propylsulfonate	EtOH-AmPr-FHxSAPrS	EtOH-AmPr-FASAPrS	C16H23F13N2O6S2	Zwitterionic	(+)	2a	6:2 FTSA-PrB	12.30-380.02
8:2 fluorotelomer sulfonamido propyl betaine	8:2 FTSA-PrB	X:2 FTSA-PrB	C17H19F17N2O4S	Zwitterionic	(+)	3c	6:2 FTSA-PrB	12.15-24.29
N-trimethylammoniopropyl N-methylperfluorobutanesulfonamide	TAmPr-N-MeFBSA	TAmPr-N-MeFASA	C11H17F9N2O2S	Cationic	(+)	2a	TAmPr-FHxSA	2.47-13.87
N-trimethylammoniopropyl N-methylperfluoropentanesulfonamide	TAmPr-N-MeFPeSA	TAmPr-N-MeFASA	C12H17F11N2O2S	Cationic	(+)	2a	TAmPr-FHxSA	2.47-13.87
N-trimethylammoniopropyl N-methylperfluorohexanesulfonamide	TAmPr-N-MeFHxSA	TAmPr-N-MeFASA	C13H17F13N2O2S	Cationic	(+)	2b	TAmPr-FHxSA	2.46-55.81
N-trimethylammoniopropyl N-methylperfluorooctanesulfonamide	TAmPr-N-MeFOSA	TAmPr-N-MeFASA	C15H17F17N2O2S	Cationic	(+)	2b	TAmPr-FHxSA	2.47-16.09
N-Trimethylammoniopropyl perfluoropentane sulfonamide	TAmPr-FPeSA	TAmPr-FASA	C11H15F11N2O2S	Cationic	(+)	2a	TAmPr-FHxSA	2.47-16.09
N-Trimethylammoniopropyl perfluoroheptane sulfonamide	TAmPr-FHpSA	TAmPr-FASA	C13H15F15N2O2S	Cationic	(+)	2a	TAmPr-FHxSA	2.46-16.09
N-Trimethylammoniopropyl perfluorooctane sulfonamide	TAmPr-FOSA	TAmPr-FASA	C14H15F17N2O2S	Cationic	(+)	2a	TAmPr-FHxSA	2.46-13.73
N-ethyl dimethyl ammonio propyl perfluoroethane N-ethyl sulfonamide	EtAmPr-FEt-N-EtSA	EtAmPr-FA-N-EtSA	C11H21F5N2O2S	Cationic	(+)	3c	TAmPr-FHxSA	2.48-16.09
N-ethyl dimethyl ammonio propyl perfluoropropane N-ethyl sulfonamide	EtAmPr-FPr-N-EtSA	EtAmPr-FA-N-EtSA	C12H21F7N2O2S	Cationic	(+)	2b	TAmPr-FHxSA	2.48-13.87
N-ethyl dimethyl ammonio propyl perfluorobutane N-ethyl sulfonamide	EtAmPr-FB-N-EtSA	EtAmPr-FA-N-EtSA	C13H21F9N2O2S	Cationic	(+)	2b	TAmPr-FHxSA	2.48-13.87
N-ethyl dimethyl ammonio propyl perfluoropentane N-ethyl sulfonamide	EtAmPr-FPe-N-EtSA	EtAmPr-FA-N-EtSA	C14H21F11N2O2S	Cationic	(+)	2b	TAmPr-FHxSA	2.48-13.87
N-ethyl dimethyl ammonio propyl perfluorohexane N-ethyl sulfonamide	EtAmPr-FHx-N-EtSA	EtAmPr-FA-N-EtSA	C15H21F13N2O2S	Cationic	(+)	2b	TAmPr-FHxSA	2.48-36.78
N-ethyl dimethyl ammonio propyl perfluoroheptane N-ethyl sulfonamide	EtAmPr-FHp-N-EtSA	EtAmPr-FA-N-EtSA	C16H21F15N2O2S	Cationic	(+)	3c	TAmPr-FHxSA	2.48-16.09
N-ethyl dimethyl ammonio propyl perfluorooctane N-ethyl sulfonamide	EtAmPr-FO-N-EtSA	EtAmPr-FA-N-EtSA	C17H21F17N2O2S	Cationic	(+)	3c	TAmPr-FHxSA	2.48-16.09

Figure S1. Detection frequency heatmap of all PFAAs across cores C02 through C10

Only concentrations above reporting limits were used in this plot. Non-shallow detections, meaning a member of the PFAS class exceeded its reporting limit in at least one depth interval except the shallowest of the core, are represented with light-blue squares. Shallowest-depth detections, meaning a member of the PFAS class exceeded its reporting limit in at least one depth interval including the shallowest of the core, are represented with dark-blue squares.

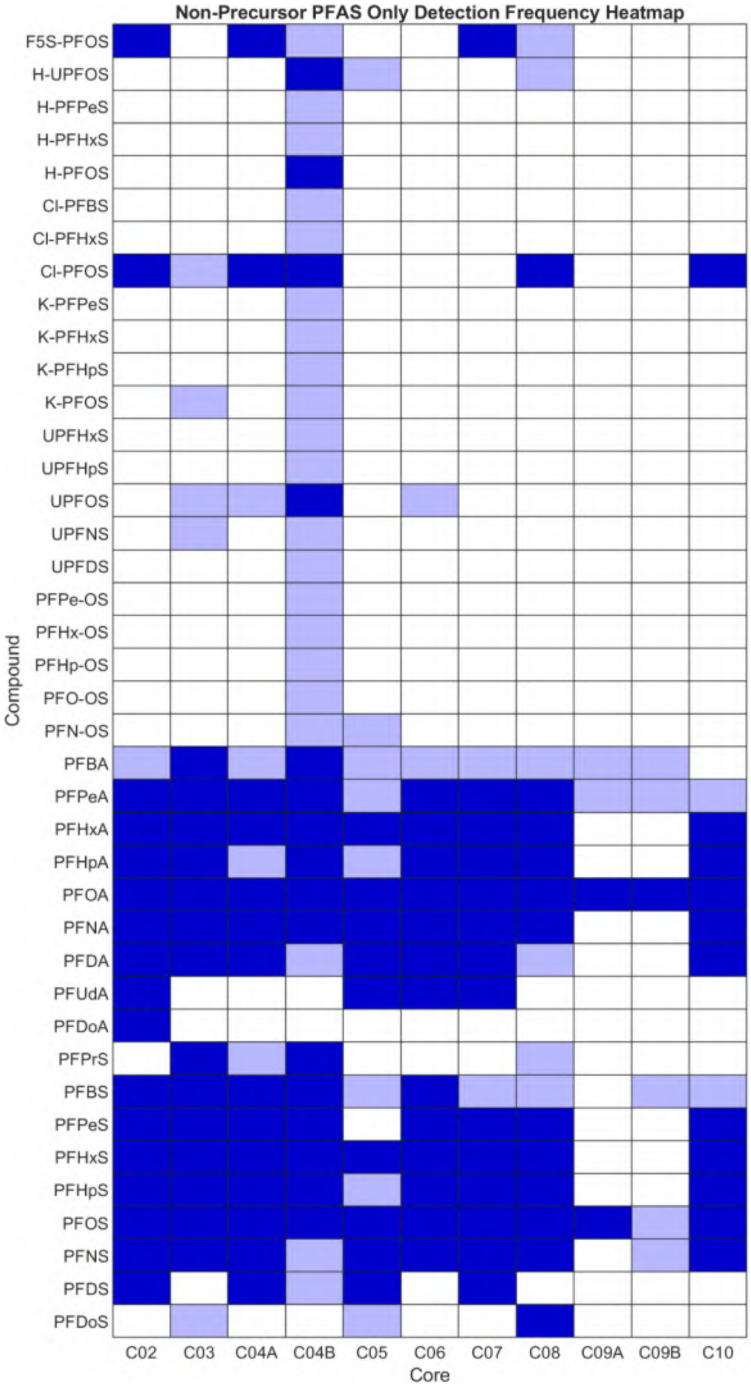

Figure S2. Detection frequency heatmap of all PFAA precursors across cores C02 through C10

Only concentrations above reporting limits were used in this plot. Non-shallow detections, meaning a member of the PFAS class exceeded its reporting limit in at least one depth interval except the shallowest of the core, are represented with light-blue squares. Shallowest-depth detections, meaning a member of the PFAS class exceeded its reporting limit in at least one depth interval including the shallowest of the core, are represented with dark-blue squares.

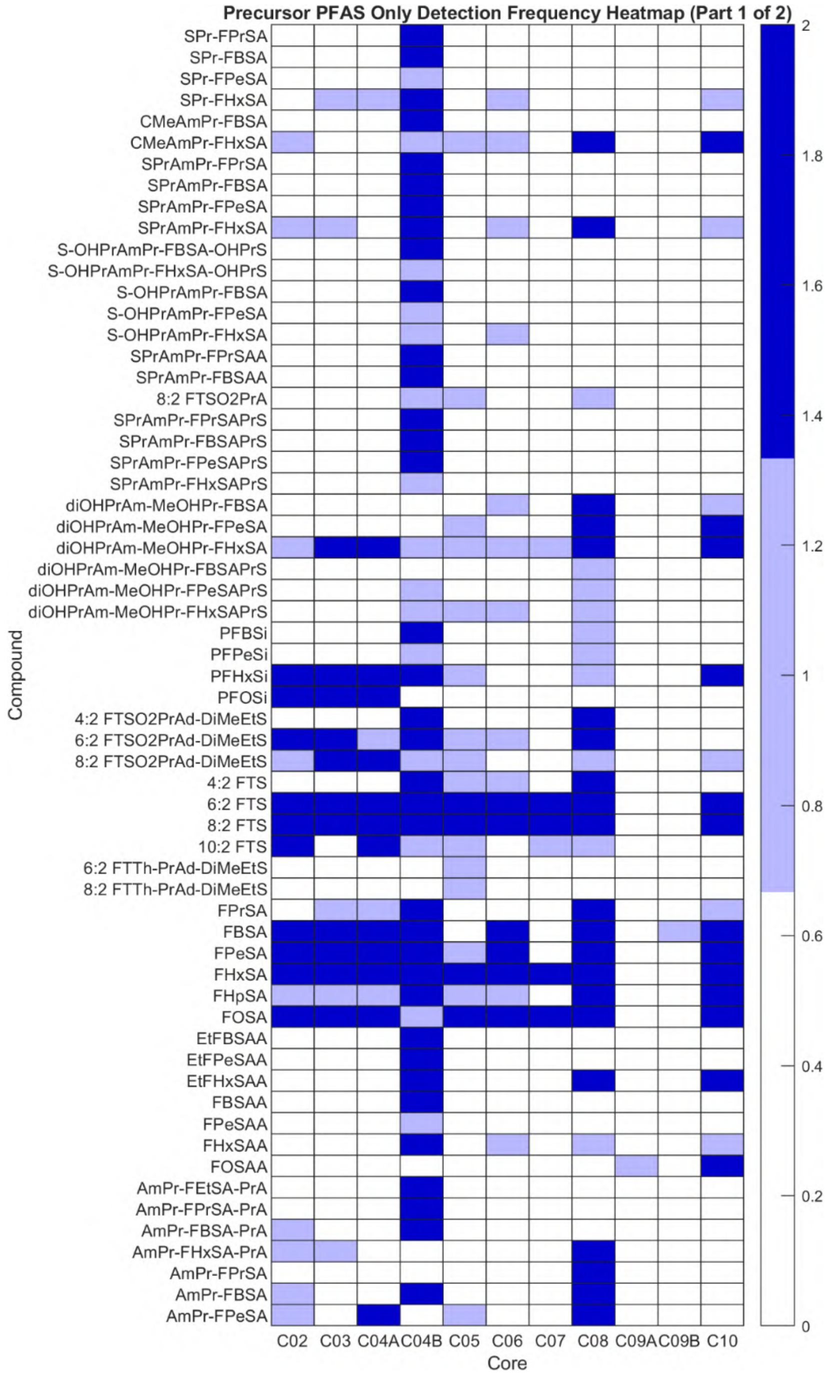

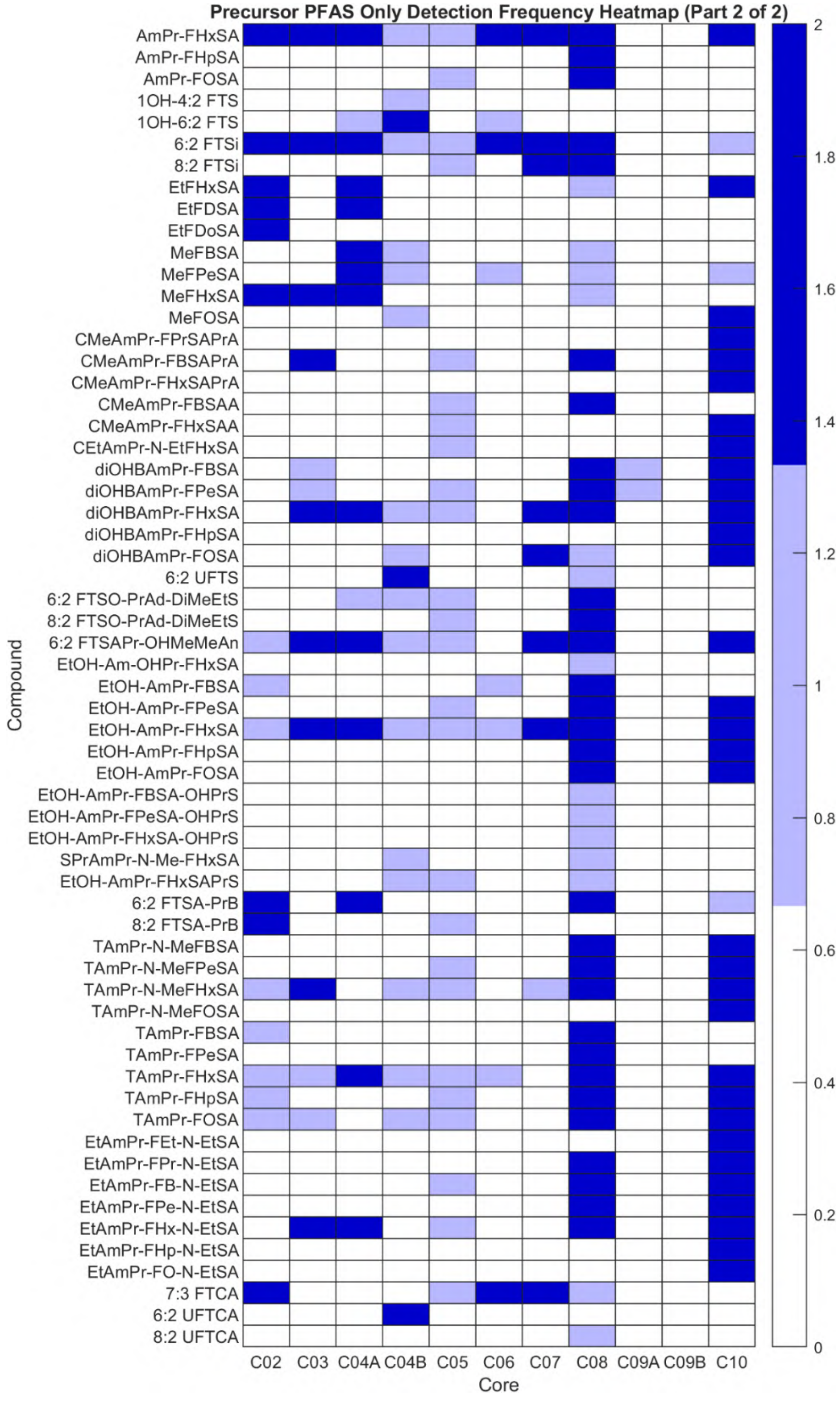
Precursor PFAS Only Detection Frequency Heatmap (Part 2 of 2)
AmPr-FHxSA
AmPr-FHpSA
AmPr-FOSA
1OH-4:2 FTS
1OH-6:2 FTS
6:2 FTSi
8:2 FTSi
EtFHxSA
EtFDSA
EtFDoSA
MeFBSA
MeFPeSA
MeFHxSA
MeFOSA
CMeAmPr-FPrSAPrA
CMeAmPr-FBSAPrA
CMeAmPr-FHxSAPrA
CMeAmPr-FBSAA
CMeAmPr-FHxSAA
CEtAmPr-N-EtFHxSA
diOHBAmPr-FBSA
diOHBAmPr-FPeSA
diOHBAmPr-FHxSA
diOHBAmPr-FHpSA
diOHBAmPr-FOSA
6:2 UFTS
6:2 FTSO-PrAd-DiMeEtS
8:2 FTSO-PrAd-DiMeEtS
6:2 FTSAPr-OHMeMeAn
EtOH-Am-OHPr-FHxSA
EtOH-AmPr-FBSA
EtOH-AmPr-FPeSA
EtOH-AmPr-FHxSA
EtOH-AmPr-FHpSA
EtOH-AmPr-FOSA
EtOH-AmPr-FBSA-OHPrS
EtOH-AmPr-FPeSA-OHPrS
EtOH-AmPr-FHxSA-OHPrS
SPrAmPr-N-Me-FHxSA
EtOH-AmPr-FHxSAPrS
6:2 FTSA-PrB
8:2 FTSA-PrB
TAmPr-N-MeFBSA
TAmPr-N-MeFPeSA
TAmPr-N-MeFHxSA
TAmPr-N-MeFOSA
TAmPr-FBSA
TAmPr-FPeSA
TAmPr-FHxSA
TAmPr-FHpSA
TAmPr-FOSA
EtAmPr-FEt-N-EtSA
EtAmPr-FPr-N-EtSA
EtAmPr-FB-N-EtSA
EtAmPr-FPe-N-EtSA
EtAmPr-FHx-N-EtSA
EtAmPr-FHp-N-EtSA
EtAmPr-FO-N-EtSA
7:3 FTCA
6:2 UFTCA
8:2 UFTCA
Compound
C02
C03
C04A
C04B
C05
C06
C07
C08
C09A
C09B
C10
Core
2
1.8
1.6
1.4
1.2
1
0.8
0.6
0.4
0.2
0

Figure S3. Vertical distribution profile of **SPr-FPrSA** across the twelve studied cores

Note that the concentration is shown on a log-base-10 scale. For any given plot and compound, open markers with dashed-line connectors represent sampled depth intervals where the compounds' concentration was below the reporting limit – the location of the open marker along the x-axis is representative of those reporting limits.

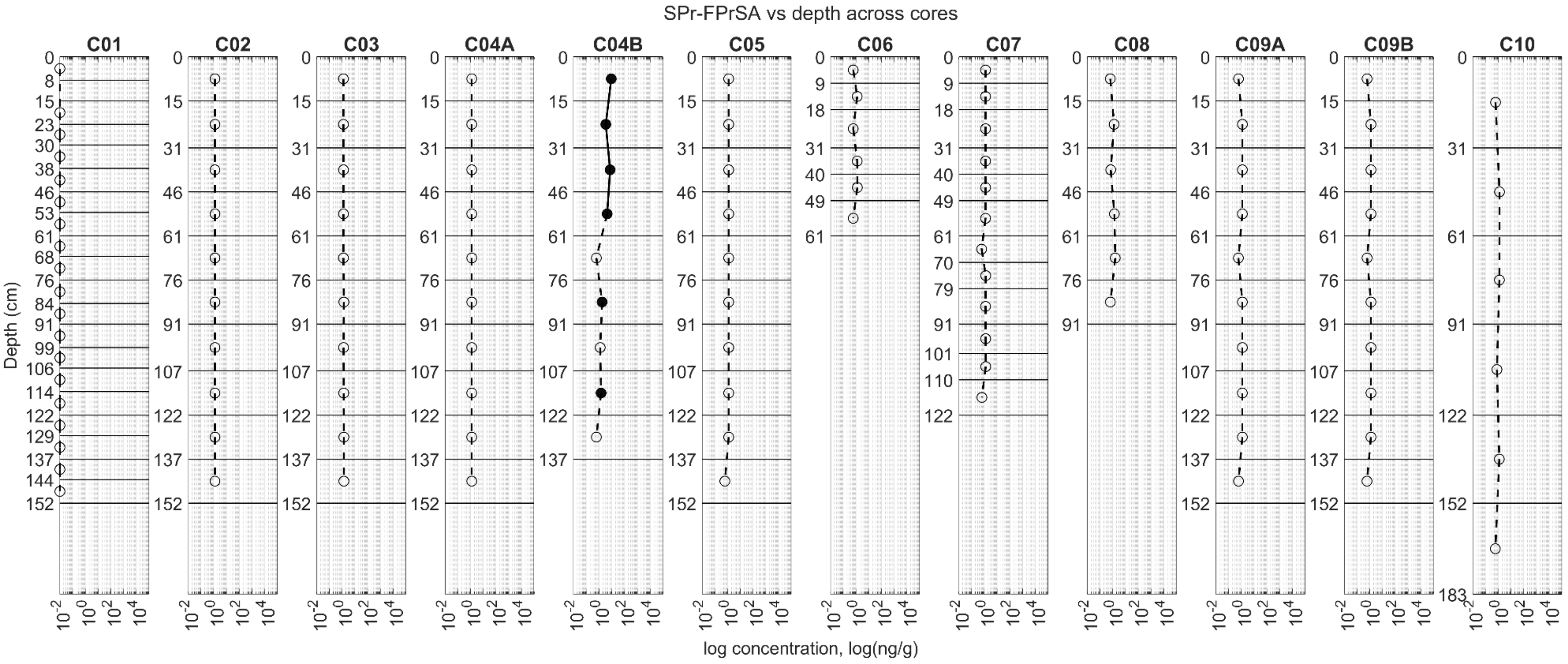

Figure S4. Vertical distribution profile of **SPr-FBSA** across the twelve studied cores

Note that the concentration is shown on a log-base-10 scale. For any given plot and compound, open markers with dashed-line connectors represent sampled depth intervals where the compounds' concentration was below the reporting limit – the location of the open marker along the x-axis is representative of those reporting limits.

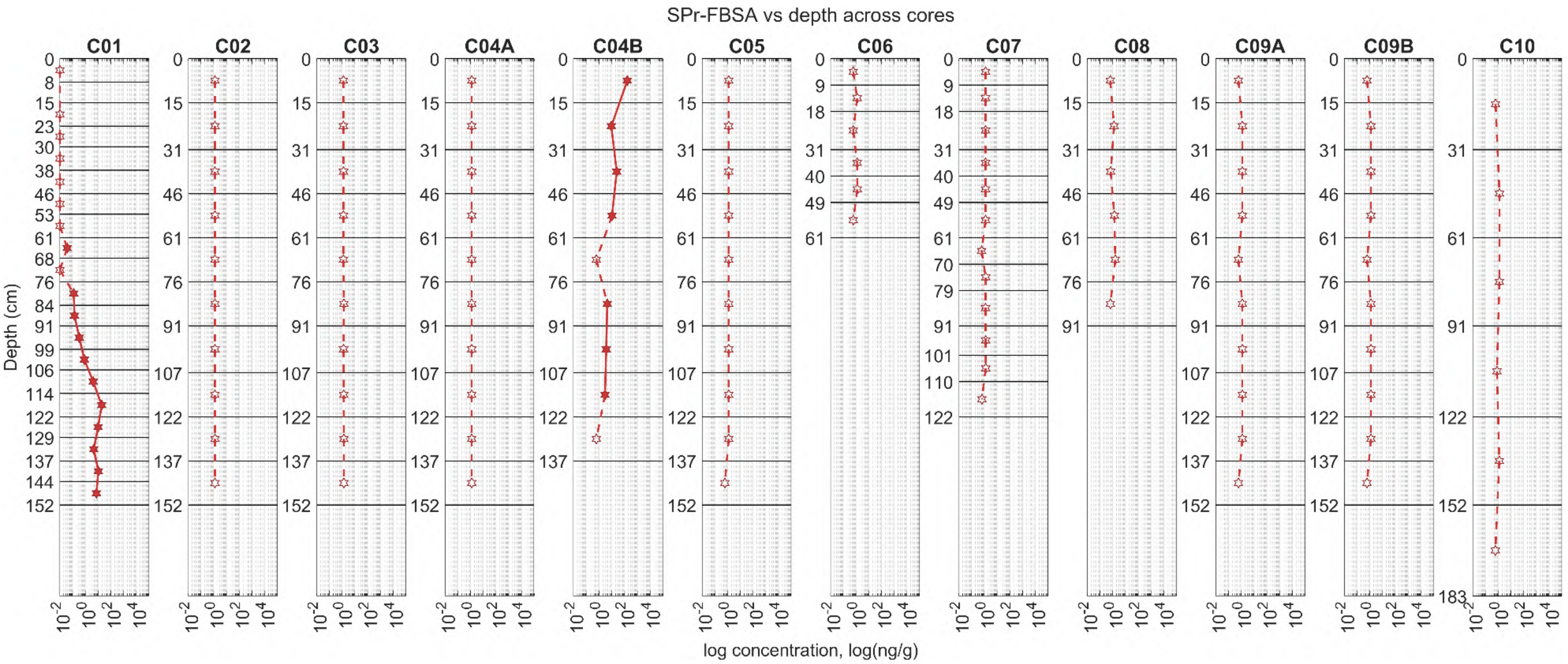

Figure S5. Vertical distribution profile of **SPr-FPeSA** across the twelve studied cores

Note that the concentration is shown on a log-base-10 scale. For any given plot and compound, open markers with dashed-line connectors represent sampled depth intervals where the compounds' concentration was below the reporting limit – the location of the open marker along the x-axis is representative of those reporting limits.

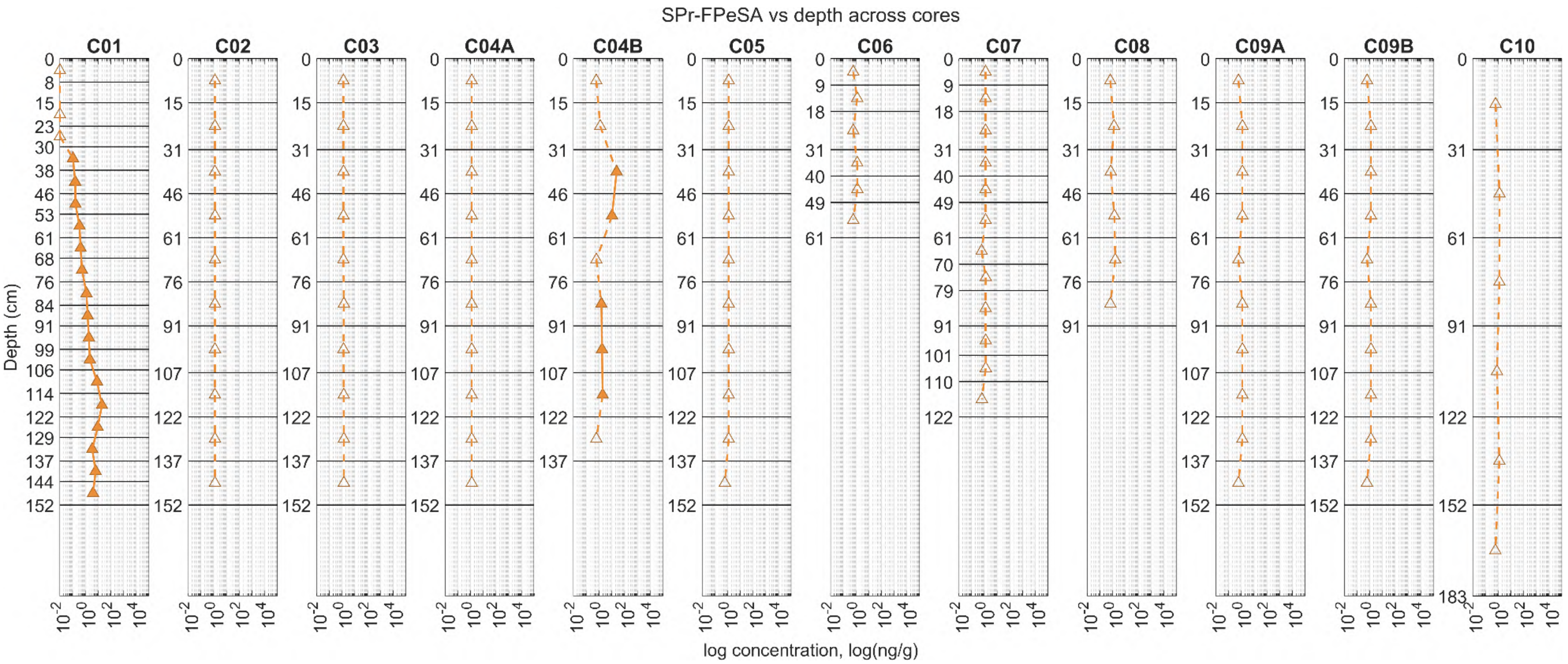

Figure S6. Vertical distribution profile of **SPr-FHxSA** across the twelve studied cores

Note that the concentration is shown on a log-base-10 scale. For any given plot and compound, open markers with dashed-line connectors represent sampled depth intervals where the compounds' concentration was below the reporting limit – the location of the open marker along the x-axis is representative of those reporting limits.

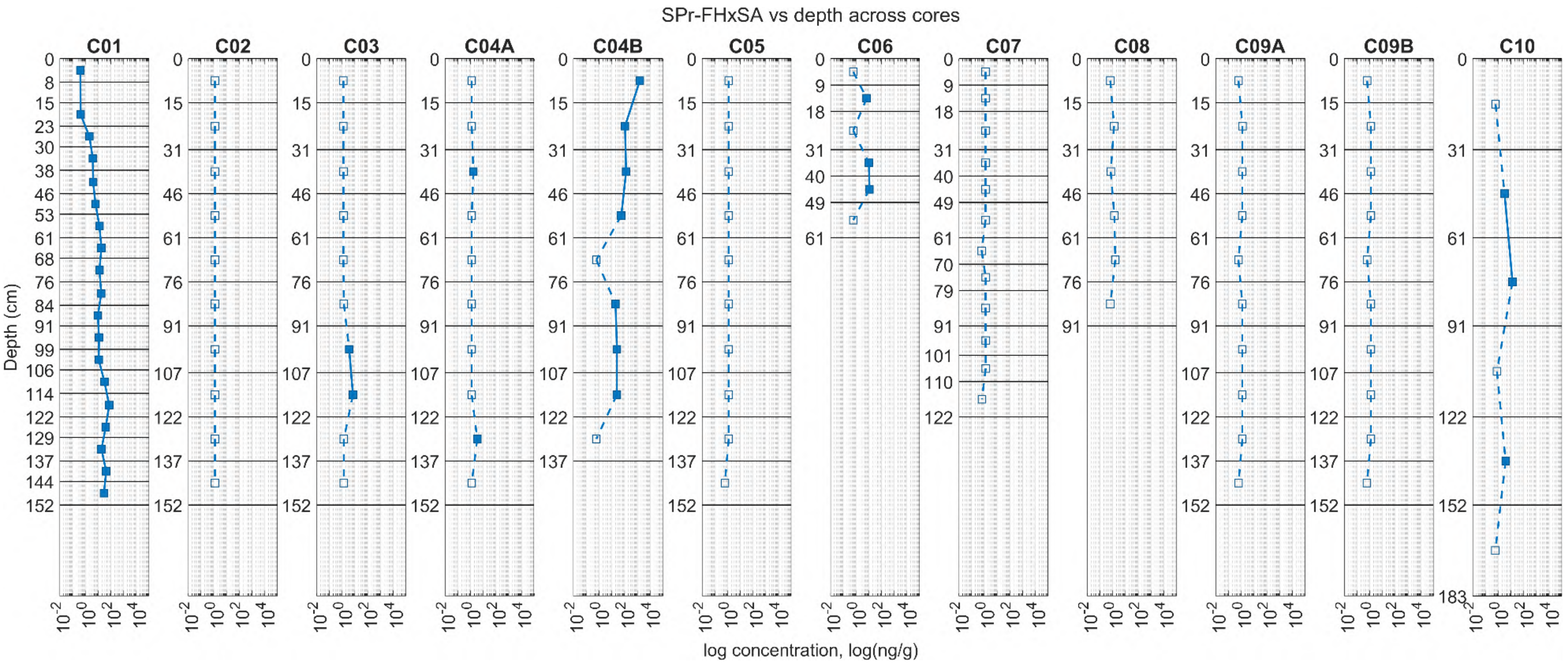

Figure S7. Vertical distribution profile of **CMeAmPr-FBSA** across the twelve studied cores

Note that the concentration is shown on a log-base-10 scale. For any given plot and compound, open markers with dashed-line connectors represent sampled depth intervals where the compounds' concentration was below the reporting limit – the location of the open marker along the x-axis is representative of those reporting limits.

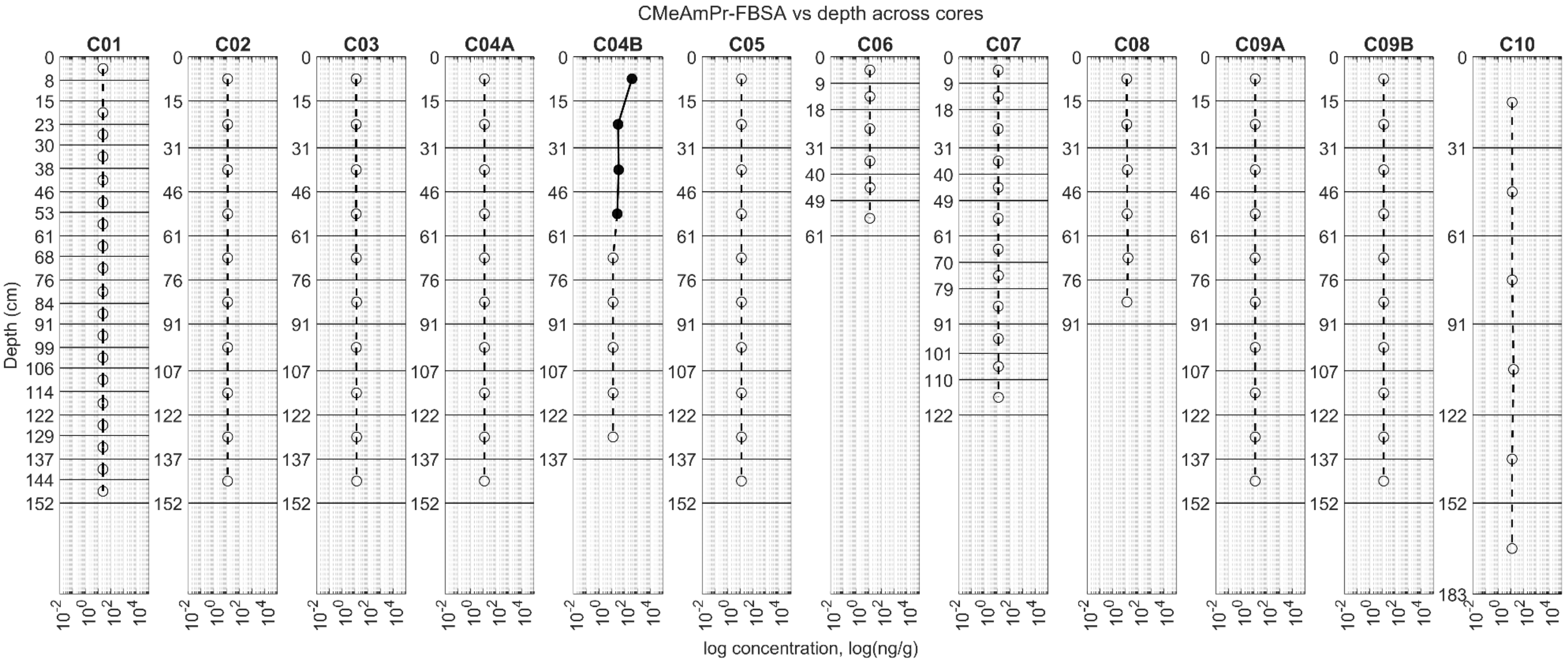

Figure S8. Vertical distribution profile of **CMeAmPr-FHxSA** across the twelve studied cores

Note that the concentration is shown on a log-base-10 scale. For any given plot and compound, open markers with dashed-line connectors represent sampled depth intervals where the compounds' concentration was below the reporting limit – the location of the open marker along the x-axis is representative of those reporting limits.

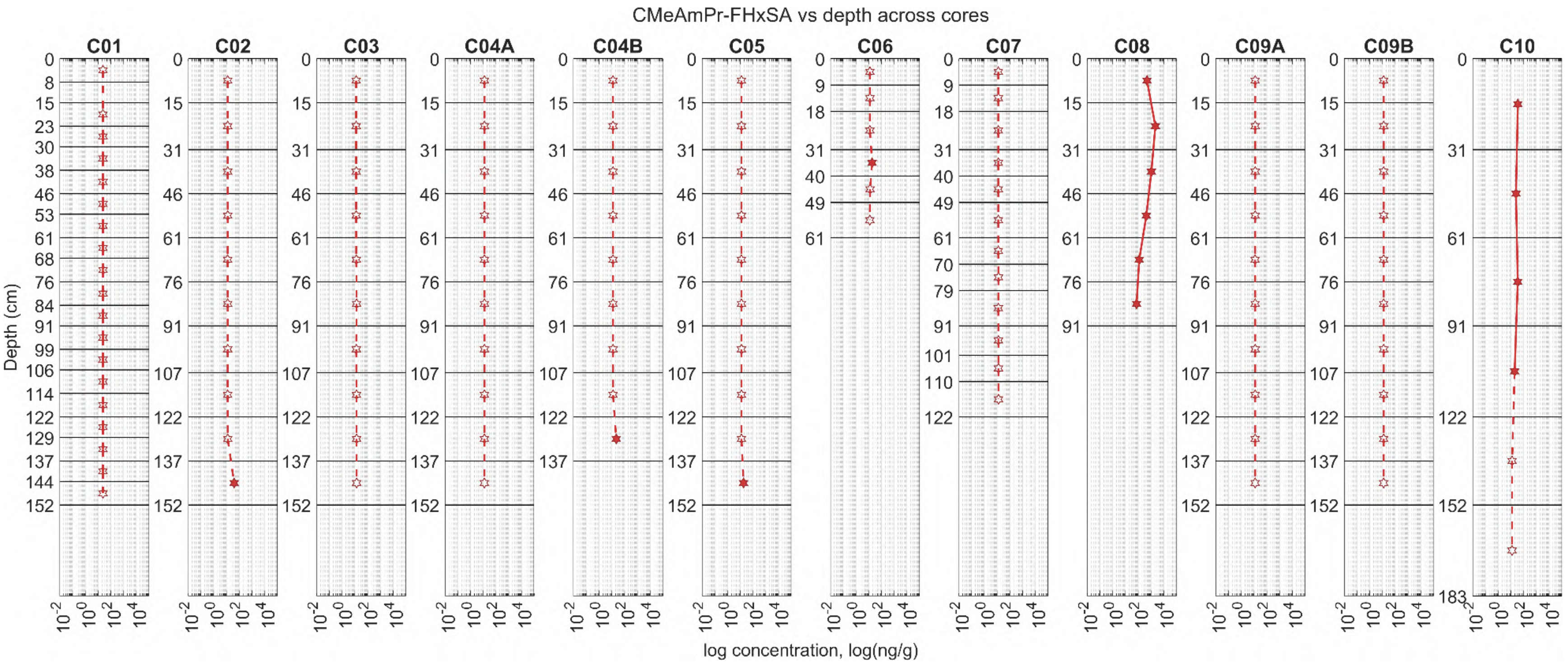

Figure S9. Vertical distribution profile of **SPrAmPr-FPrSA** across the twelve studied cores

Note that the concentration is shown on a log-base-10 scale. For any given plot and compound, open markers with dashed-line connectors represent sampled depth intervals where the compounds' concentration was below the reporting limit – the location of the open marker along the x-axis is representative of those reporting limits.

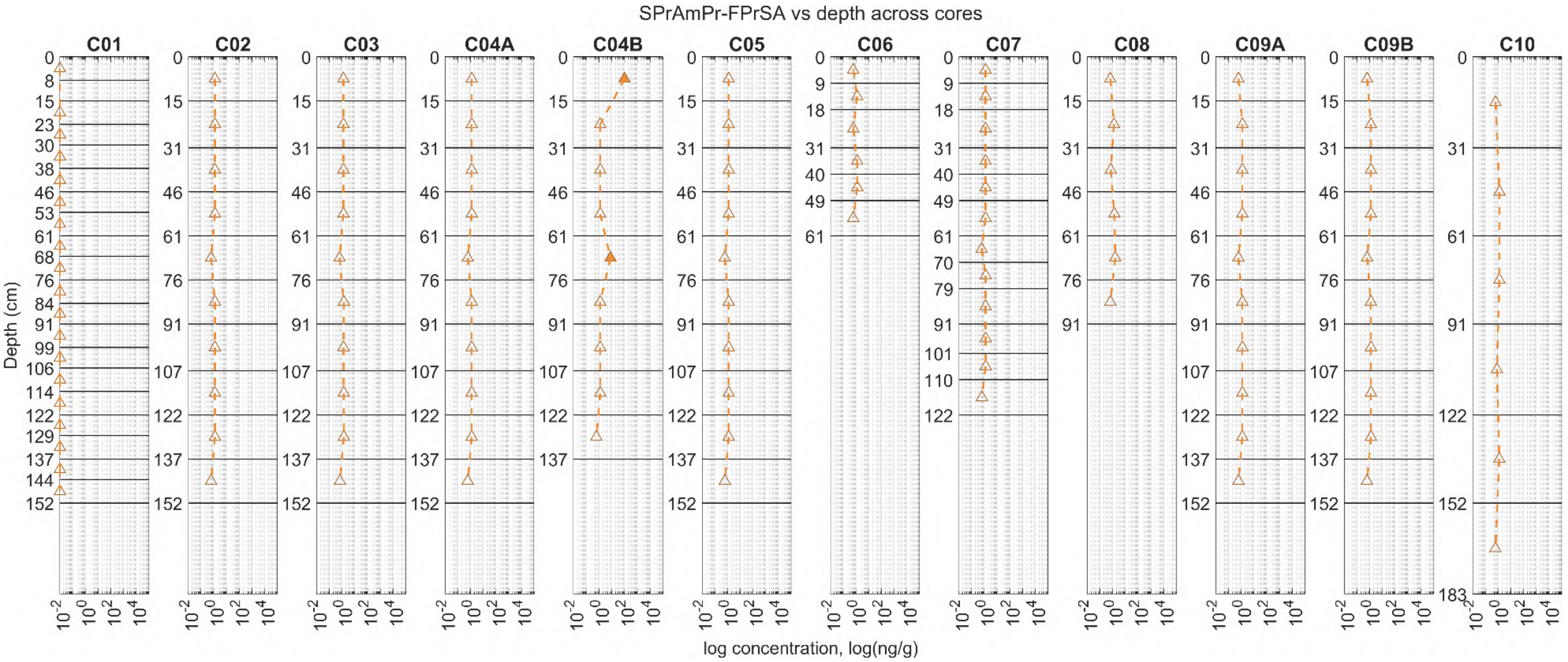

Figure S10. Vertical distribution profile of **SPrAmPr-FBSA** across the twelve studied cores

Note that the concentration is shown on a log-base-10 scale. For any given plot and compound, open markers with dashed-line connectors represent sampled depth intervals where the compounds' concentration was below the reporting limit – the location of the open marker along the x-axis is representative of those reporting limits.

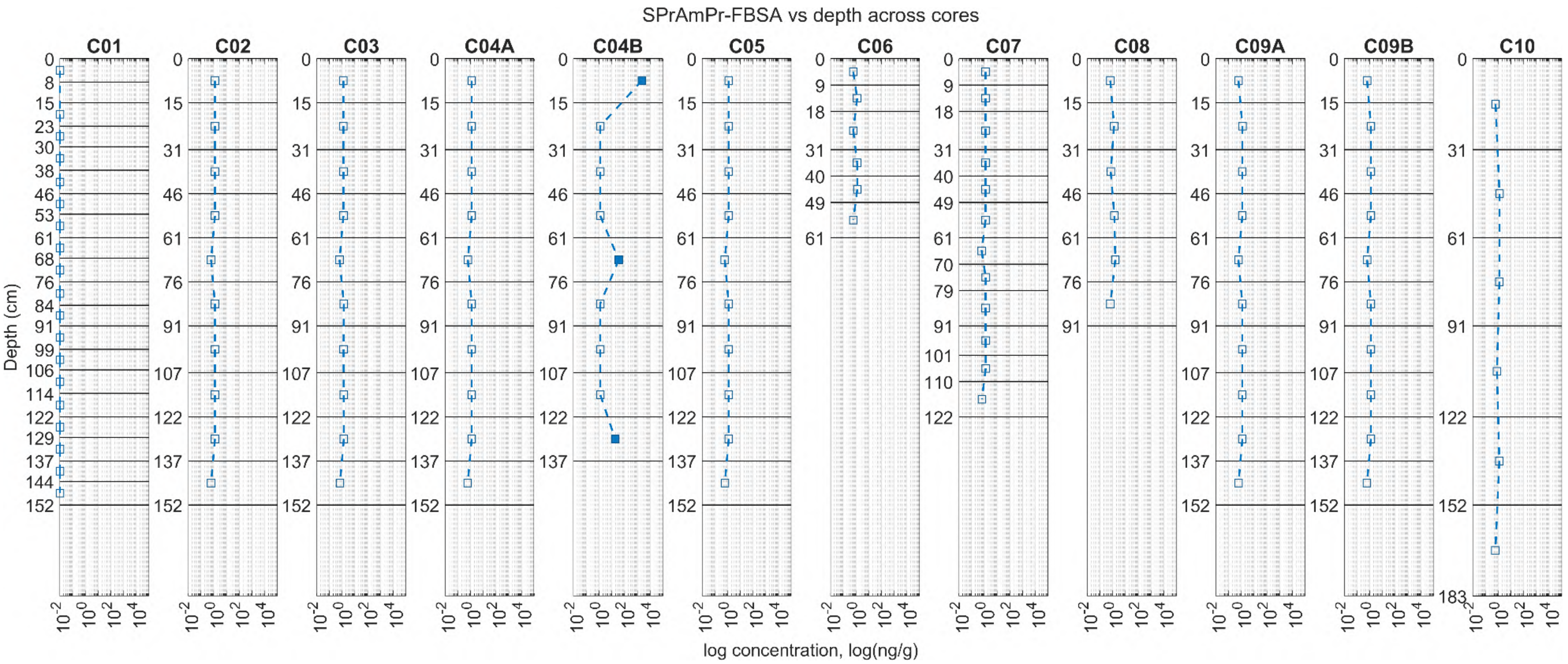

Figure S11. Vertical distribution profile of **SPrAmPr-FPeSA** across the twelve studied cores

Note that the concentration is shown on a log-base-10 scale. For any given plot and compound, open markers with dashed-line connectors represent sampled depth intervals where the compounds' concentration was below the reporting limit – the location of the open marker along the x-axis is representative of those reporting limits.

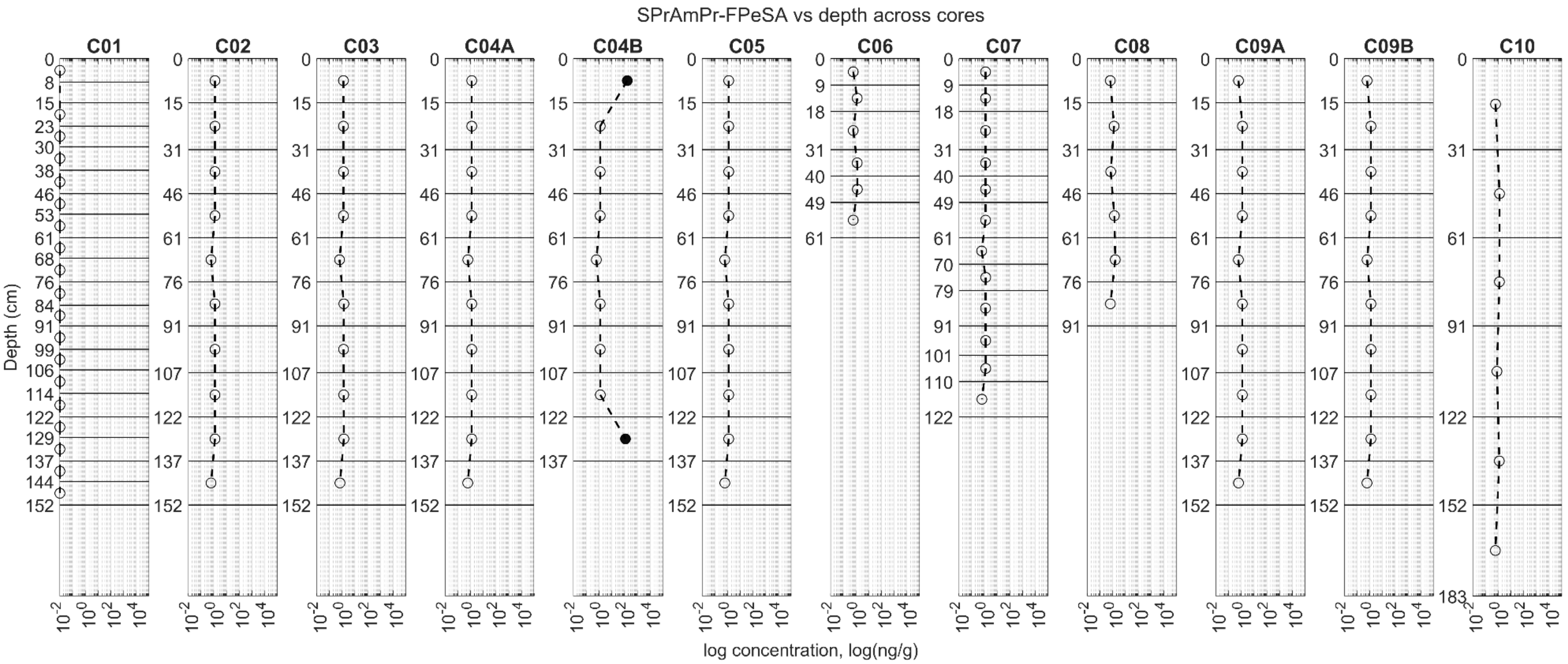

Figure S12. Vertical distribution profile of **SPrAmPr-FHxSA** across the twelve studied cores

Note that the concentration is shown on a log-base-10 scale. For any given plot and compound, open markers with dashed-line connectors represent sampled depth intervals where the compounds' concentration was below the reporting limit – the location of the open marker along the x-axis is representative of those reporting limits.

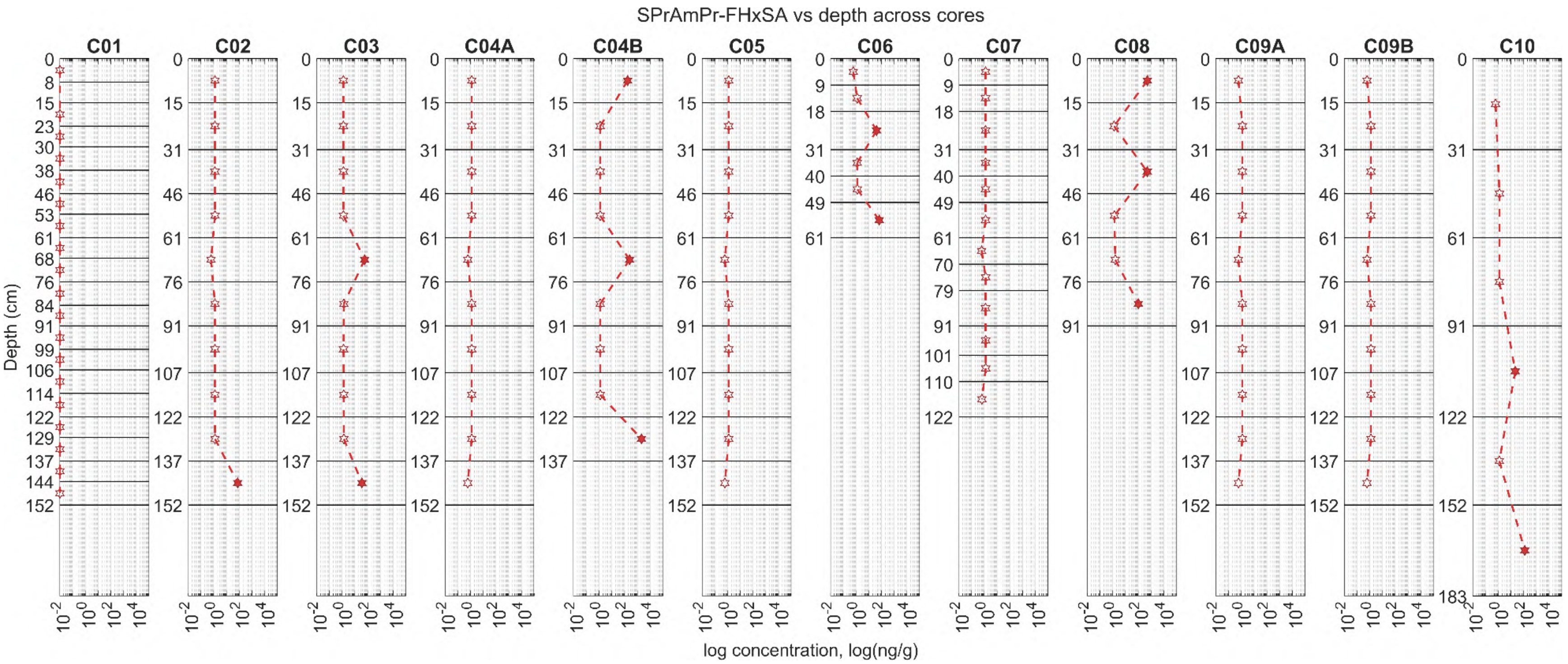

Figure S13. Vertical distribution profile of **F5S-PFOS** across the twelve studied cores

Note that the concentration is shown on a log-base-10 scale. For any given plot and compound, open markers with dashed-line connectors represent sampled depth intervals where the compounds' concentration was below the reporting limit – the location of the open marker along the x-axis is representative of those reporting limits.

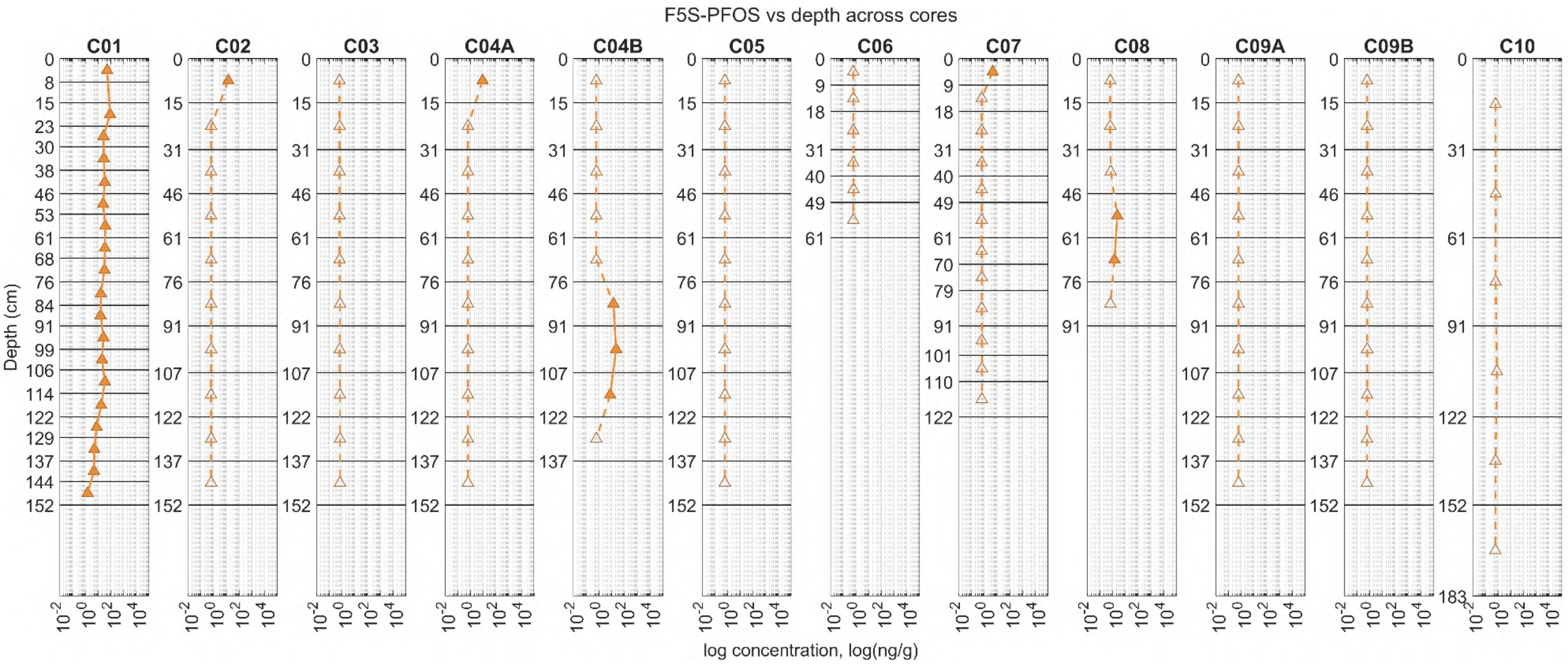

Figure S14. Vertical distribution profile of **H-UPFOS** across the twelve studied cores

Note that the concentration is shown on a log-base-10 scale. For any given plot and compound, open markers with dashed-line connectors represent sampled depth intervals where the compounds' concentration was below the reporting limit – the location of the open marker along the x-axis is representative of those reporting limits.

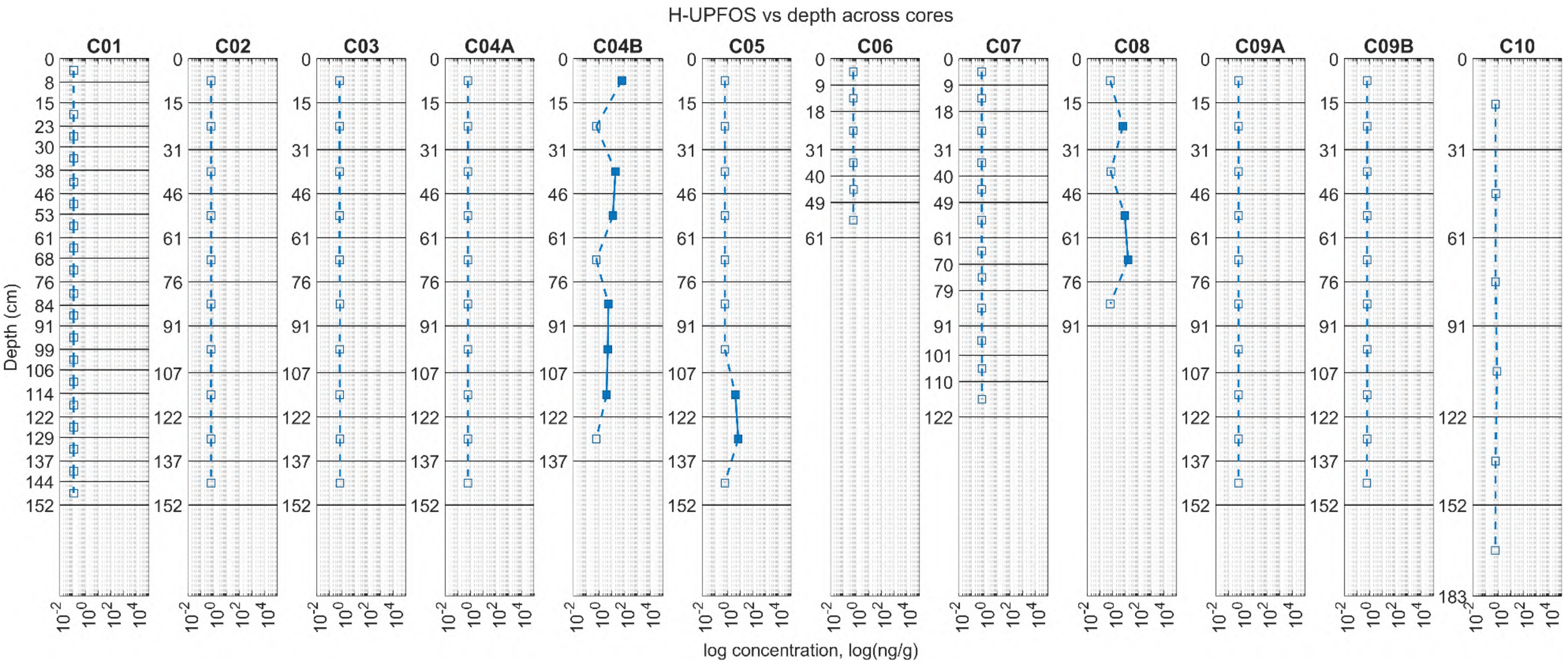

Figure S15. Vertical distribution profile of **H-PFPeS** across the twelve studied cores

Note that the concentration is shown on a log-base-10 scale. For any given plot and compound, open markers with dashed-line connectors represent sampled depth intervals where the compounds' concentration was below the reporting limit – the location of the open marker along the x-axis is representative of those reporting limits.

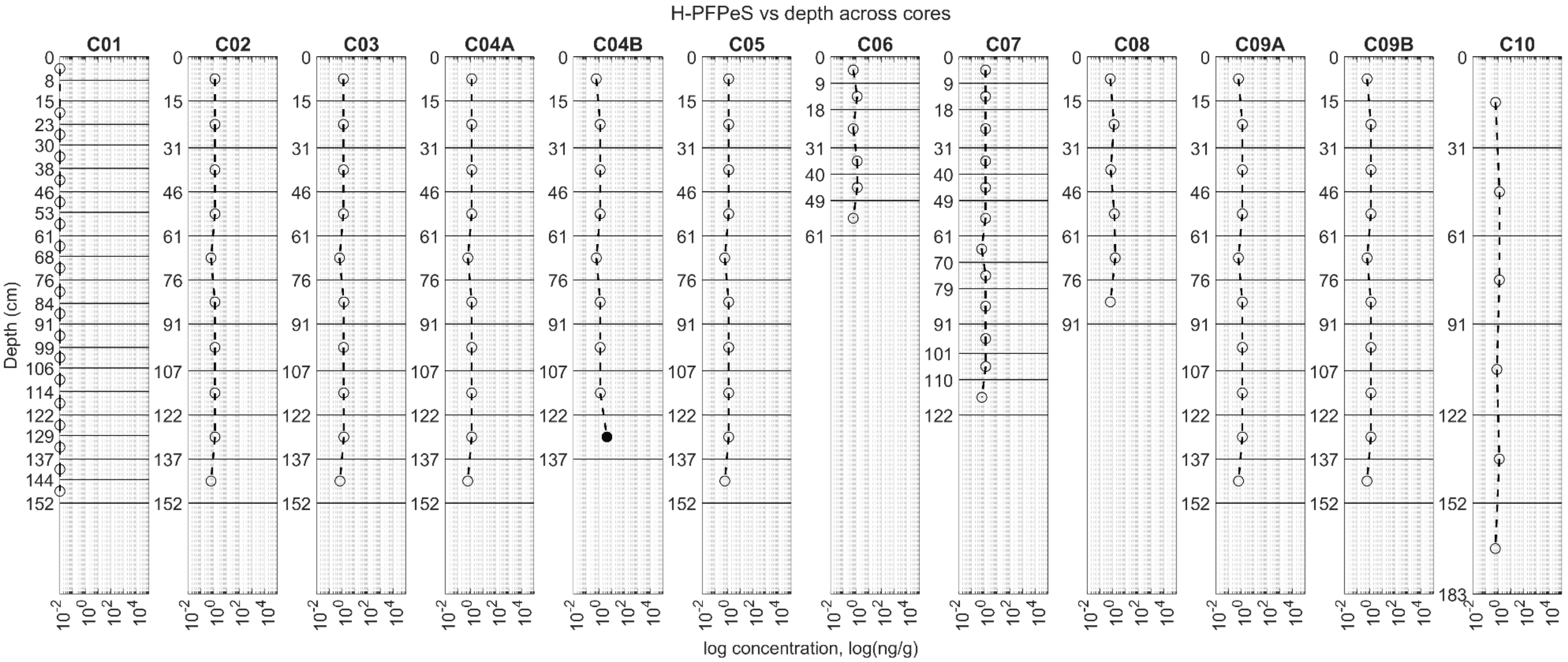

Figure S16. Vertical distribution profile of **H-PFHxS** across the twelve studied cores

Note that the concentration is shown on a log-base-10 scale. For any given plot and compound, open markers with dashed-line connectors represent sampled depth intervals where the compounds' concentration was below the reporting limit – the location of the open marker along the x-axis is representative of those reporting limits.

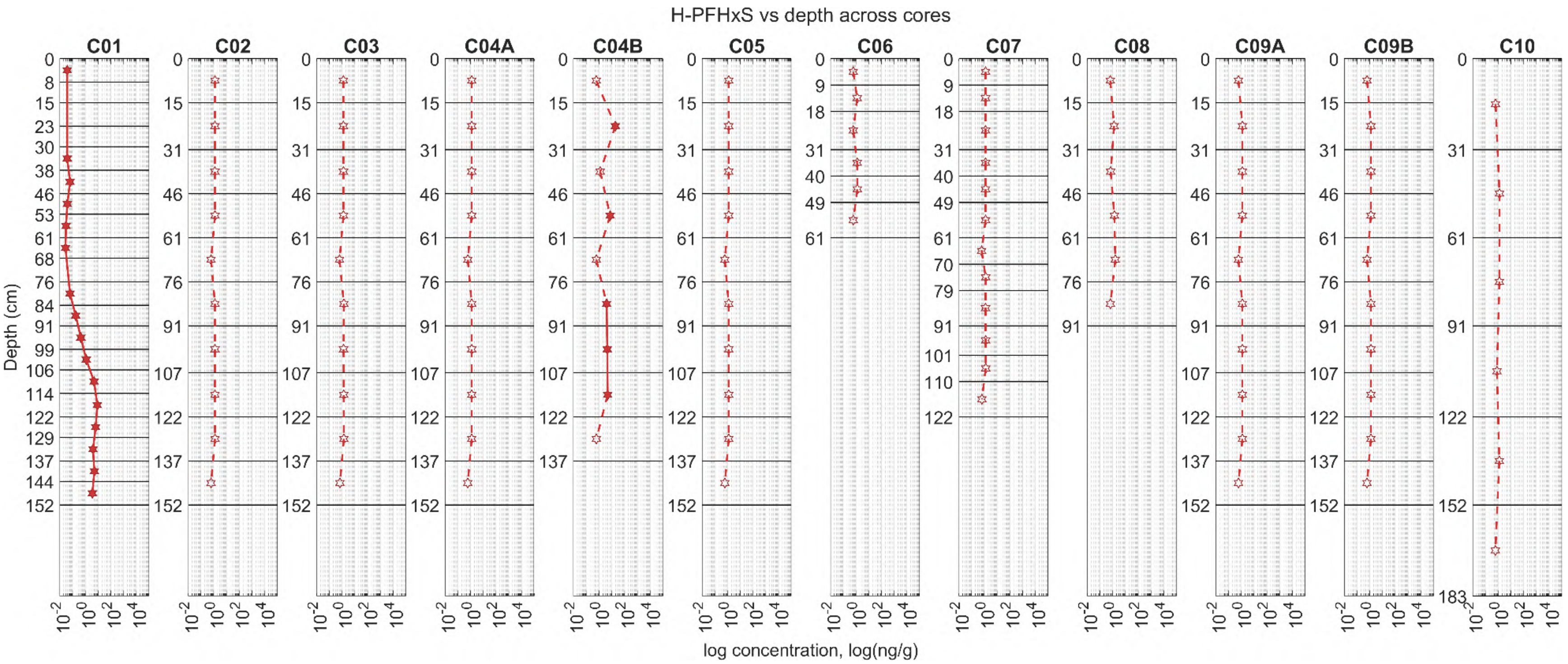

Figure S17. Vertical distribution profile of **H-PFOS** across the twelve studied cores

Note that the concentration is shown on a log-base-10 scale. For any given plot and compound, open markers with dashed-line connectors represent sampled depth intervals where the compounds' concentration was below the reporting limit – the location of the open marker along the x-axis is representative of those reporting limits.

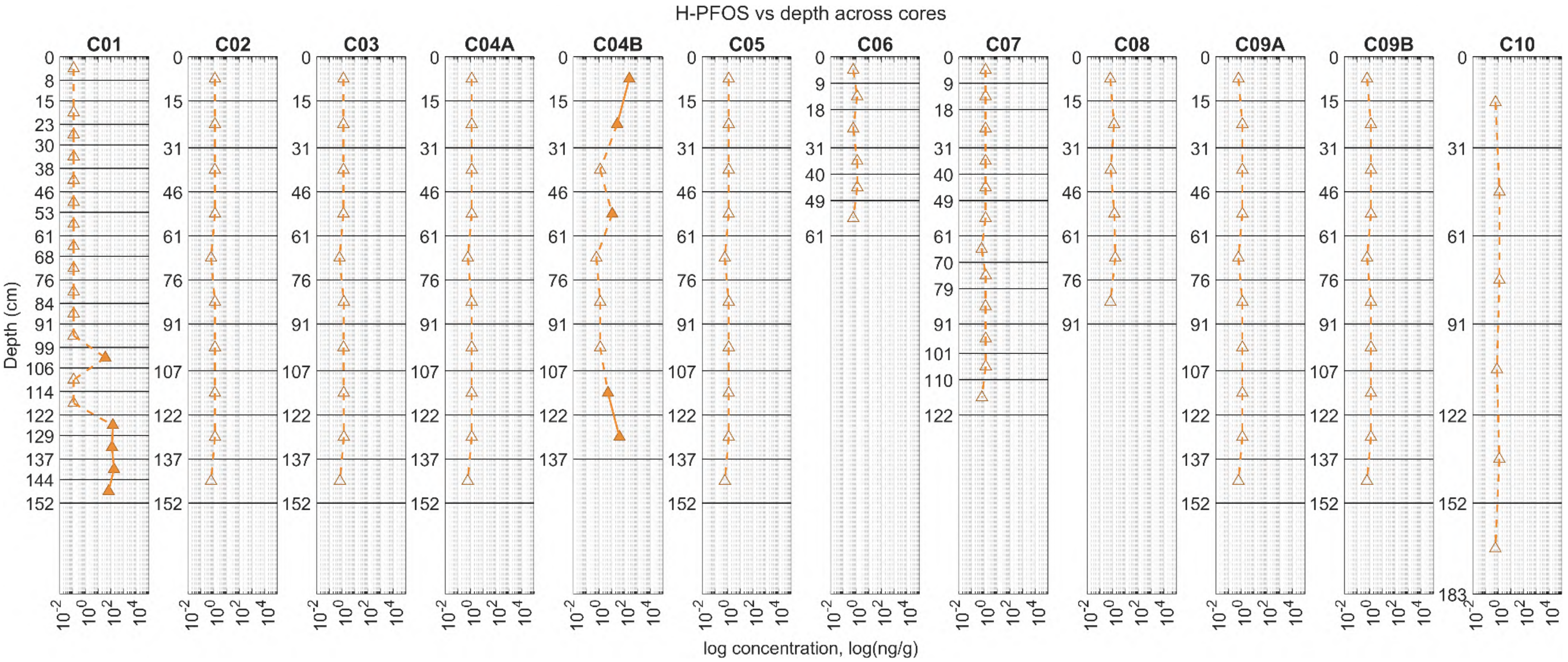

Figure S18. Vertical distribution profile of **S-OHPrAmPr-FBSA-OHPrS** across the twelve studied cores

Note that the concentration is shown on a log-base-10 scale. For any given plot and compound, open markers with dashed-line connectors represent sampled depth intervals where the compounds' concentration was below the reporting limit – the location of the open marker along the x-axis is representative of those reporting limits.

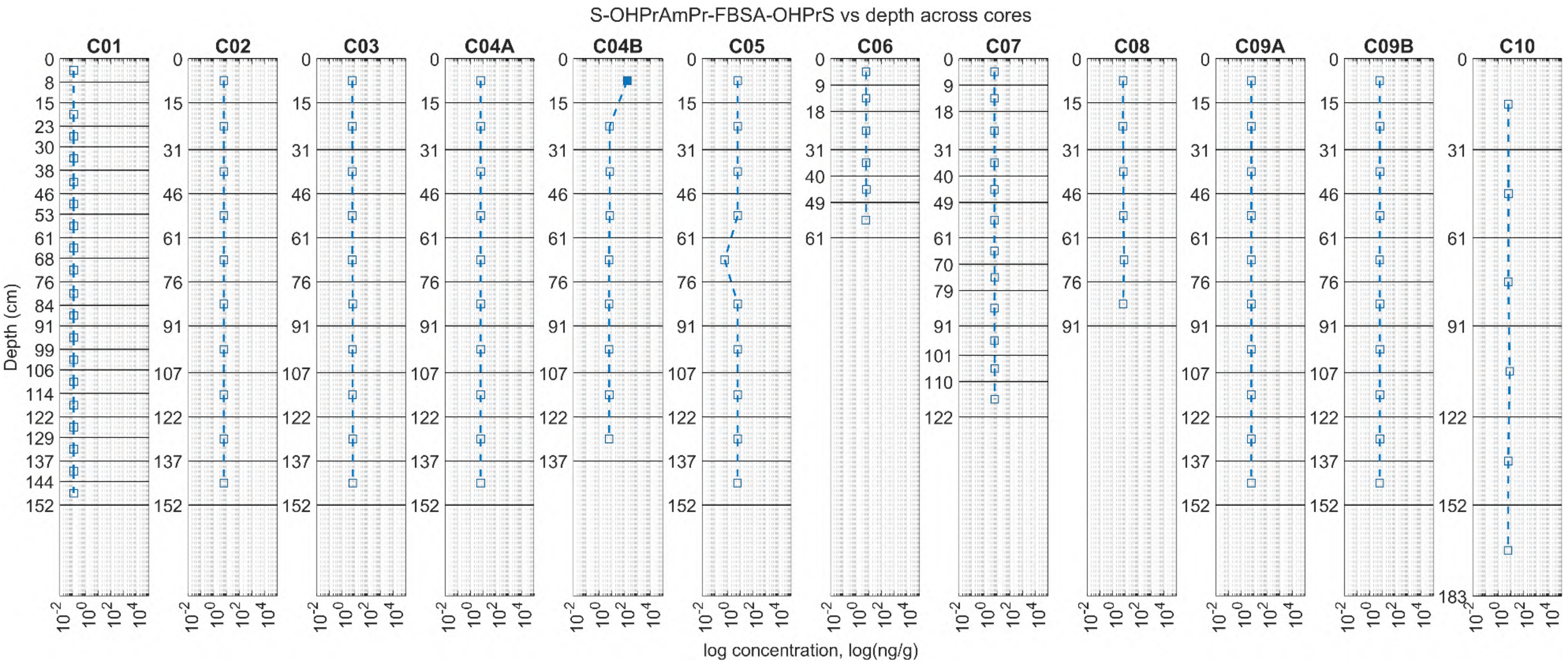

Figure S19. Vertical distribution profile of **S-OHPrAmPr-FHxSA-OHPrS** across the twelve studied cores

Note that the concentration is shown on a log-base-10 scale. For any given plot and compound, open markers with dashed-line connectors represent sampled depth intervals where the compounds' concentration was below the reporting limit – the location of the open marker along the x-axis is representative of those reporting limits.

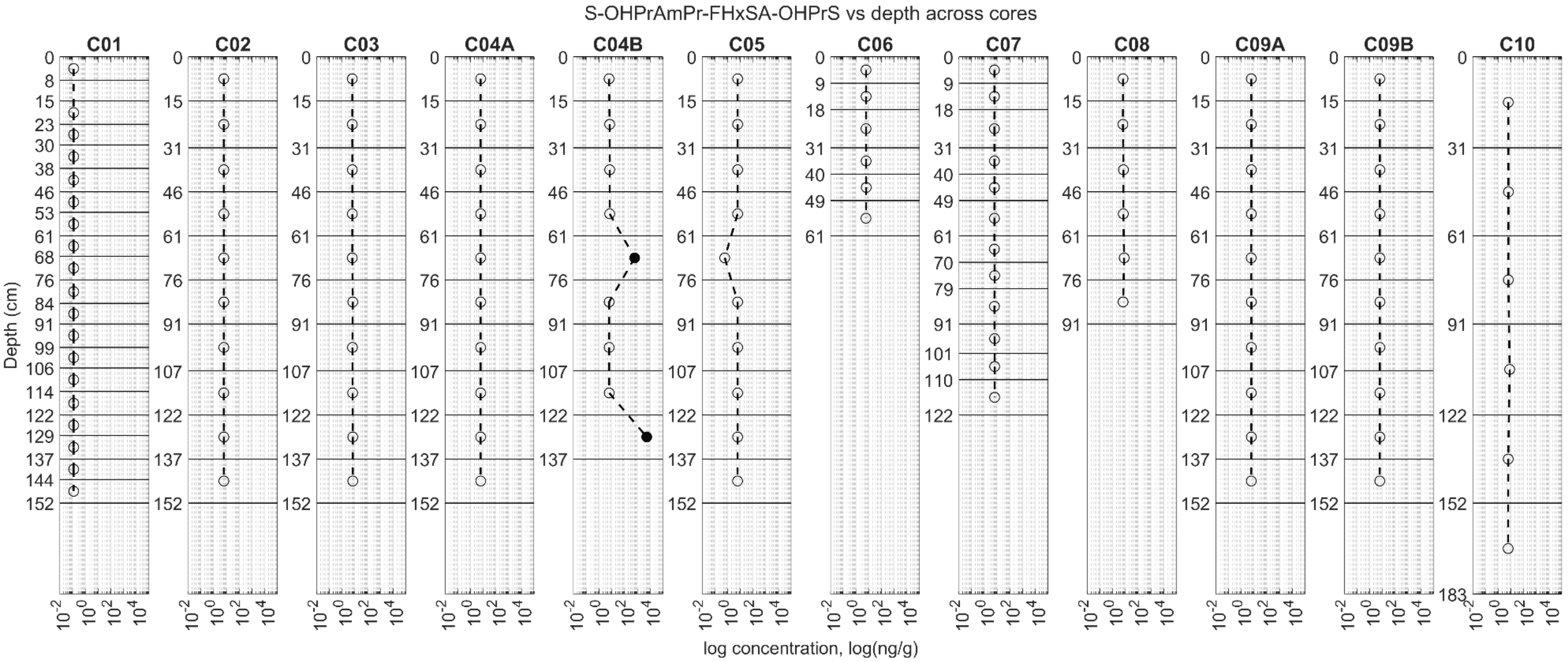

Figure S20. Vertical distribution profile of **S-OHPrAmPr-FBSA** across the twelve studied cores

Note that the concentration is shown on a log-base-10 scale. For any given plot and compound, open markers with dashed-line connectors represent sampled depth intervals where the compounds' concentration was below the reporting limit – the location of the open marker along the x-axis is representative of those reporting limits.

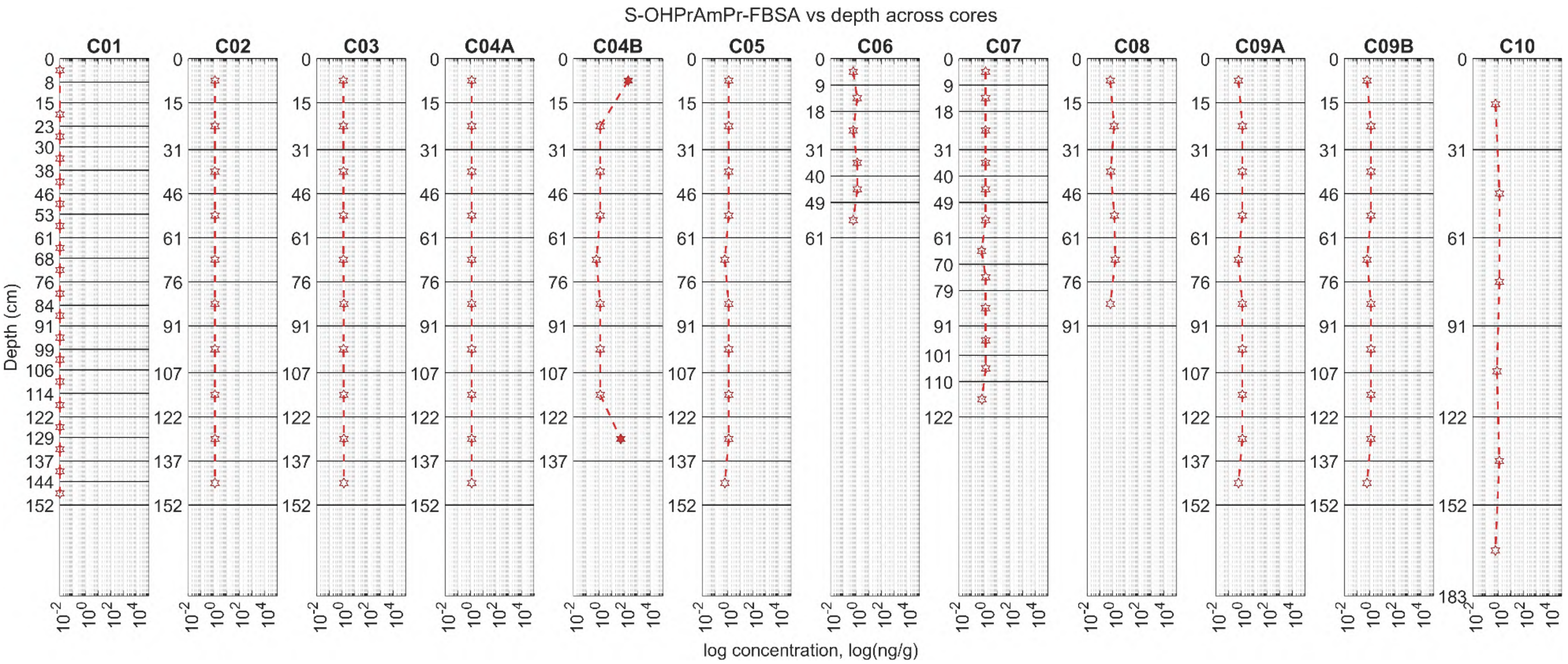

Figure S21. Vertical distribution profile of **S-OHPrAmPr-FPeSA** across the twelve studied cores

Note that the concentration is shown on a log-base-10 scale. For any given plot and compound, open markers with dashed-line connectors represent sampled depth intervals where the compounds' concentration was below the reporting limit – the location of the open marker along the x-axis is representative of those reporting limits.

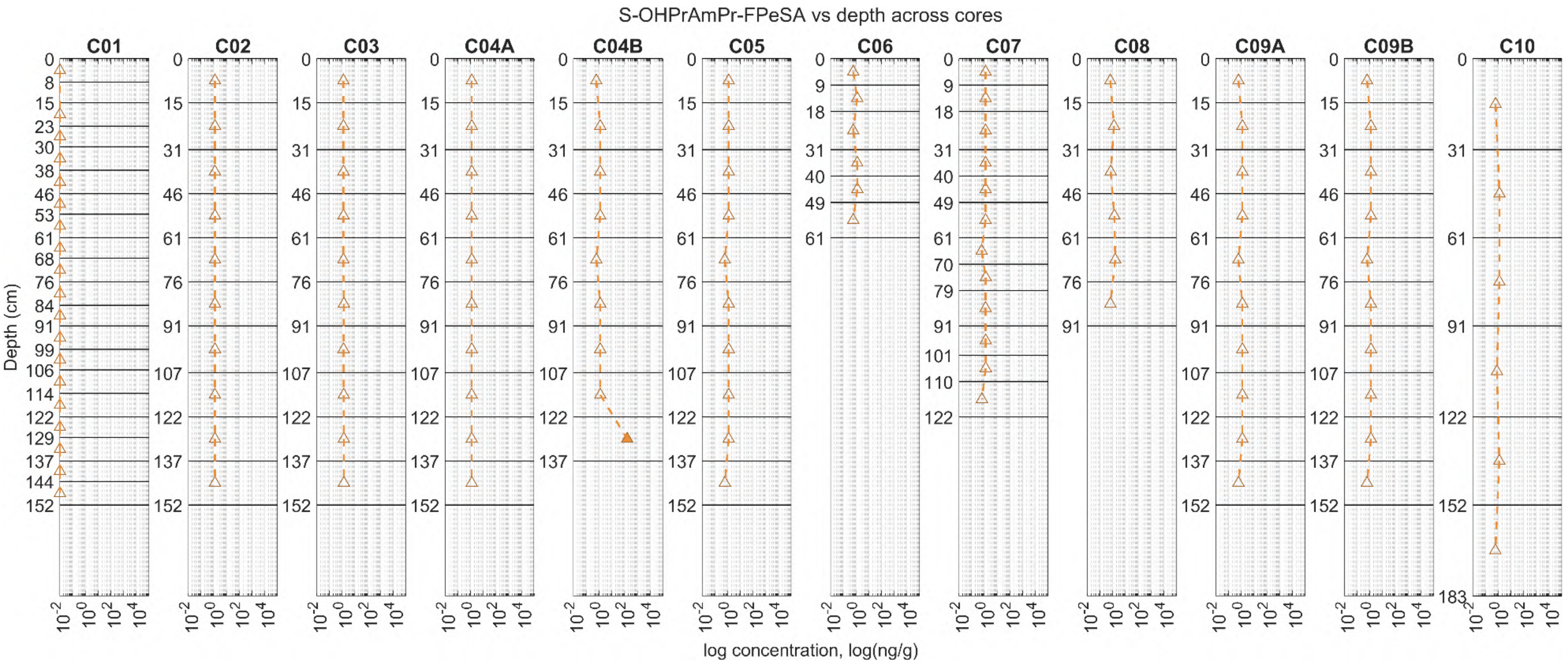

Figure S22. Vertical distribution profile of **S-OHPrAmPr-FHxSA** across the twelve studied cores

Note that the concentration is shown on a log-base-10 scale. For any given plot and compound, open markers with dashed-line connectors represent sampled depth intervals where the compounds' concentration was below the reporting limit – the location of the open marker along the x-axis is representative of those reporting limits.

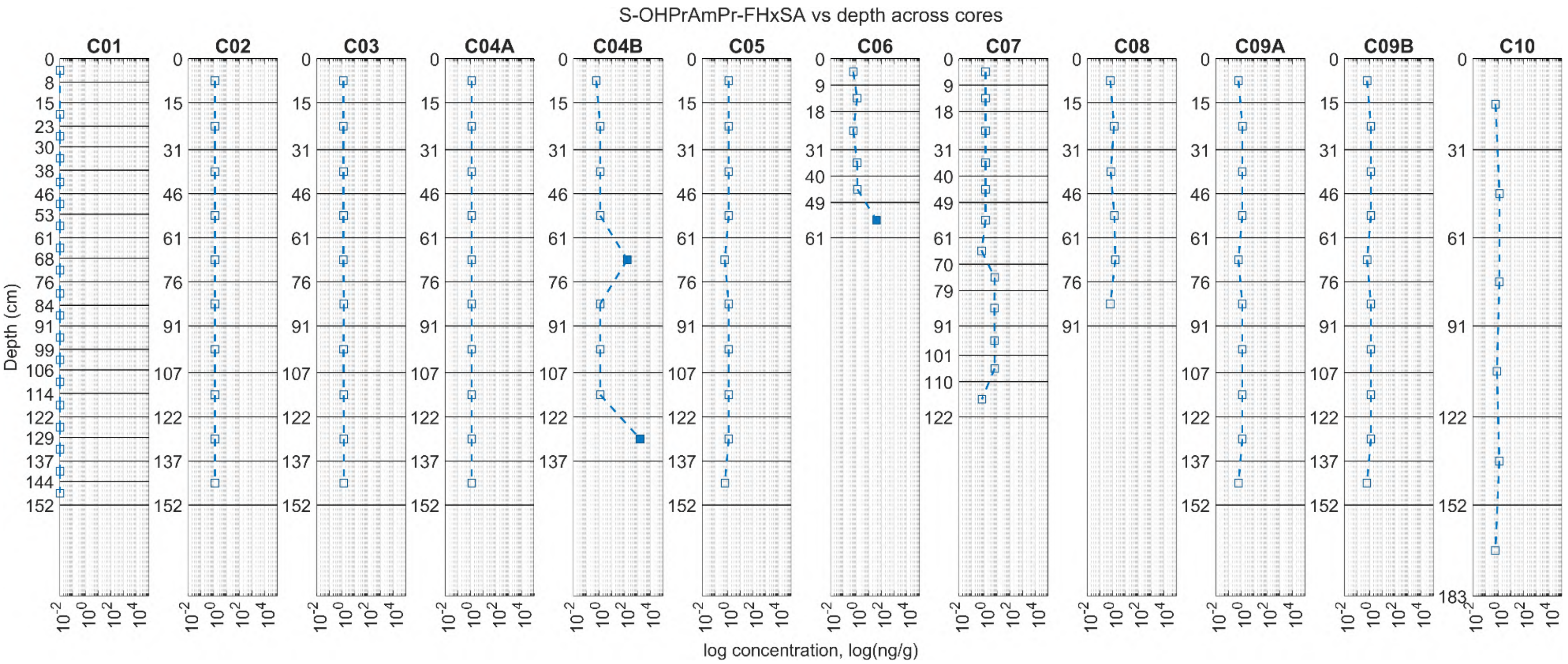

Figure S23. Vertical distribution profile of **SPrAmPr-FPrSAA** across the twelve studied cores

Note that the concentration is shown on a log-base-10 scale. For any given plot and compound, open markers with dashed-line connectors represent sampled depth intervals where the compounds' concentration was below the reporting limit – the location of the open marker along the x-axis is representative of those reporting limits.

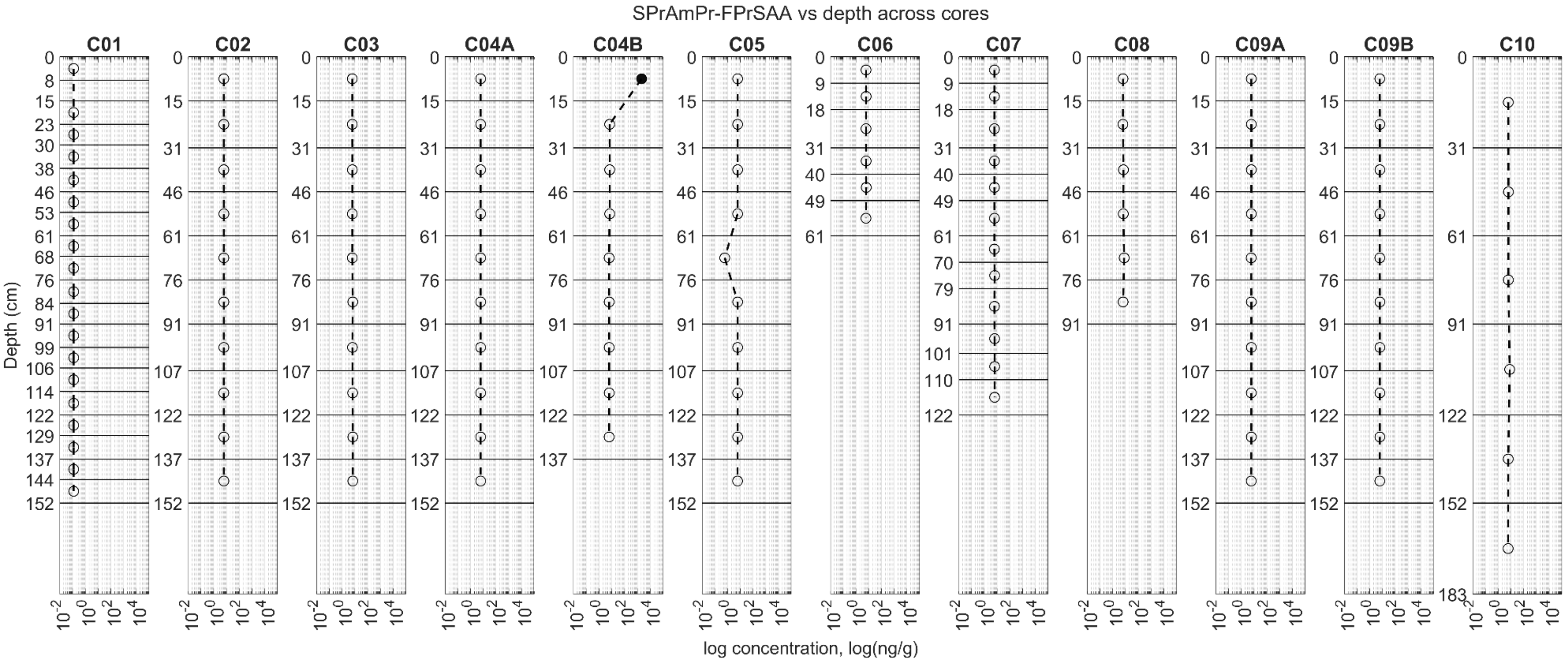

Figure S24. Vertical distribution profile of **SPrAmPr-FBSAA** across the twelve studied cores

Note that the concentration is shown on a log-base-10 scale. For any given plot and compound, open markers with dashed-line connectors represent sampled depth intervals where the compounds' concentration was below the reporting limit – the location of the open marker along the x-axis is representative of those reporting limits.

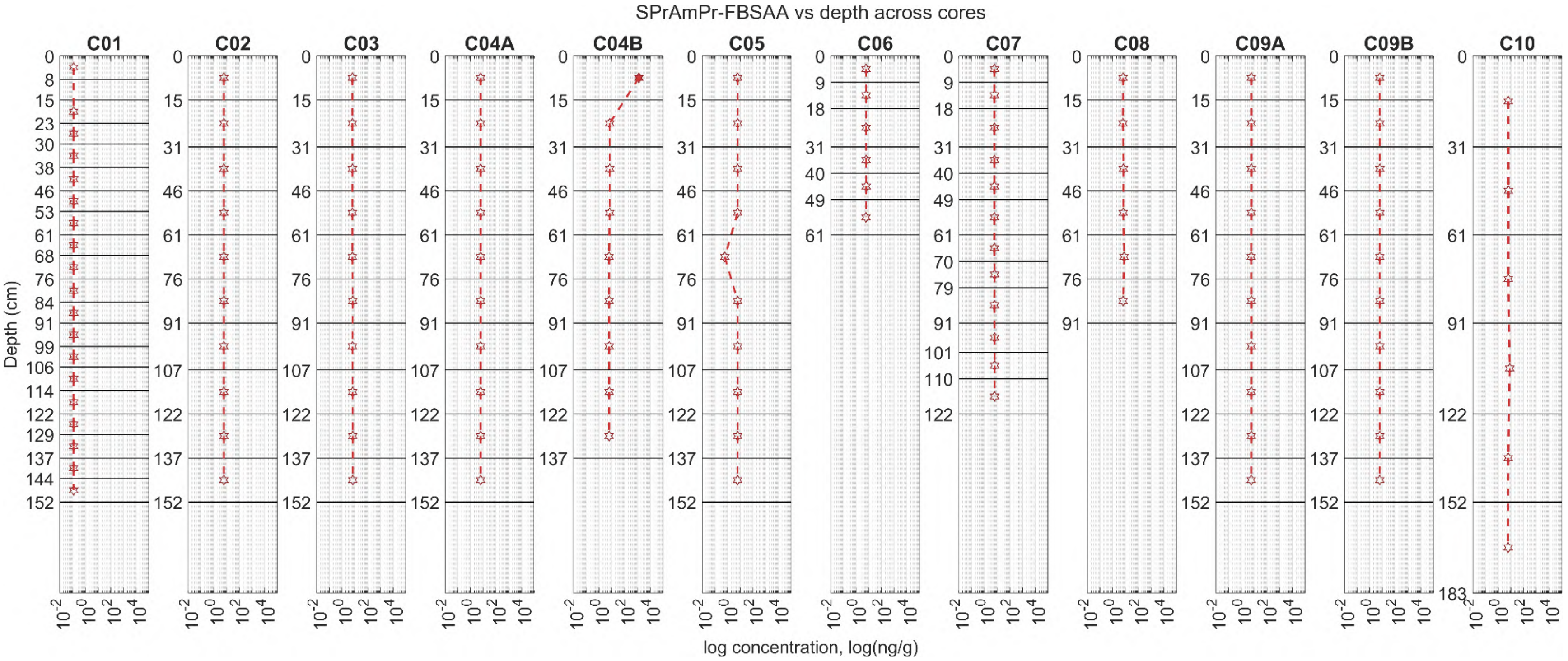

Figure S25. Vertical distribution profile of **8:2 FTSO2PrA** across the twelve studied cores

Note that the concentration is shown on a log-base-10 scale. For any given plot and compound, open markers with dashed-line connectors represent sampled depth intervals where the compounds' concentration was below the reporting limit – the location of the open marker along the x-axis is representative of those reporting limits.

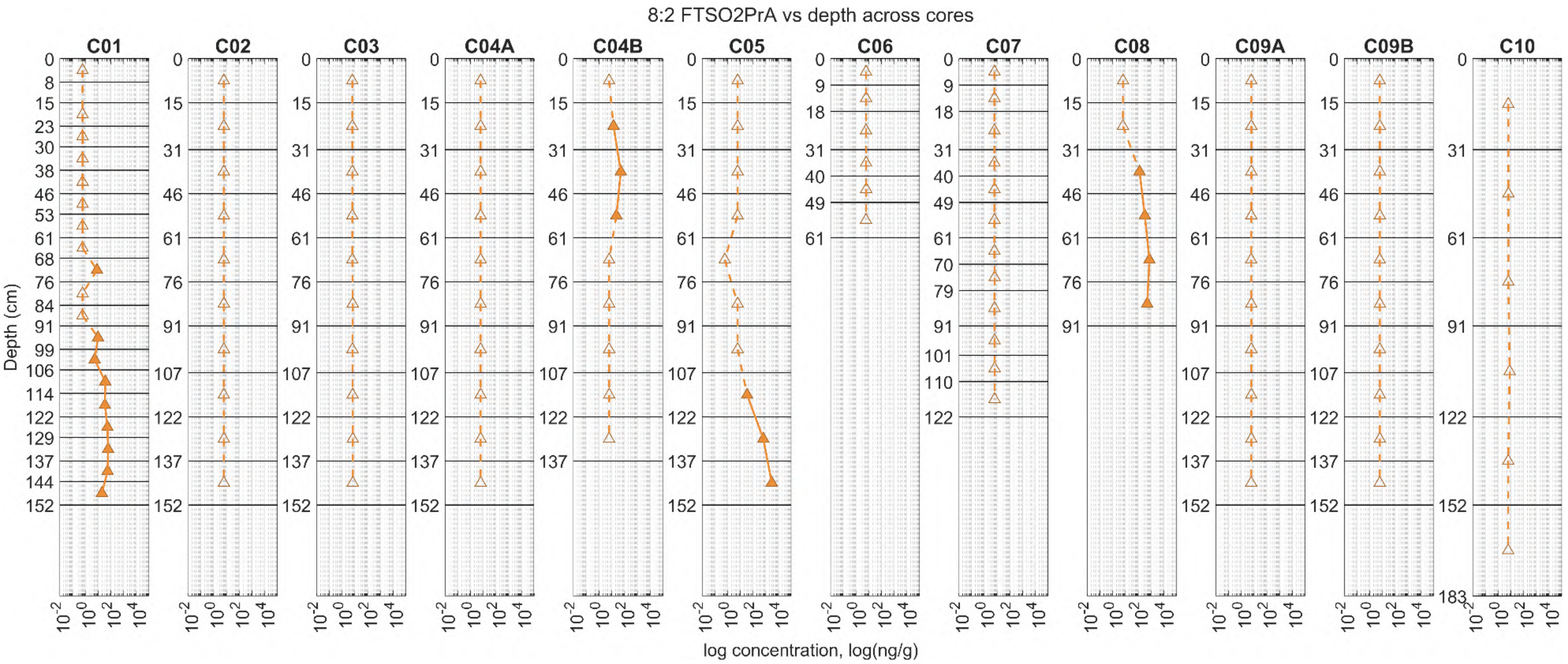

Figure S26. Vertical distribution profile of **SPrAmPr-FPrSAPrS** across the twelve studied cores

Note that the concentration is shown on a log-base-10 scale. For any given plot and compound, open markers with dashed-line connectors represent sampled depth intervals where the compounds' concentration was below the reporting limit – the location of the open marker along the x-axis is representative of those reporting limits.

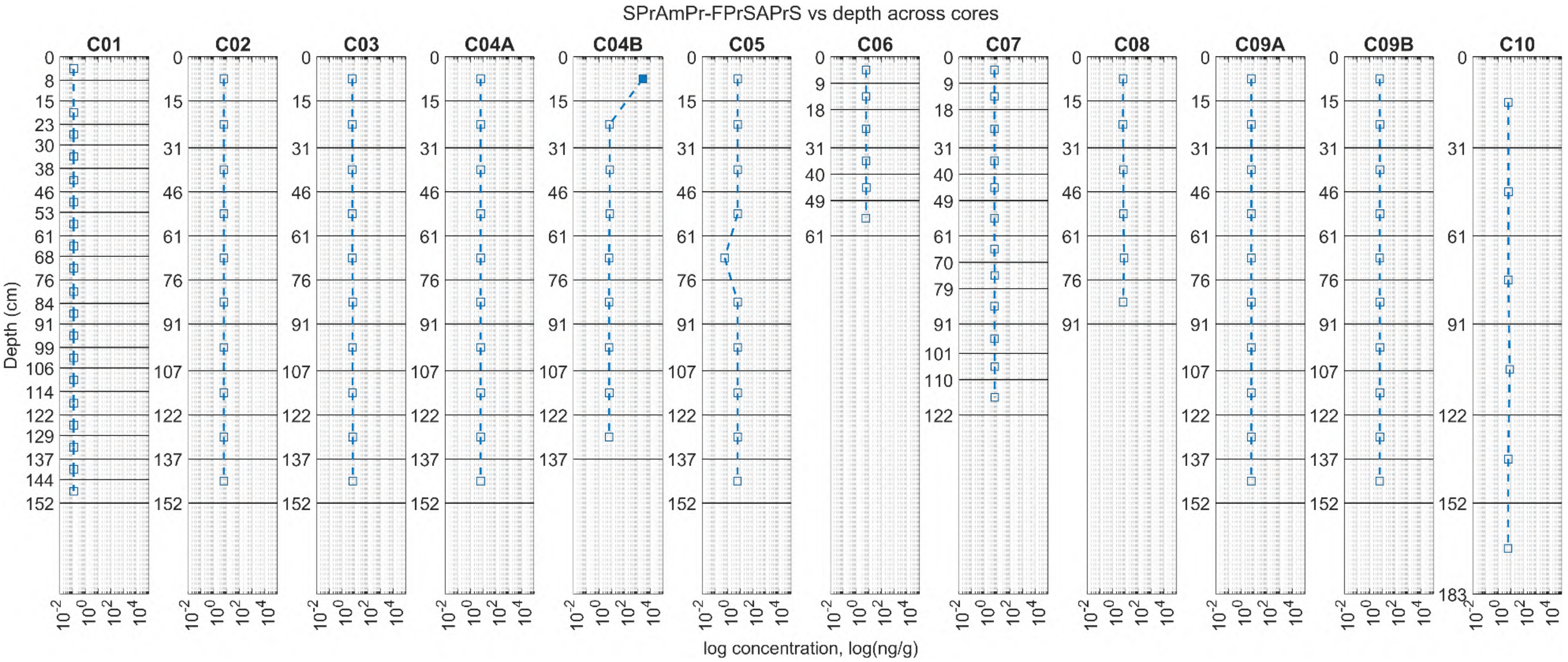

Figure S27. Vertical distribution profile of **SPrAmPr-FBSAPrS** across the twelve studied cores

Note that the concentration is shown on a log-base-10 scale. For any given plot and compound, open markers with dashed-line connectors represent sampled depth intervals where the compounds' concentration was below the reporting limit – the location of the open marker along the x-axis is representative of those reporting limits.

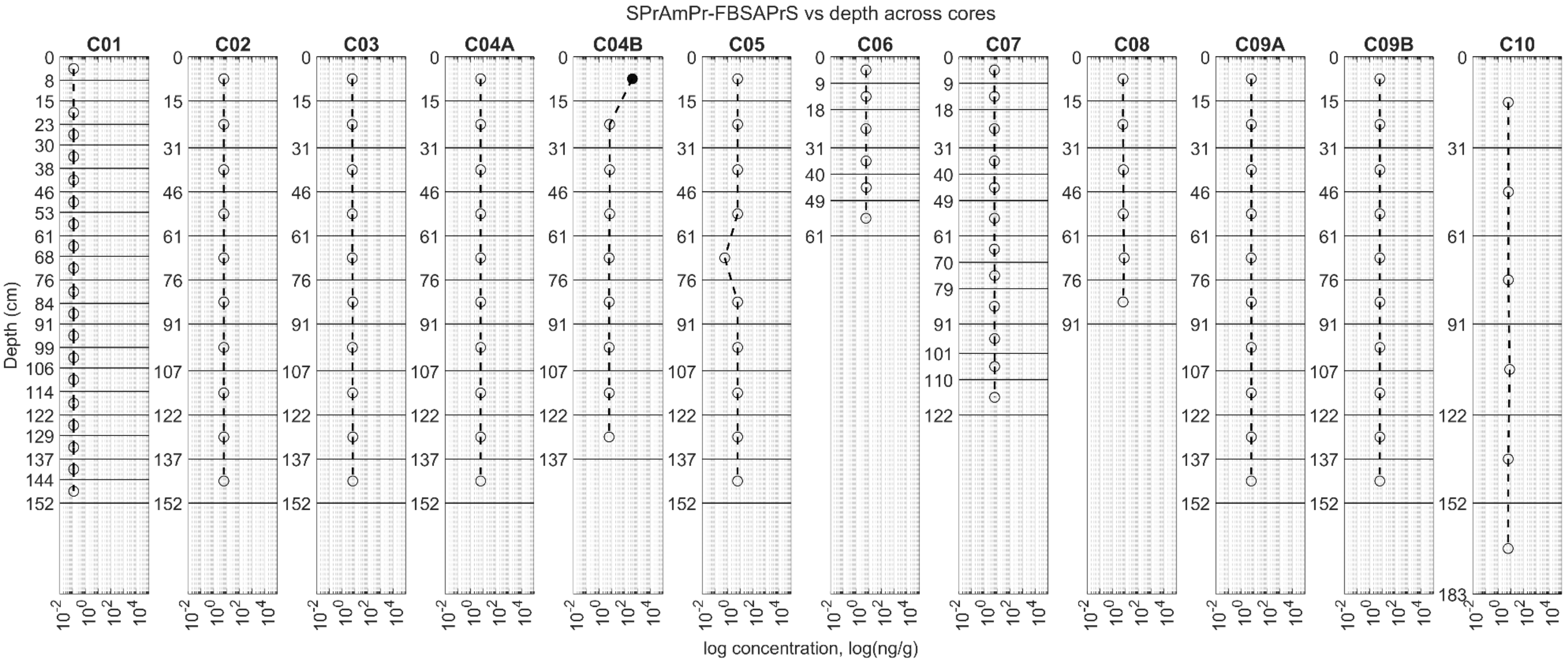

Figure S28. Vertical distribution profile of **SPrAmPr-FPeSAPrS** across the twelve studied cores

Note that the concentration is shown on a log-base-10 scale. For any given plot and compound, open markers with dashed-line connectors represent sampled depth intervals where the compounds' concentration was below the reporting limit – the location of the open marker along the x-axis is representative of those reporting limits.

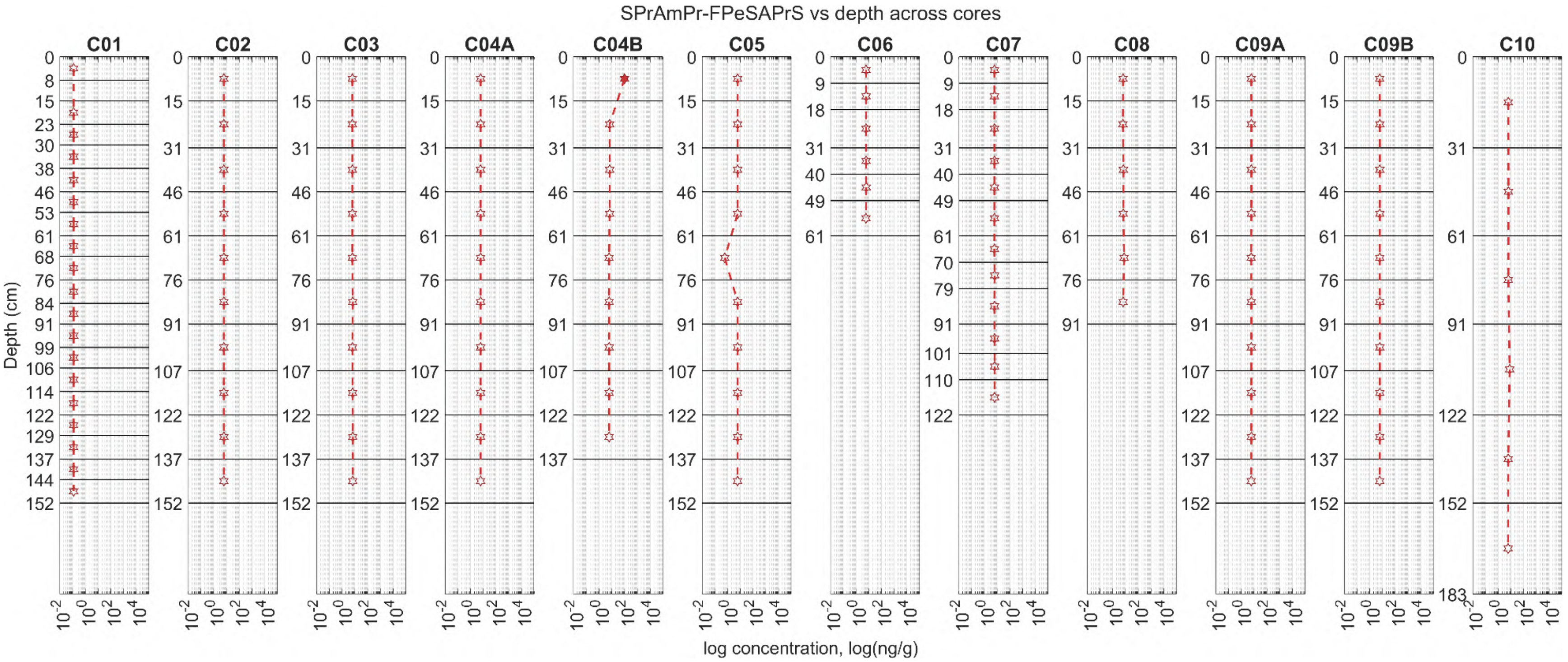

Figure S29. Vertical distribution profile of **SPrAmPr-FHxSAPrS** across the twelve studied cores

Note that the concentration is shown on a log-base-10 scale. For any given plot and compound, open markers with dashed-line connectors represent sampled depth intervals where the compounds' concentration was below the reporting limit – the location of the open marker along the x-axis is representative of those reporting limits.

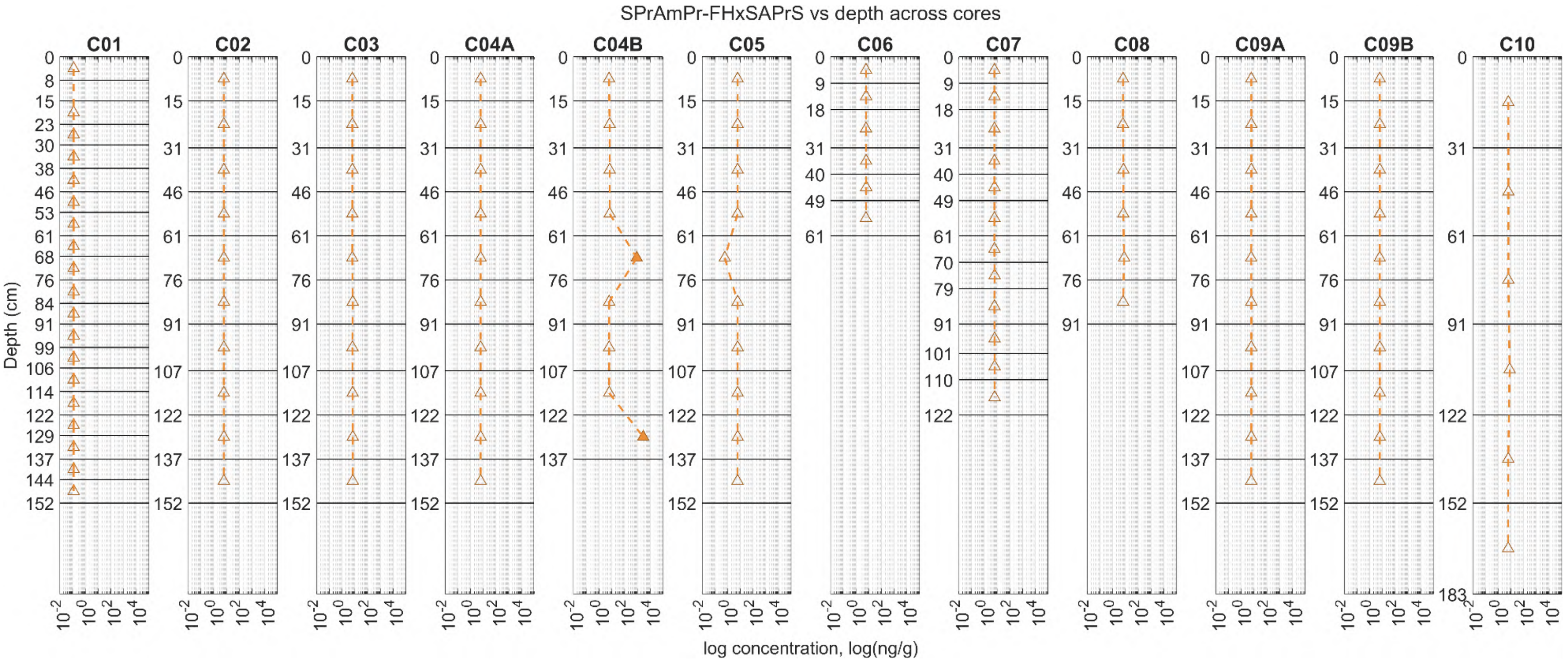

Figure S30. Vertical distribution profile of **diOHPrAm-MeOHPr-FBSA** across the twelve studied cores

Note that the concentration is shown on a log-base-10 scale. For any given plot and compound, open markers with dashed-line connectors represent sampled depth intervals where the compounds' concentration was below the reporting limit – the location of the open marker along the x-axis is representative of those reporting limits.

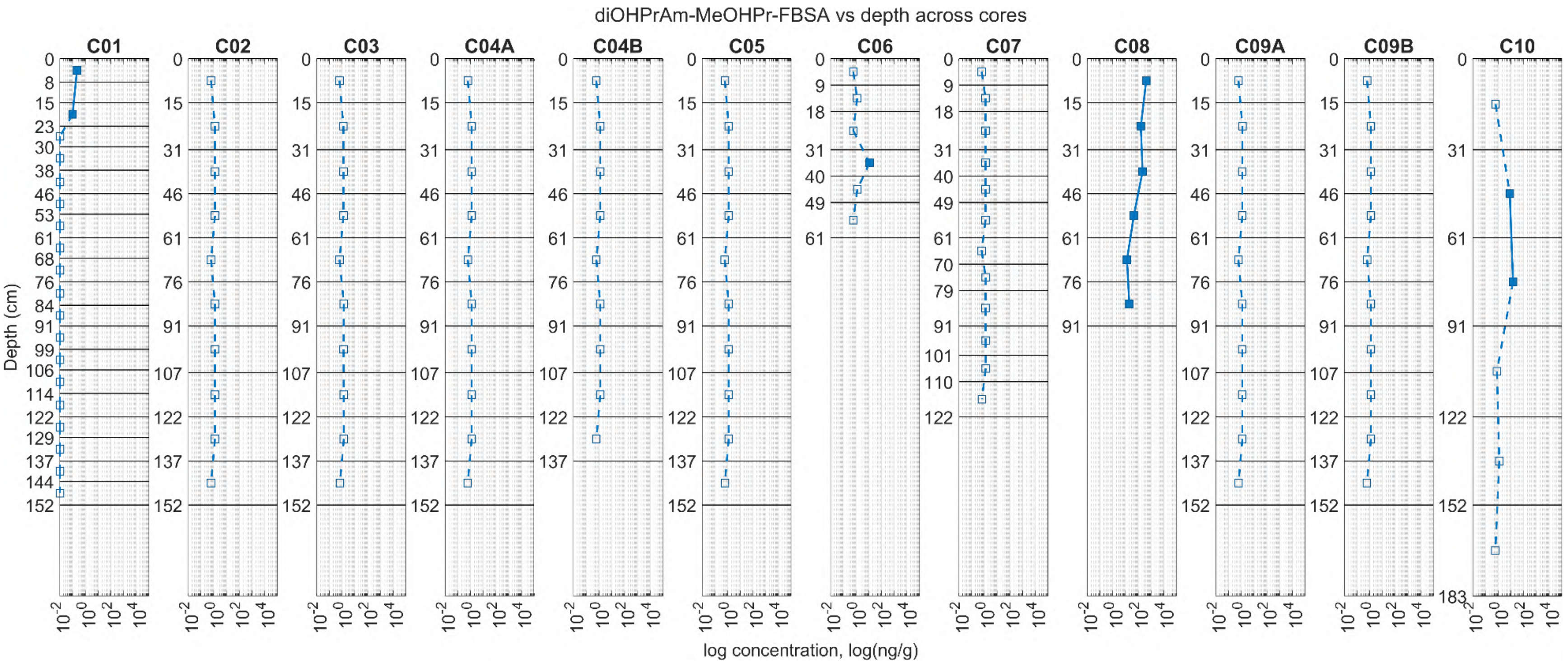

Figure S31. Vertical distribution profile of **diOHPrAm-MeOHPr-FPeSA** across the twelve studied cores

Note that the concentration is shown on a log-base-10 scale. For any given plot and compound, open markers with dashed-line connectors represent sampled depth intervals where the compounds' concentration was below the reporting limit – the location of the open marker along the x-axis is representative of those reporting limits.

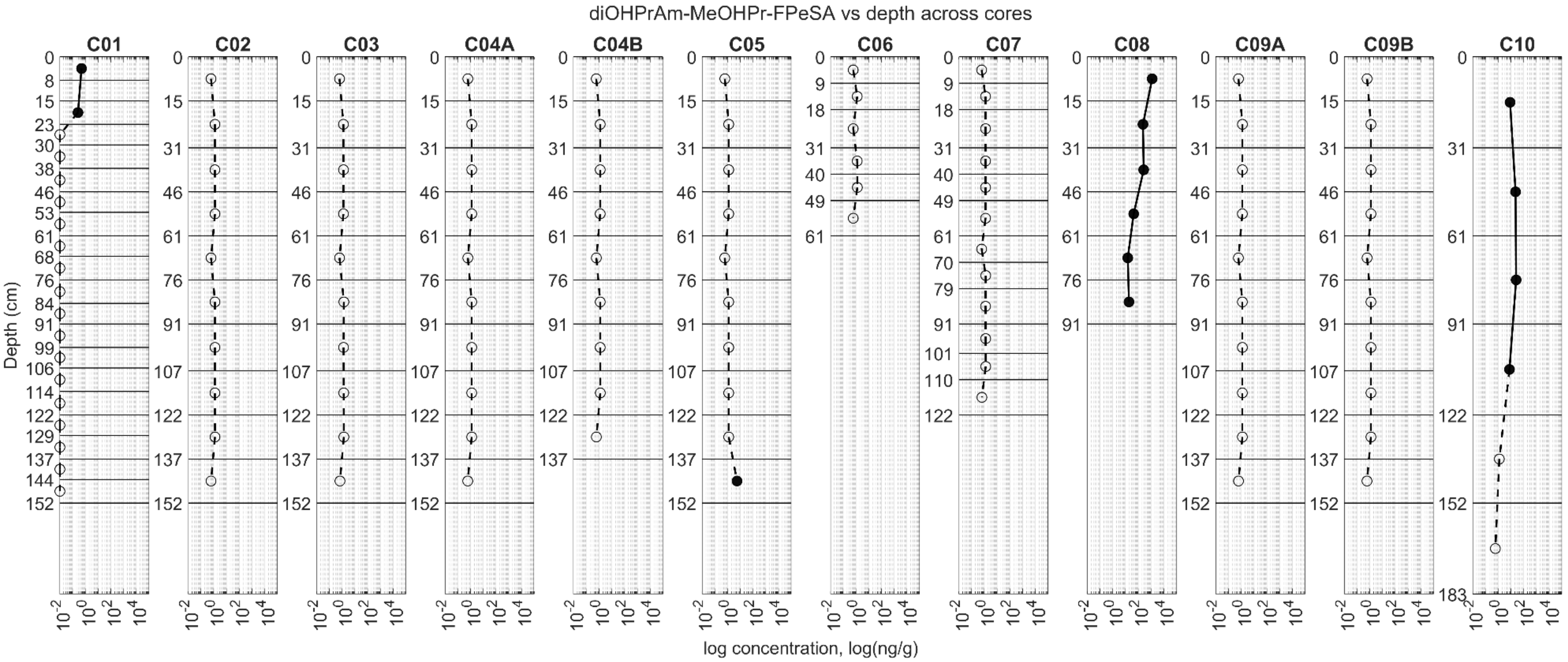

Figure S32. Vertical distribution profile of **diOHPrAm-MeOHPr-FHxSA** across the twelve studied cores

Note that the concentration is shown on a log-base-10 scale. For any given plot and compound, open markers with dashed-line connectors represent sampled depth intervals where the compounds' concentration was below the reporting limit – the location of the open marker along the x-axis is representative of those reporting limits.

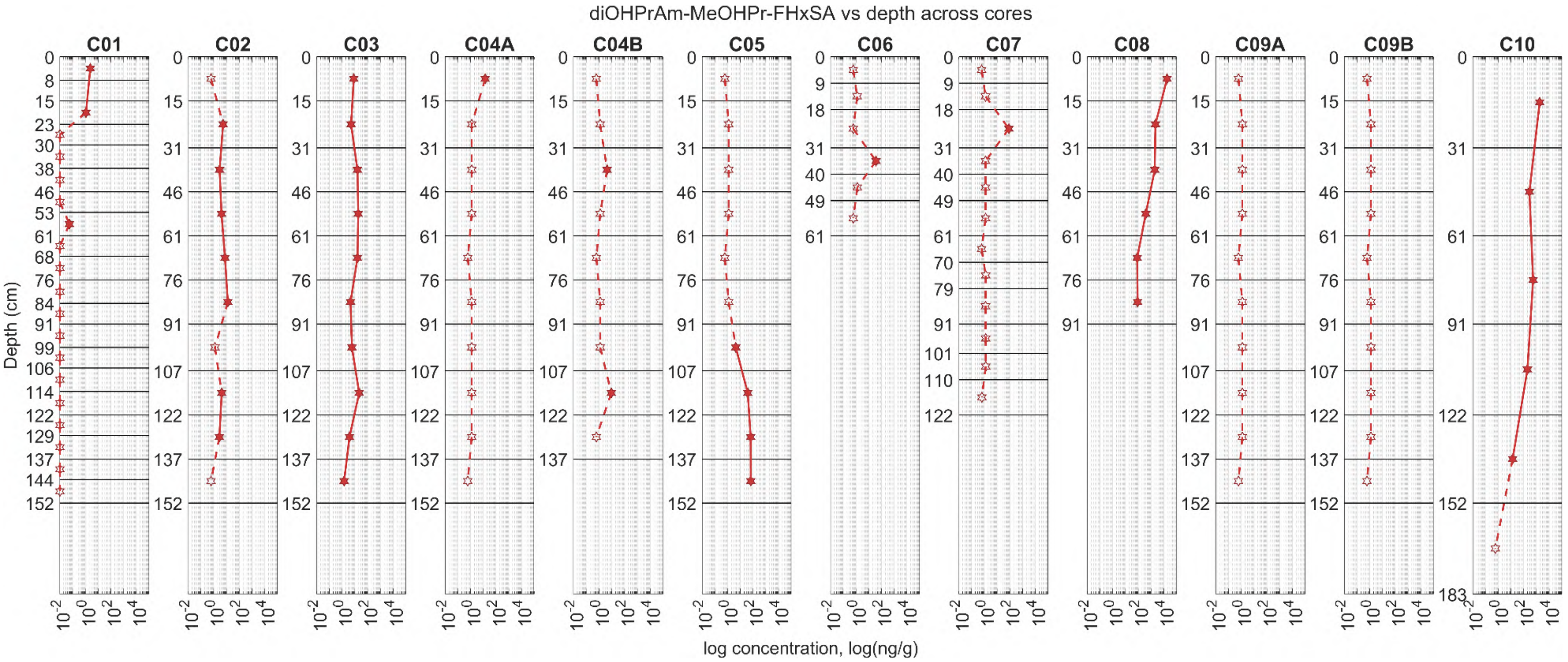

Figure S33. Vertical distribution profile of **diOHPrAm-MeOHPr-FBSAPrS** across the twelve studied cores

Note that the concentration is shown on a log-base-10 scale. For any given plot and compound, open markers with dashed-line connectors represent sampled depth intervals where the compounds' concentration was below the reporting limit – the location of the open marker along the x-axis is representative of those reporting limits.

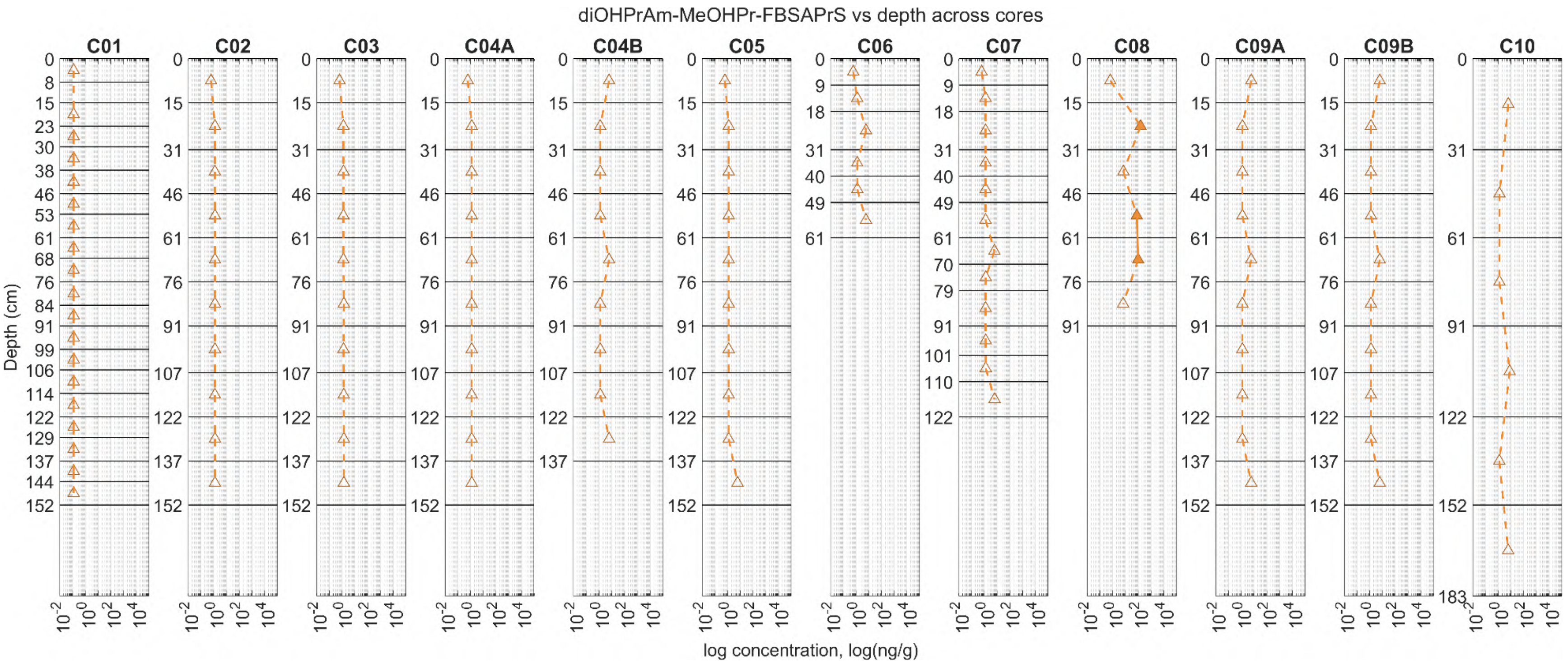

Figure S34. Vertical distribution profile of **diOHPrAm-MeOHPr-FPeSAPrS** across the twelve studied cores

Note that the concentration is shown on a log-base-10 scale. For any given plot and compound, open markers with dashed-line connectors represent sampled depth intervals where the compounds' concentration was below the reporting limit – the location of the open marker along the x-axis is representative of those reporting limits.

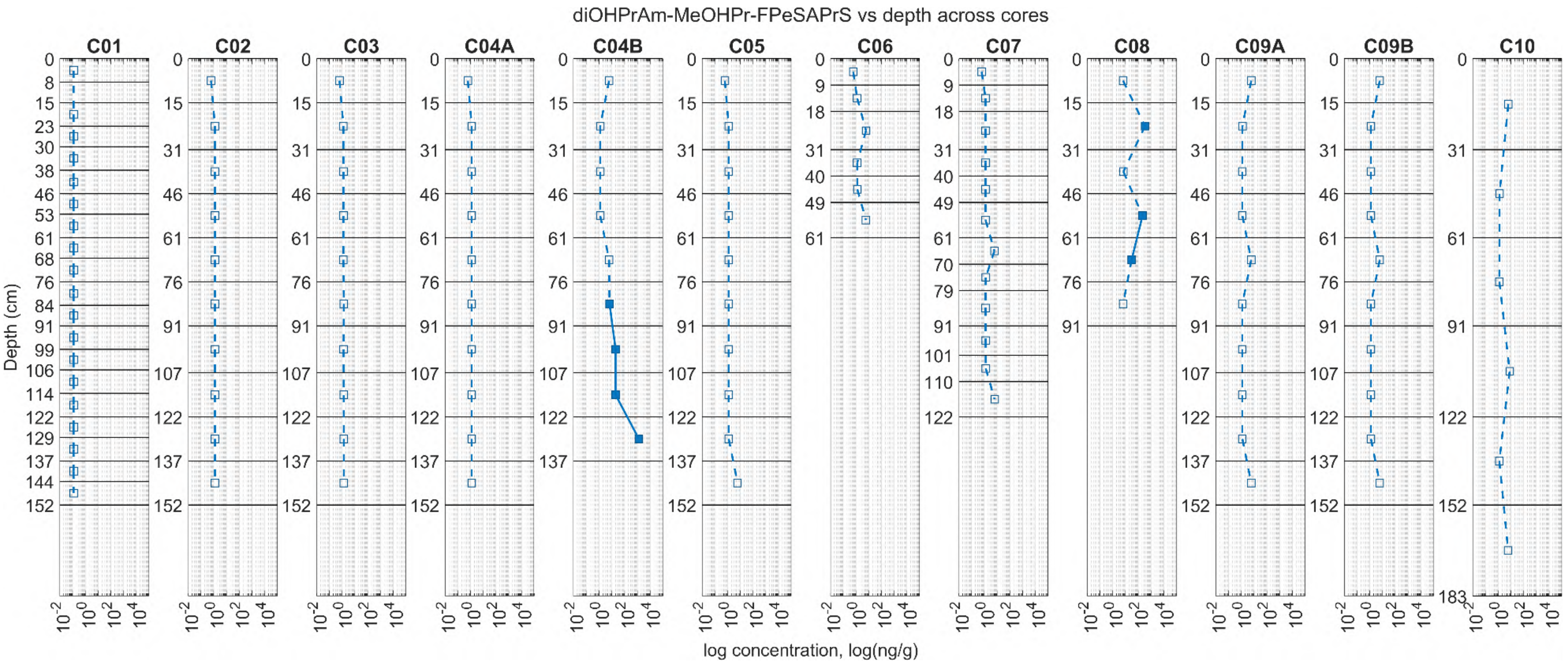

Figure S35. Vertical distribution profile of **diOHPrAm-MeOHPr-FHxSAPrS** across the twelve studied cores

Note that the concentration is shown on a log-base-10 scale. For any given plot and compound, open markers with dashed-line connectors represent sampled depth intervals where the compounds' concentration was below the reporting limit – the location of the open marker along the x-axis is representative of those reporting limits.

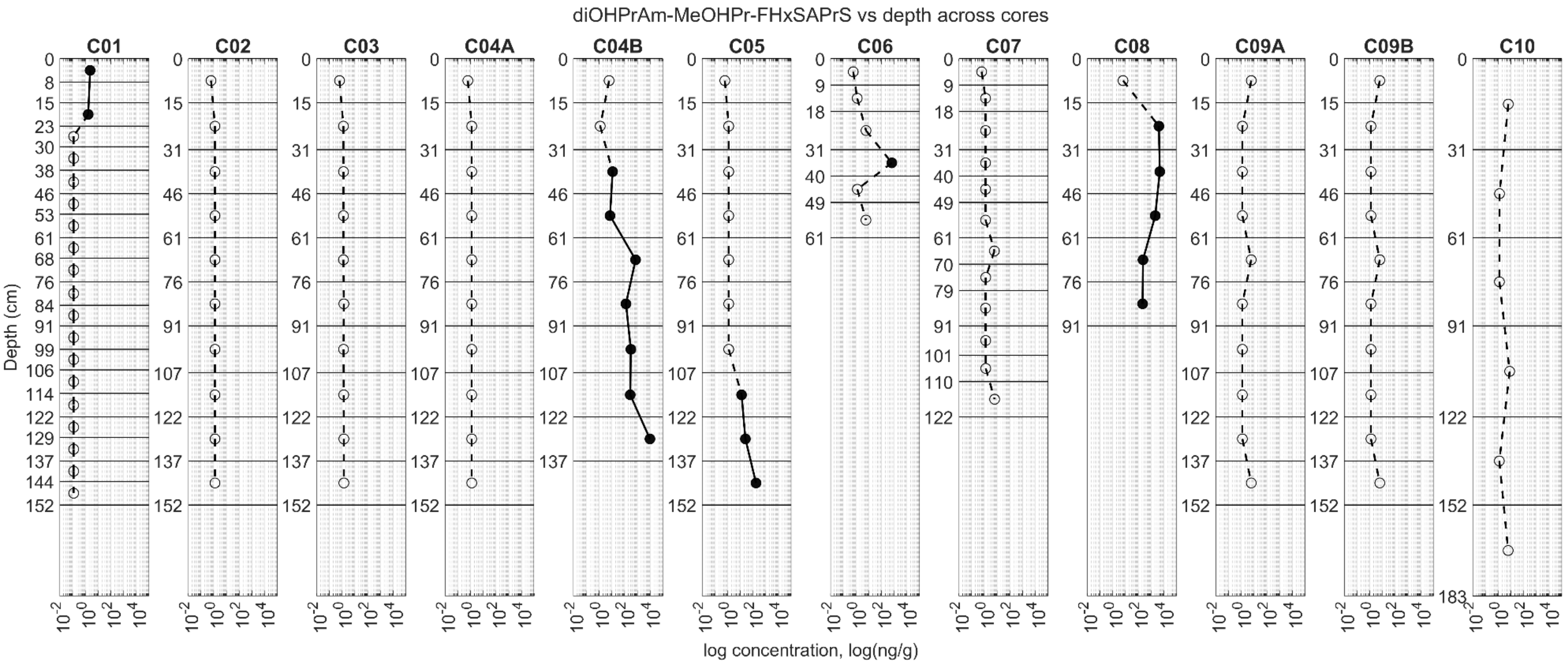

Figure S36. Vertical distribution profile of **Cl-PFBS** across the twelve studied cores

Note that the concentration is shown on a log-base-10 scale. For any given plot and compound, open markers with dashed-line connectors represent sampled depth intervals where the compounds' concentration was below the reporting limit – the location of the open marker along the x-axis is representative of those reporting limits.

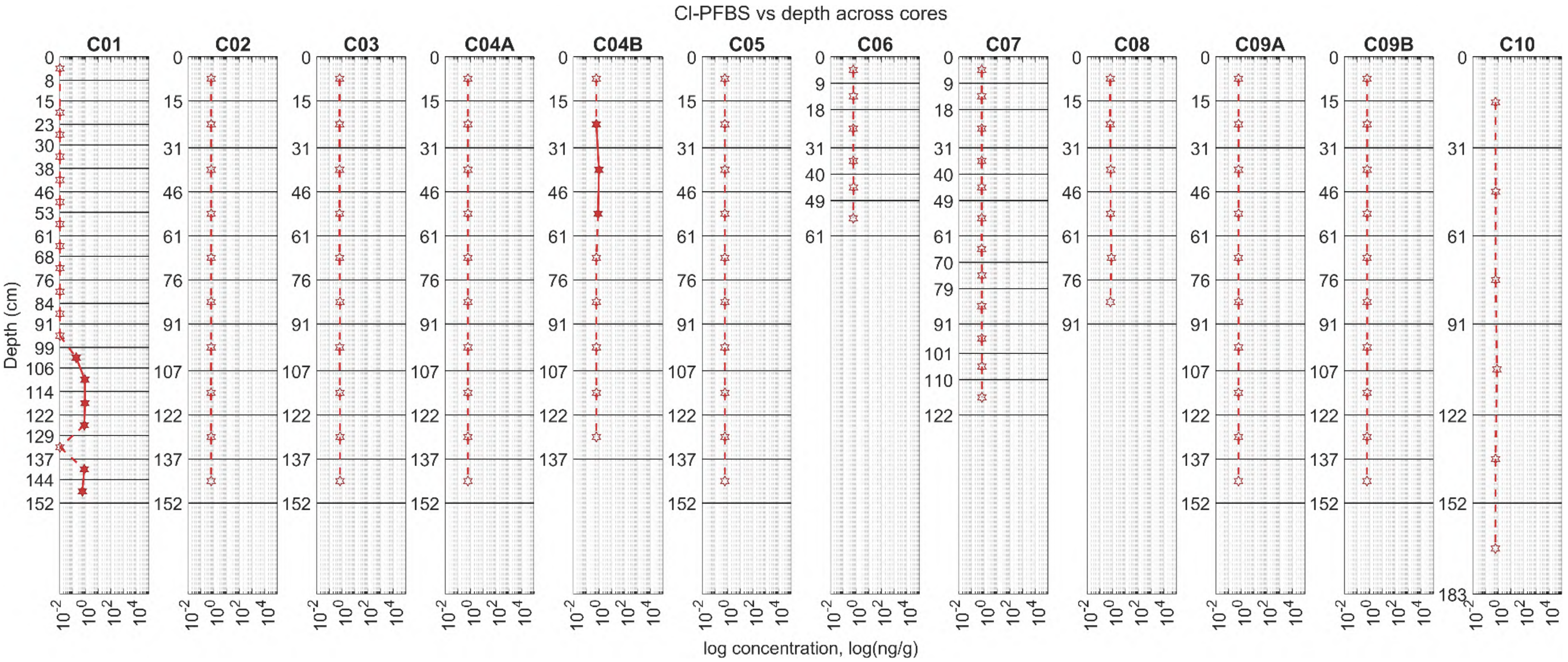

Figure S37. Vertical distribution profile of **Cl-PFHxS** across the twelve studied cores

Note that the concentration is shown on a log-base-10 scale. For any given plot and compound, open markers with dashed-line connectors represent sampled depth intervals where the compounds' concentration was below the reporting limit – the location of the open marker along the x-axis is representative of those reporting limits.

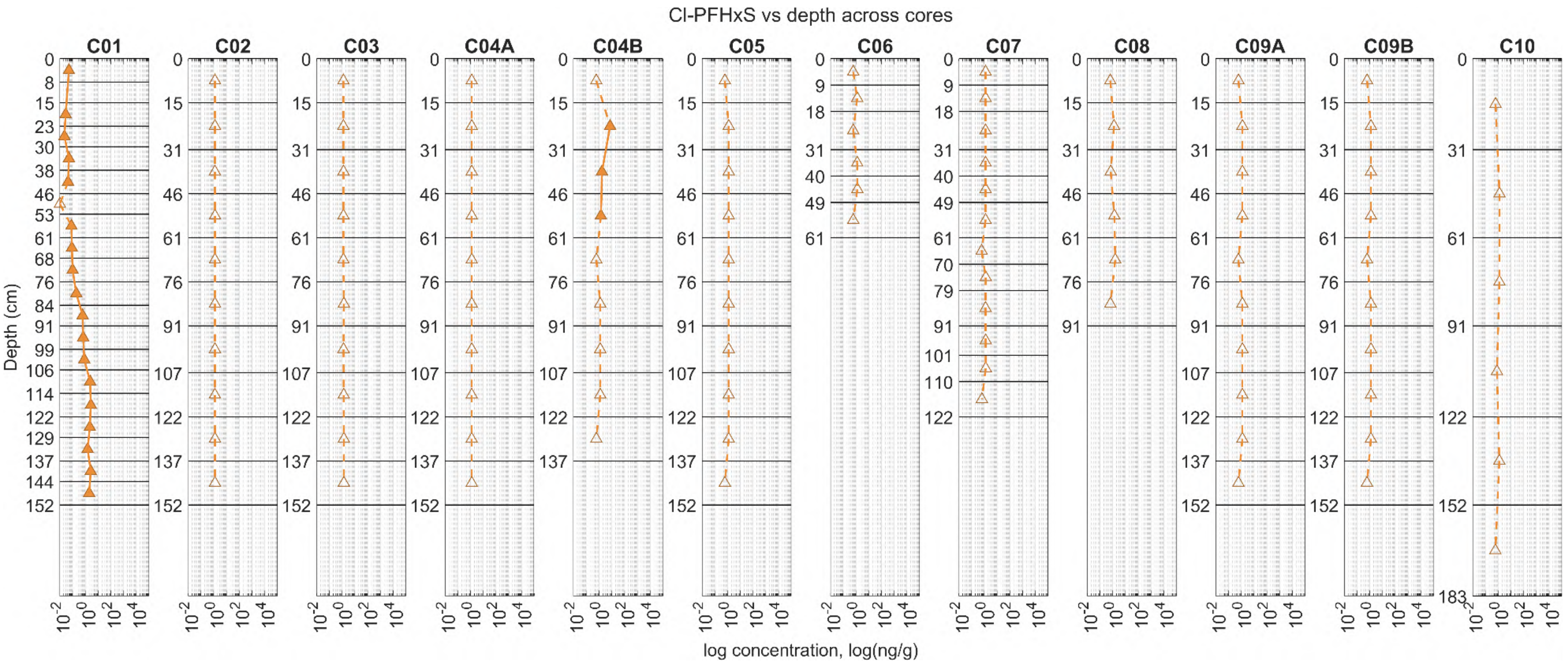

Figure S38. Vertical distribution profile of **Cl-PFOS** across the twelve studied cores

Note that the concentration is shown on a log-base-10 scale. For any given plot and compound, open markers with dashed-line connectors represent sampled depth intervals where the compounds' concentration was below the reporting limit – the location of the open marker along the x-axis is representative of those reporting limits.

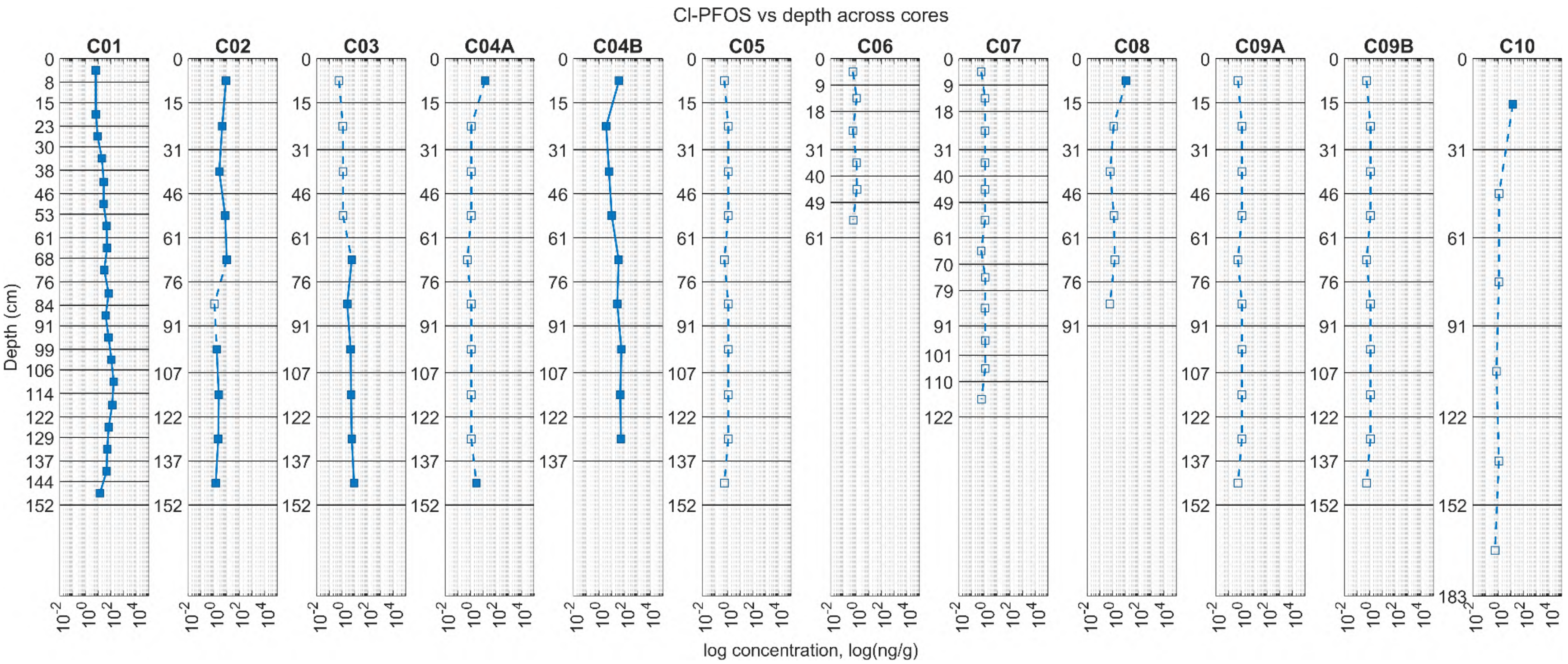

Figure S39. Vertical distribution profile of **PFBSi** across the twelve studied cores

Note that the concentration is shown on a log-base-10 scale. For any given plot and compound, open markers with dashed-line connectors represent sampled depth intervals where the compounds' concentration was below the reporting limit – the location of the open marker along the x-axis is representative of those reporting limits.

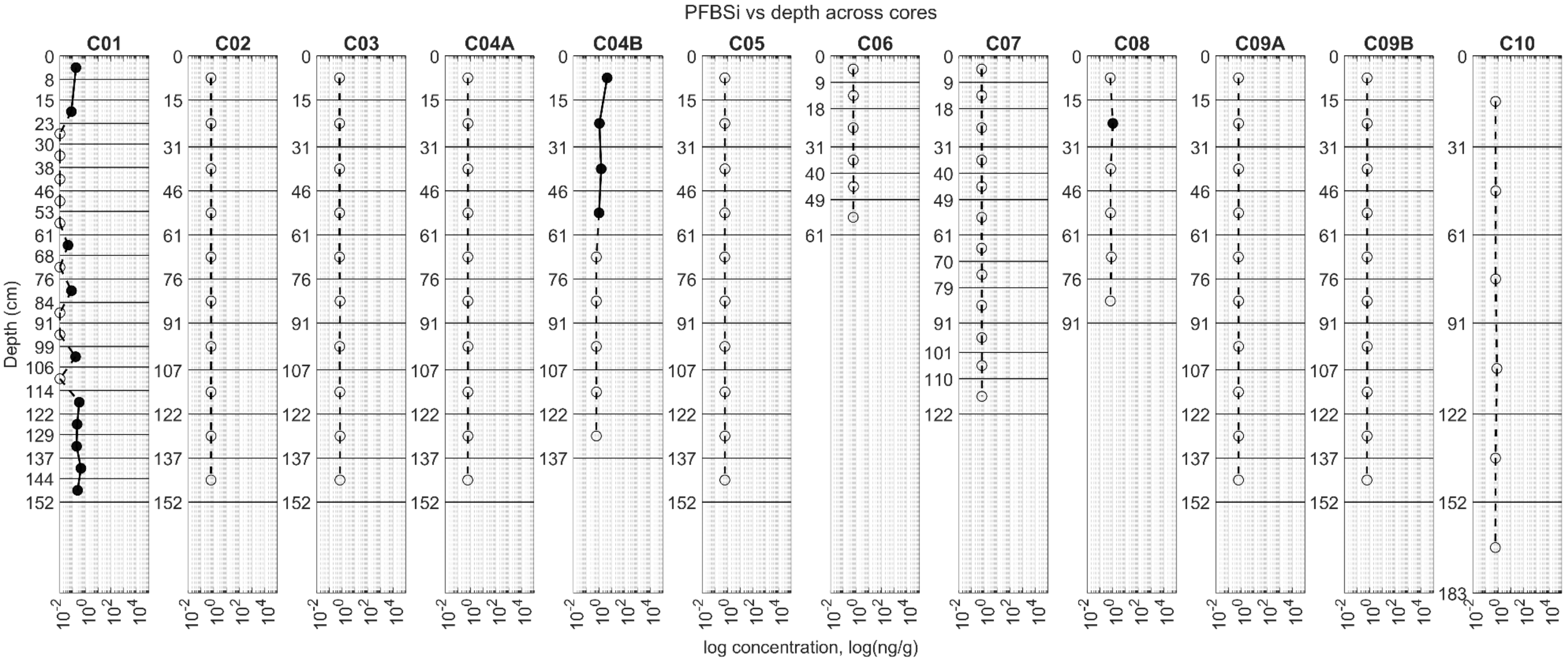

Figure S40. Vertical distribution profile of **PFPeSi** across the twelve studied cores

Note that the concentration is shown on a log-base-10 scale. For any given plot and compound, open markers with dashed-line connectors represent sampled depth intervals where the compounds' concentration was below the reporting limit – the location of the open marker along the x-axis is representative of those reporting limits.

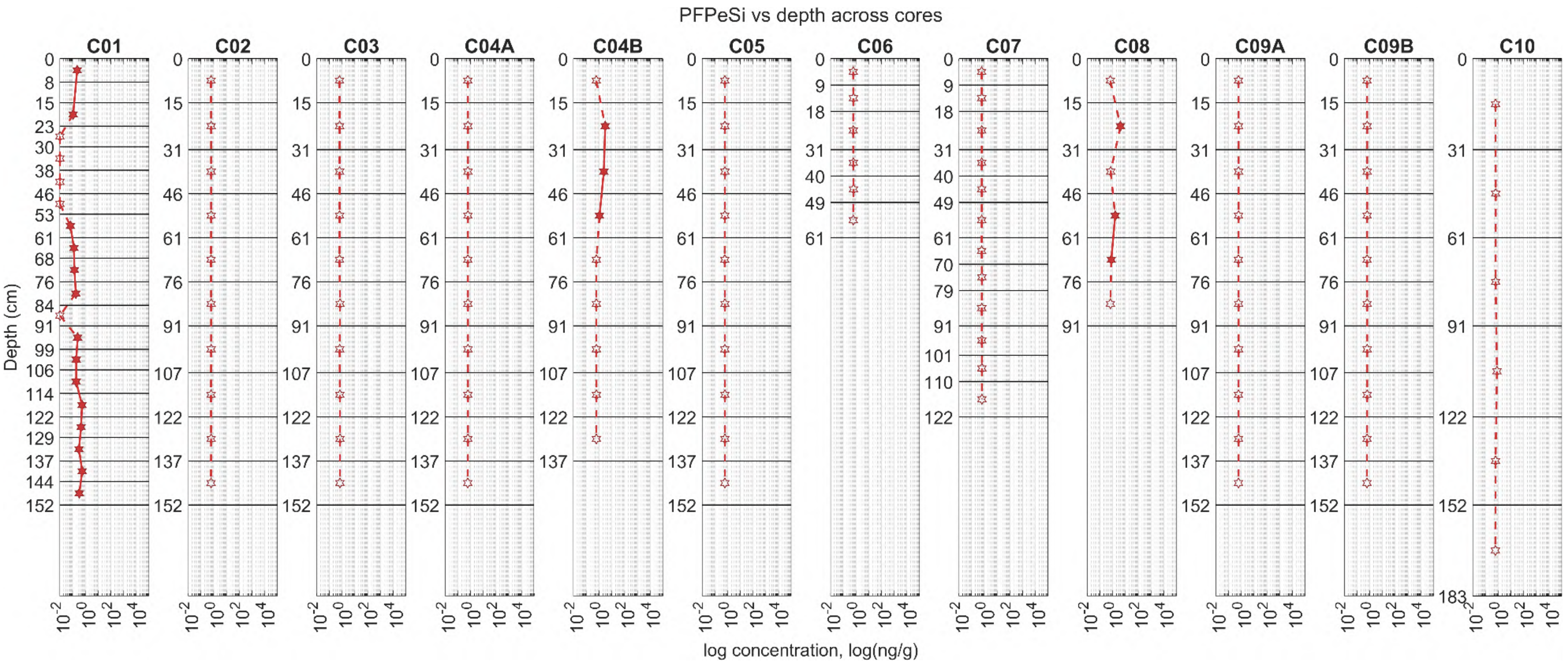

Figure S41. Vertical distribution profile of **PFHxSi** across the twelve studied cores

Note that the concentration is shown on a log-base-10 scale. For any given plot and compound, open markers with dashed-line connectors represent sampled depth intervals where the compounds' concentration was below the reporting limit – the location of the open marker along the x-axis is representative of those reporting limits.

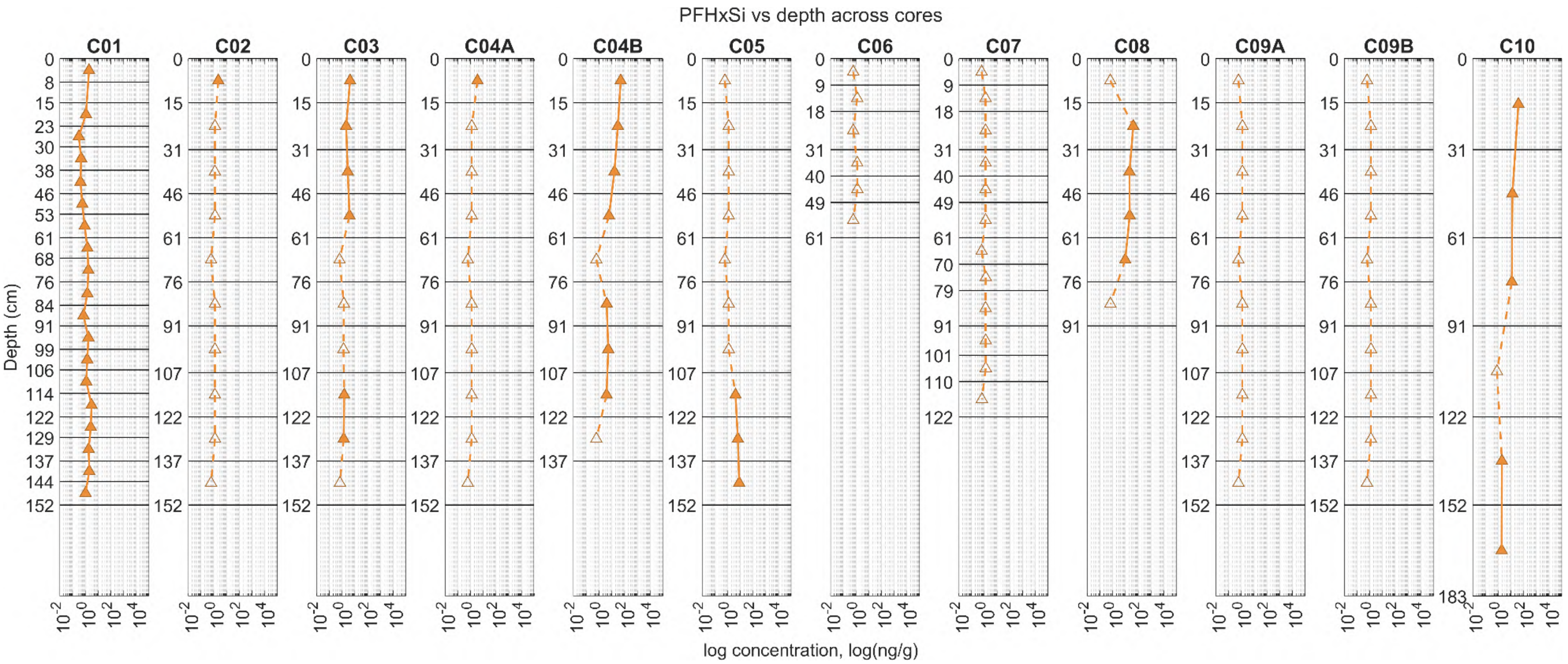

Figure S42. Vertical distribution profile of **PFOSi** across the twelve studied cores

Note that the concentration is shown on a log-base-10 scale. For any given plot and compound, open markers with dashed-line connectors represent sampled depth intervals where the compounds' concentration was below the reporting limit – the location of the open marker along the x-axis is representative of those reporting limits.

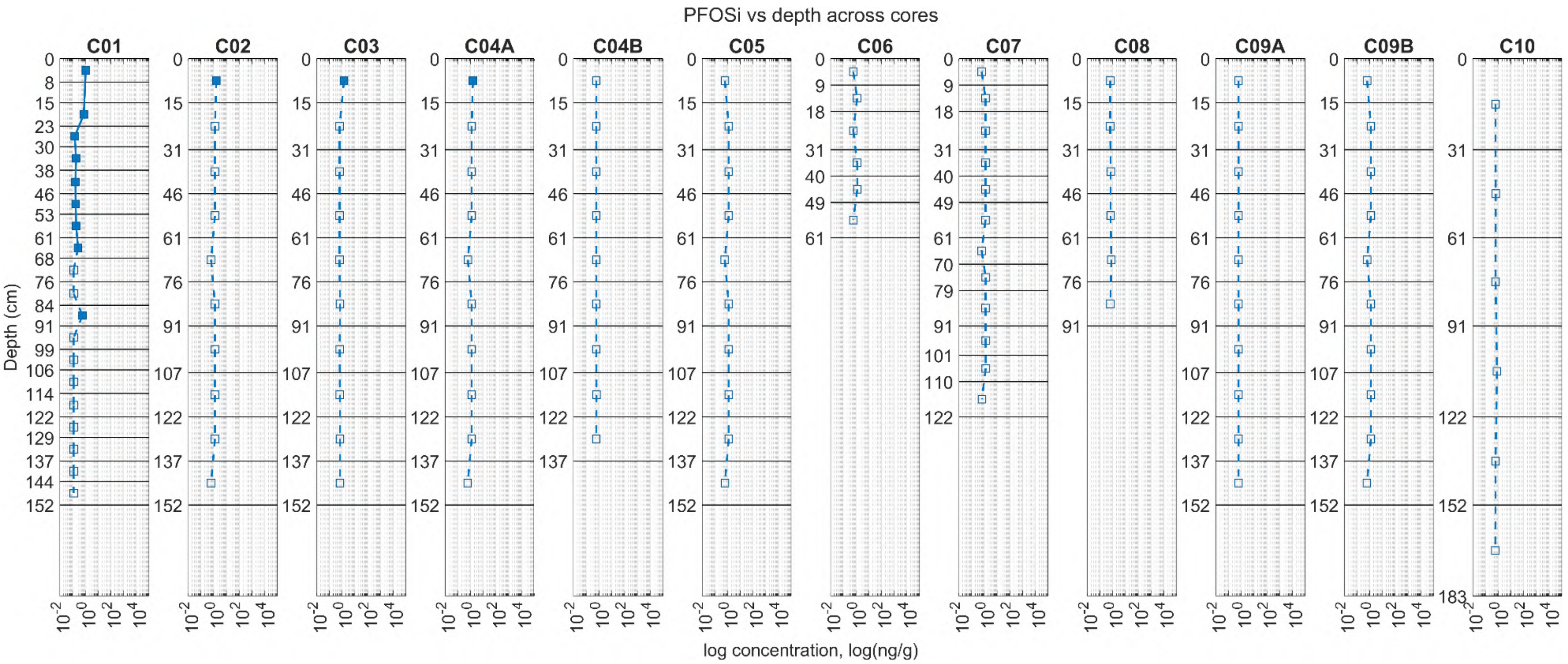

Figure S43. Vertical distribution profile of **4:2 FTSO2PrAd-DiMeEtS** across the twelve studied cores

Note that the concentration is shown on a log-base-10 scale. For any given plot and compound, open markers with dashed-line connectors represent sampled depth intervals where the compounds' concentration was below the reporting limit – the location of the open marker along the x-axis is representative of those reporting limits.

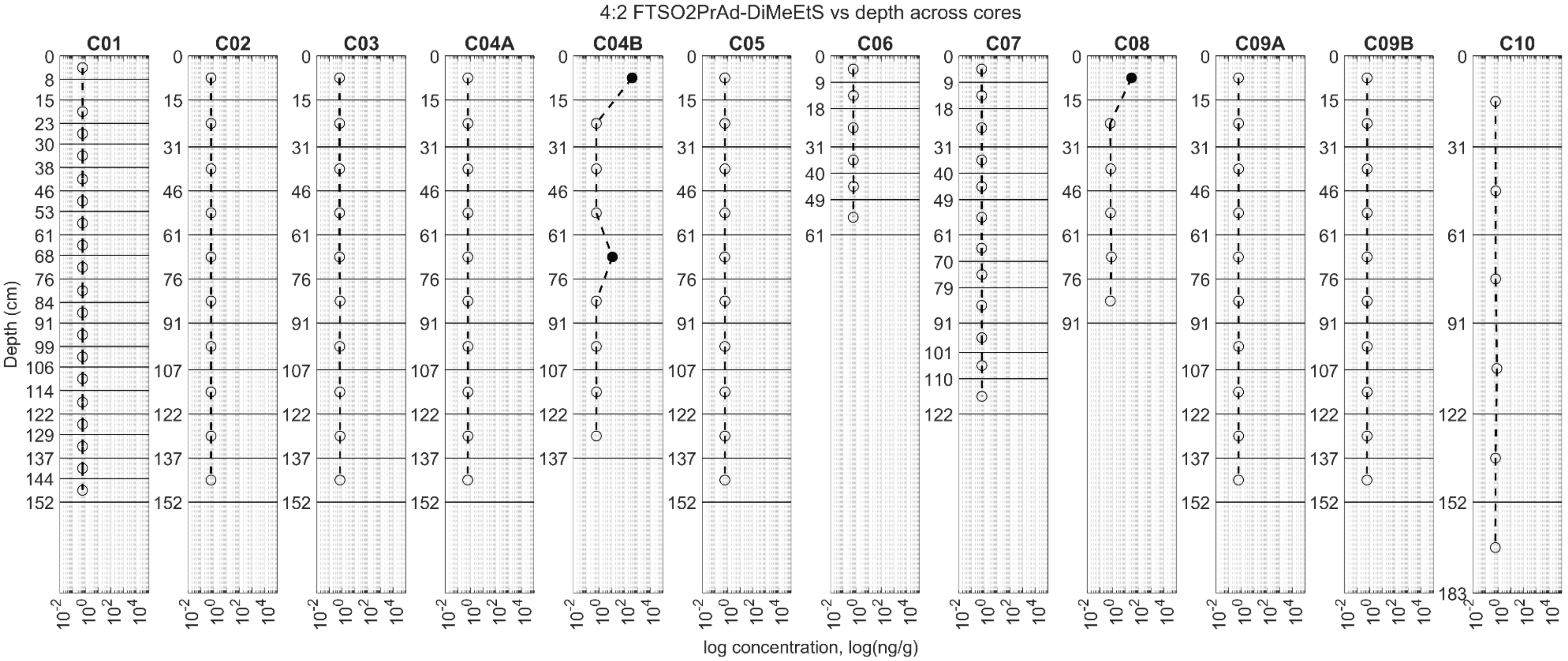

Figure S44. Vertical distribution profile of **6:2 FTSO2PrAd-DiMeEtS** across the twelve studied cores

Note that the concentration is shown on a log-base-10 scale. For any given plot and compound, open markers with dashed-line connectors represent sampled depth intervals where the compounds' concentration was below the reporting limit – the location of the open marker along the x-axis is representative of those reporting limits.

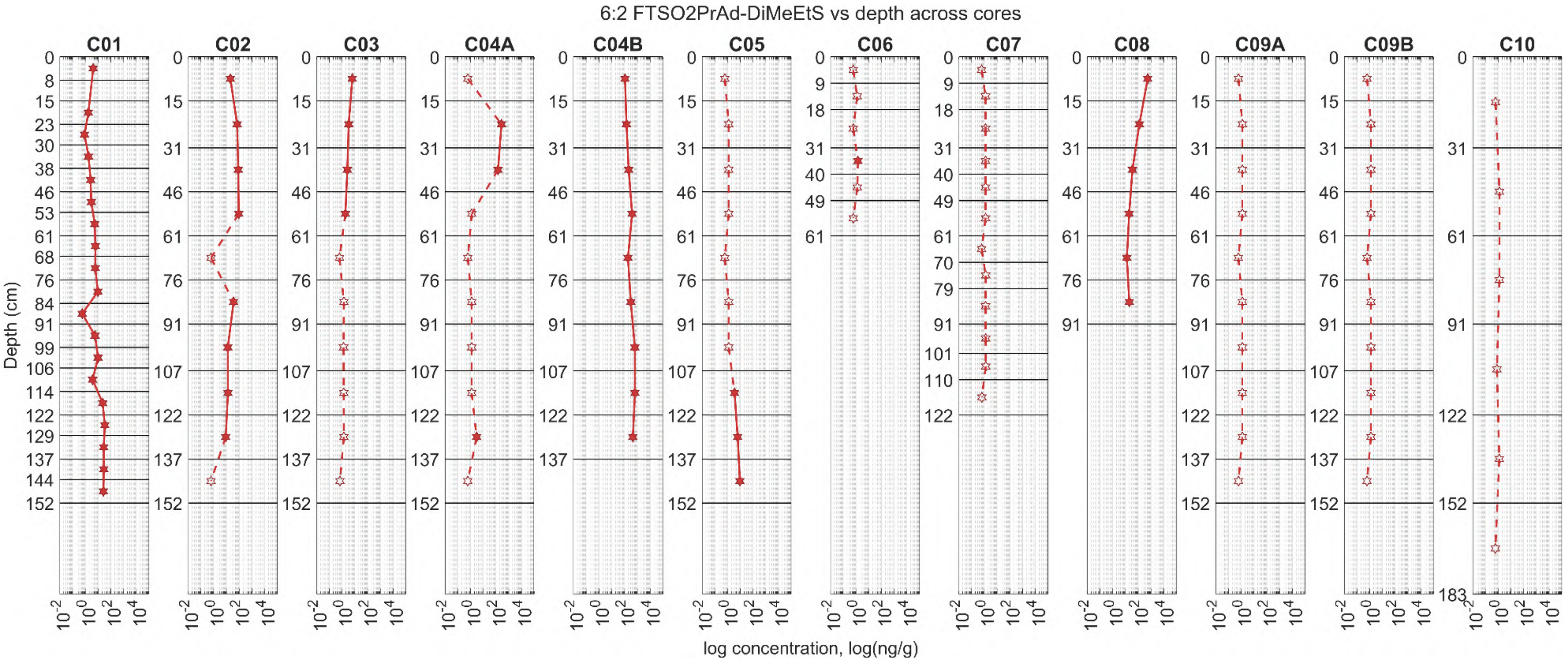

Figure S45. Vertical distribution profile of **8:2 FTSO2PrAd-DiMeEtS** across the twelve studied cores

Note that the concentration is shown on a log-base-10 scale. For any given plot and compound, open markers with dashed-line connectors represent sampled depth intervals where the compounds' concentration was below the reporting limit – the location of the open marker along the x-axis is representative of those reporting limits.

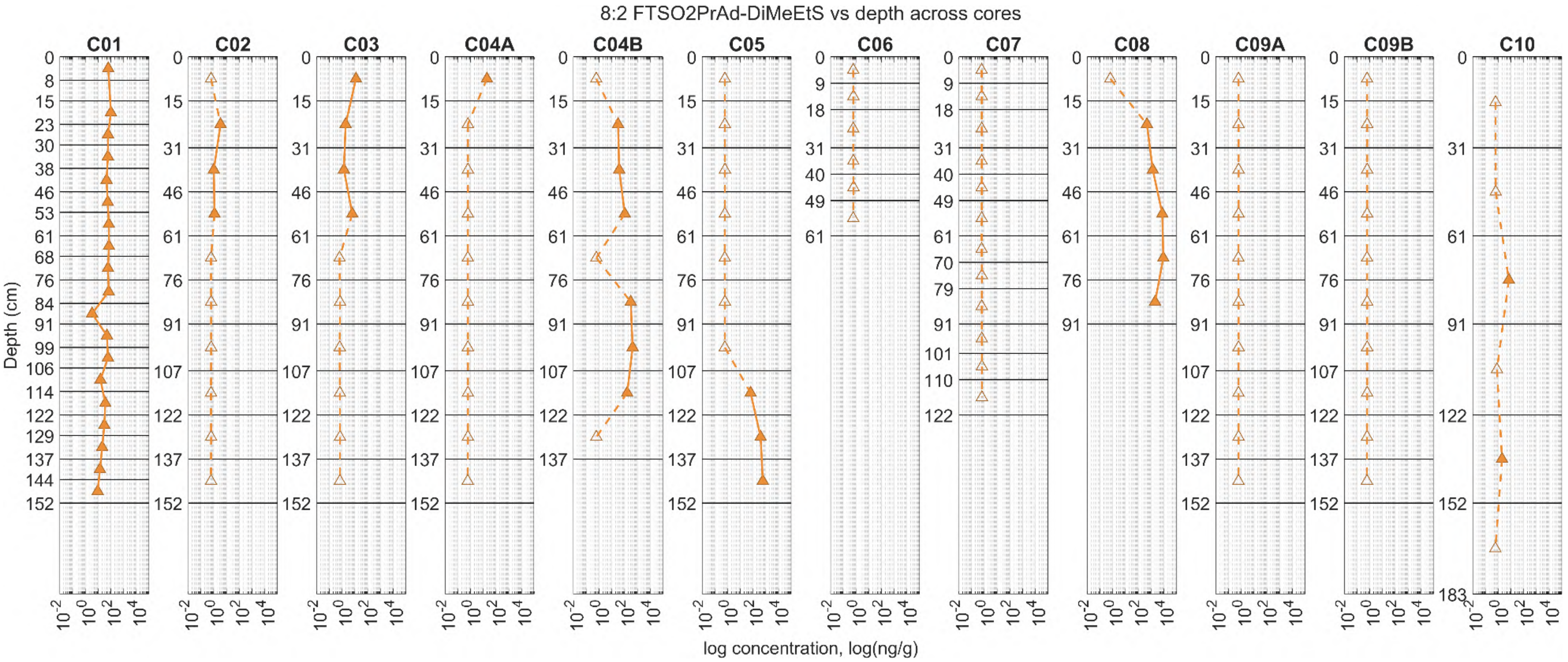

Figure S46. Vertical distribution profile of **4:2 FTS** across the twelve studied cores

Note that the concentration is shown on a log-base-10 scale. For any given plot and compound, open markers with dashed-line connectors represent sampled depth intervals where the compounds' concentration was below the reporting limit – the location of the open marker along the x-axis is representative of those reporting limits.

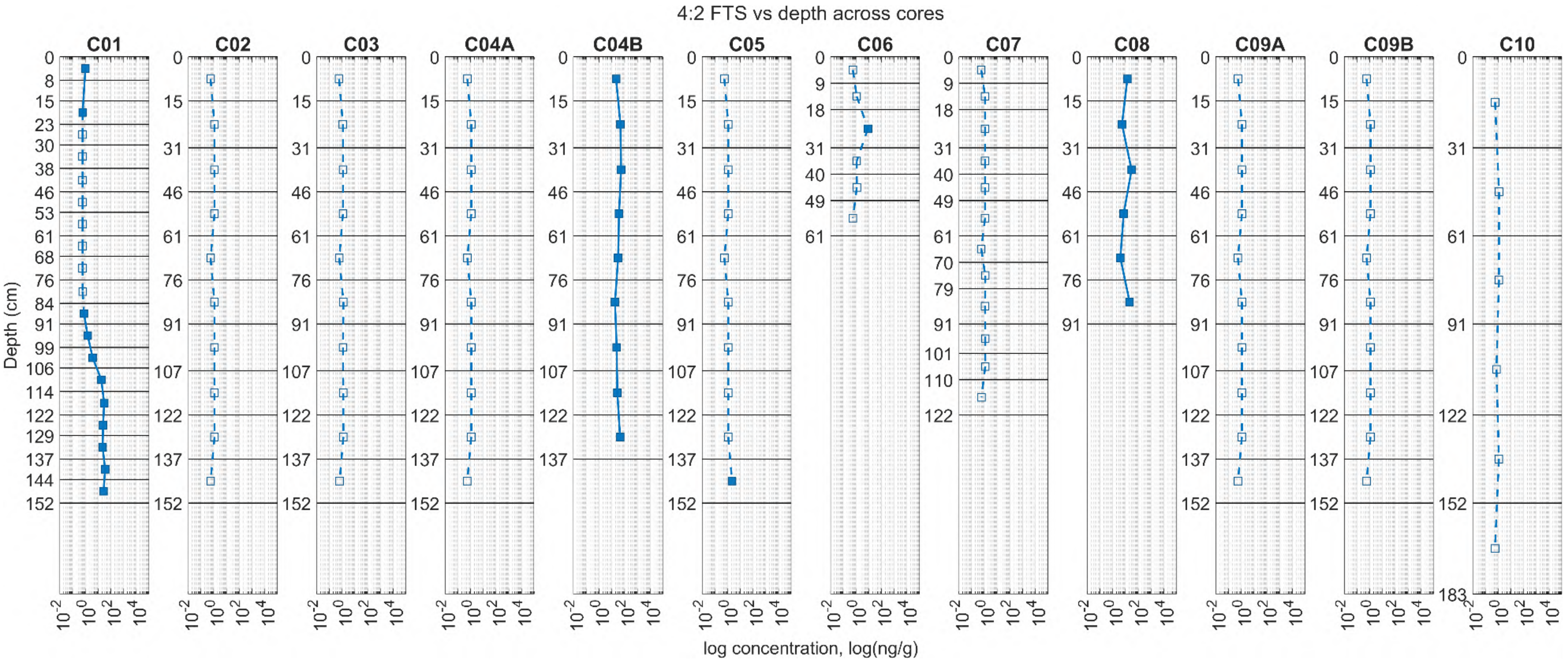

Figure S47. Vertical distribution profile of **6:2 FTS** across the twelve studied cores

Note that the concentration is shown on a log-base-10 scale. For any given plot and compound, open markers with dashed-line connectors represent sampled depth intervals where the compounds' concentration was below the reporting limit – the location of the open marker along the x-axis is representative of those reporting limits.

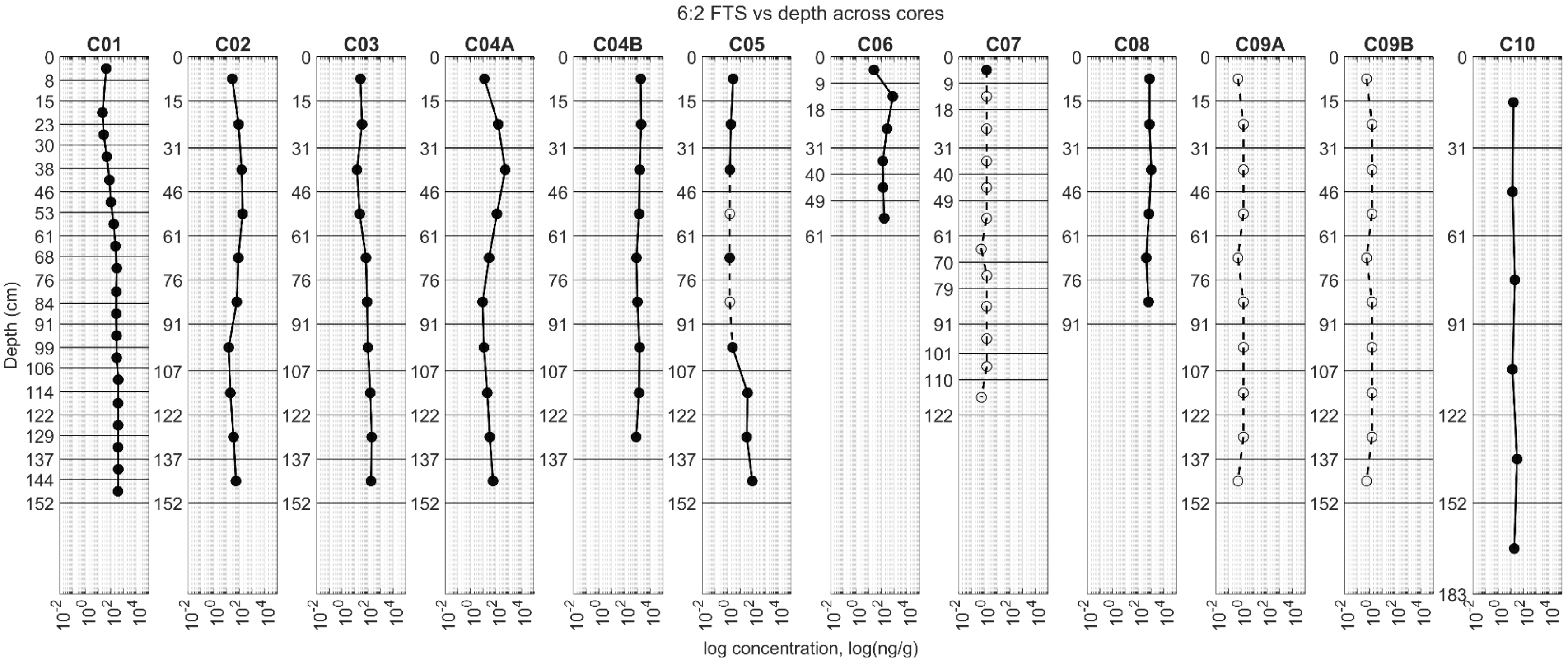

Figure S48. Vertical distribution profile of **8:2 FTS** across the twelve studied cores

Note that the concentration is shown on a log-base-10 scale. For any given plot and compound, open markers with dashed-line connectors represent sampled depth intervals where the compounds' concentration was below the reporting limit – the location of the open marker along the x-axis is representative of those reporting limits.

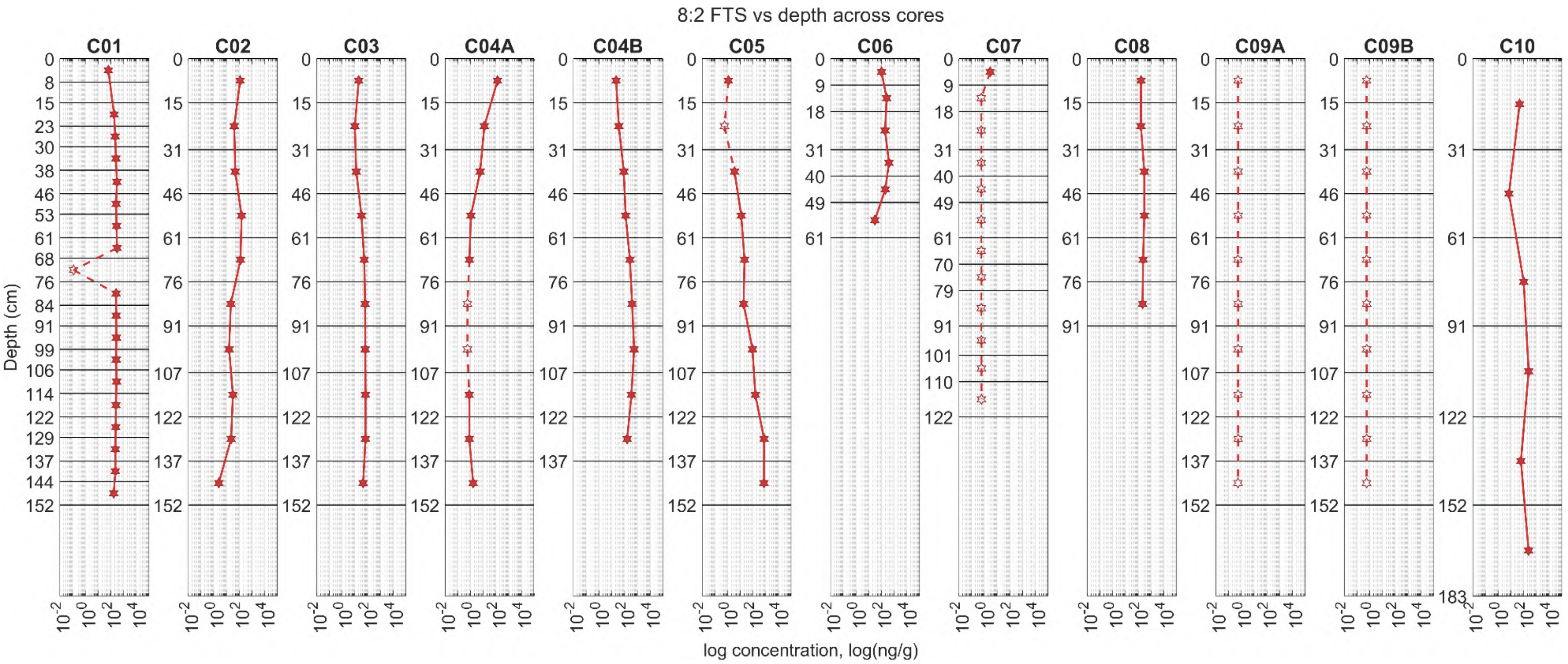

Figure S49. Vertical distribution profile of **10:2 FTS** across the twelve studied cores

Note that the concentration is shown on a log-base-10 scale. For any given plot and compound, open markers with dashed-line connectors represent sampled depth intervals where the compounds' concentration was below the reporting limit – the location of the open marker along the x-axis is representative of those reporting limits.

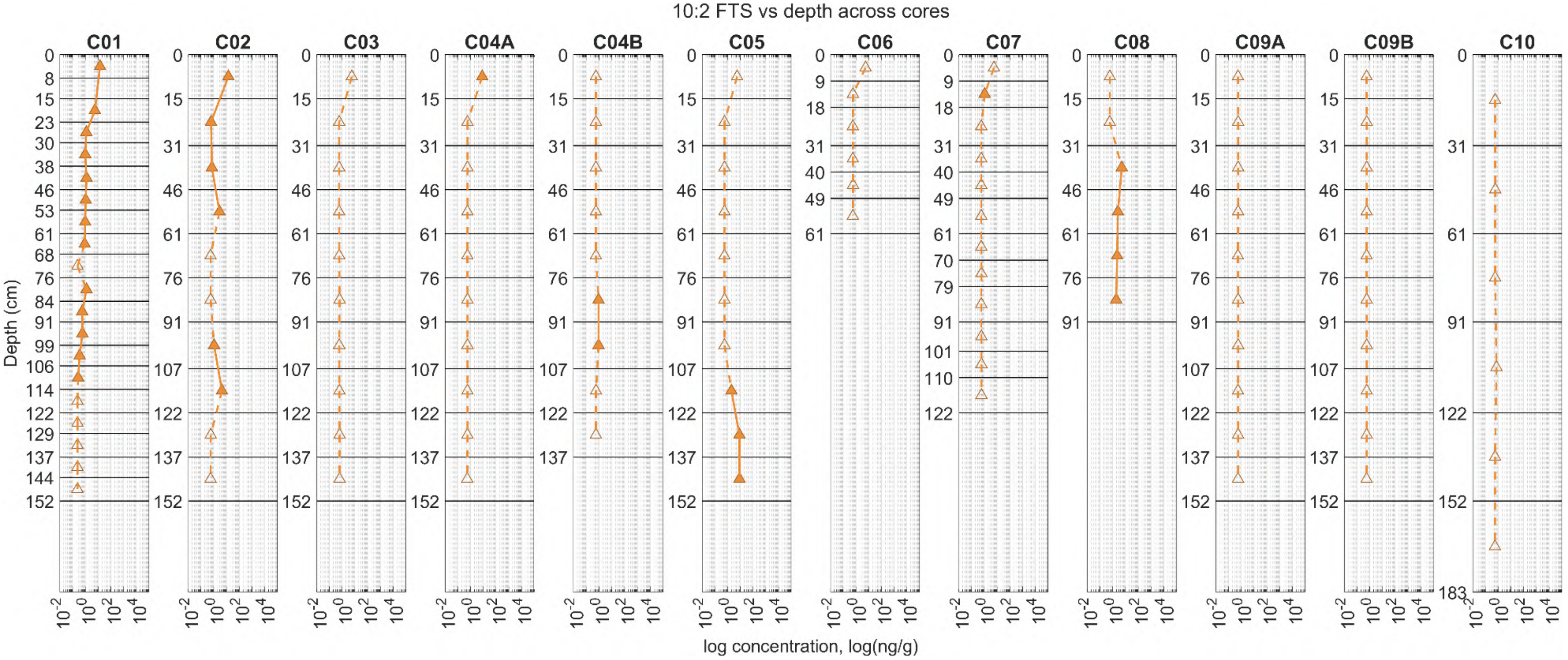

Figure S50. Vertical distribution profile of **6:2 FTTh-PrAd-DiMeEtS** across the twelve studied cores

Note that the concentration is shown on a log-base-10 scale. For any given plot and compound, open markers with dashed-line connectors represent sampled depth intervals where the compounds' concentration was below the reporting limit – the location of the open marker along the x-axis is representative of those reporting limits.

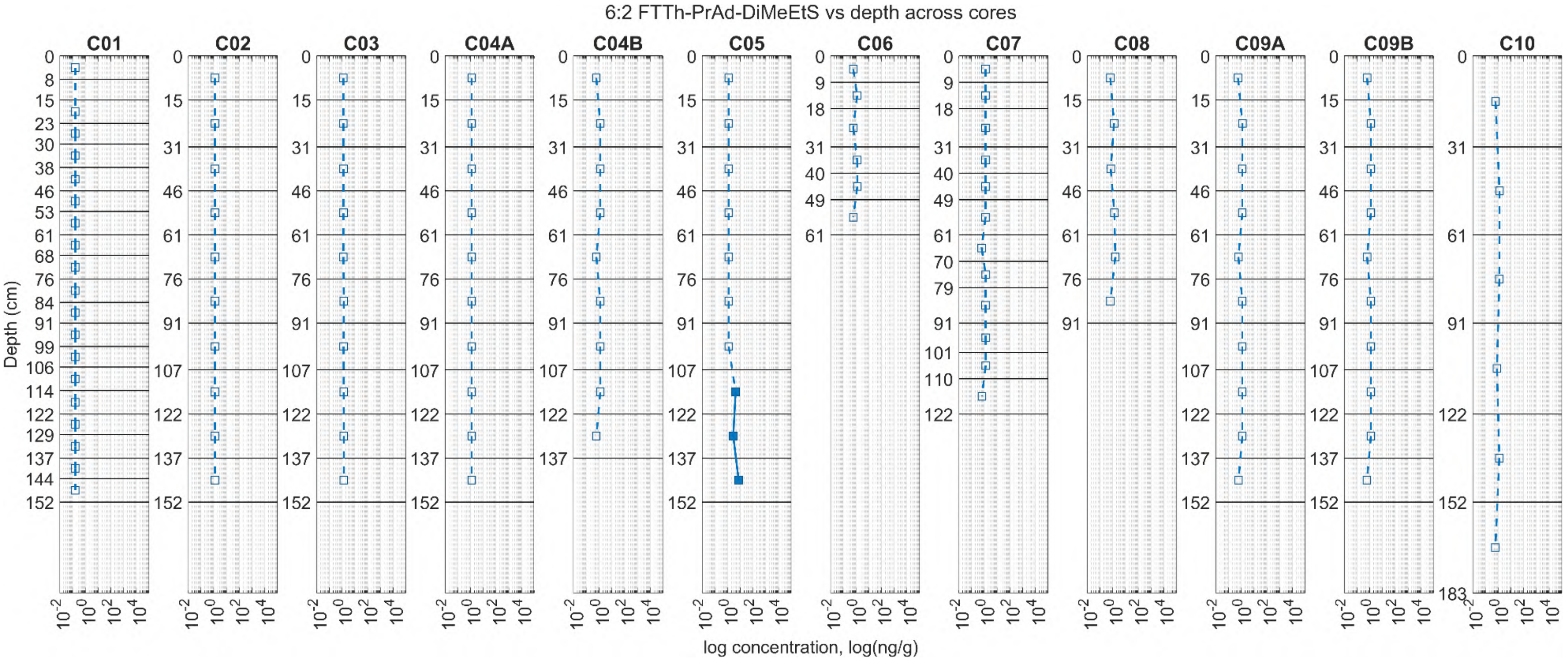

Figure S51. Vertical distribution profile of **8:2 FTTh-PrAd-DiMeEtS** across the twelve studied cores

Note that the concentration is shown on a log-base-10 scale. For any given plot and compound, open markers with dashed-line connectors represent sampled depth intervals where the compounds' concentration was below the reporting limit – the location of the open marker along the x-axis is representative of those reporting limits.

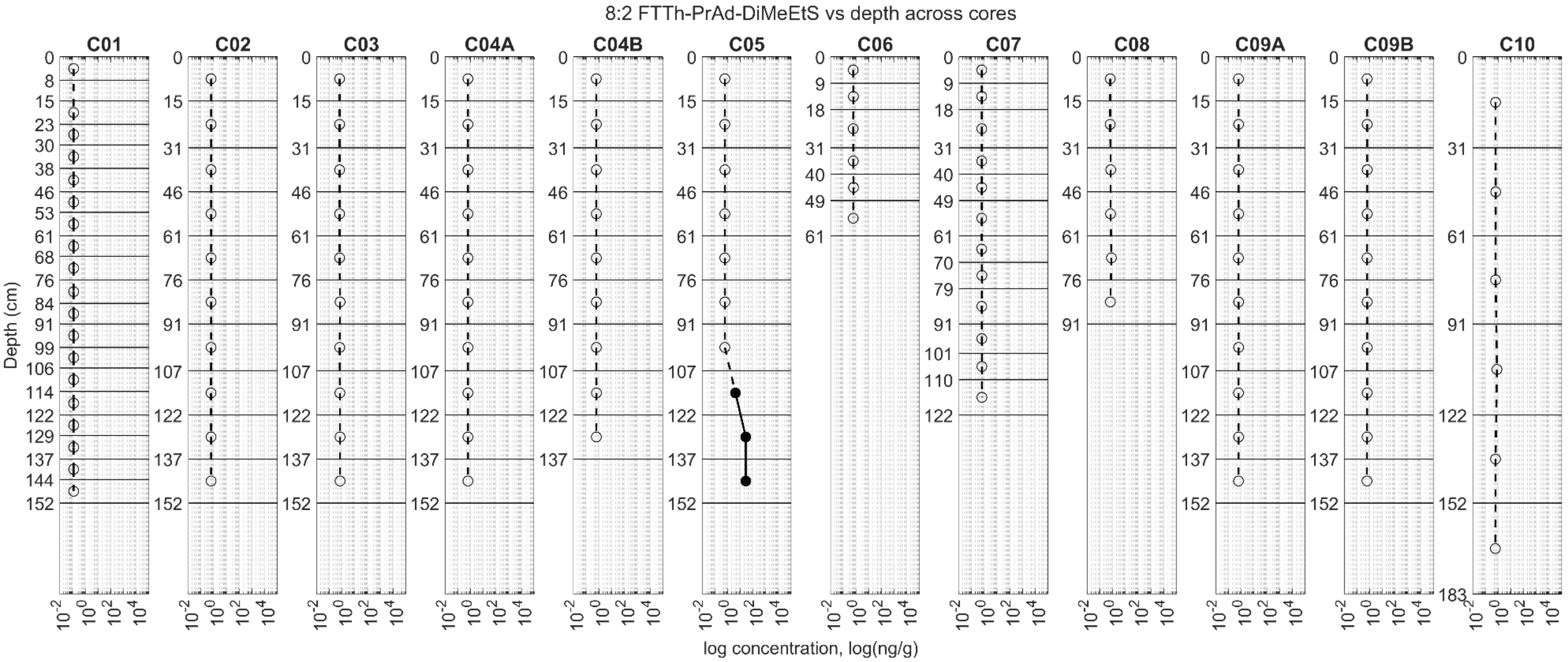

Figure S52. Vertical distribution profile of **K-PFPeS** across the twelve studied cores

Note that the concentration is shown on a log-base-10 scale. For any given plot and compound, open markers with dashed-line connectors represent sampled depth intervals where the compounds' concentration was below the reporting limit – the location of the open marker along the x-axis is representative of those reporting limits.

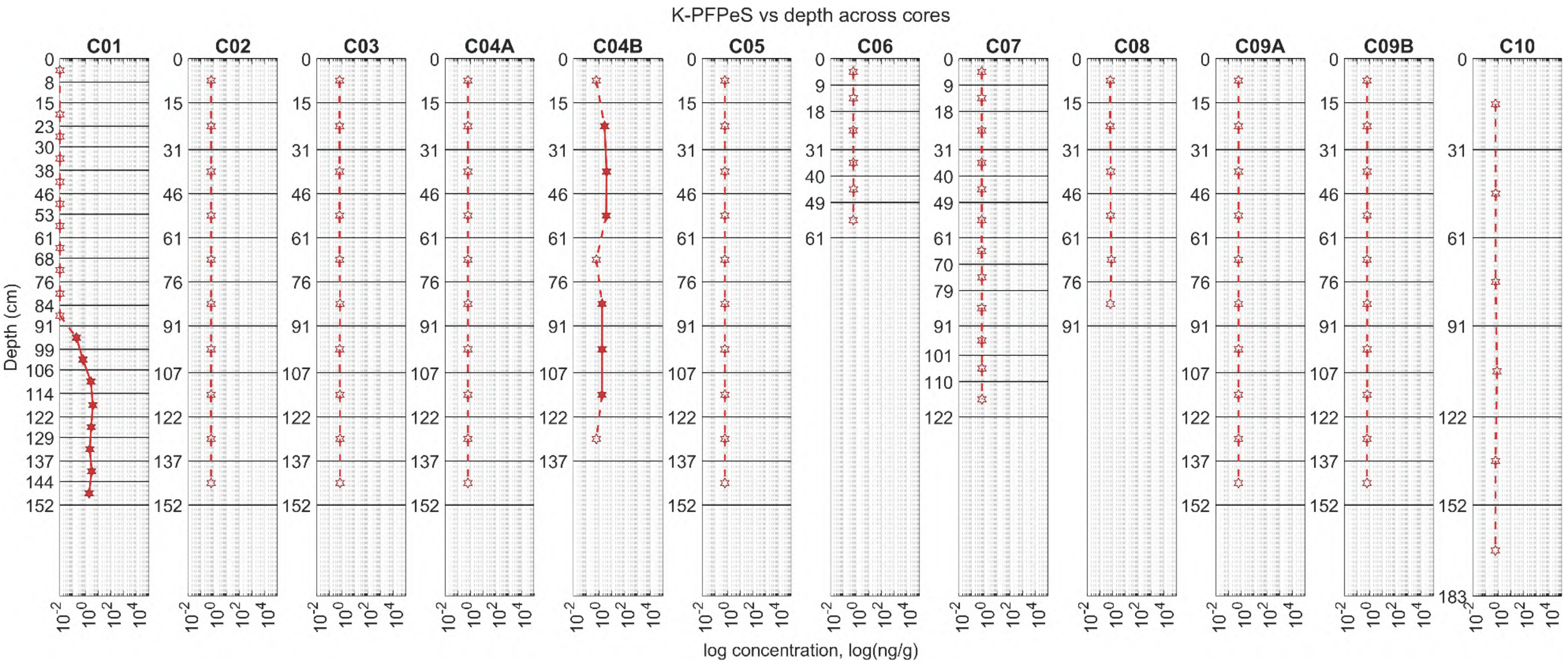

Figure S53. Vertical distribution profile of **K-PFHxS** across the twelve studied cores

Note that the concentration is shown on a log-base-10 scale. For any given plot and compound, open markers with dashed-line connectors represent sampled depth intervals where the compounds' concentration was below the reporting limit – the location of the open marker along the x-axis is representative of those reporting limits.

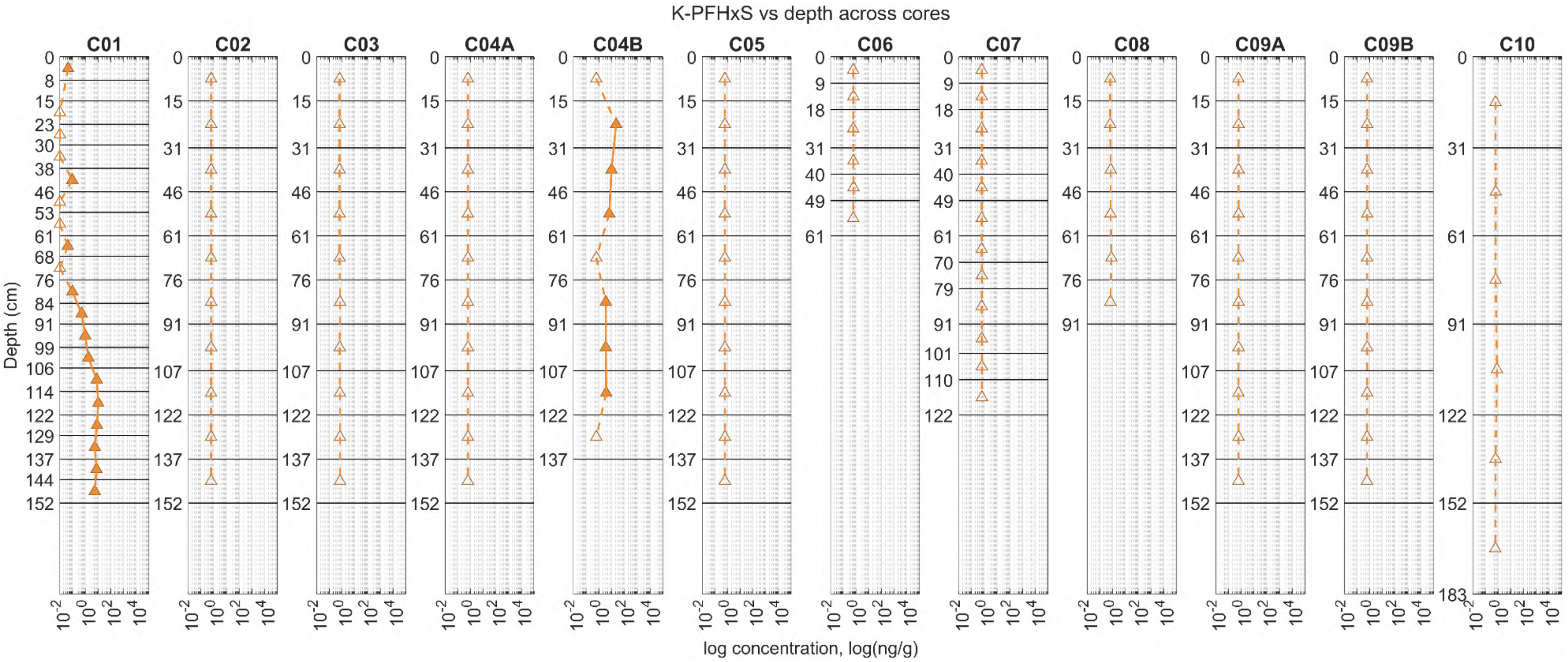

Figure S54. Vertical distribution profile of **K-PFHpS** across the twelve studied cores

Note that the concentration is shown on a log-base-10 scale. For any given plot and compound, open markers with dashed-line connectors represent sampled depth intervals where the compounds' concentration was below the reporting limit – the location of the open marker along the x-axis is representative of those reporting limits.

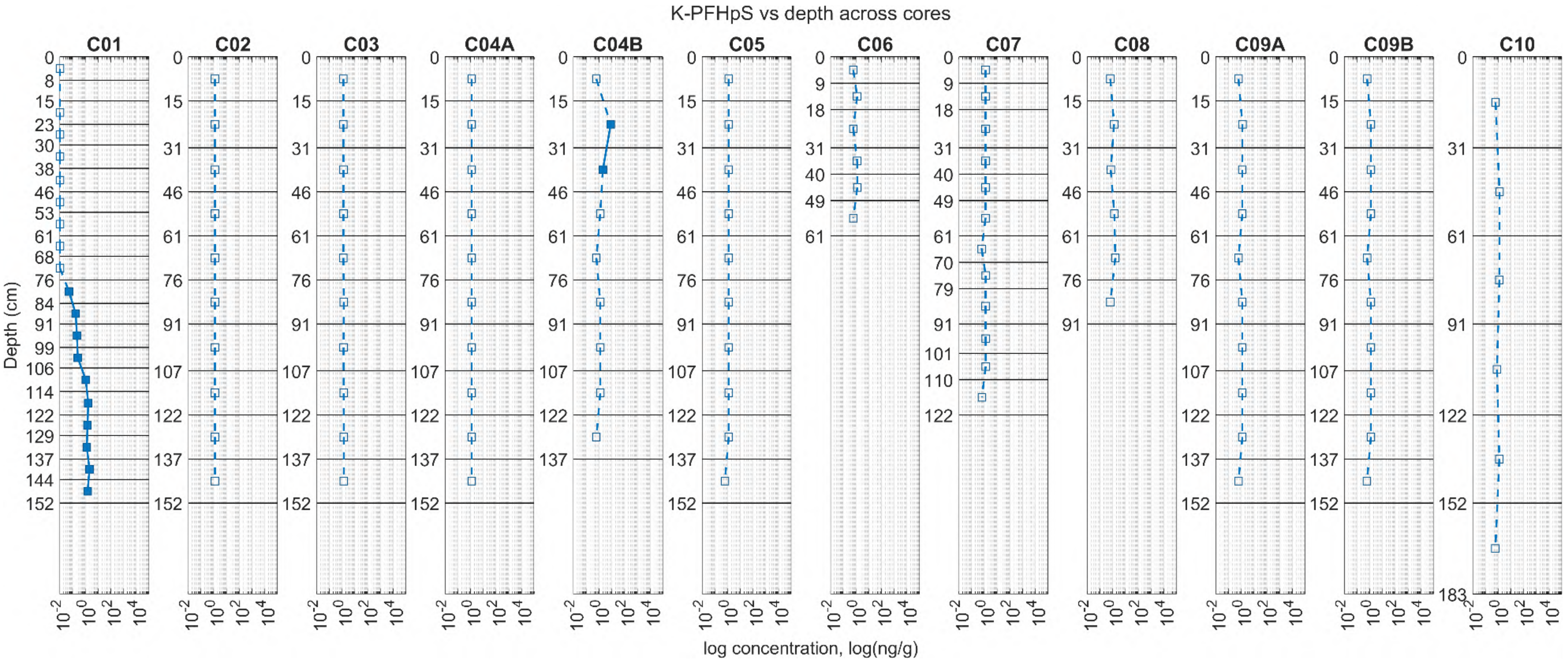

Figure S55. Vertical distribution profile of **K-PFOS** across the twelve studied cores

Note that the concentration is shown on a log-base-10 scale. For any given plot and compound, open markers with dashed-line connectors represent sampled depth intervals where the compounds' concentration was below the reporting limit – the location of the open marker along the x-axis is representative of those reporting limits.

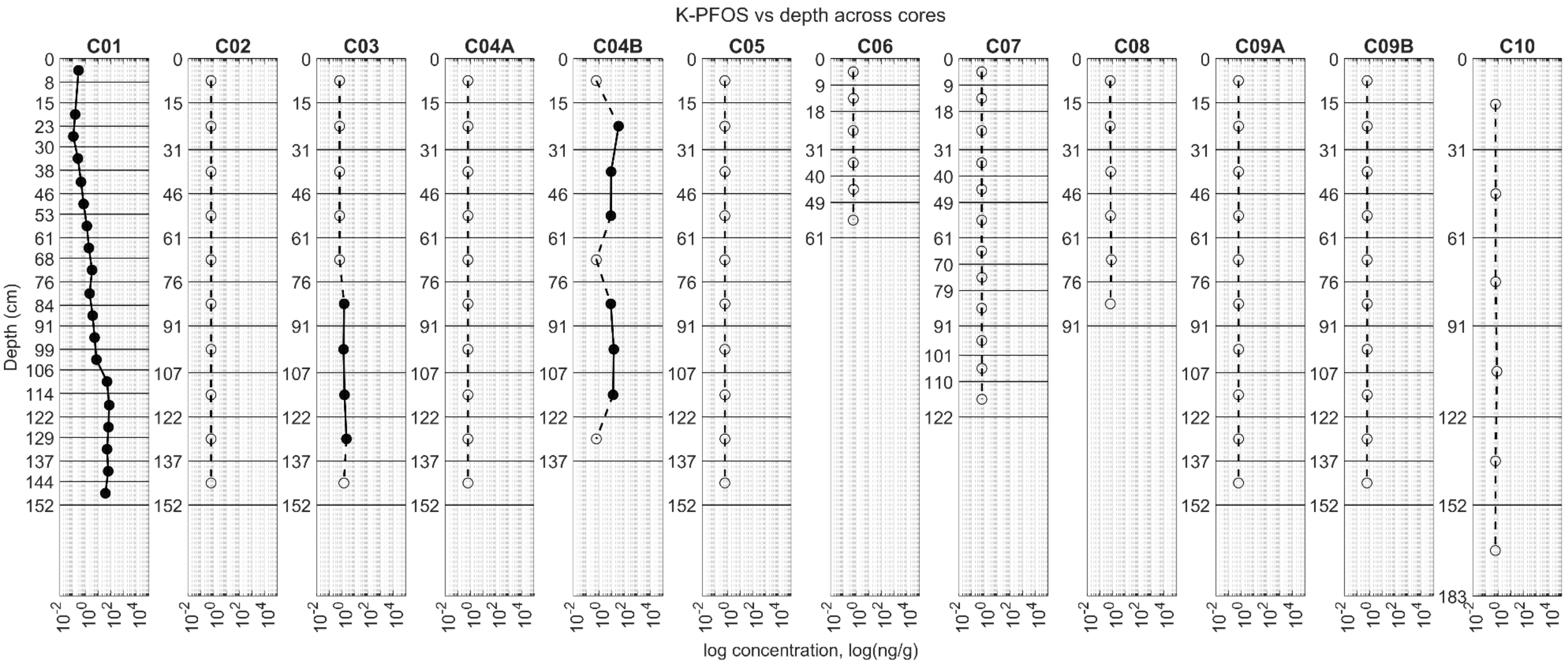

Figure S56. Vertical distribution profile of **FPrSA** across the twelve studied cores

Note that the concentration is shown on a log-base-10 scale. For any given plot and compound, open markers with dashed-line connectors represent sampled depth intervals where the compounds' concentration was below the reporting limit – the location of the open marker along the x-axis is representative of those reporting limits.

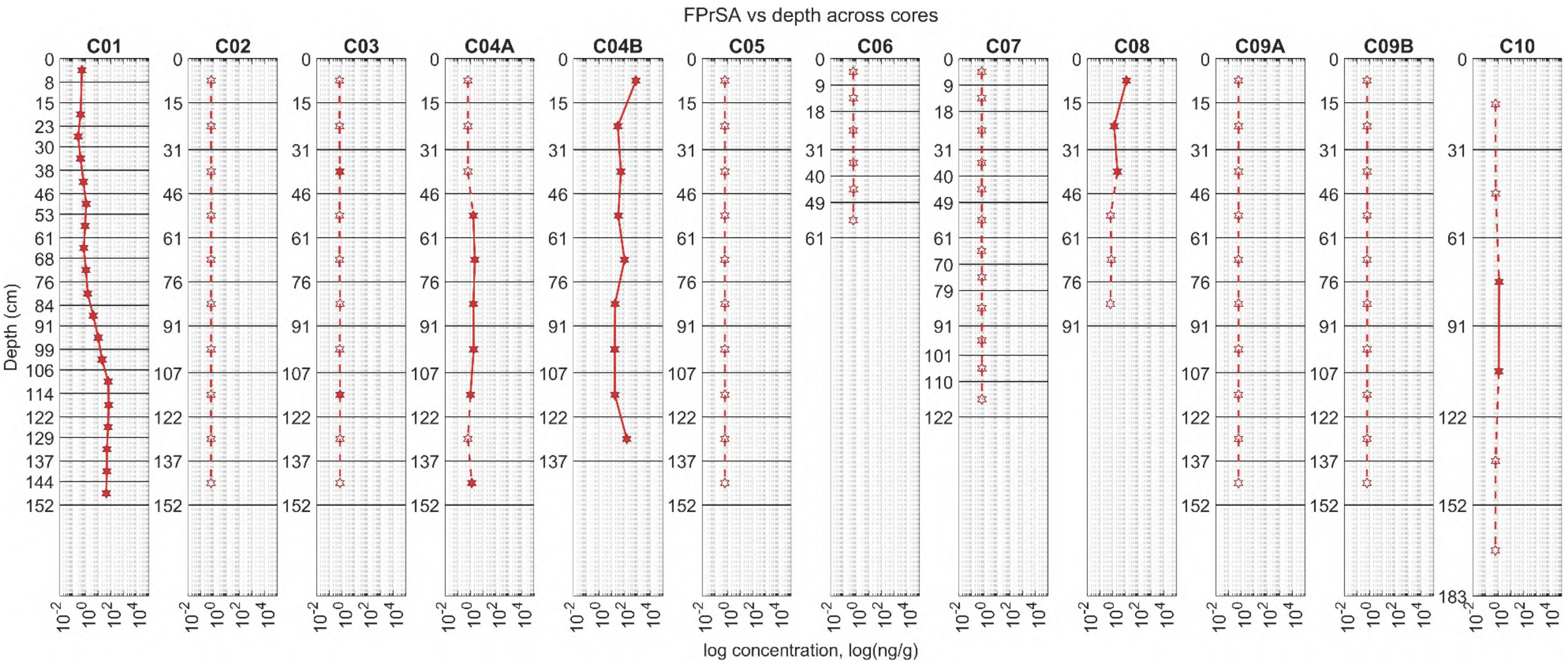

Figure S57. Vertical distribution profile of **FBSA** across the twelve studied cores

Note that the concentration is shown on a log-base-10 scale. For any given plot and compound, open markers with dashed-line connectors represent sampled depth intervals where the compounds' concentration was below the reporting limit – the location of the open marker along the x-axis is representative of those reporting limits.

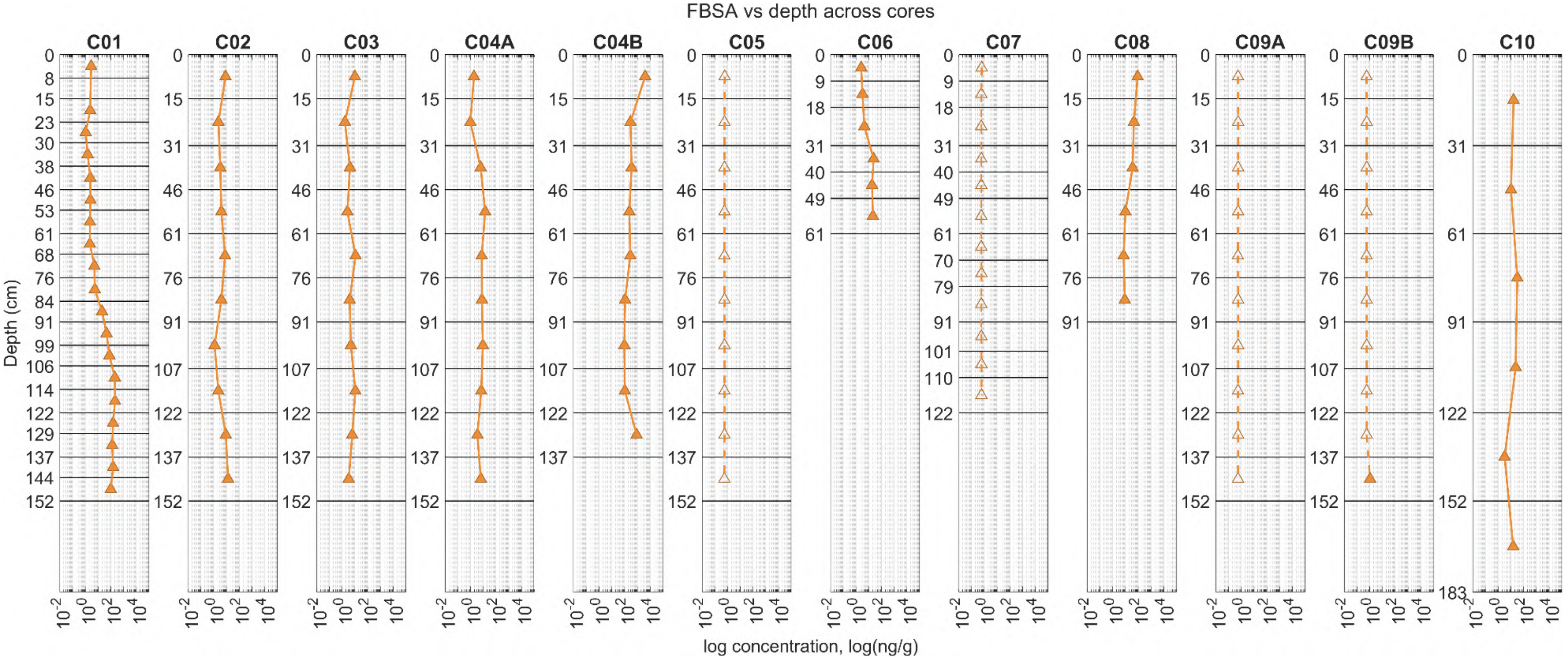

Figure S58. Vertical distribution profile of **FPeSA** across the twelve studied cores

Note that the concentration is shown on a log-base-10 scale. For any given plot and compound, open markers with dashed-line connectors represent sampled depth intervals where the compounds' concentration was below the reporting limit – the location of the open marker along the x-axis is representative of those reporting limits.

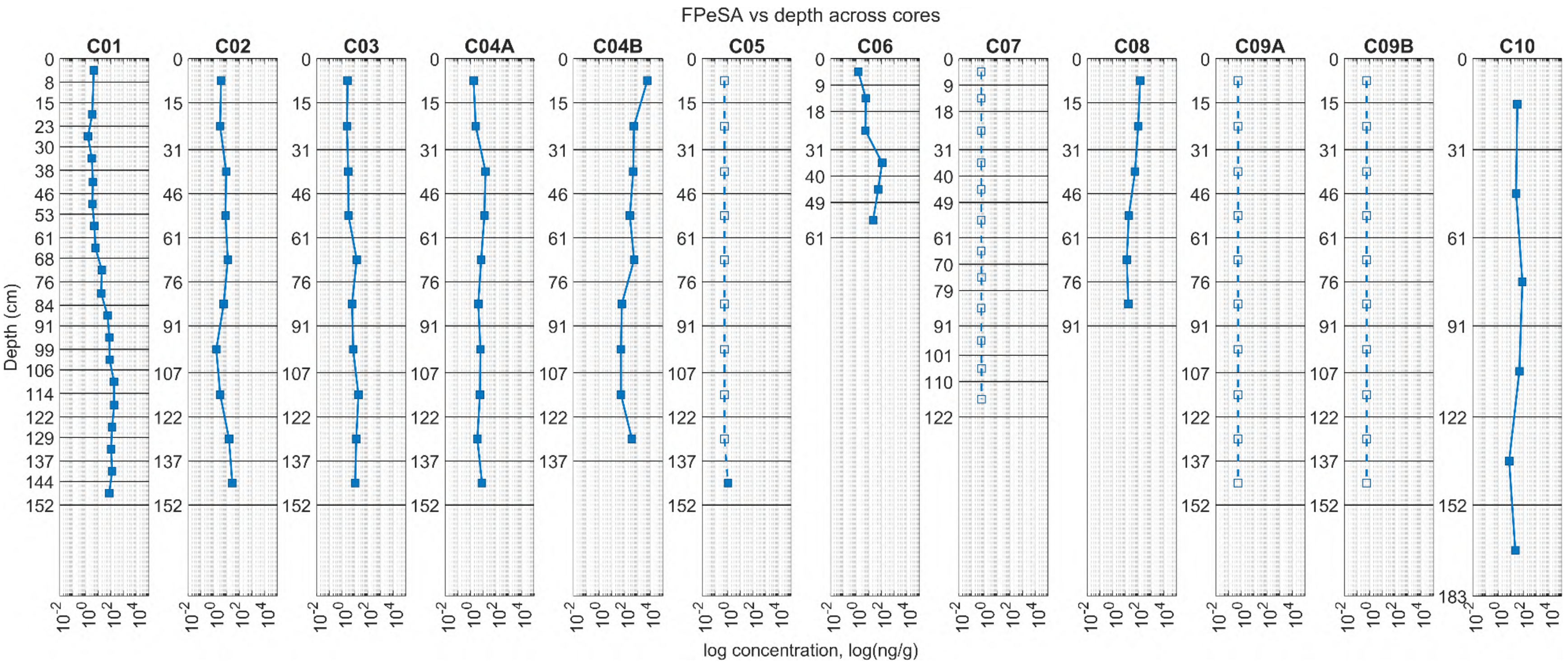

Figure S59. Vertical distribution profile of **FHxSA** across the twelve studied cores

Note that the concentration is shown on a log-base-10 scale. For any given plot and compound, open markers with dashed-line connectors represent sampled depth intervals where the compounds' concentration was below the reporting limit – the location of the open marker along the x-axis is representative of those reporting limits.

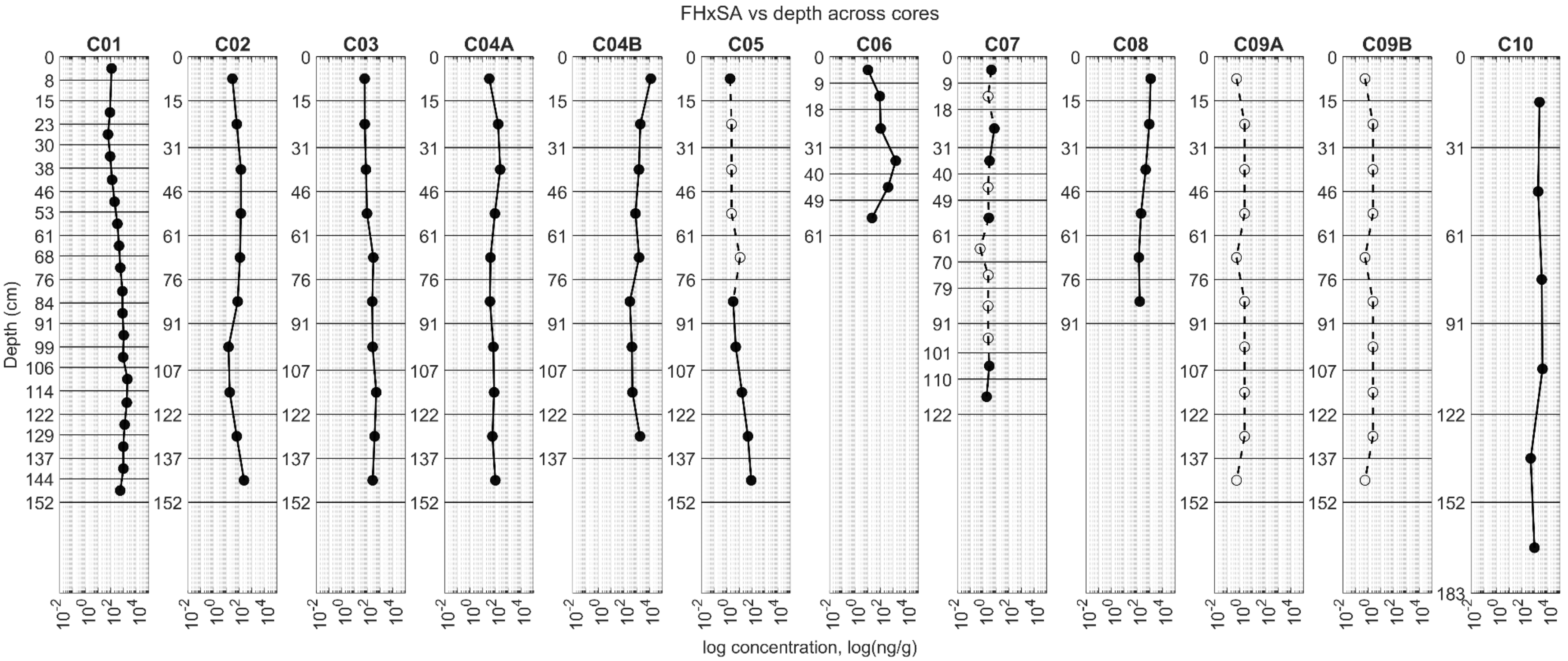

Figure S60. Vertical distribution profile of **FHpSA** across the twelve studied cores

Note that the concentration is shown on a log-base-10 scale. For any given plot and compound, open markers with dashed-line connectors represent sampled depth intervals where the compounds' concentration was below the reporting limit – the location of the open marker along the x-axis is representative of those reporting limits.

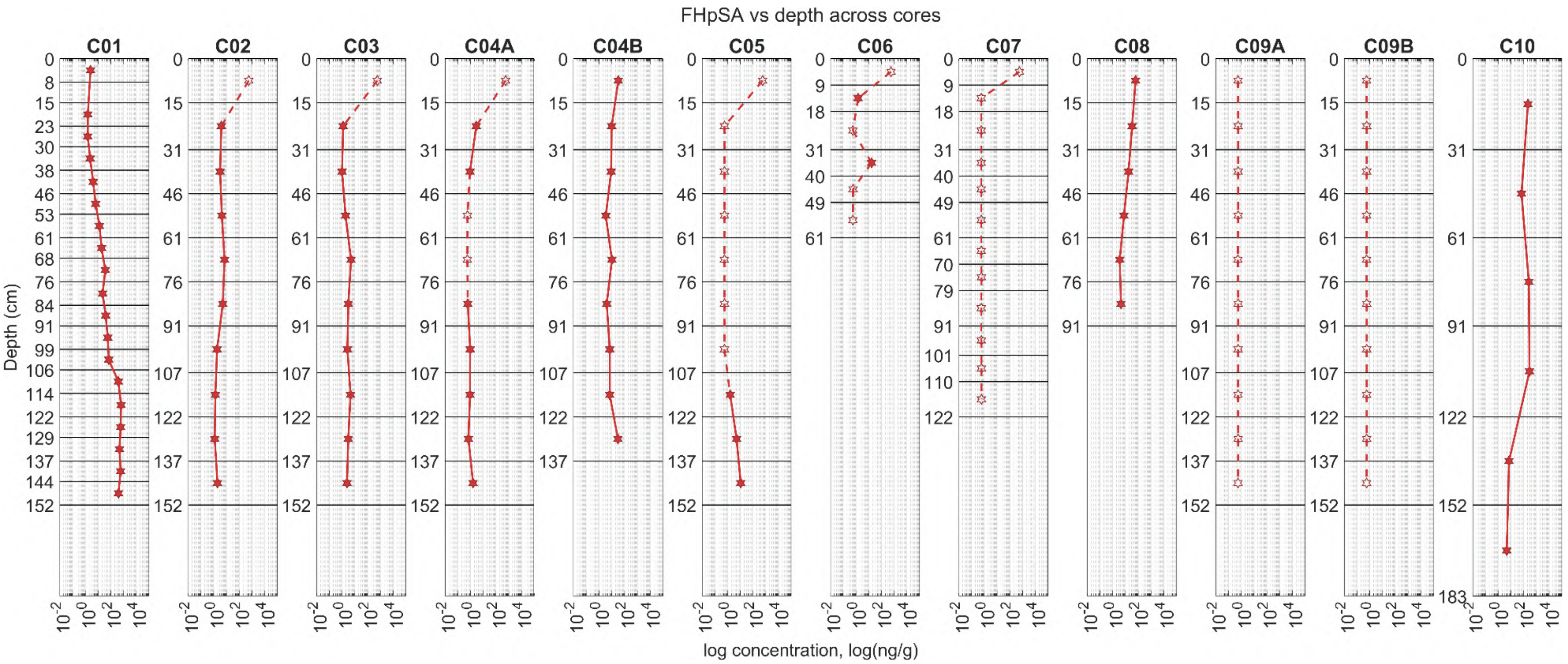

Figure S61. Vertical distribution profile of **FOSA** across the twelve studied cores

Note that the concentration is shown on a log-base-10 scale. For any given plot and compound, open markers with dashed-line connectors represent sampled depth intervals where the compounds' concentration was below the reporting limit – the location of the open marker along the x-axis is representative of those reporting limits.

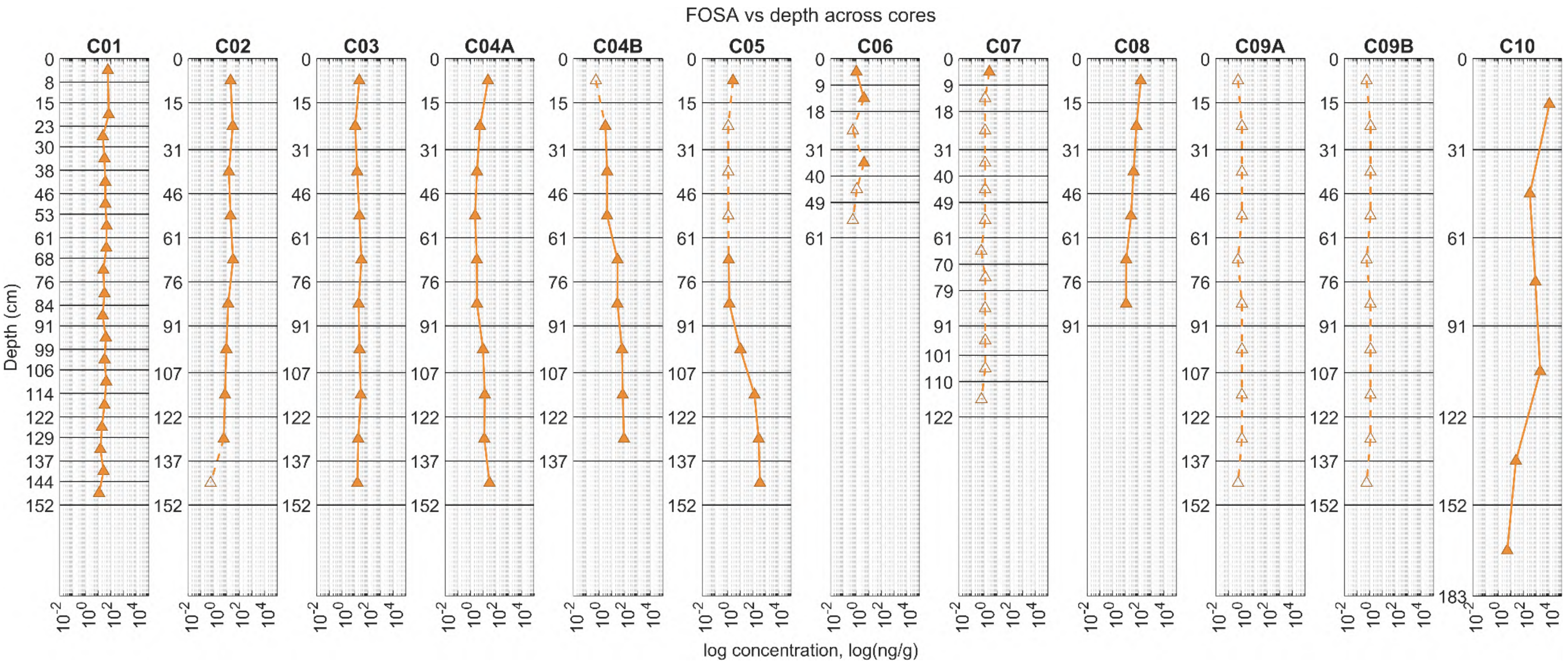

Figure S62. Vertical distribution profile of **EtFBSAA** across the twelve studied cores

Note that the concentration is shown on a log-base-10 scale. For any given plot and compound, open markers with dashed-line connectors represent sampled depth intervals where the compounds' concentration was below the reporting limit – the location of the open marker along the x-axis is representative of those reporting limits.

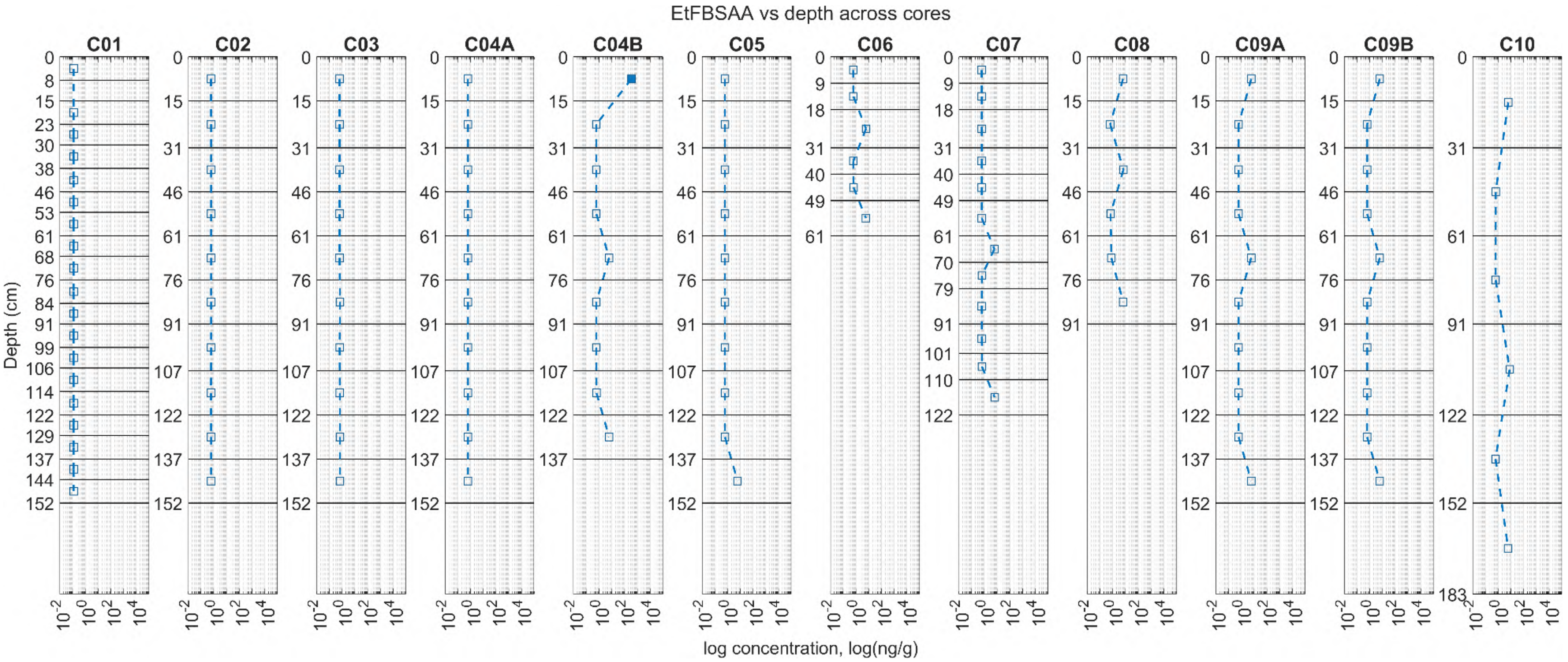

Figure S63. Vertical distribution profile of **EtFPeSAA** across the twelve studied cores

Note that the concentration is shown on a log-base-10 scale. For any given plot and compound, open markers with dashed-line connectors represent sampled depth intervals where the compounds' concentration was below the reporting limit – the location of the open marker along the x-axis is representative of those reporting limits.

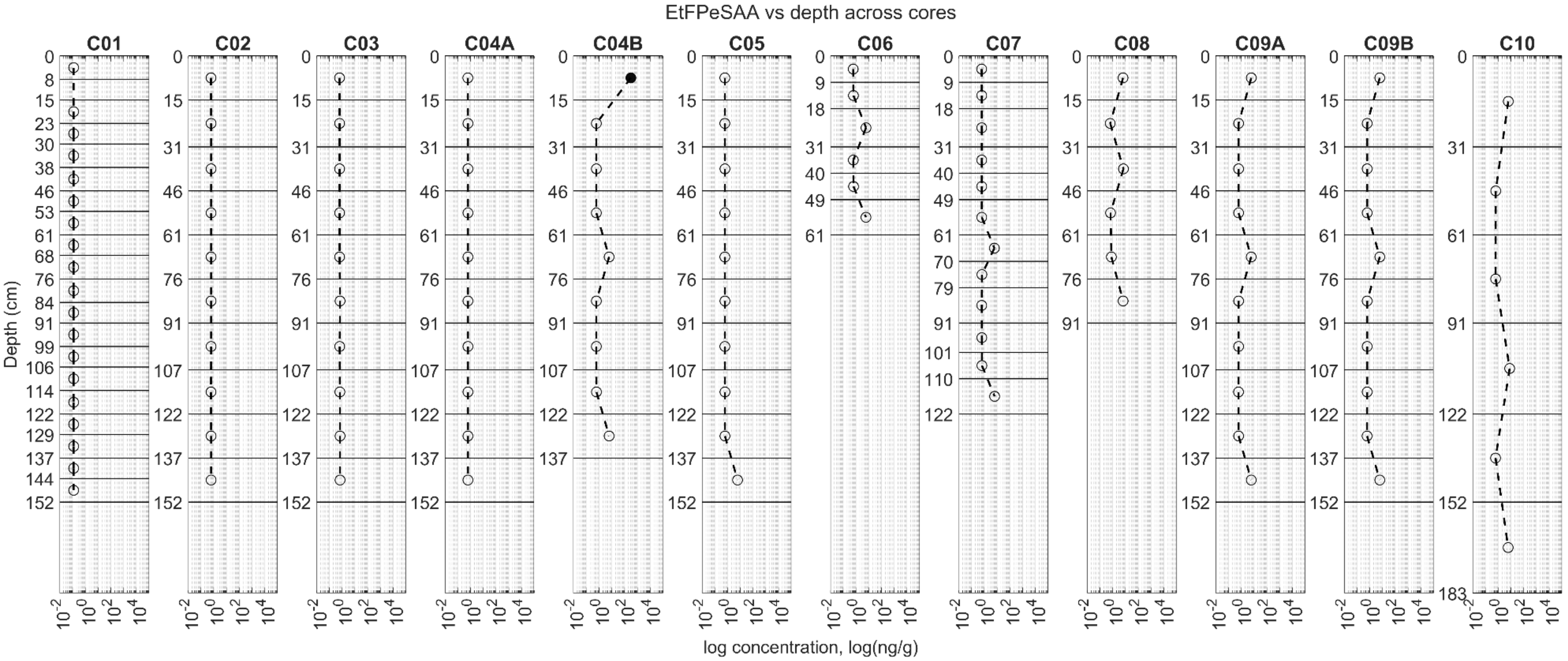

Figure S64. Vertical distribution profile of **EtFHxSAA** across the twelve studied cores

Note that the concentration is shown on a log-base-10 scale. For any given plot and compound, open markers with dashed-line connectors represent sampled depth intervals where the compounds' concentration was below the reporting limit – the location of the open marker along the x-axis is representative of those reporting limits.

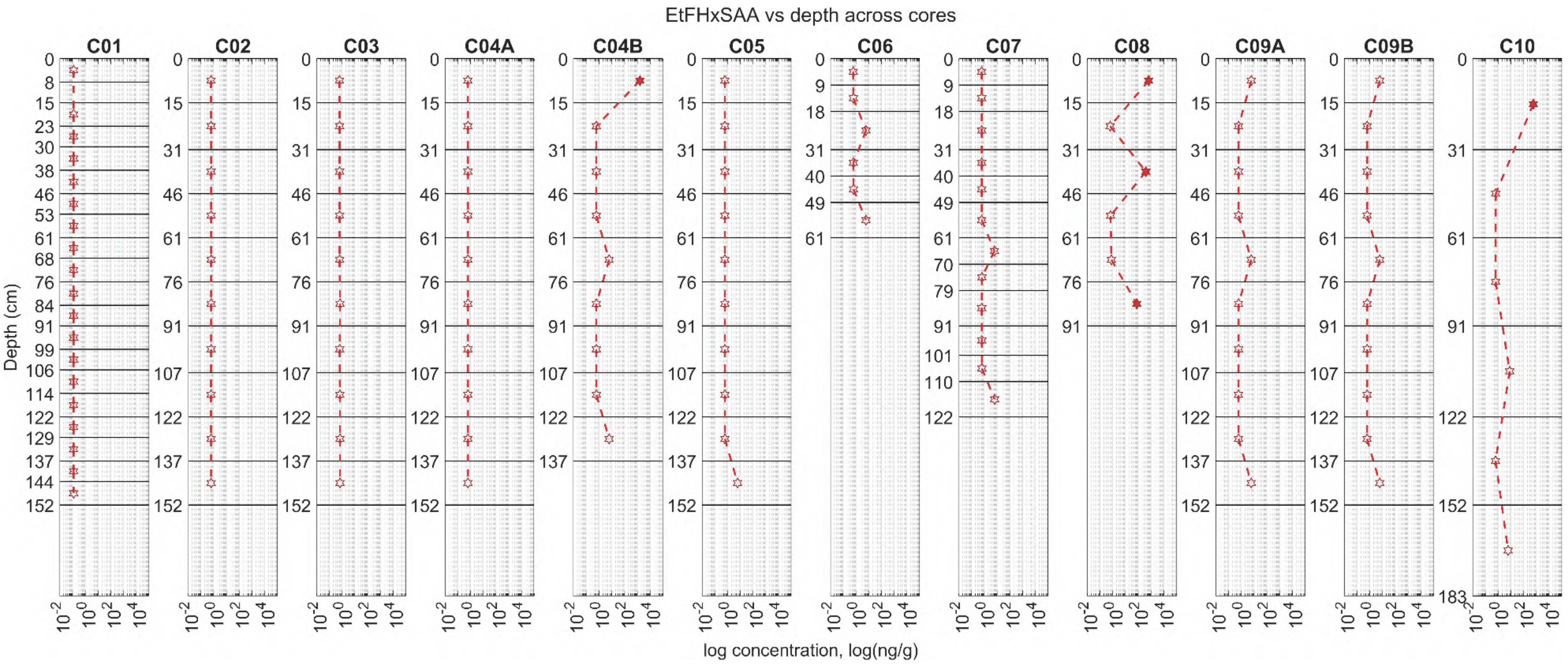

Figure S65. Vertical distribution profile of **FBSAA** across the twelve studied cores

Note that the concentration is shown on a log-base-10 scale. For any given plot and compound, open markers with dashed-line connectors represent sampled depth intervals where the compounds' concentration was below the reporting limit – the location of the open marker along the x-axis is representative of those reporting limits.

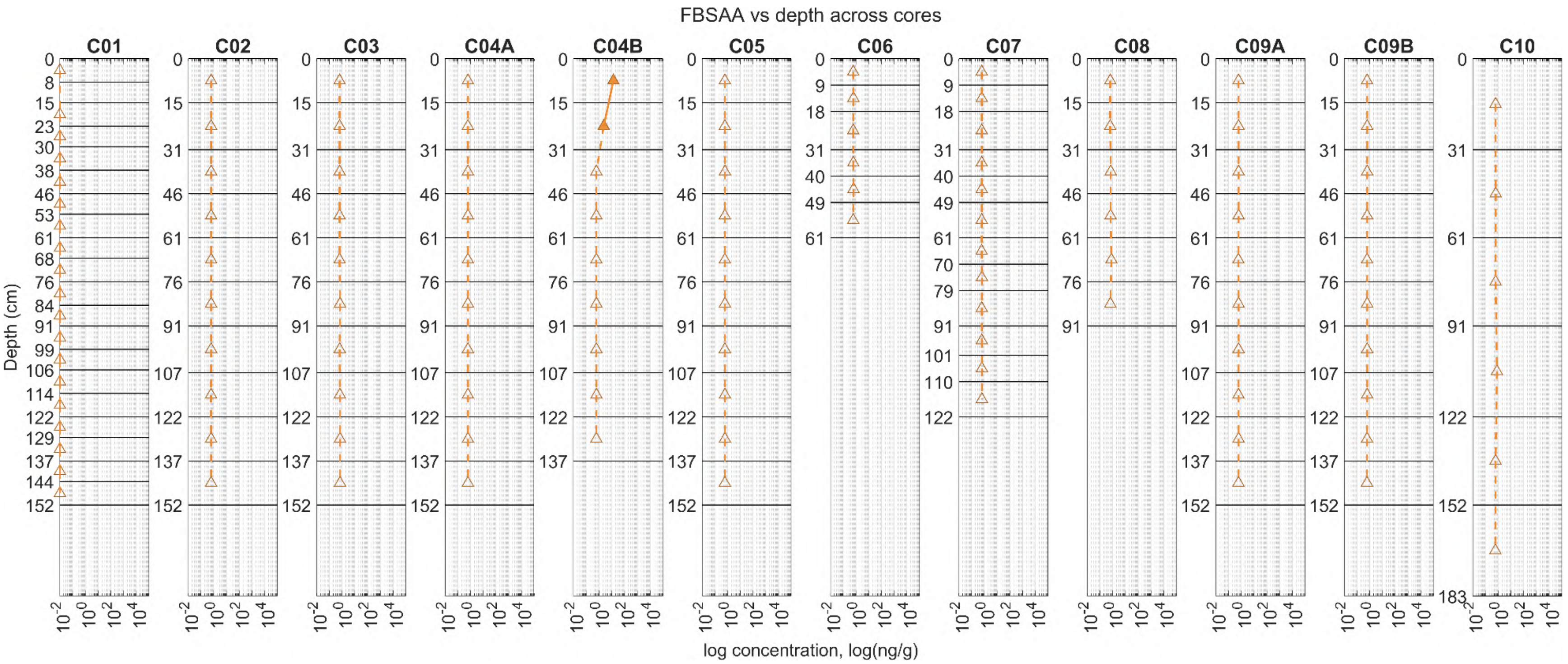

Figure S66. Vertical distribution profile of **FPeSAA** across the twelve studied cores

Note that the concentration is shown on a log-base-10 scale. For any given plot and compound, open markers with dashed-line connectors represent sampled depth intervals where the compounds' concentration was below the reporting limit – the location of the open marker along the x-axis is representative of those reporting limits.

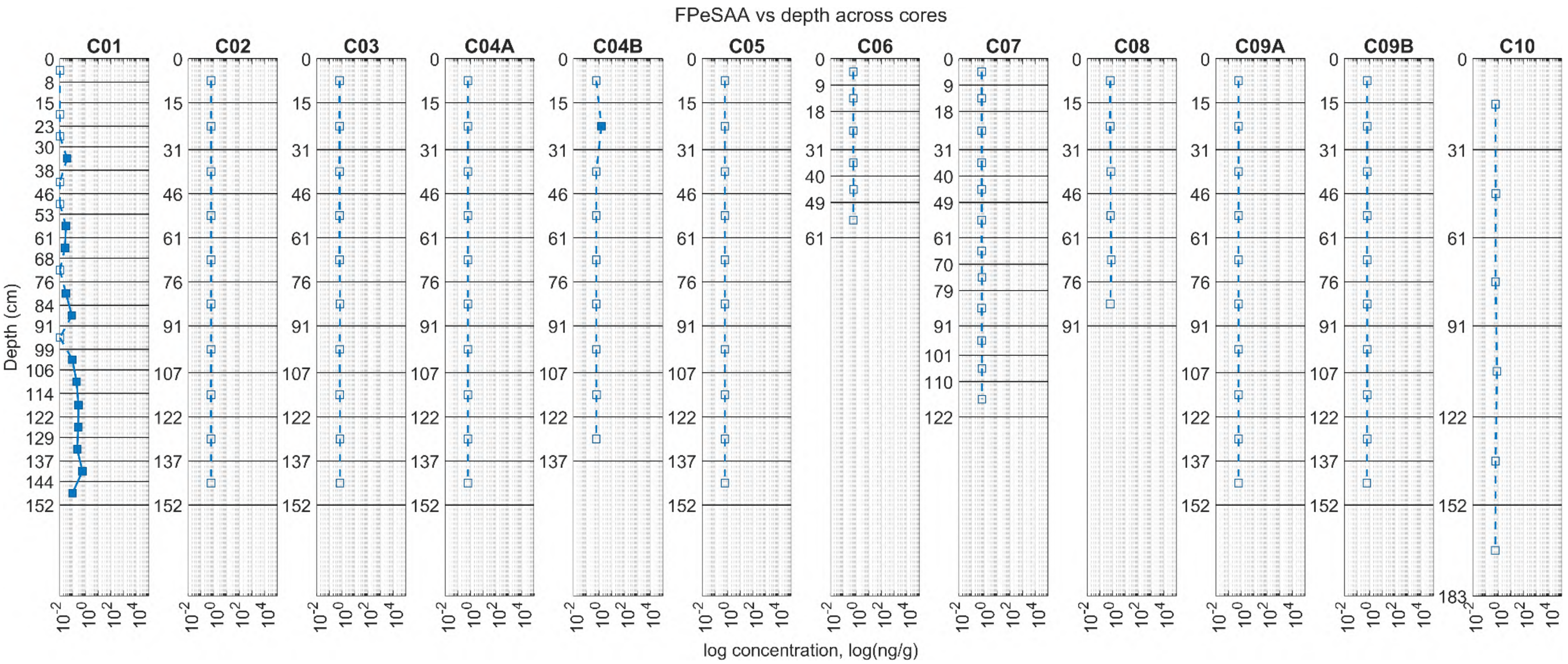

Figure S67. Vertical distribution profile of **FHxSAA** across the twelve studied cores

Note that the concentration is shown on a log-base-10 scale. For any given plot and compound, open markers with dashed-line connectors represent sampled depth intervals where the compounds' concentration was below the reporting limit – the location of the open marker along the x-axis is representative of those reporting limits.

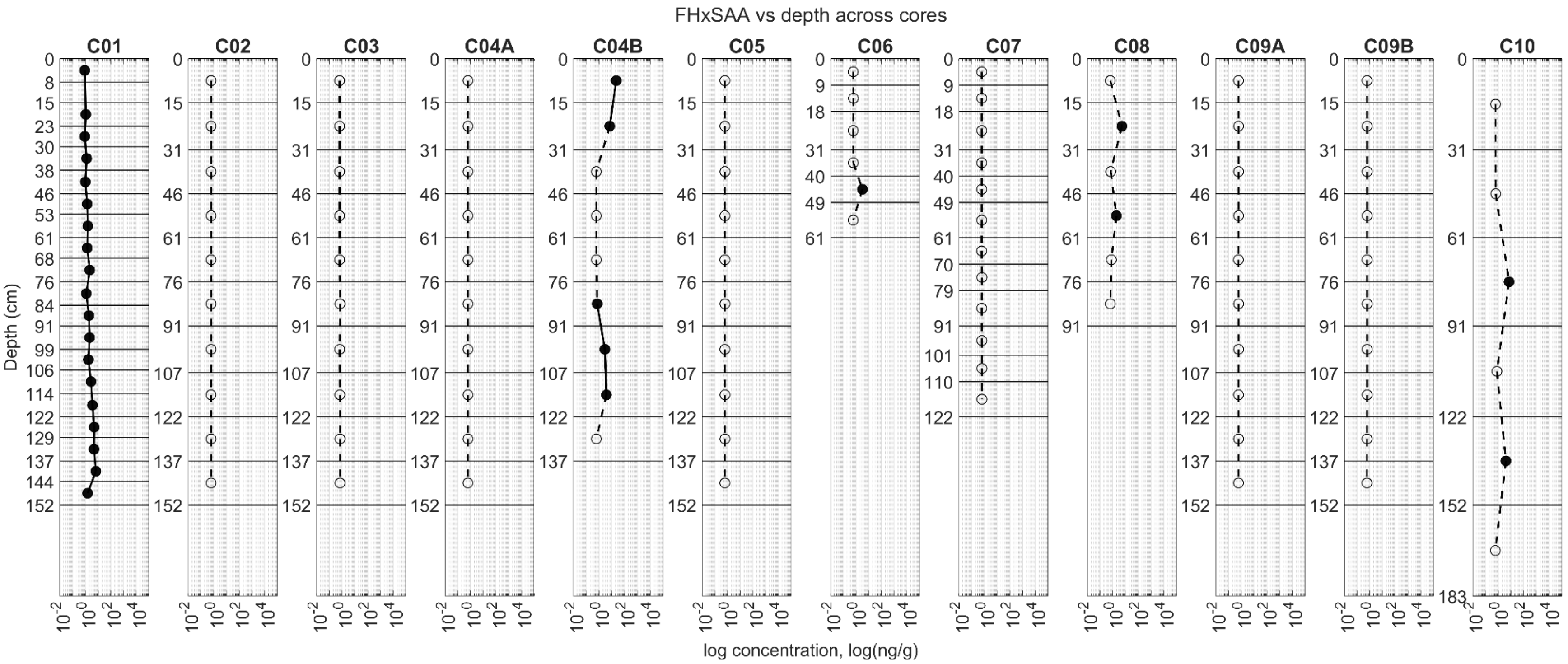

Figure S68. Vertical distribution profile of **FOSAA** across the twelve studied cores

Note that the concentration is shown on a log-base-10 scale. For any given plot and compound, open markers with dashed-line connectors represent sampled depth intervals where the compounds' concentration was below the reporting limit – the location of the open marker along the x-axis is representative of those reporting limits.

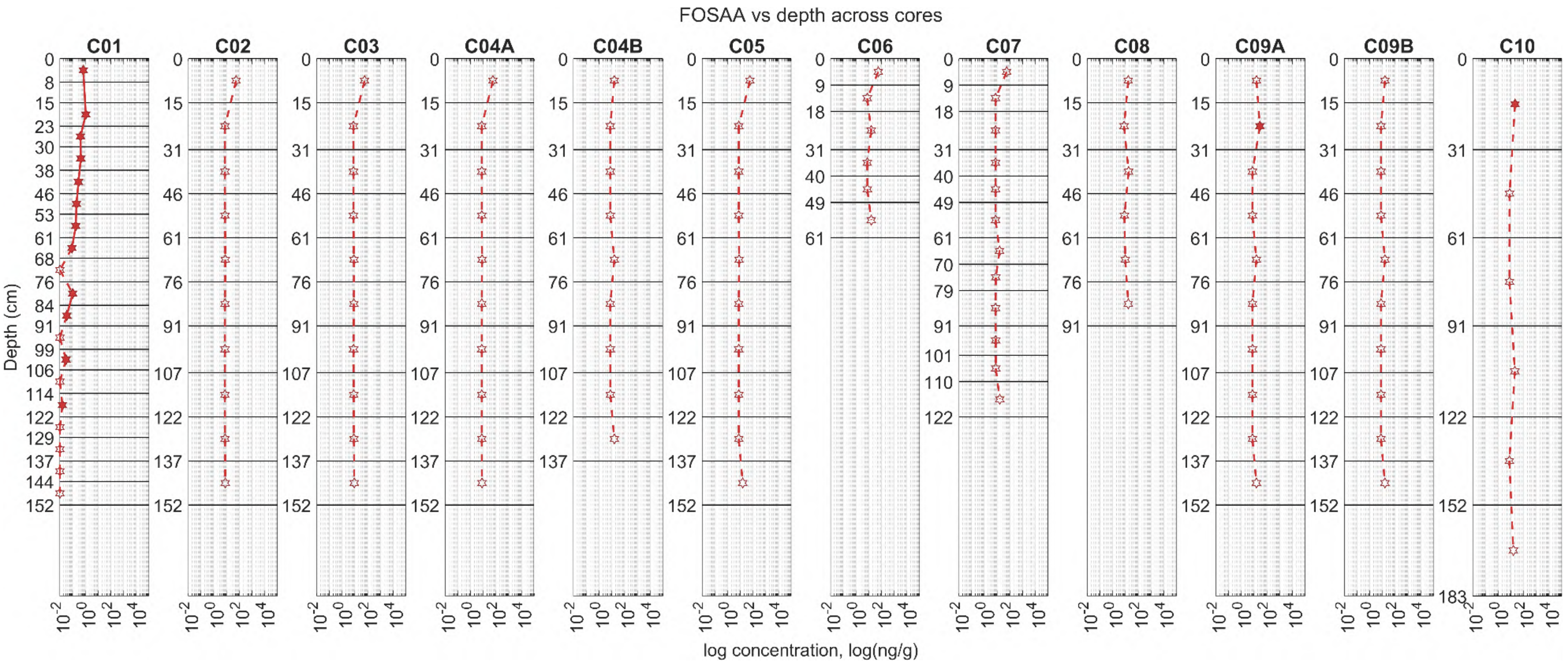

Figure S69. Vertical distribution profile of **AmPr-FEtSA-PrA** across the twelve studied cores

Note that the concentration is shown on a log-base-10 scale. For any given plot and compound, open markers with dashed-line connectors represent sampled depth intervals where the compounds' concentration was below the reporting limit – the location of the open marker along the x-axis is representative of those reporting limits.

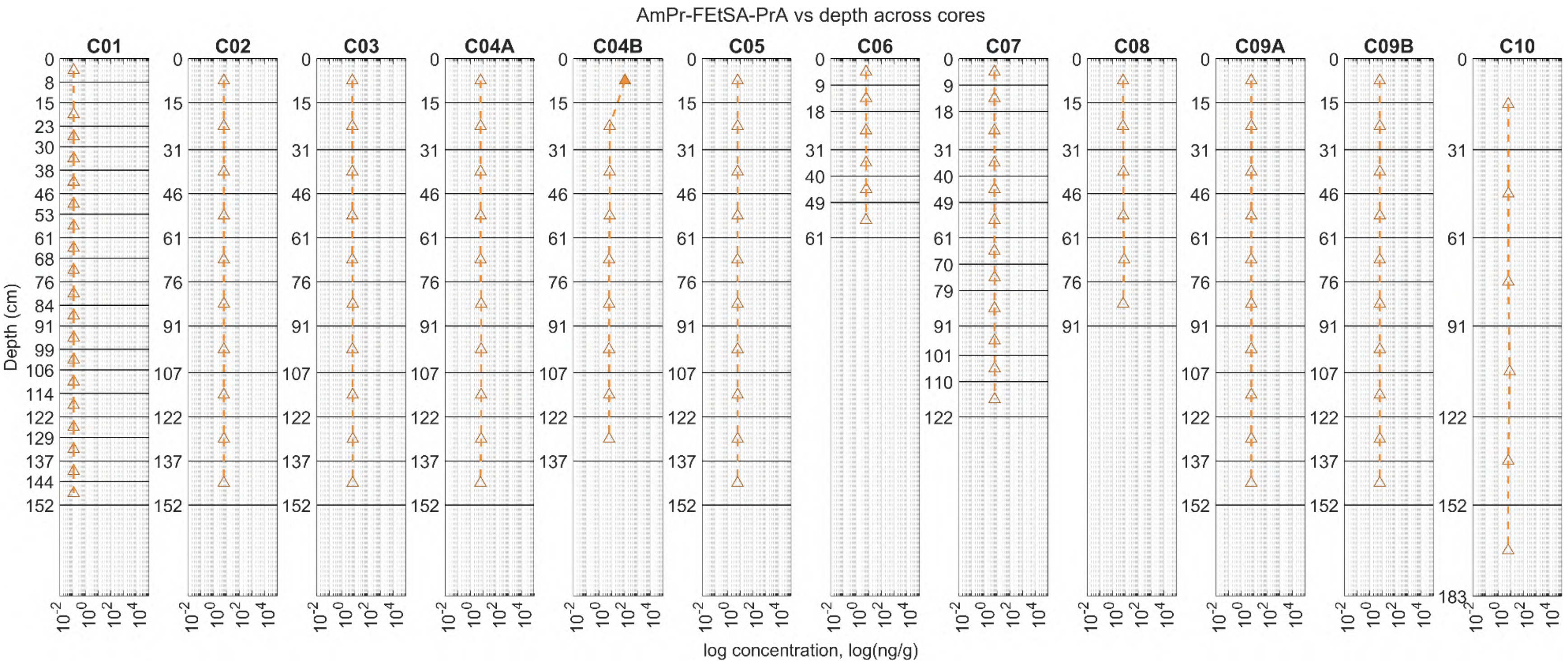

Figure S70. Vertical distribution profile of **AmPr-FPrSA-PrA** across the twelve studied cores

Note that the concentration is shown on a log-base-10 scale. For any given plot and compound, open markers with dashed-line connectors represent sampled depth intervals where the compounds' concentration was below the reporting limit – the location of the open marker along the x-axis is representative of those reporting limits.

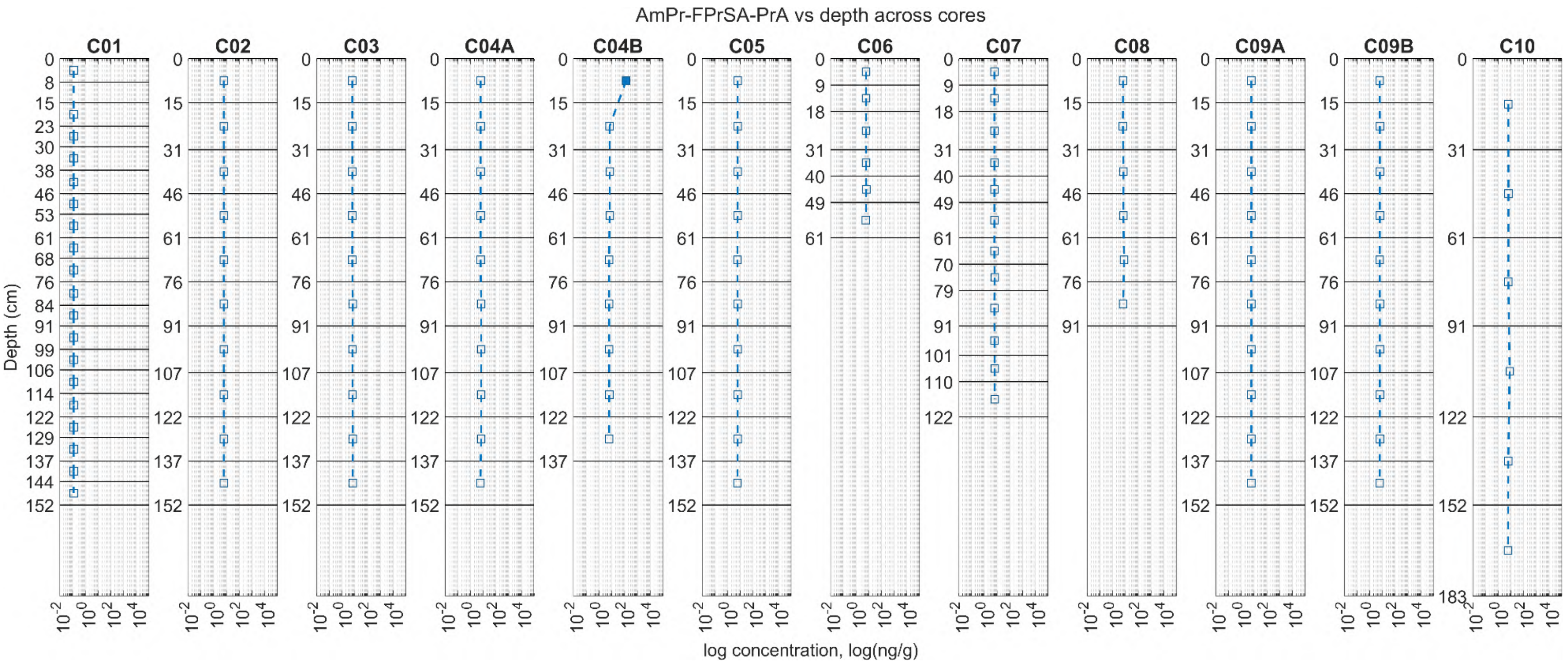

Figure S71. Vertical distribution profile of **AmPr-FBSA-PrA** across the twelve studied cores

Note that the concentration is shown on a log-base-10 scale. For any given plot and compound, open markers with dashed-line connectors represent sampled depth intervals where the compounds' concentration was below the reporting limit – the location of the open marker along the x-axis is representative of those reporting limits.

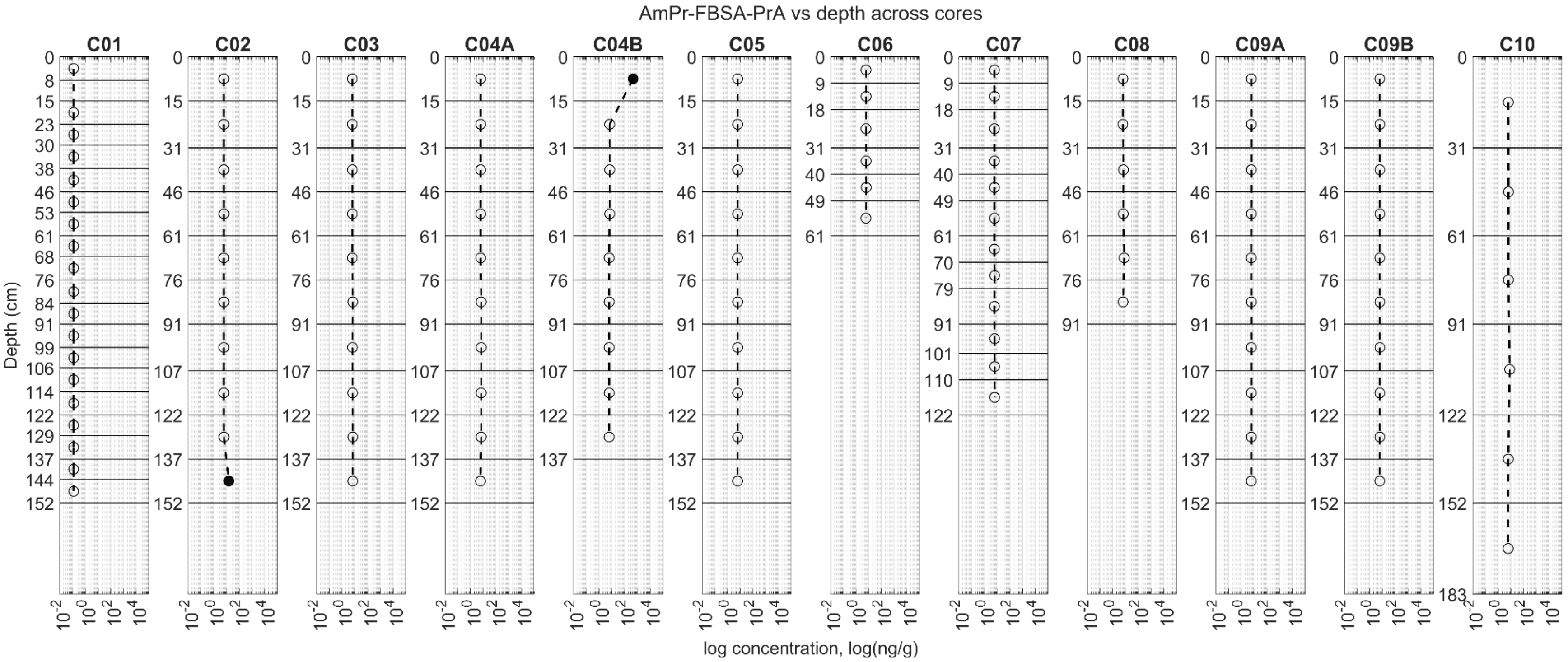

Figure S72. Vertical distribution profile of **AmPr-FHxSA-PrA** across the twelve studied cores

Note that the concentration is shown on a log-base-10 scale. For any given plot and compound, open markers with dashed-line connectors represent sampled depth intervals where the compounds' concentration was below the reporting limit – the location of the open marker along the x-axis is representative of those reporting limits.

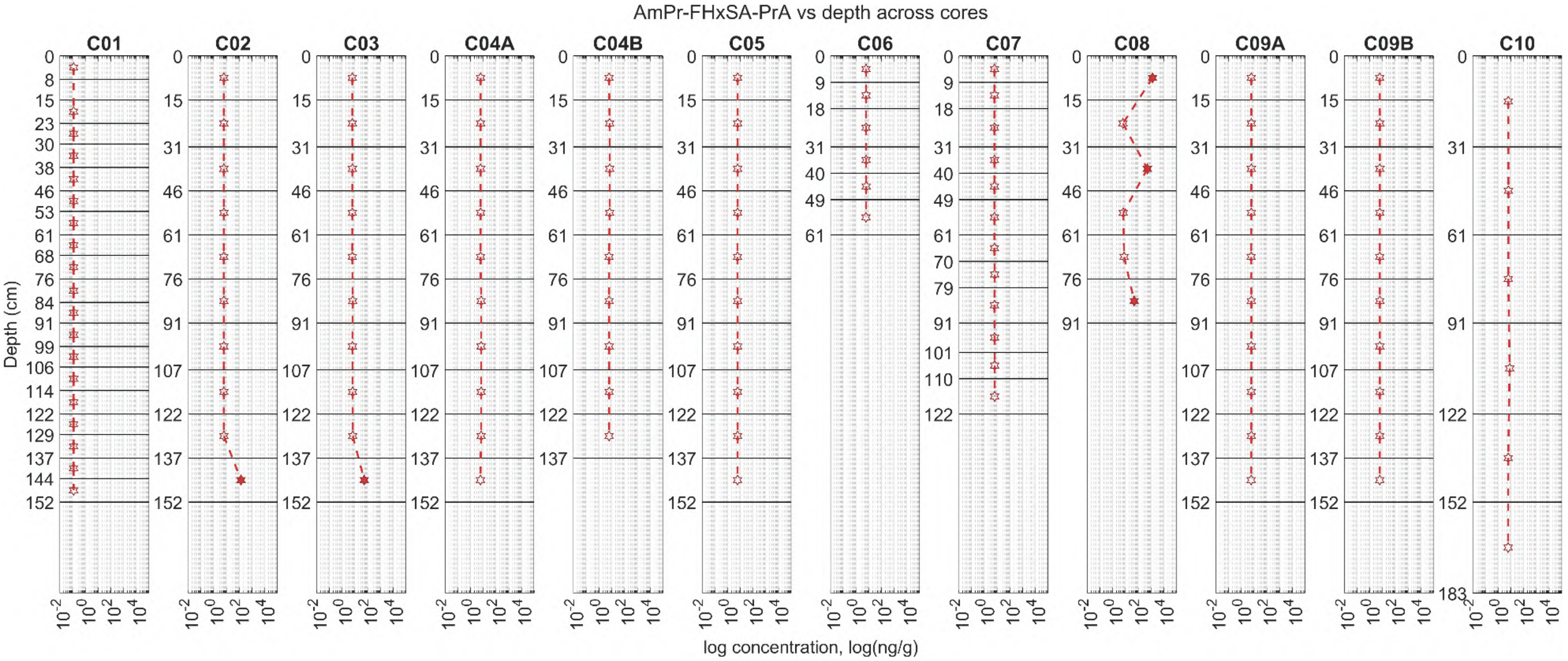

Figure S73. Vertical distribution profile of **AmPr-FPrSA** across the twelve studied cores

Note that the concentration is shown on a log-base-10 scale. For any given plot and compound, open markers with dashed-line connectors represent sampled depth intervals where the compounds' concentration was below the reporting limit – the location of the open marker along the x-axis is representative of those reporting limits.

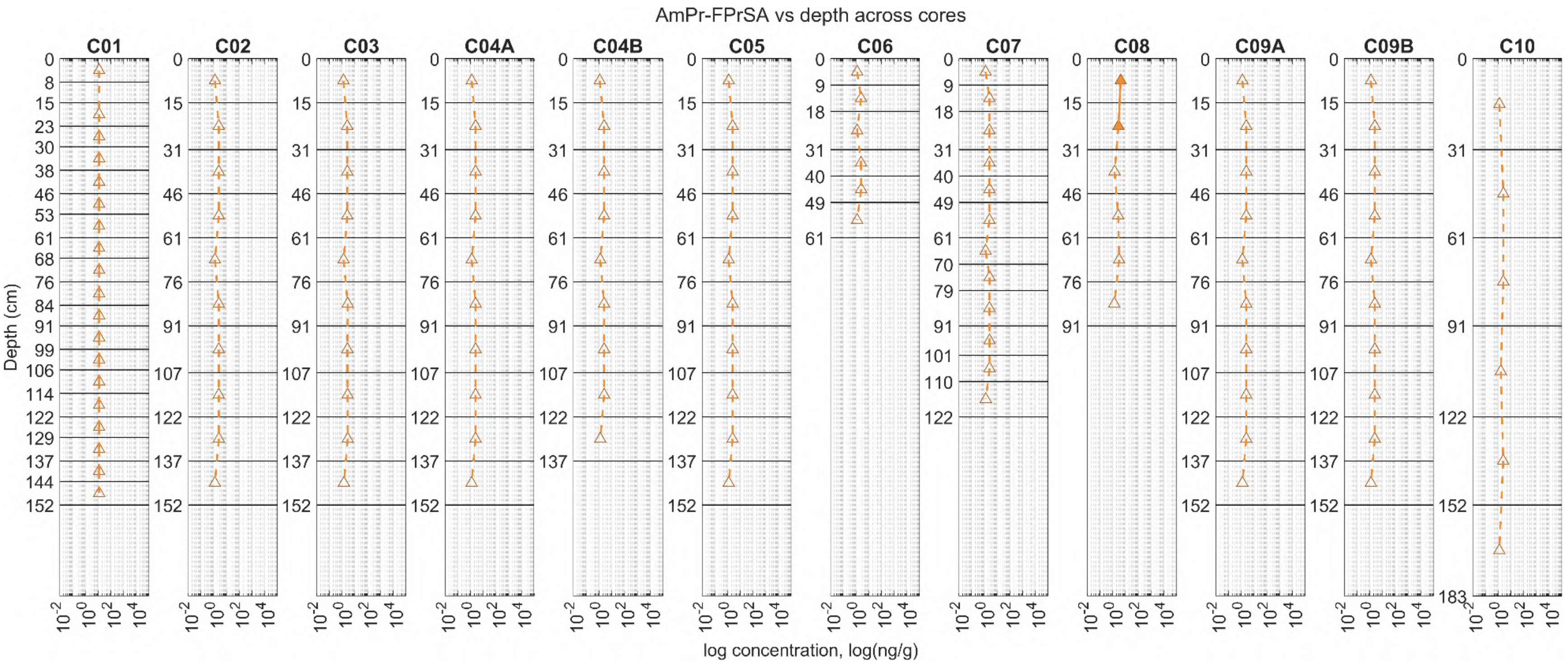

Figure S74. Vertical distribution profile of **AmPr-FBSA** across the twelve studied cores

Note that the concentration is shown on a log-base-10 scale. For any given plot and compound, open markers with dashed-line connectors represent sampled depth intervals where the compounds' concentration was below the reporting limit – the location of the open marker along the x-axis is representative of those reporting limits.

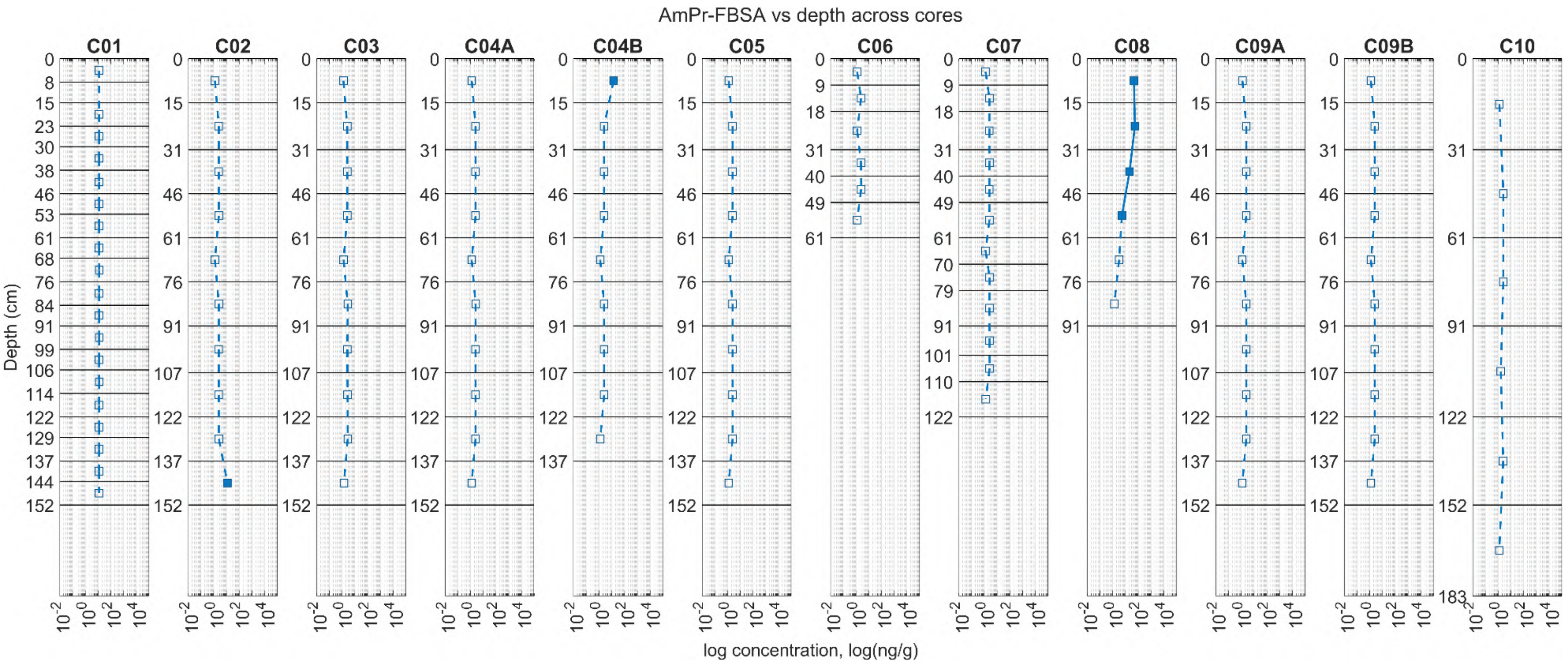

Figure S75. Vertical distribution profile of **AmPr-FPeSA** across the twelve studied cores

Note that the concentration is shown on a log-base-10 scale. For any given plot and compound, open markers with dashed-line connectors represent sampled depth intervals where the compounds' concentration was below the reporting limit – the location of the open marker along the x-axis is representative of those reporting limits.

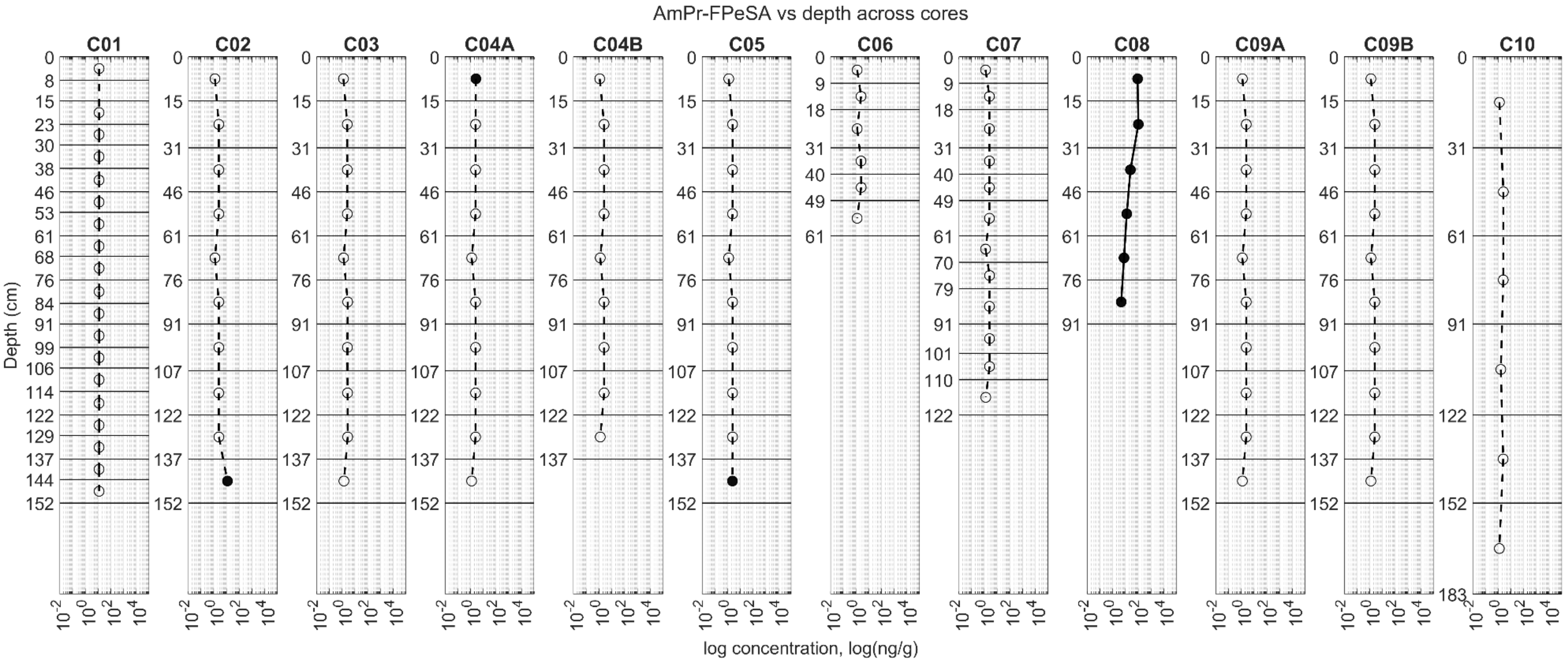

Figure S76. Vertical distribution profile of **AmPr-FHxSA** across the twelve studied cores

Note that the concentration is shown on a log-base-10 scale. For any given plot and compound, open markers with dashed-line connectors represent sampled depth intervals where the compounds' concentration was below the reporting limit – the location of the open marker along the x-axis is representative of those reporting limits.

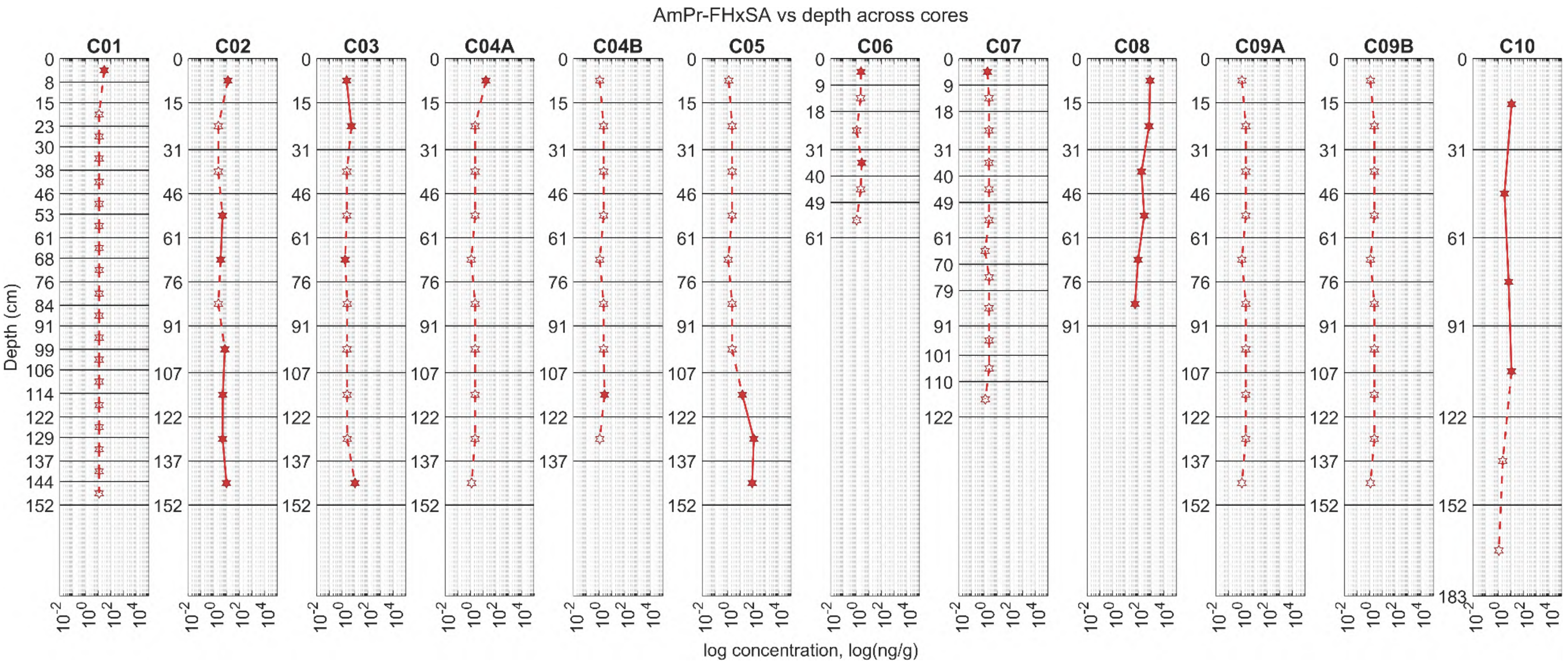

Figure S77. Vertical distribution profile of **AmPr-FHpSA** across the twelve studied cores

Note that the concentration is shown on a log-base-10 scale. For any given plot and compound, open markers with dashed-line connectors represent sampled depth intervals where the compounds' concentration was below the reporting limit – the location of the open marker along the x-axis is representative of those reporting limits.

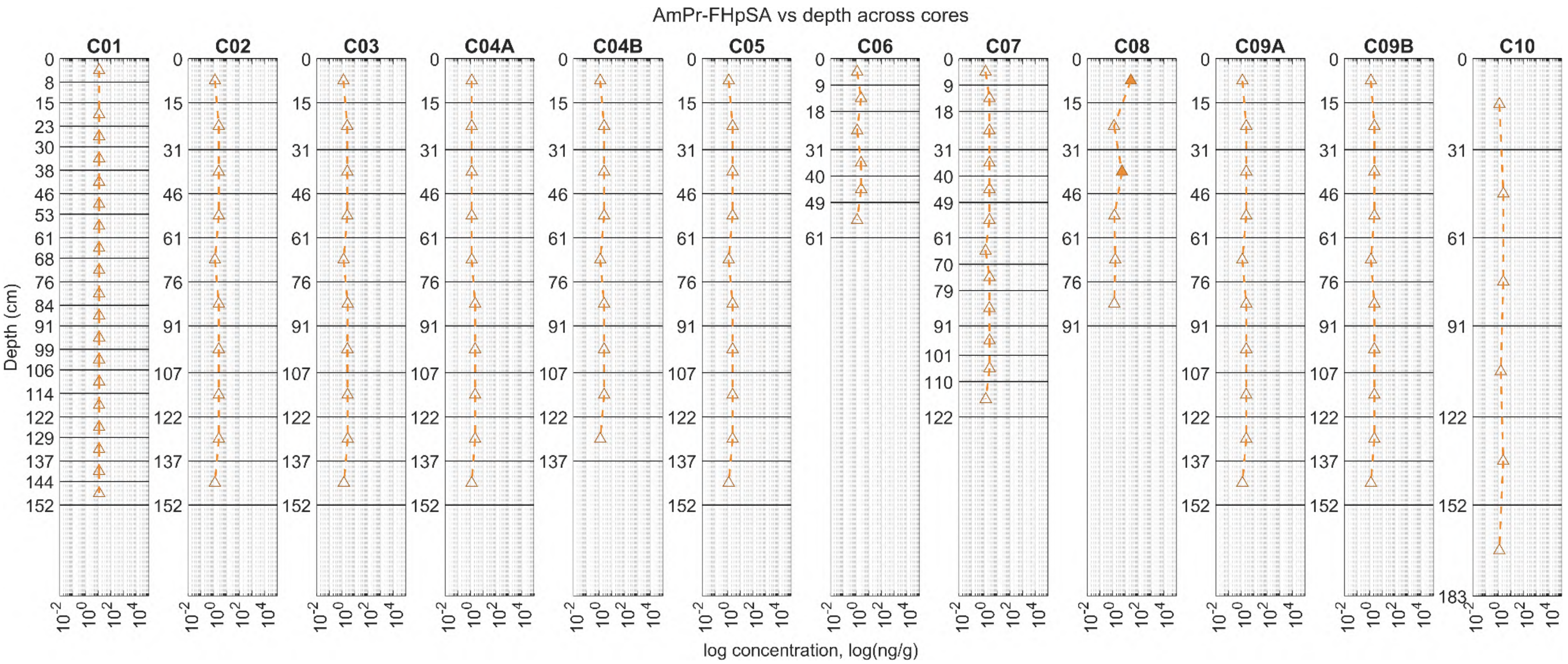

Figure S78. Vertical distribution profile of **AmPr-FOSA** across the twelve studied cores

Note that the concentration is shown on a log-base-10 scale. For any given plot and compound, open markers with dashed-line connectors represent sampled depth intervals where the compounds' concentration was below the reporting limit – the location of the open marker along the x-axis is representative of those reporting limits.

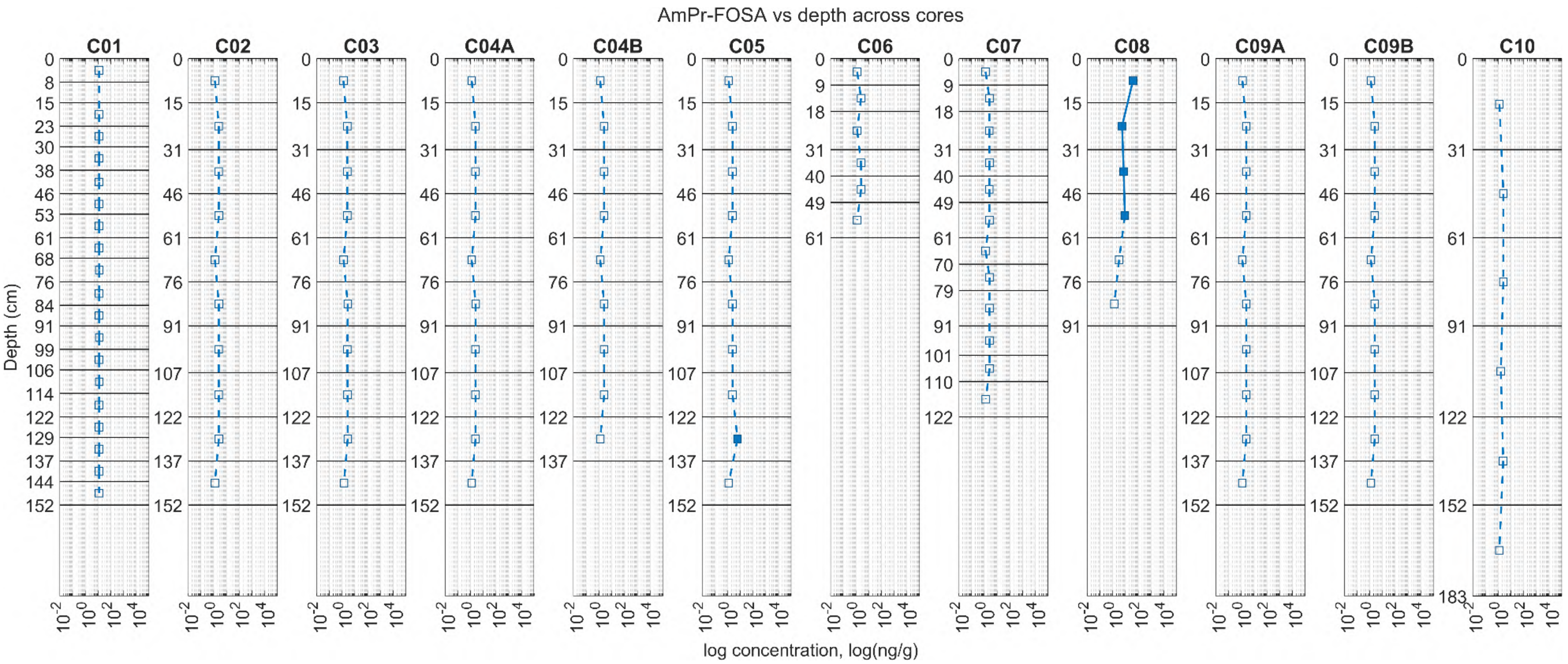

Figure S79. Vertical distribution profile of **UPFHxS** across the twelve studied cores

Note that the concentration is shown on a log-base-10 scale. For any given plot and compound, open markers with dashed-line connectors represent sampled depth intervals where the compounds' concentration was below the reporting limit – the location of the open marker along the x-axis is representative of those reporting limits.

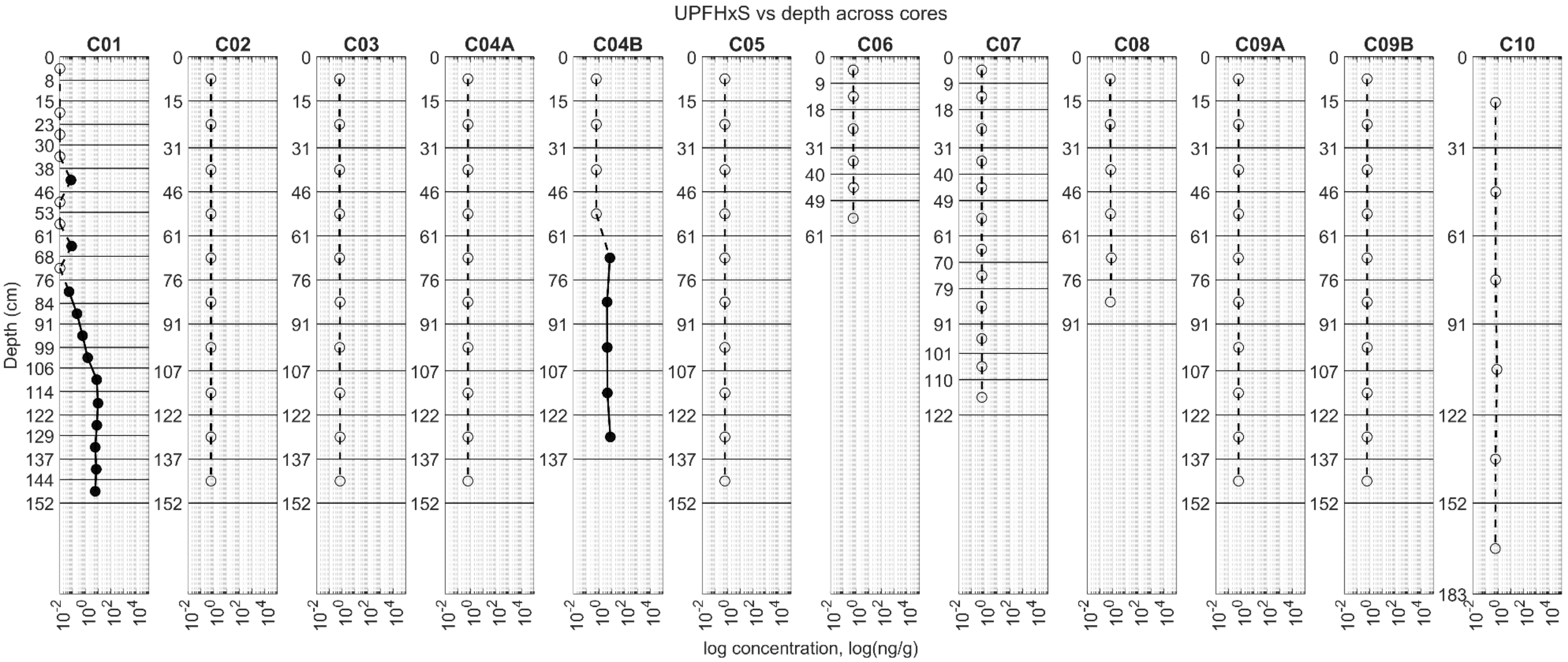

Figure S80. Vertical distribution profile of **UPFHpS** across the twelve studied cores

Note that the concentration is shown on a log-base-10 scale. For any given plot and compound, open markers with dashed-line connectors represent sampled depth intervals where the compounds' concentration was below the reporting limit – the location of the open marker along the x-axis is representative of those reporting limits.

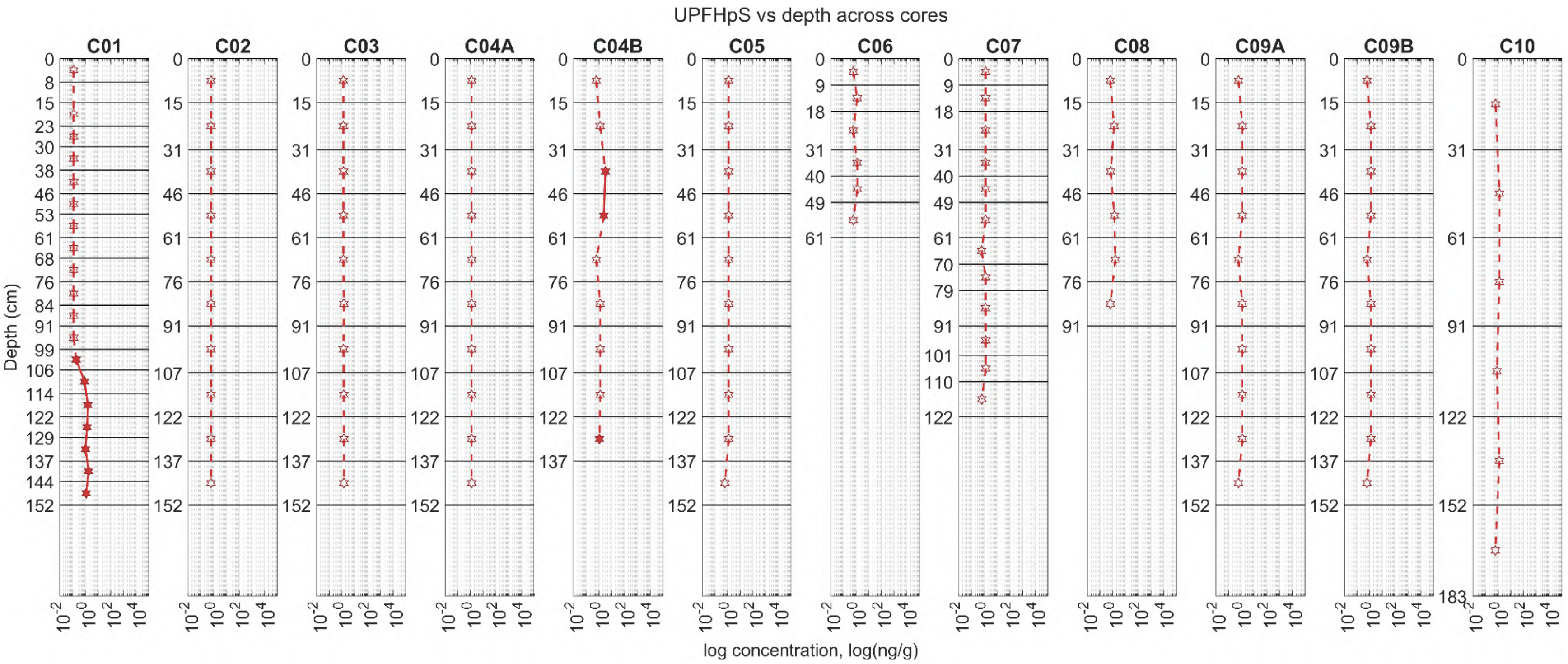

Figure S81. Vertical distribution profile of **UPFOS** across the twelve studied cores

Note that the concentration is shown on a log-base-10 scale. For any given plot and compound, open markers with dashed-line connectors represent sampled depth intervals where the compounds' concentration was below the reporting limit – the location of the open marker along the x-axis is representative of those reporting limits.

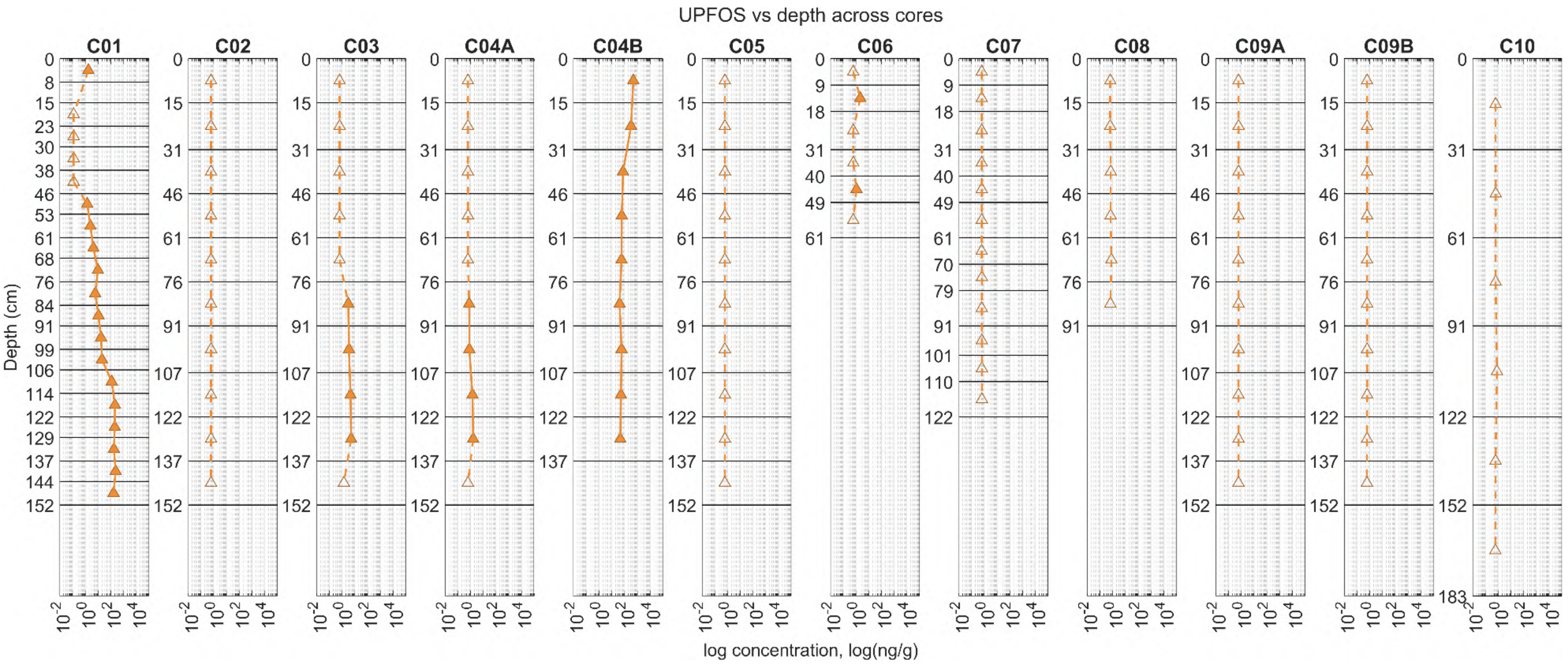

Figure S82. Vertical distribution profile of **UPFNS** across the twelve studied cores

Note that the concentration is shown on a log-base-10 scale. For any given plot and compound, open markers with dashed-line connectors represent sampled depth intervals where the compounds' concentration was below the reporting limit – the location of the open marker along the x-axis is representative of those reporting limits.

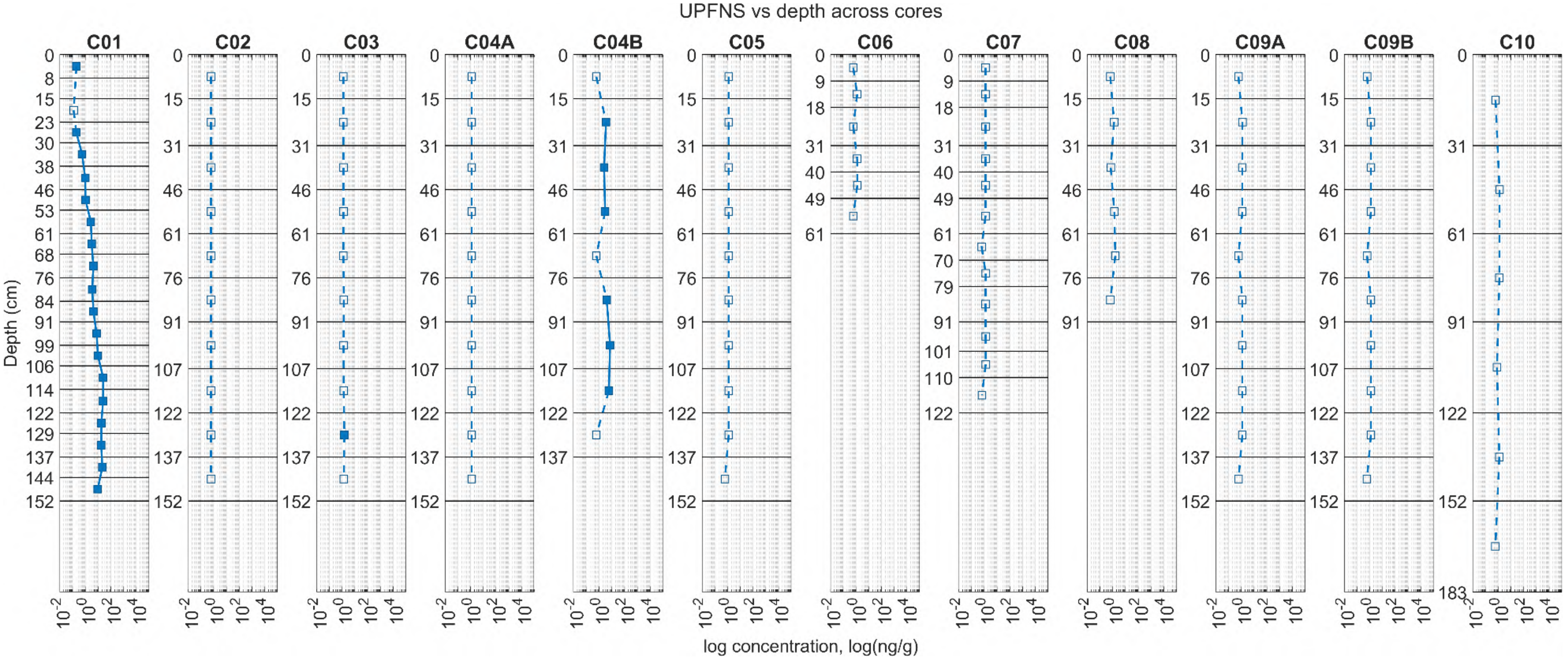

Figure S83. Vertical distribution profile of **UPFDS** across the twelve studied cores

Note that the concentration is shown on a log-base-10 scale. For any given plot and compound, open markers with dashed-line connectors represent sampled depth intervals where the compounds' concentration was below the reporting limit – the location of the open marker along the x-axis is representative of those reporting limits.

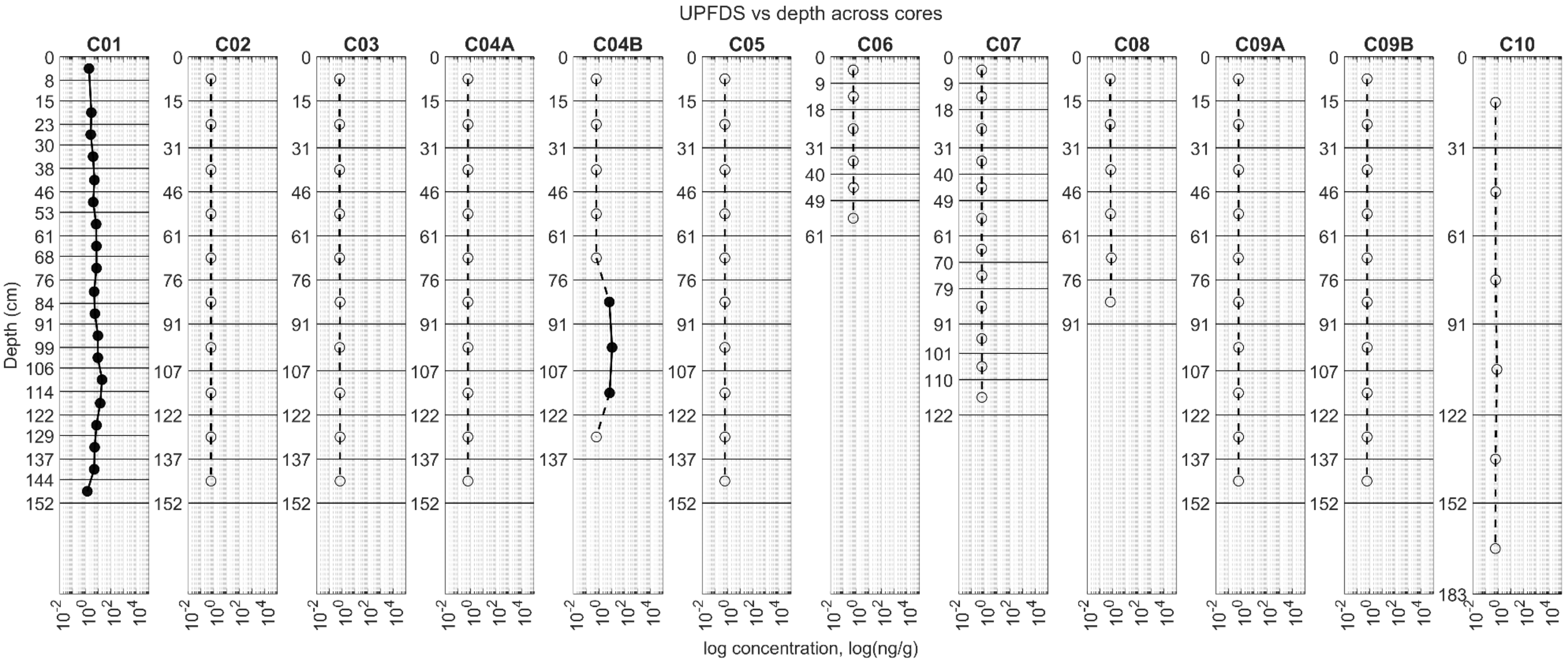

Figure S84. Vertical distribution profile of **PFPe-OS** across the twelve studied cores

Note that the concentration is shown on a log-base-10 scale. For any given plot and compound, open markers with dashed-line connectors represent sampled depth intervals where the compounds' concentration was below the reporting limit – the location of the open marker along the x-axis is representative of those reporting limits.

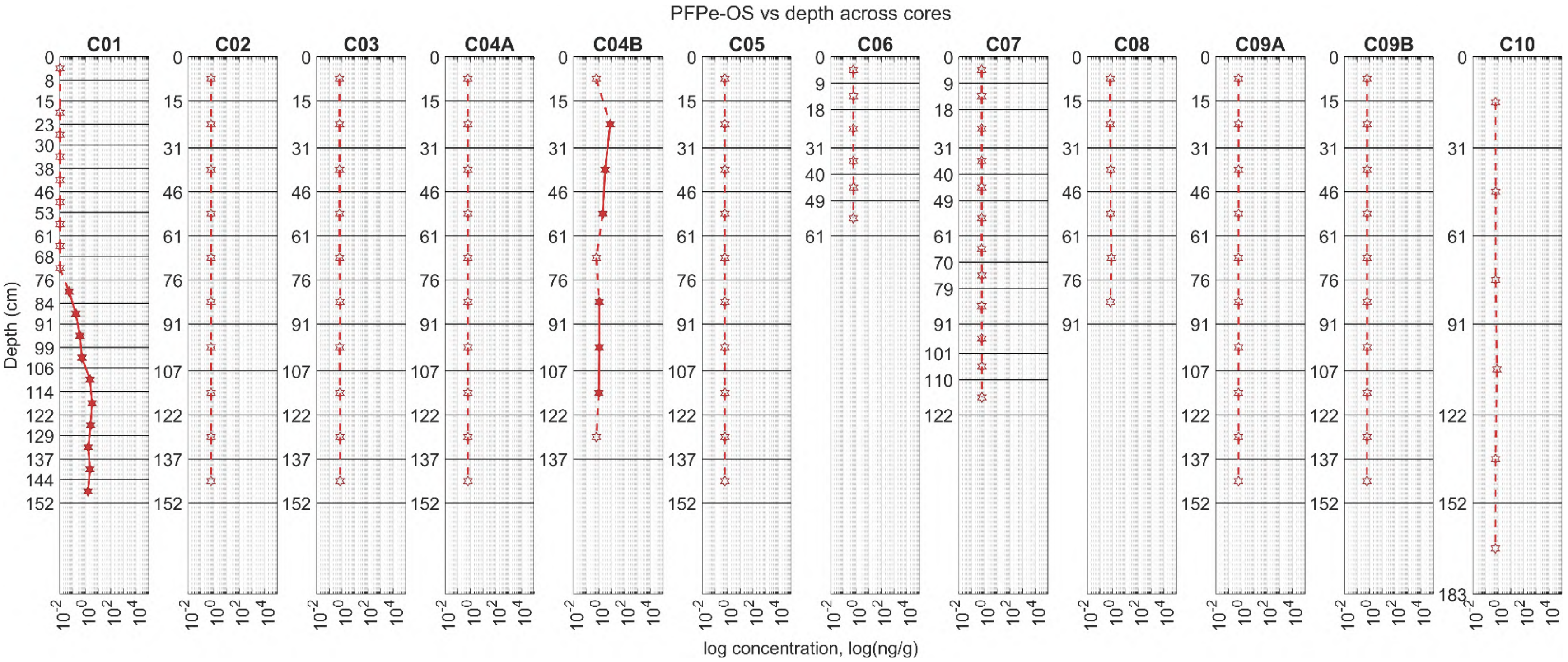

Figure S85. Vertical distribution profile of **PFHx-OS** across the twelve studied cores

Note that the concentration is shown on a log-base-10 scale. For any given plot and compound, open markers with dashed-line connectors represent sampled depth intervals where the compounds' concentration was below the reporting limit – the location of the open marker along the x-axis is representative of those reporting limits.

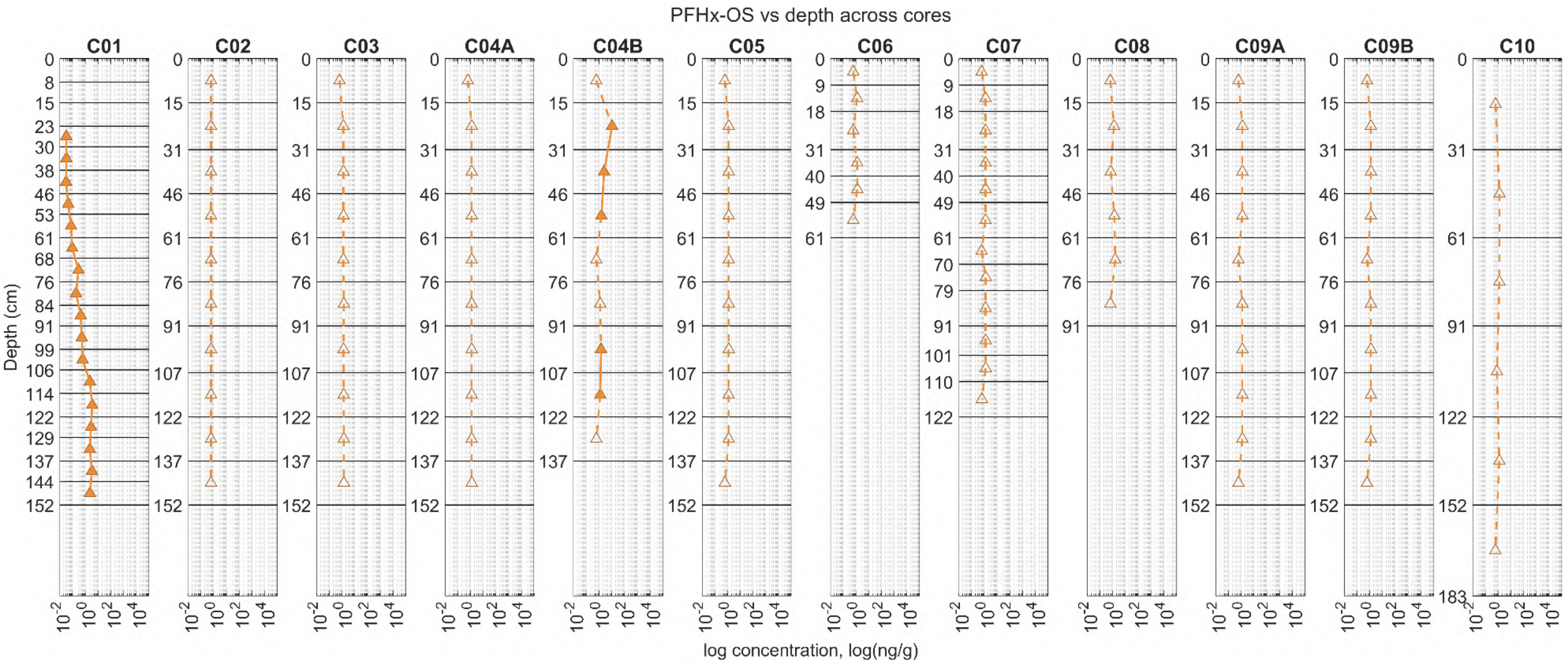

Figure S86. Vertical distribution profile of **PFHp-OS** across the twelve studied cores

Note that the concentration is shown on a log-base-10 scale. For any given plot and compound, open markers with dashed-line connectors represent sampled depth intervals where the compounds' concentration was below the reporting limit – the location of the open marker along the x-axis is representative of those reporting limits.

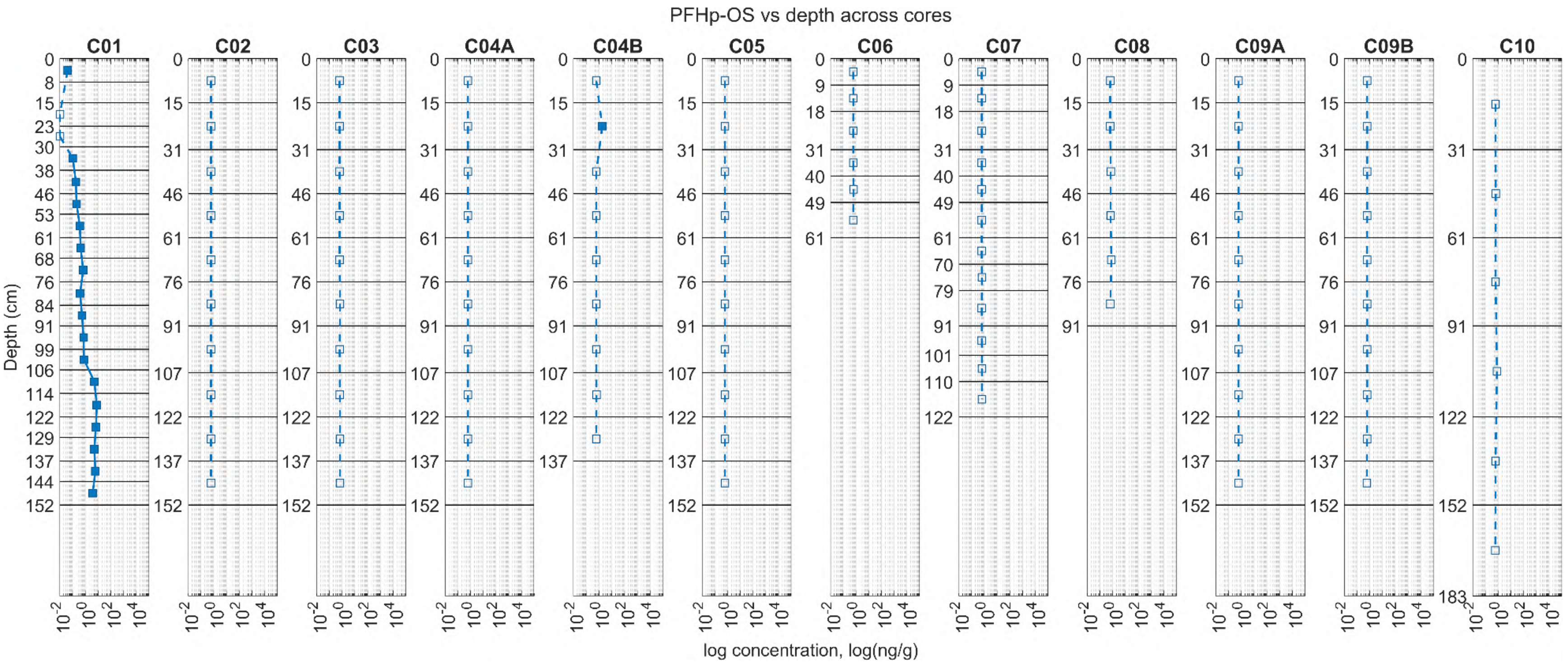

Figure S87. Vertical distribution profile of **PFO-OS** across the twelve studied cores

Note that the concentration is shown on a log-base-10 scale. For any given plot and compound, open markers with dashed-line connectors represent sampled depth intervals where the compounds' concentration was below the reporting limit – the location of the open marker along the x-axis is representative of those reporting limits.

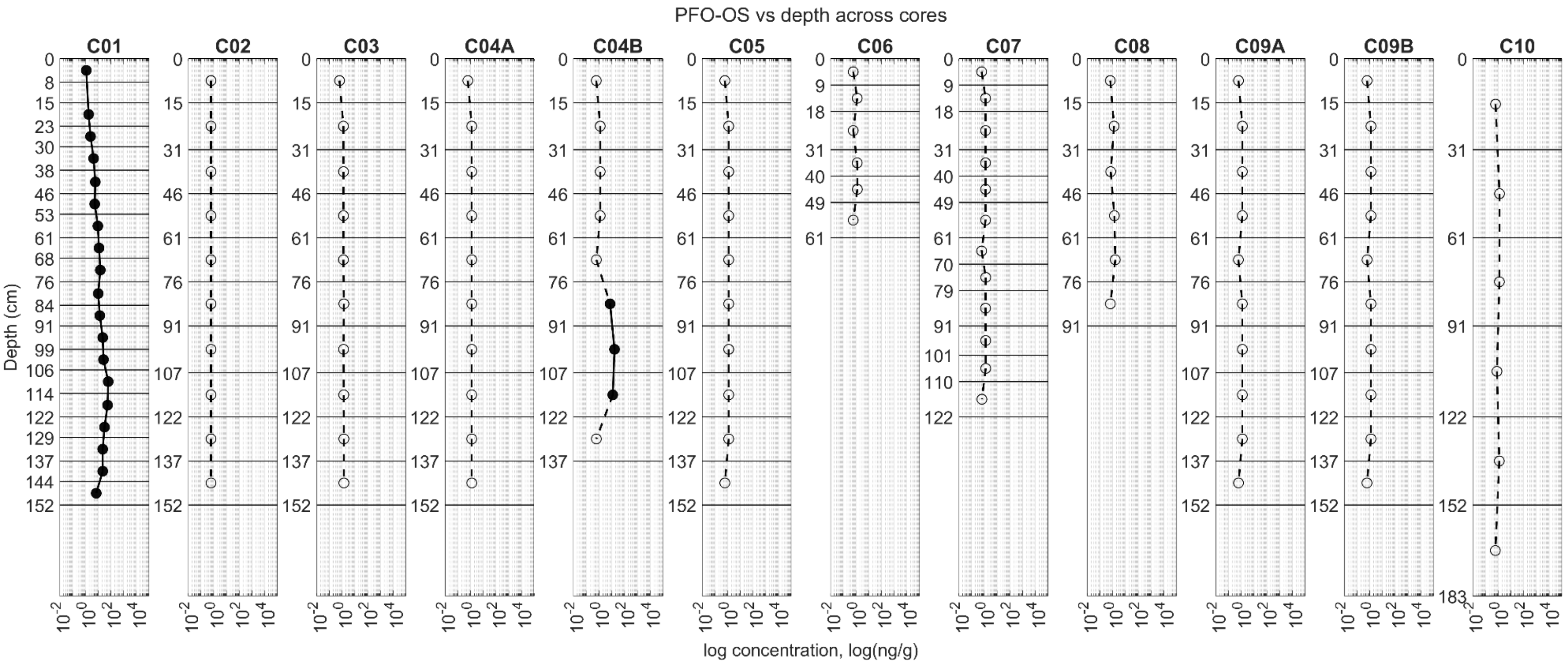

Figure S88. Vertical distribution profile of **PFN-OS** across the twelve studied cores

Note that the concentration is shown on a log-base-10 scale. For any given plot and compound, open markers with dashed-line connectors represent sampled depth intervals where the compounds' concentration was below the reporting limit – the location of the open marker along the x-axis is representative of those reporting limits.

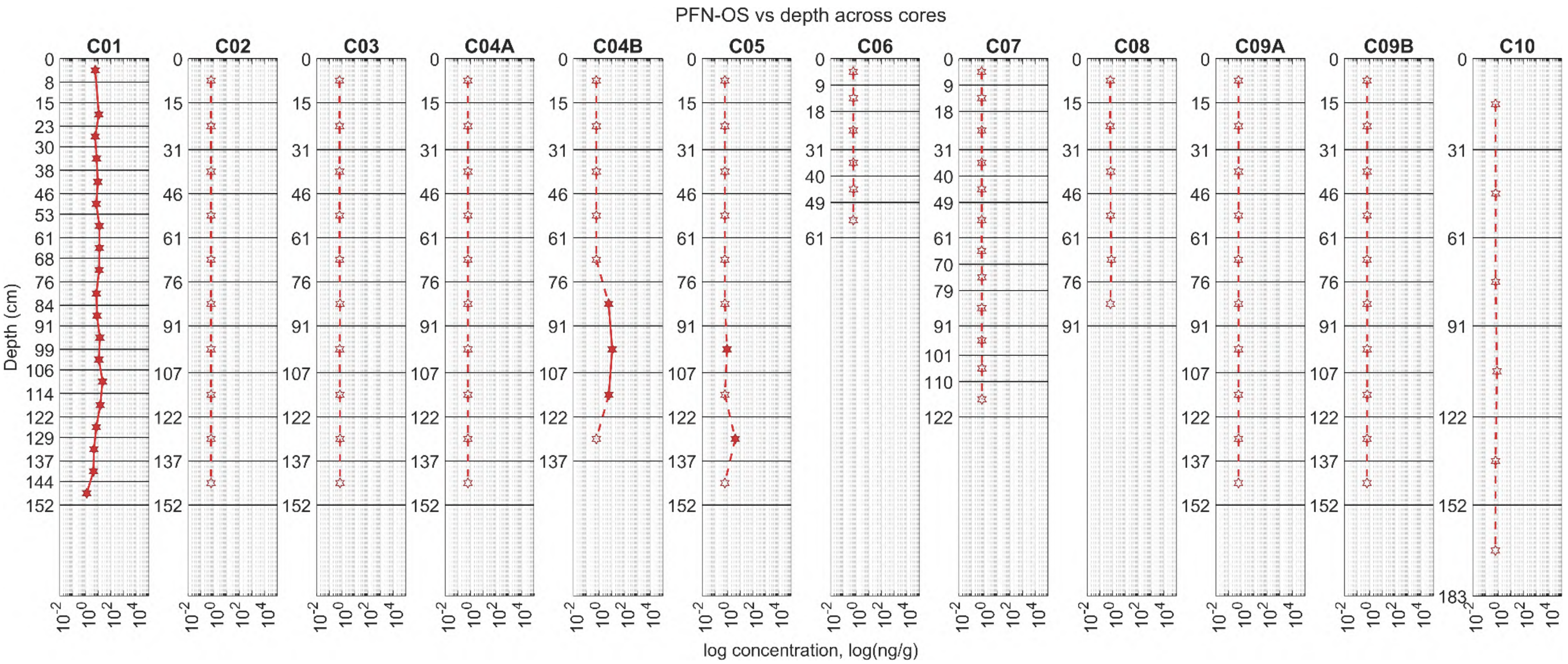

Figure S89. Vertical distribution profile of **1OH-4:2 FTS** across the twelve studied cores

Note that the concentration is shown on a log-base-10 scale. For any given plot and compound, open markers with dashed-line connectors represent sampled depth intervals where the compounds' concentration was below the reporting limit – the location of the open marker along the x-axis is representative of those reporting limits.

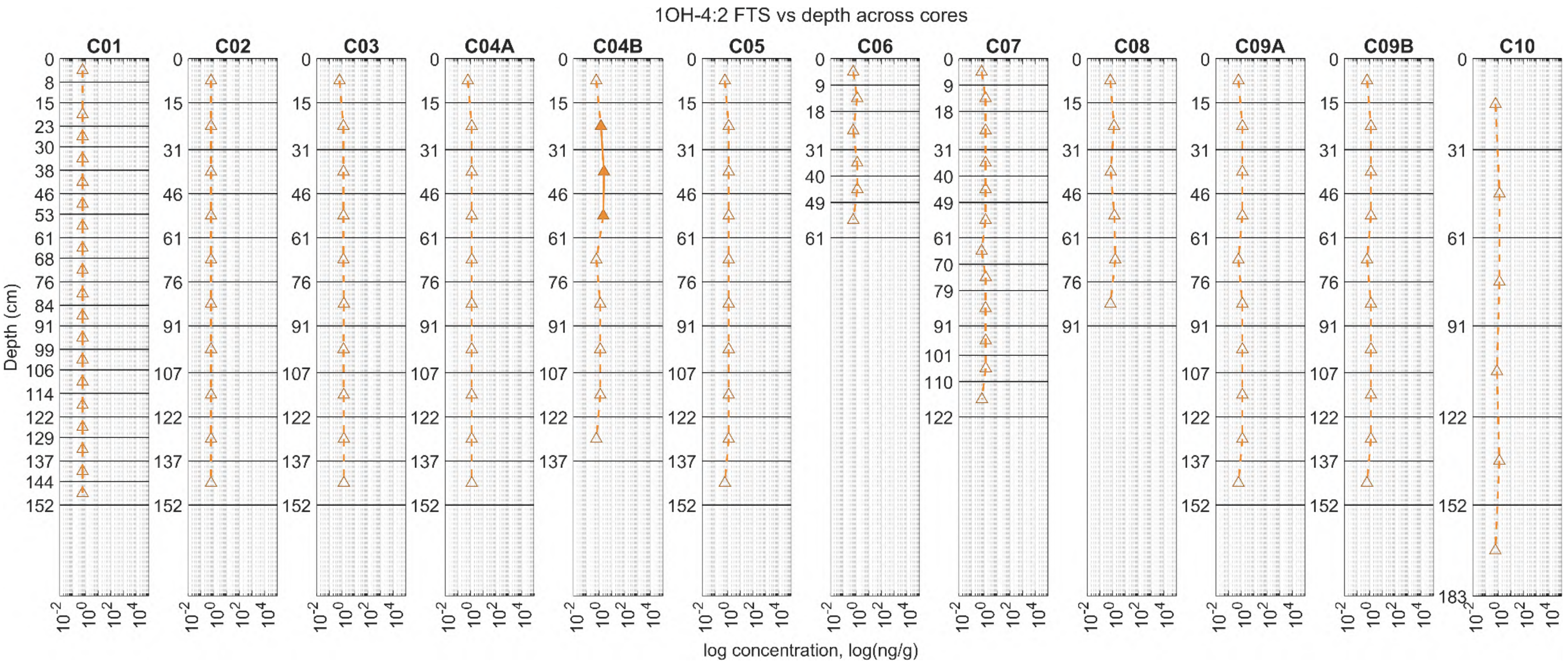

Figure S90. Vertical distribution profile of **1OH-6:2 FTS** across the twelve studied cores

Note that the concentration is shown on a log-base-10 scale. For any given plot and compound, open markers with dashed-line connectors represent sampled depth intervals where the compounds' concentration was below the reporting limit – the location of the open marker along the x-axis is representative of those reporting limits.

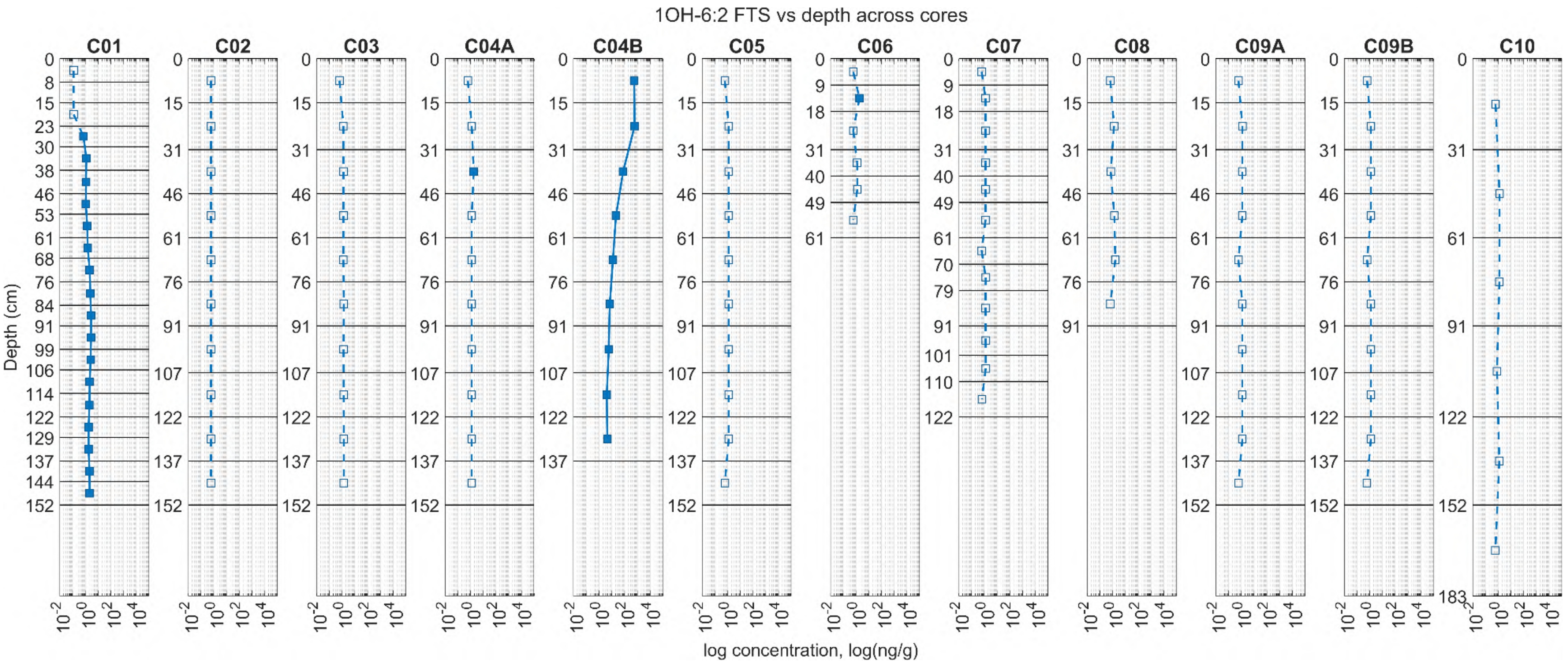

Figure S91. Vertical distribution profile of **6:2 FTSi** across the twelve studied cores

Note that the concentration is shown on a log-base-10 scale. For any given plot and compound, open markers with dashed-line connectors represent sampled depth intervals where the compounds' concentration was below the reporting limit – the location of the open marker along the x-axis is representative of those reporting limits.

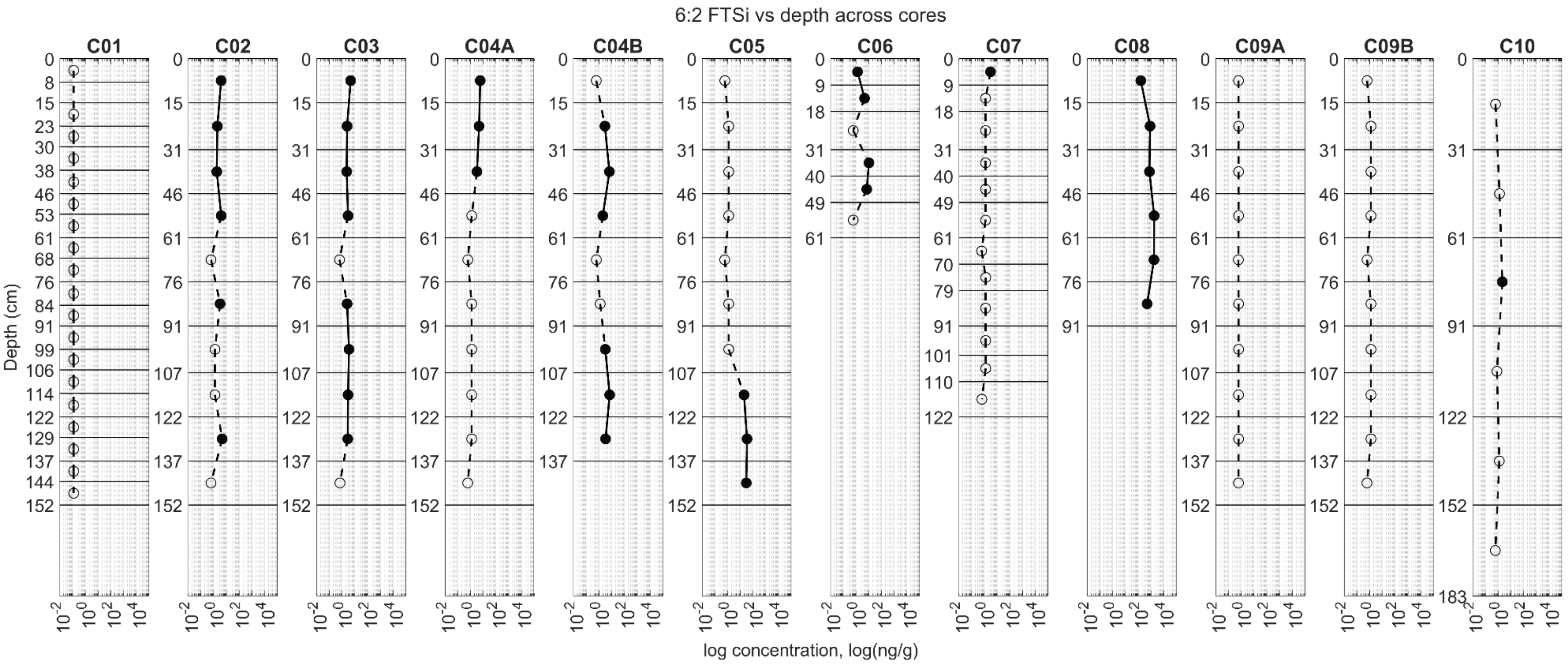

Figure S92. Vertical distribution profile of **8:2 FTSi** across the twelve studied cores

Note that the concentration is shown on a log-base-10 scale. For any given plot and compound, open markers with dashed-line connectors represent sampled depth intervals where the compounds' concentration was below the reporting limit – the location of the open marker along the x-axis is representative of those reporting limits.

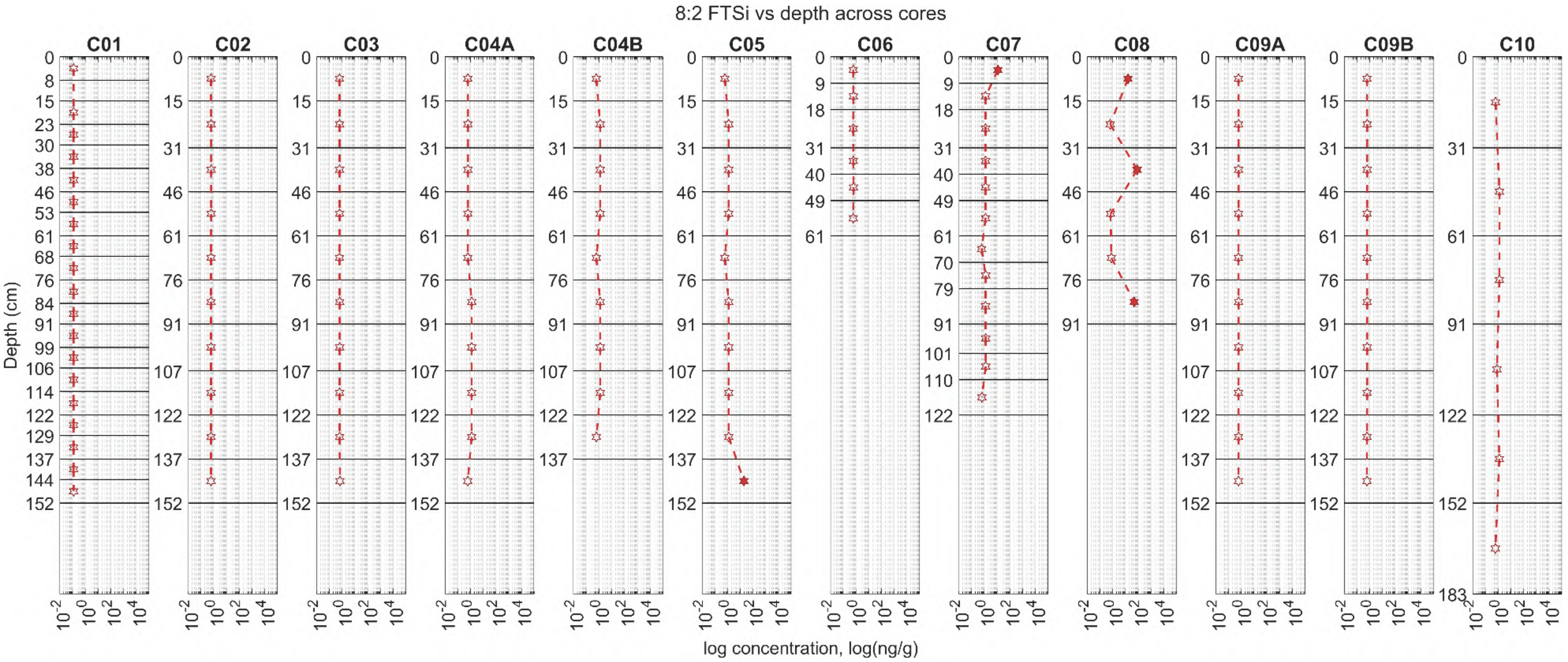

Figure S93. Vertical distribution profile of **EtFHxSA** across the twelve studied cores

Note that the concentration is shown on a log-base-10 scale. For any given plot and compound, open markers with dashed-line connectors represent sampled depth intervals where the compounds' concentration was below the reporting limit – the location of the open marker along the x-axis is representative of those reporting limits.

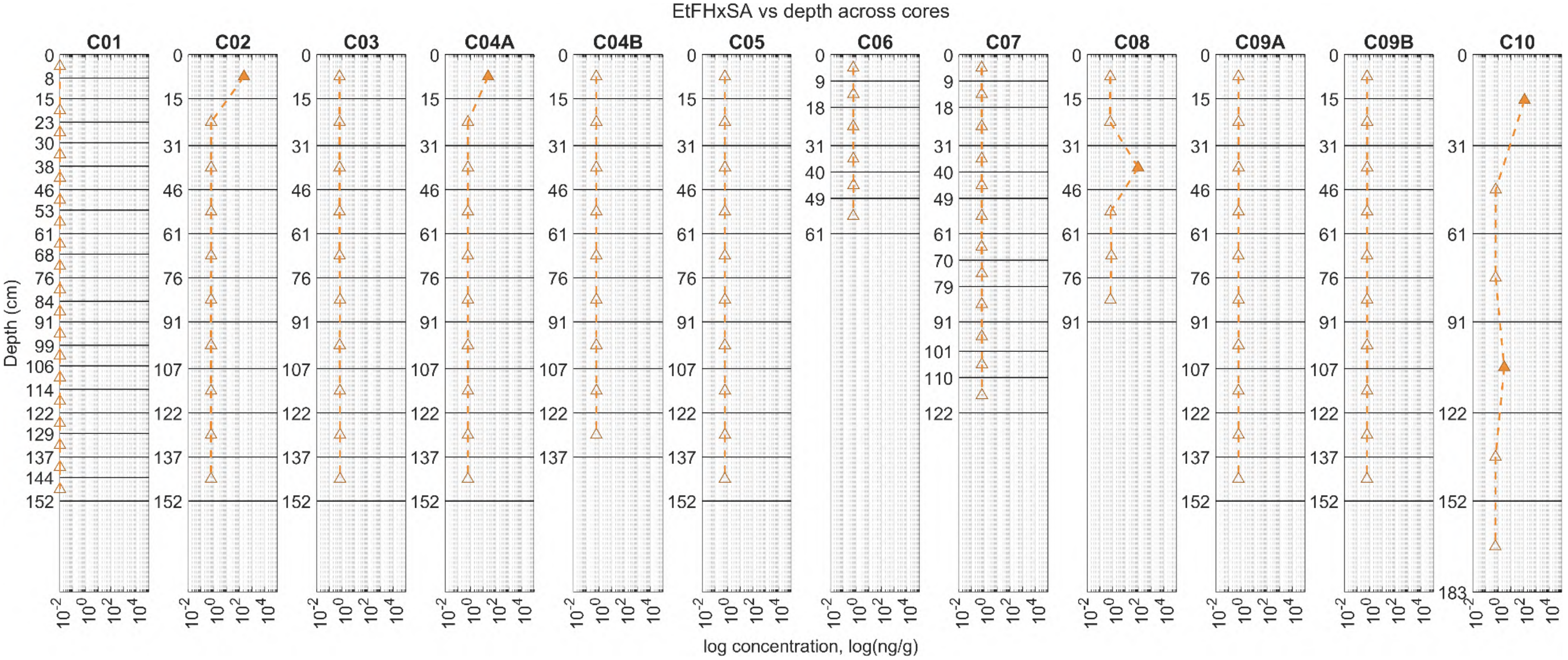

Figure S94. Vertical distribution profile of **EtFDSA** across the twelve studied cores

Note that the concentration is shown on a log-base-10 scale. For any given plot and compound, open markers with dashed-line connectors represent sampled depth intervals where the compounds' concentration was below the reporting limit – the location of the open marker along the x-axis is representative of those reporting limits.

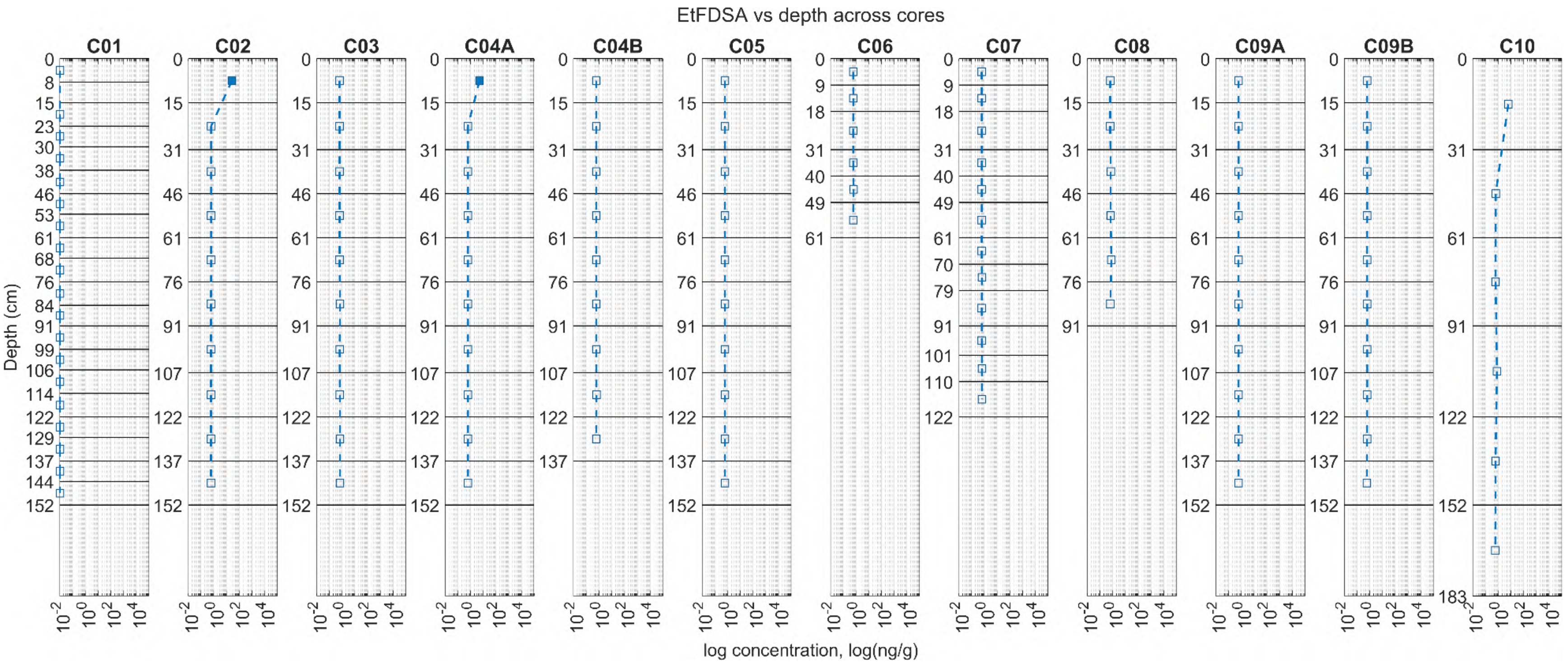

Figure S95. Vertical distribution profile of **EtFDoSA** across the twelve studied cores

Note that the concentration is shown on a log-base-10 scale. For any given plot and compound, open markers with dashed-line connectors represent sampled depth intervals where the compounds' concentration was below the reporting limit – the location of the open marker along the x-axis is representative of those reporting limits.

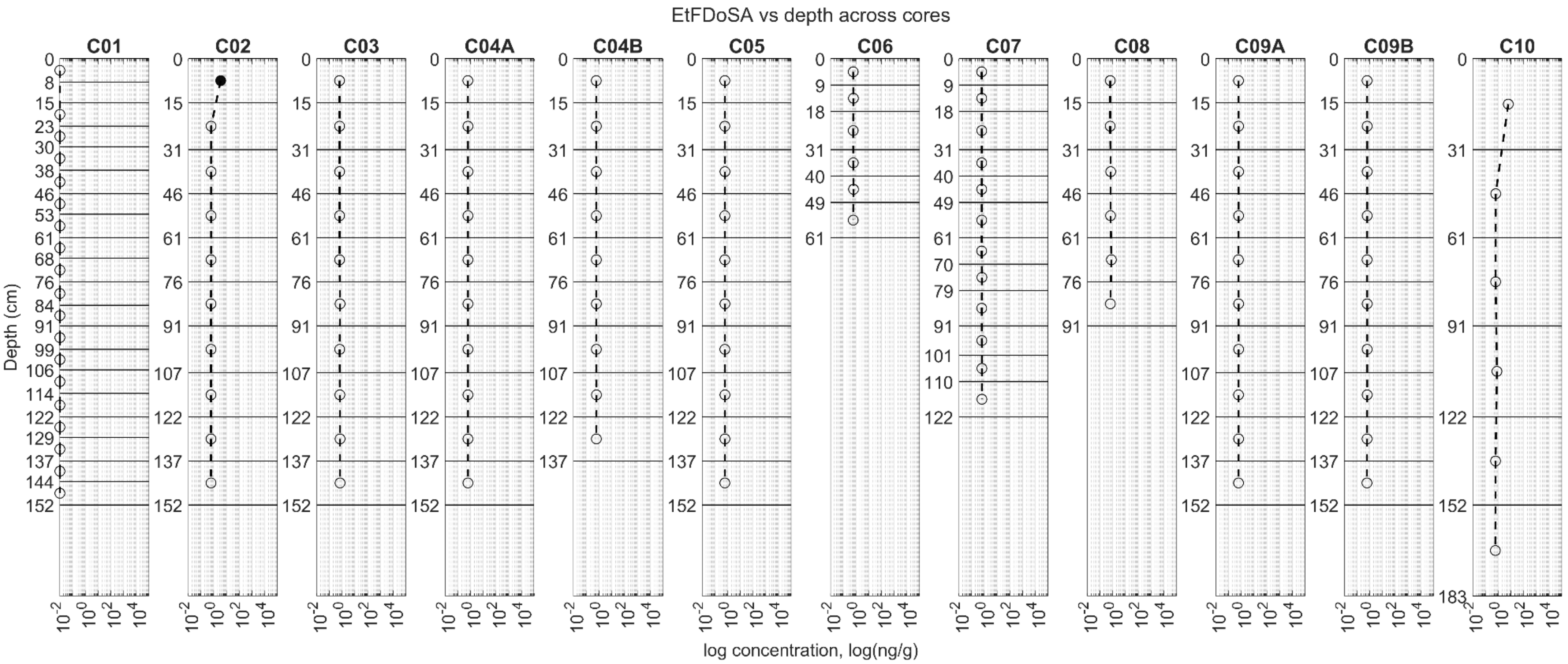

Figure S96. Vertical distribution profile of **MeFBSA** across the twelve studied cores

Note that the concentration is shown on a log-base-10 scale. For any given plot and compound, open markers with dashed-line connectors represent sampled depth intervals where the compounds' concentration was below the reporting limit – the location of the open marker along the x-axis is representative of those reporting limits.

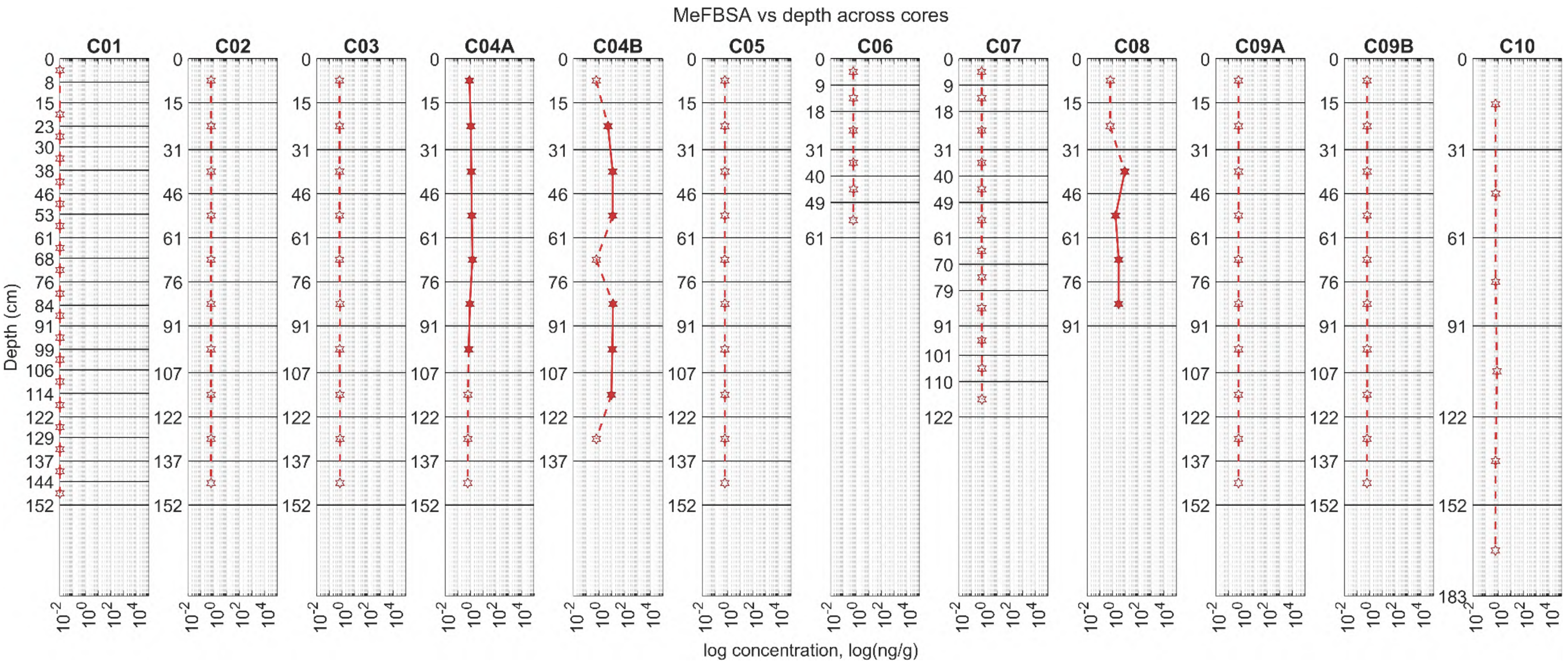

Figure S97. Vertical distribution profile of **MeFPeSA** across the twelve studied cores

Note that the concentration is shown on a log-base-10 scale. For any given plot and compound, open markers with dashed-line connectors represent sampled depth intervals where the compounds' concentration was below the reporting limit – the location of the open marker along the x-axis is representative of those reporting limits.

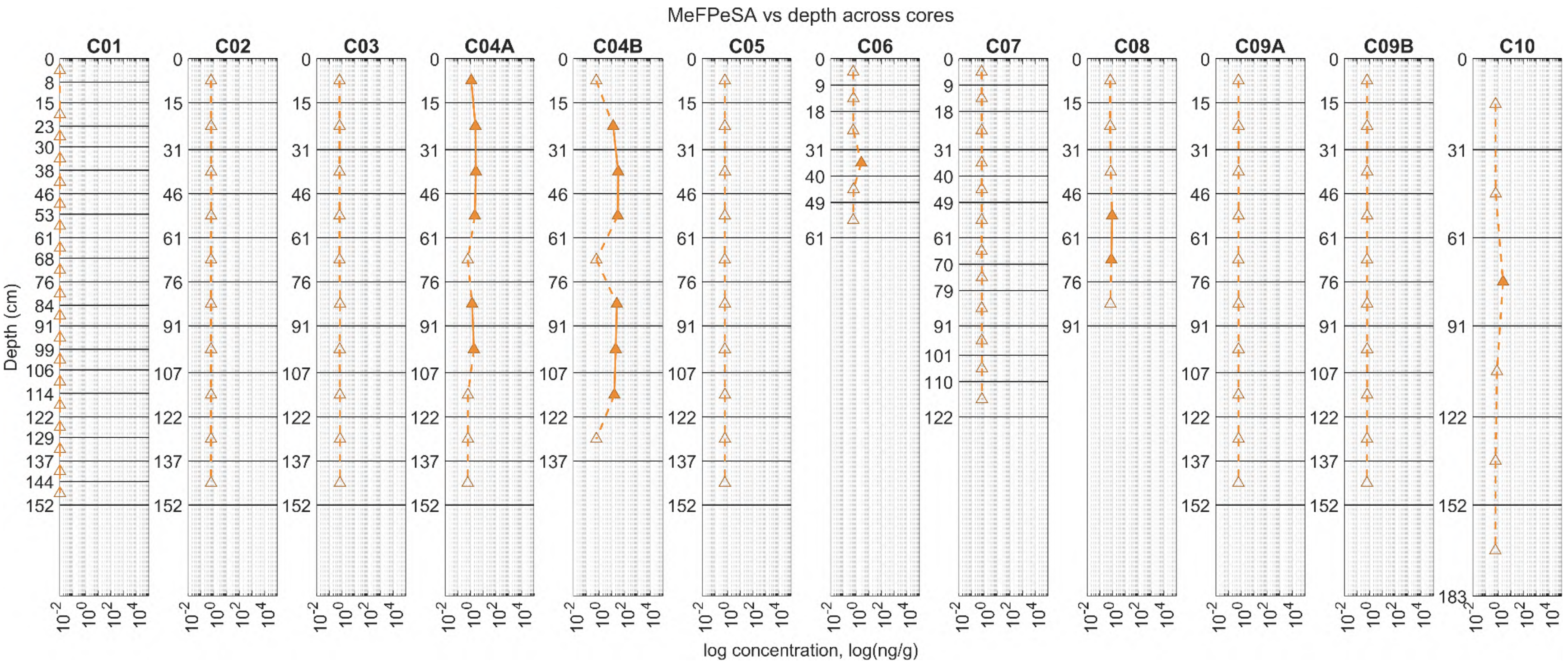

Figure S98. Vertical distribution profile of **MeFHxSA** across the twelve studied cores

Note that the concentration is shown on a log-base-10 scale. For any given plot and compound, open markers with dashed-line connectors represent sampled depth intervals where the compounds' concentration was below the reporting limit – the location of the open marker along

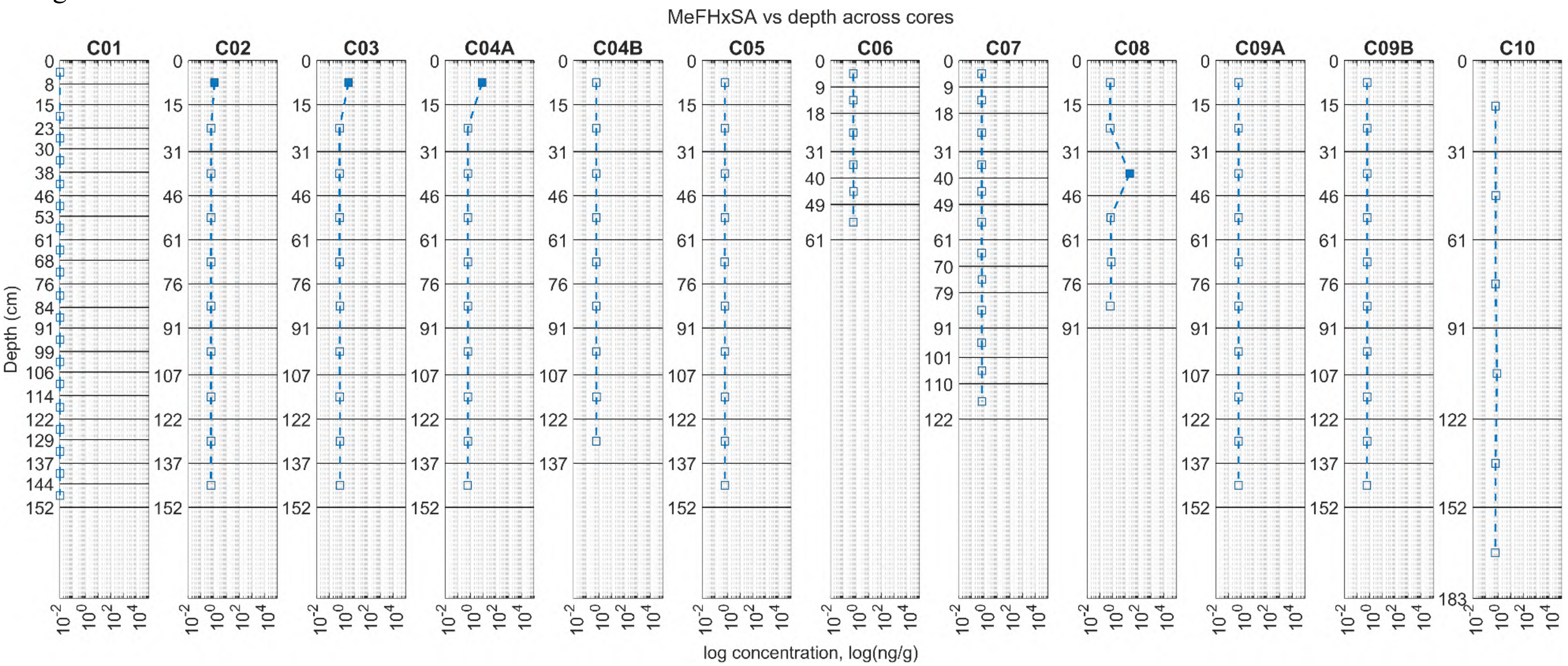

the x-axis is representative of those reporting limits.

Figure S99. Vertical distribution profile of **MeFOSA** across the twelve studied cores

Note that the concentration is shown on a log-base-10 scale. For any given plot and compound, open markers with dashed-line connectors represent sampled depth intervals where the compounds' concentration was below the reporting limit – the location of the open marker along the x-axis is representative of those reporting limits.

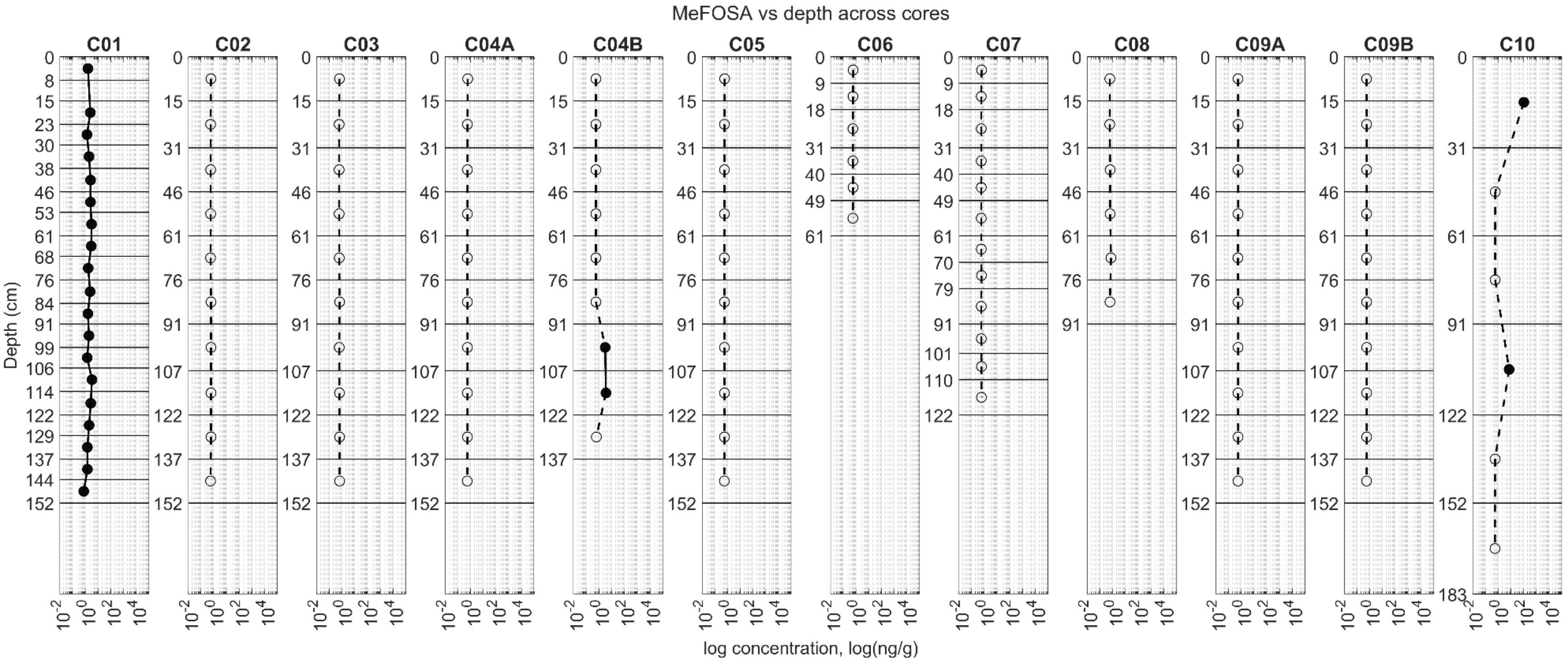

Figure S100. Vertical distribution profile of **CMeAmPr-FPrSAPrA** across the twelve studied cores

Note that the concentration is shown on a log-base-10 scale. For any given plot and compound, open markers with dashed-line connectors represent sampled depth intervals where the compounds' concentration was below the reporting limit – the location of the open marker along the x-axis is representative of those reporting limits.

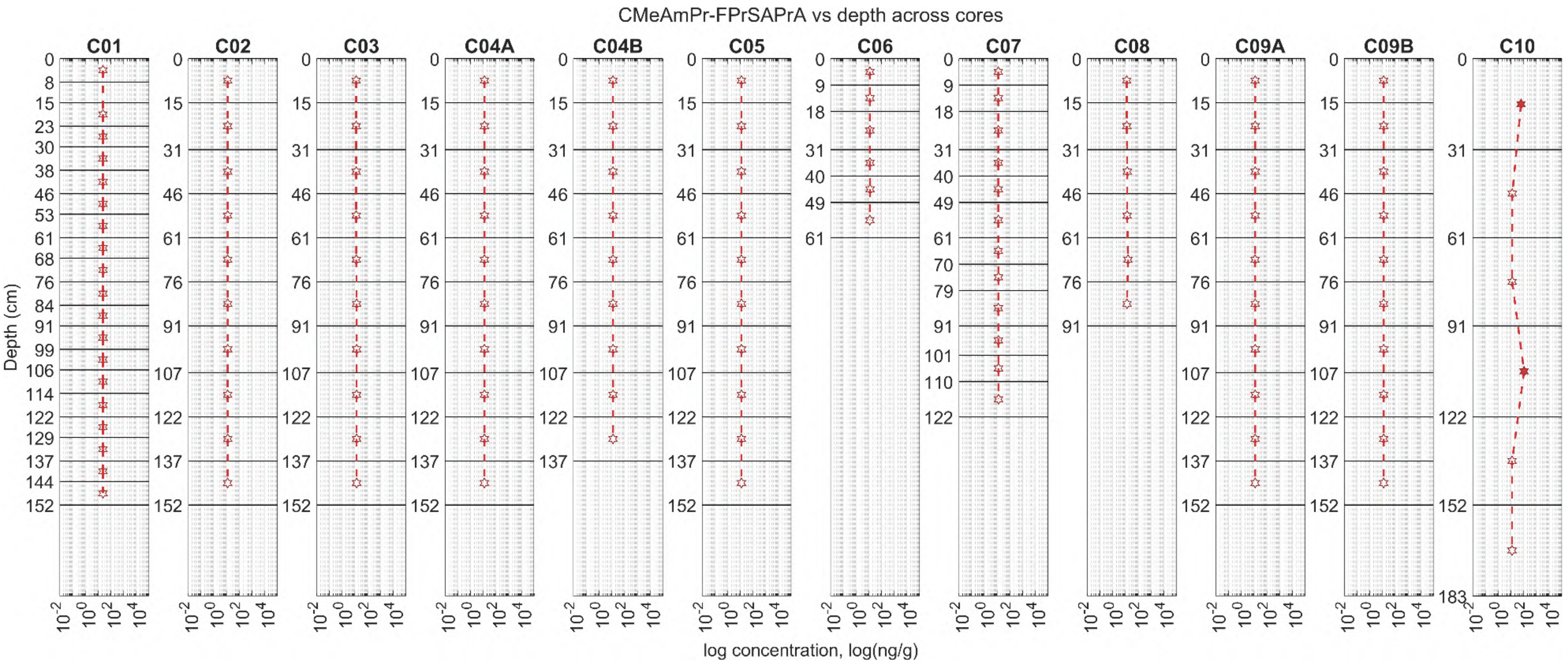

Figure S101. Vertical distribution profile of **CMeAmPr-FBSAPrA** across the twelve studied cores

Note that the concentration is shown on a log-base-10 scale. For any given plot and compound, open markers with dashed-line connectors represent sampled depth intervals where the compounds' concentration was below the reporting limit – the location of the open marker along the x-axis is representative of those reporting limits.

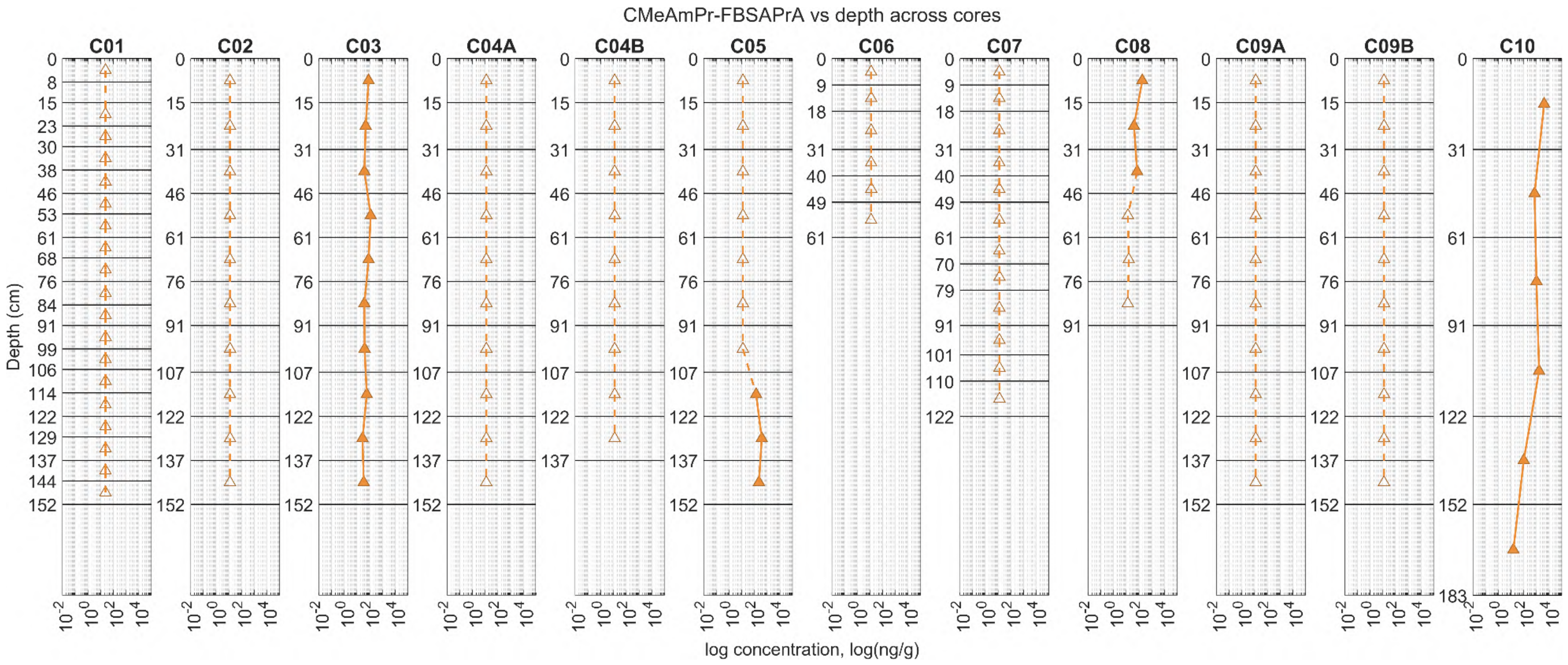

Figure S102. Vertical distribution profile of **CMeAmPr-FHxSAPrA** across the twelve studied cores

Note that the concentration is shown on a log-base-10 scale. For any given plot and compound, open markers with dashed-line connectors represent sampled depth intervals where the compounds' concentration was below the reporting limit – the location of the open marker along the x-axis is representative of those reporting limits.

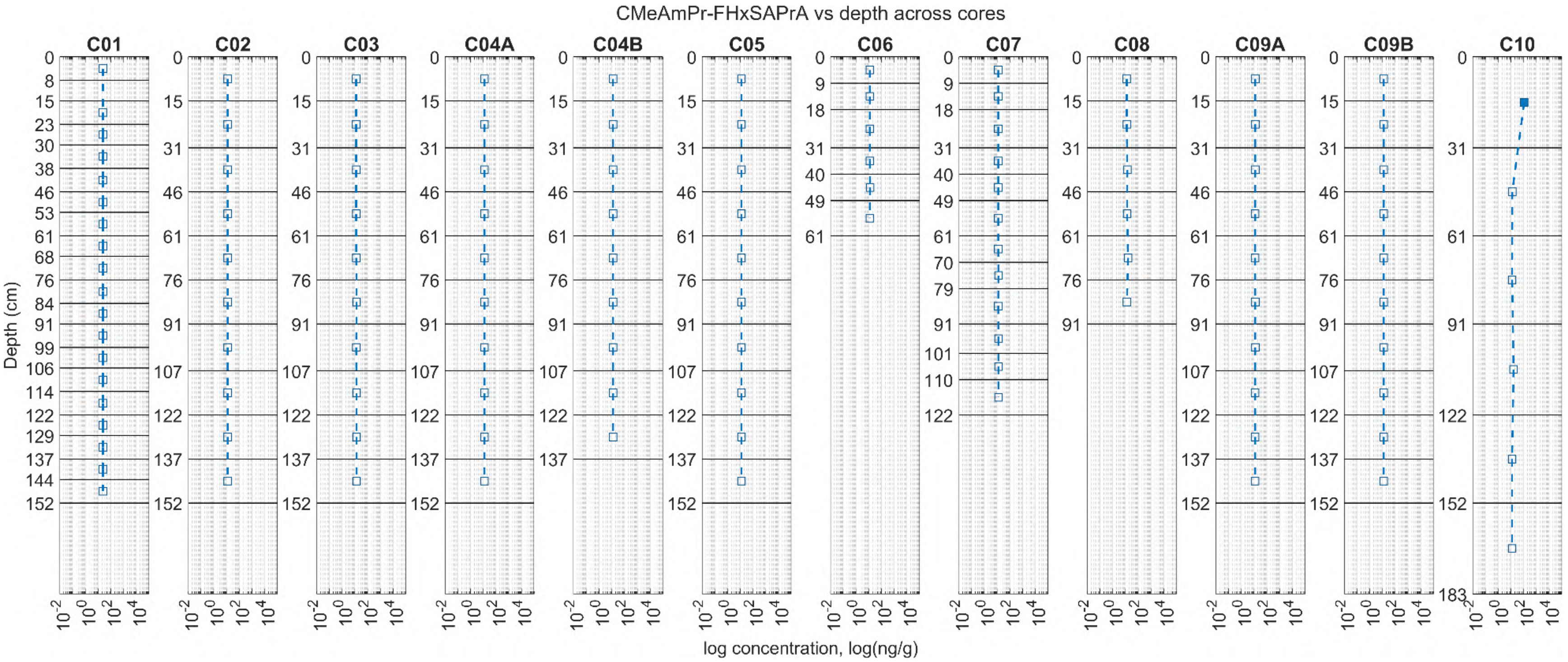

Figure S103. Vertical distribution profile of **CMeAmPr-FBSAA** across the twelve studied cores

Note that the concentration is shown on a log-base-10 scale. For any given plot and compound, open markers with dashed-line connectors represent sampled depth intervals where the compounds' concentration was below the reporting limit – the location of the open marker along the x-axis is representative of those reporting limits.

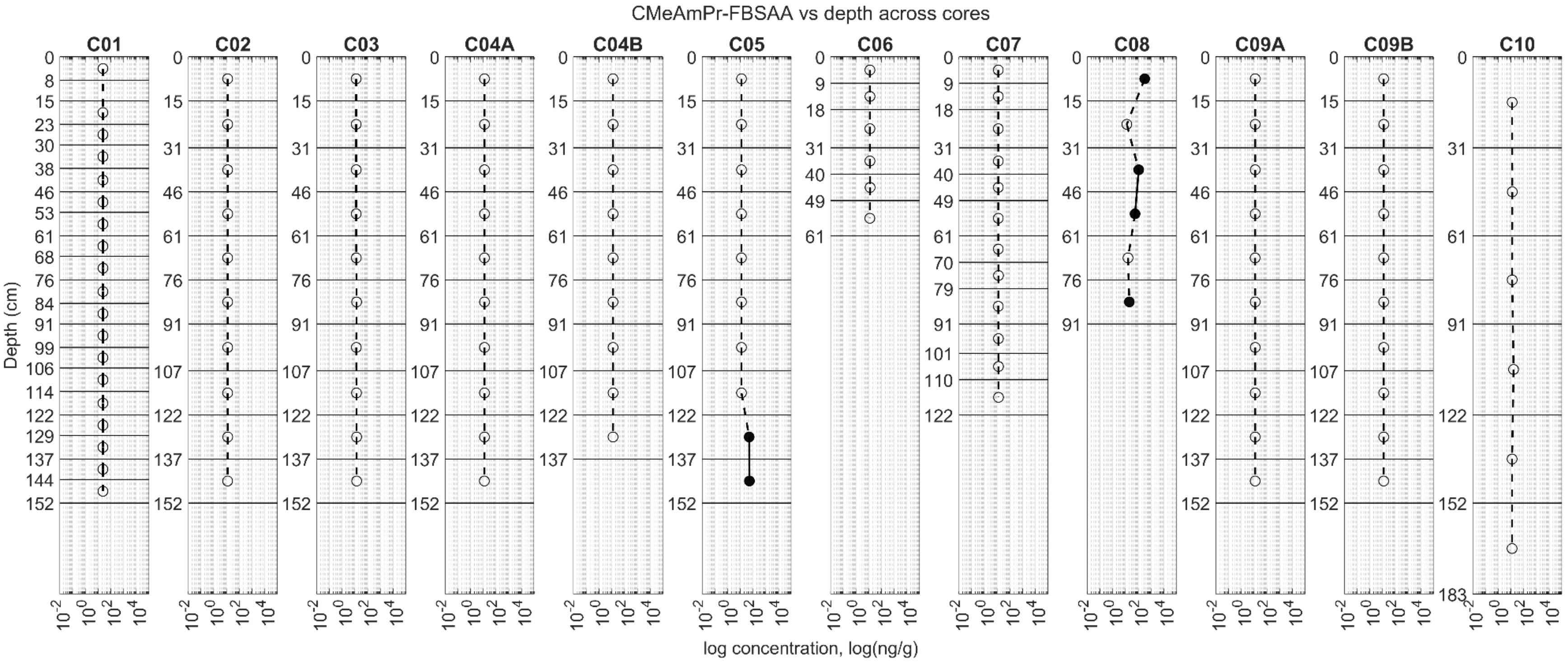

Figure S104. Vertical distribution profile of **CMeAmPr-FHxSAA** across the twelve studied cores

Note that the concentration is shown on a log-base-10 scale. For any given plot and compound, open markers with dashed-line connectors represent sampled depth intervals where the compounds' concentration was below the reporting limit – the location of the open marker along the x-axis is representative of those reporting limits.

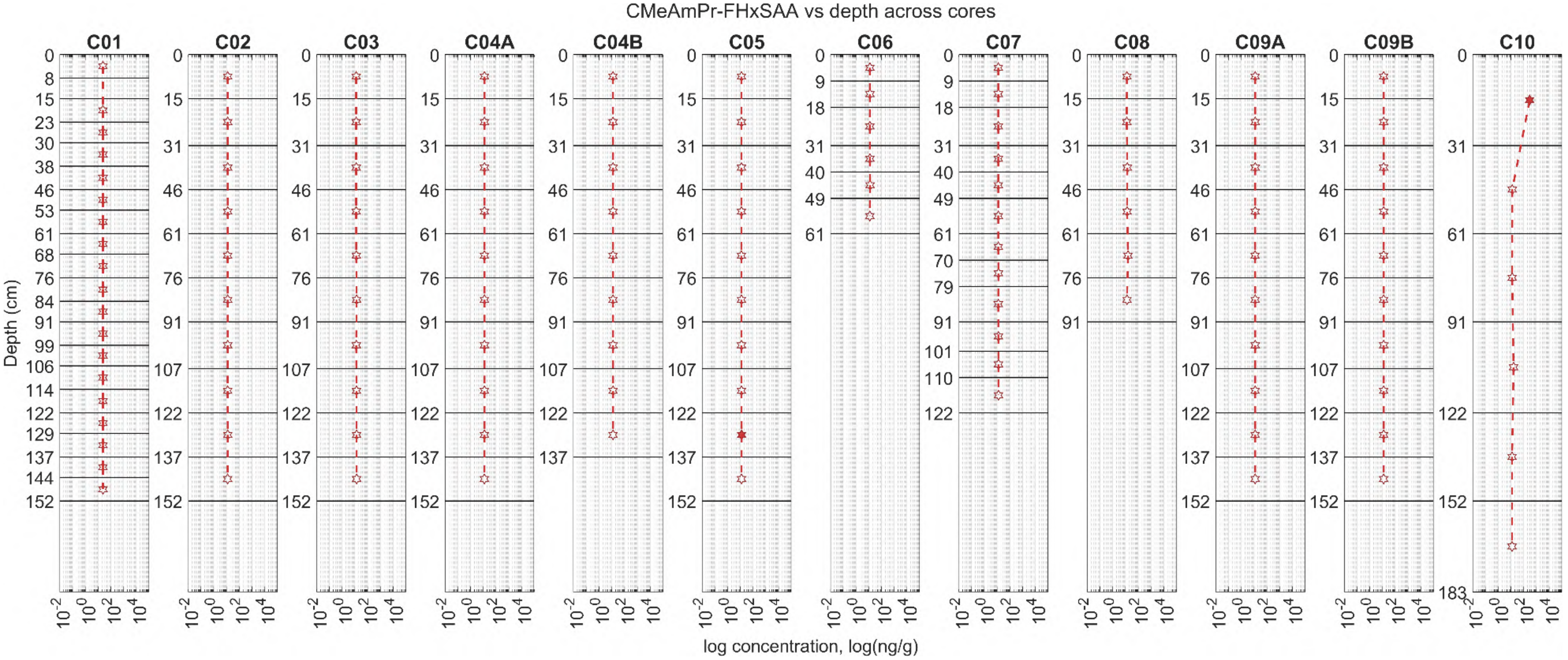

Figure S105. Vertical distribution profile of **CEtAmPr-N-EtFHxSA** across the twelve studied cores

Note that the concentration is shown on a log-base-10 scale. For any given plot and compound, open markers with dashed-line connectors represent sampled depth intervals where the compounds' concentration was below the reporting limit – the location of the open marker along the x-axis is representative of those reporting limits.

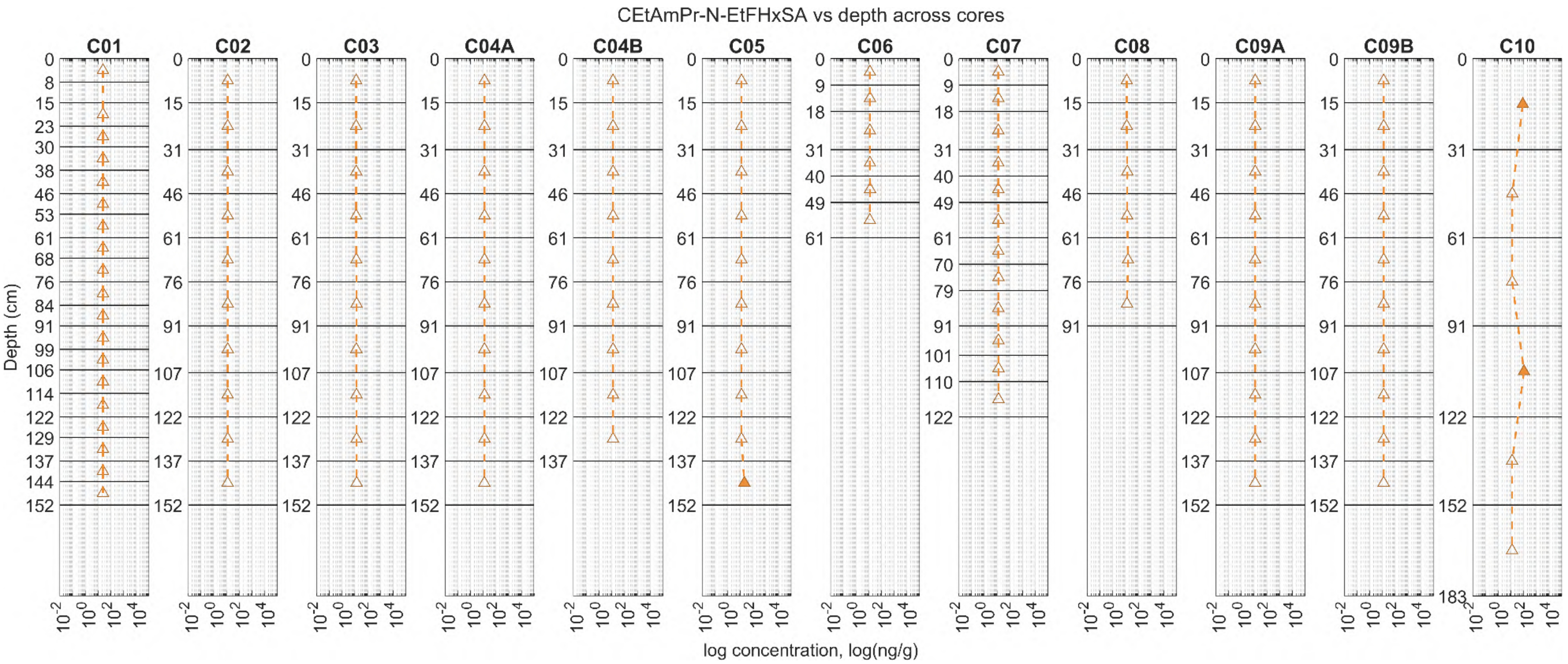

Figure S106. Vertical distribution profile of **diOHBAmPr-FBSA** across the twelve studied cores

Note that the concentration is shown on a log-base-10 scale. For any given plot and compound, open markers with dashed-line connectors represent sampled depth intervals where the compounds' concentration was below the reporting limit – the location of the open marker along the x-axis is representative of those reporting limits.

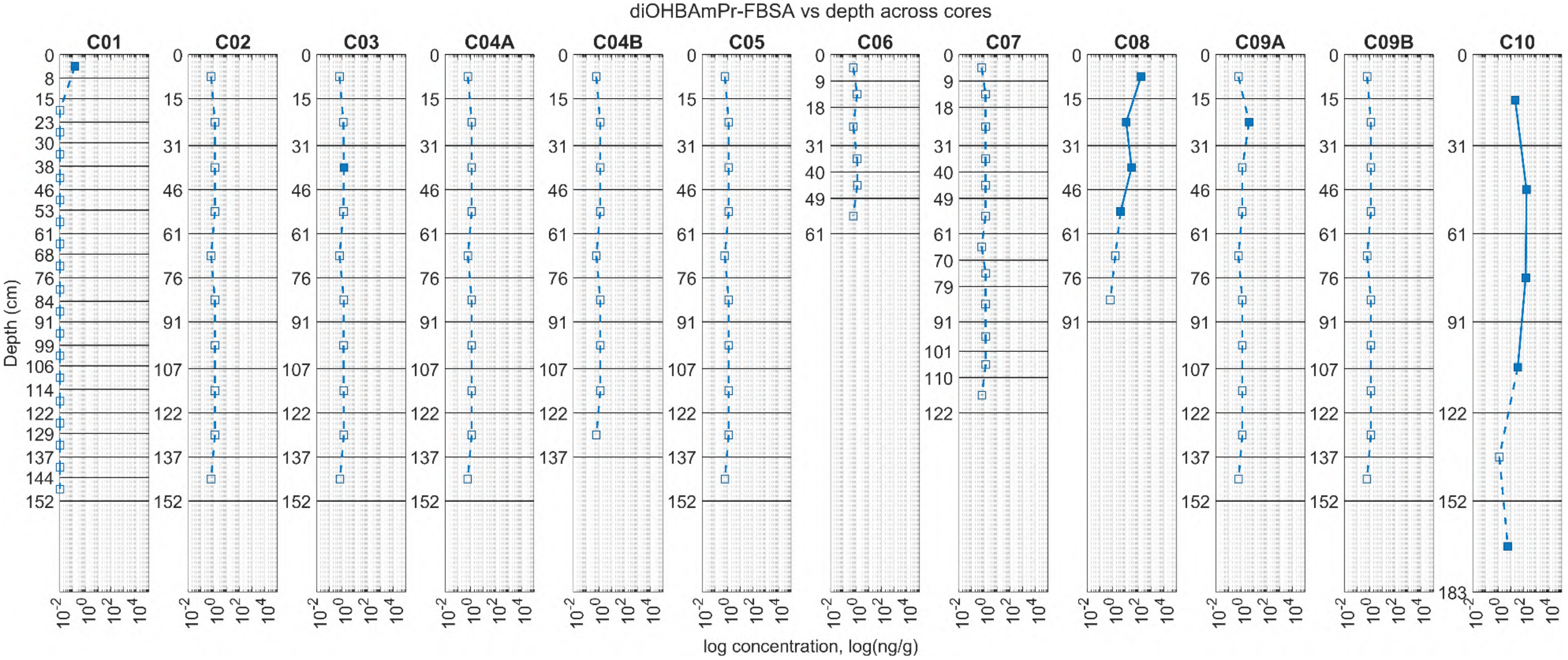

Figure S107. Vertical distribution profile of **diOHBAmPr-FPeSA** across the twelve studied cores

Note that the concentration is shown on a log-base-10 scale. For any given plot and compound, open markers with dashed-line connectors represent sampled depth intervals where the compounds' concentration was below the reporting limit – the location of the open marker along the x-axis is representative of those reporting limits.

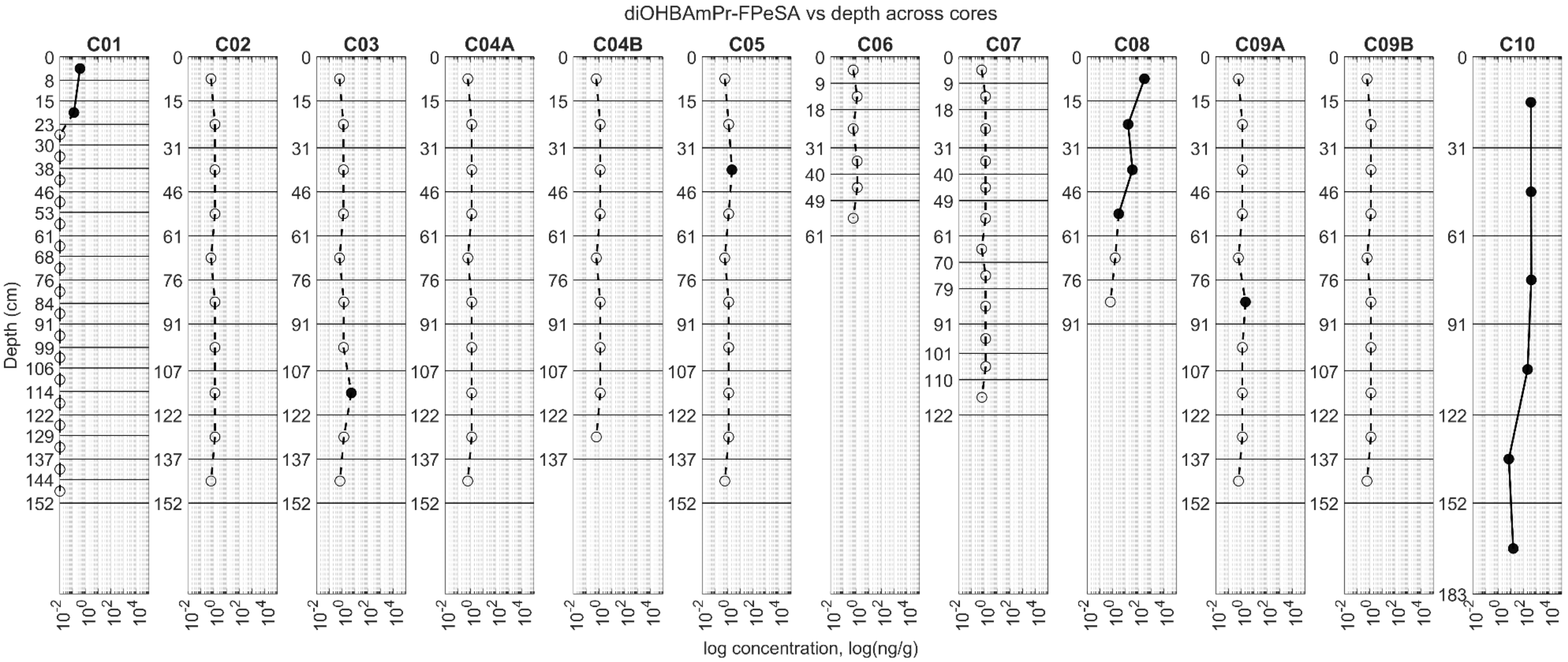

Figure S108. Vertical distribution profile of **diOHBAmPr-FHxSA** across the twelve studied cores

Note that the concentration is shown on a log-base-10 scale. For any given plot and compound, open markers with dashed-line connectors represent sampled depth intervals where the compounds' concentration was below the reporting limit – the location of the open marker along the x-axis is representative of those reporting limits.

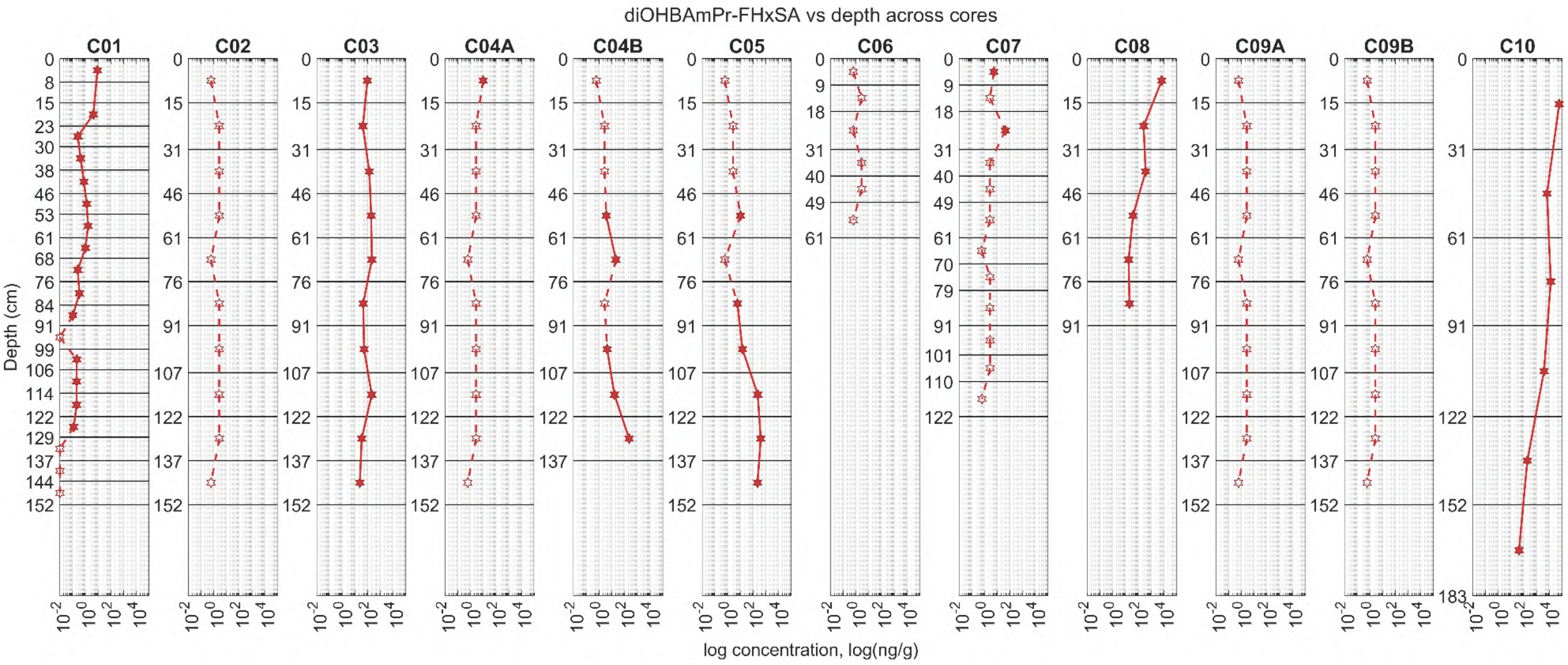

Figure S109. Vertical distribution profile of **diOHBAmPr-FHpSA** across the twelve studied cores

Note that the concentration is shown on a log-base-10 scale. For any given plot and compound, open markers with dashed-line connectors represent sampled depth intervals where the compounds' concentration was below the reporting limit – the location of the open marker along the x-axis is representative of those reporting limits.

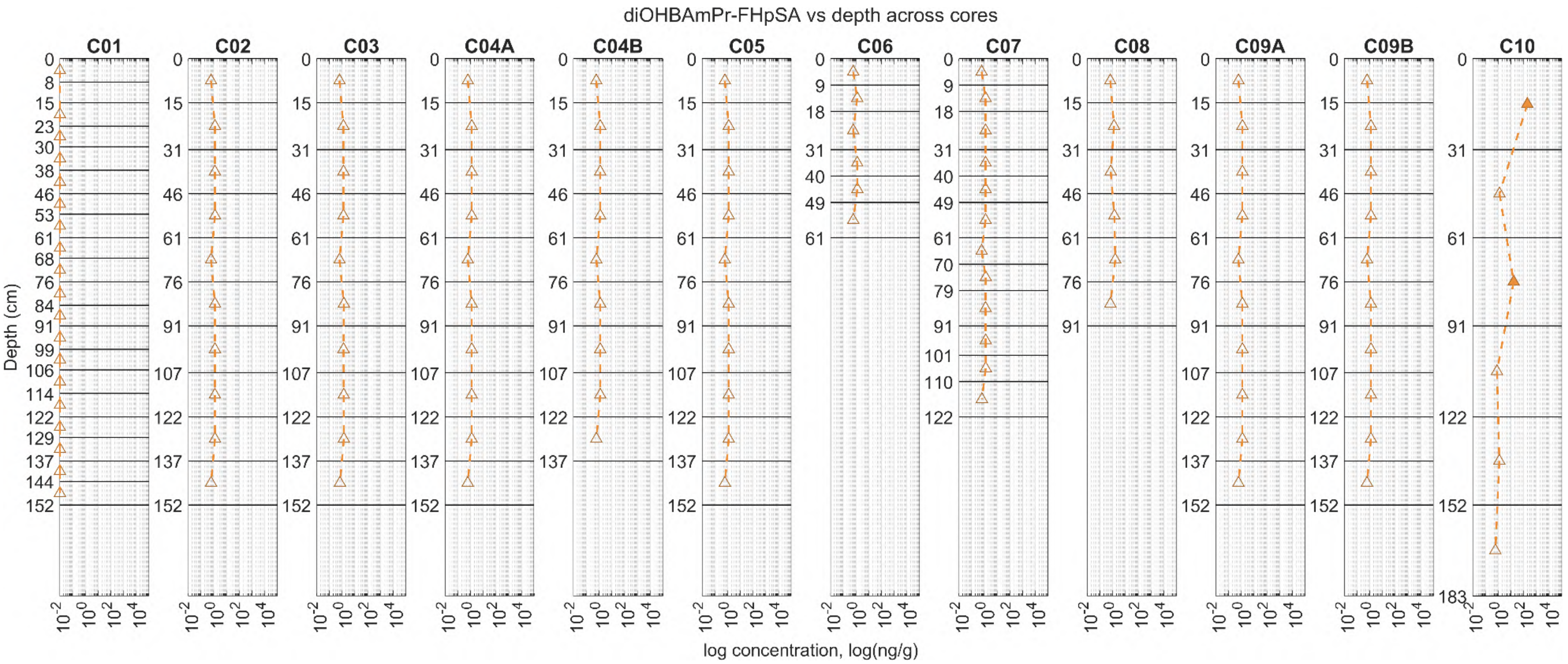

Figure S110. Vertical distribution profile of **diOHBAmPr-FOSA** across the twelve studied cores

Note that the concentration is shown on a log-base-10 scale. For any given plot and compound, open markers with dashed-line connectors represent sampled depth intervals where the compounds' concentration was below the reporting limit – the location of the open marker along the x-axis is representative of those reporting limits.

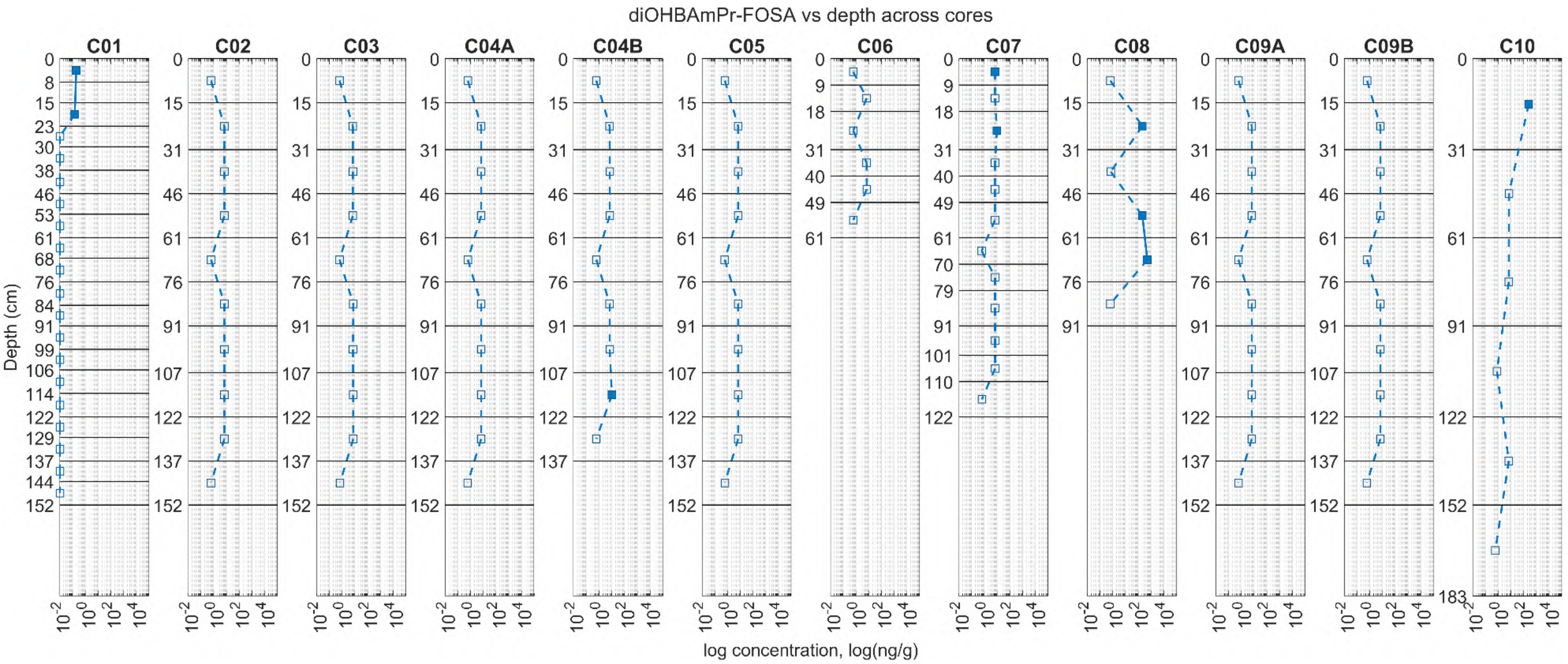

Figure S111. Vertical distribution profile of **6:2 UFTS** across the twelve studied cores

Note that the concentration is shown on a log-base-10 scale. For any given plot and compound, open markers with dashed-line connectors represent sampled depth intervals where the compounds' concentration was below the reporting limit – the location of the open marker along the x-axis is representative of those reporting limits.

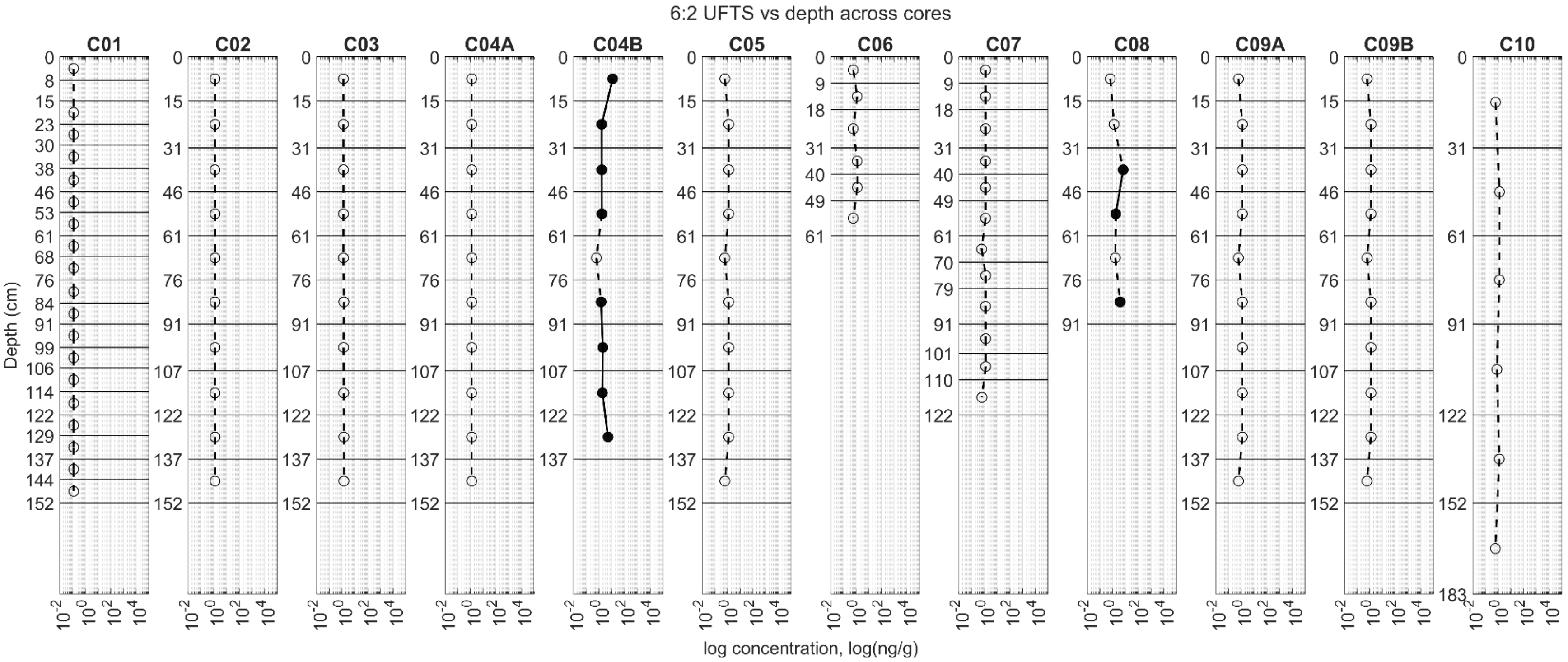

Figure S112. Vertical distribution profile of **6:2 FTSO-PrAd-DiMeEtS** across the twelve studied cores

Note that the concentration is shown on a log-base-10 scale. For any given plot and compound, open markers with dashed-line connectors represent sampled depth intervals where the compounds' concentration was below the reporting limit – the location of the open marker along the x-axis is representative of those reporting limits.

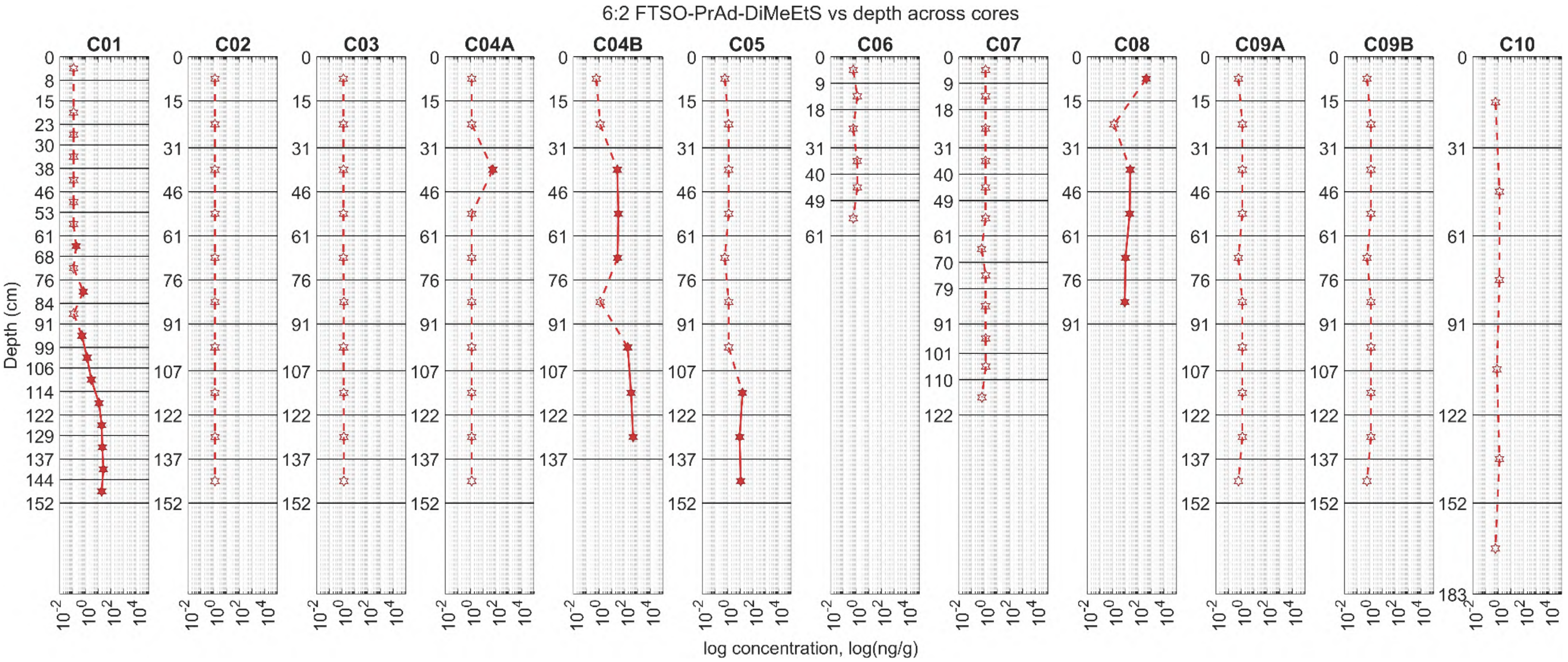

Figure S113. Vertical distribution profile of **8:2 FTSO-PrAd-DiMeEtS** across the twelve studied cores

Note that the concentration is shown on a log-base-10 scale. For any given plot and compound, open markers with dashed-line connectors represent sampled depth intervals where the compounds' concentration was below the reporting limit – the location of the open marker along the x-axis is representative of those reporting limits.

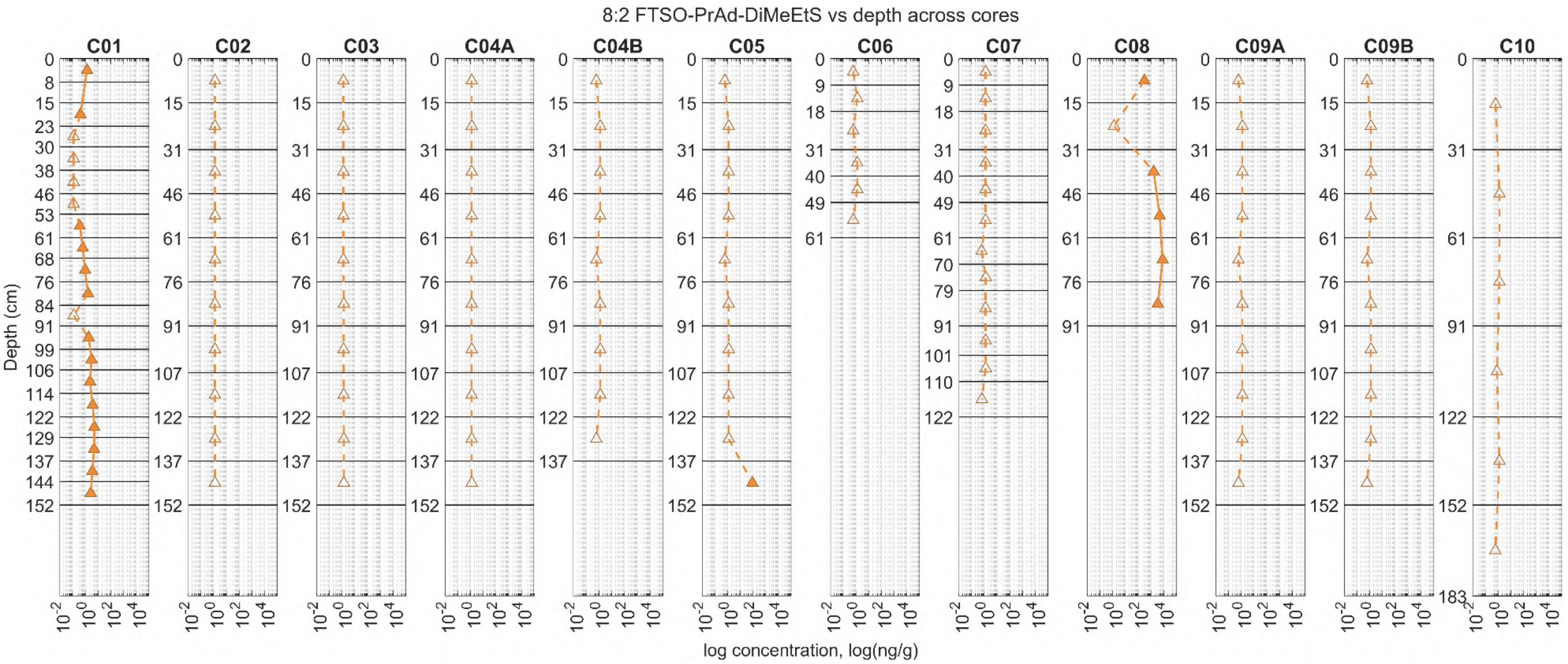

Figure S114. Vertical distribution profile of **6:2 FTSAPr-OHMeMeAn** across the twelve studied cores

Note that the concentration is shown on a log-base-10 scale. For any given plot and compound, open markers with dashed-line connectors represent sampled depth intervals where the compounds' concentration was below the reporting limit – the location of the open marker along the x-axis is representative of those reporting limits.

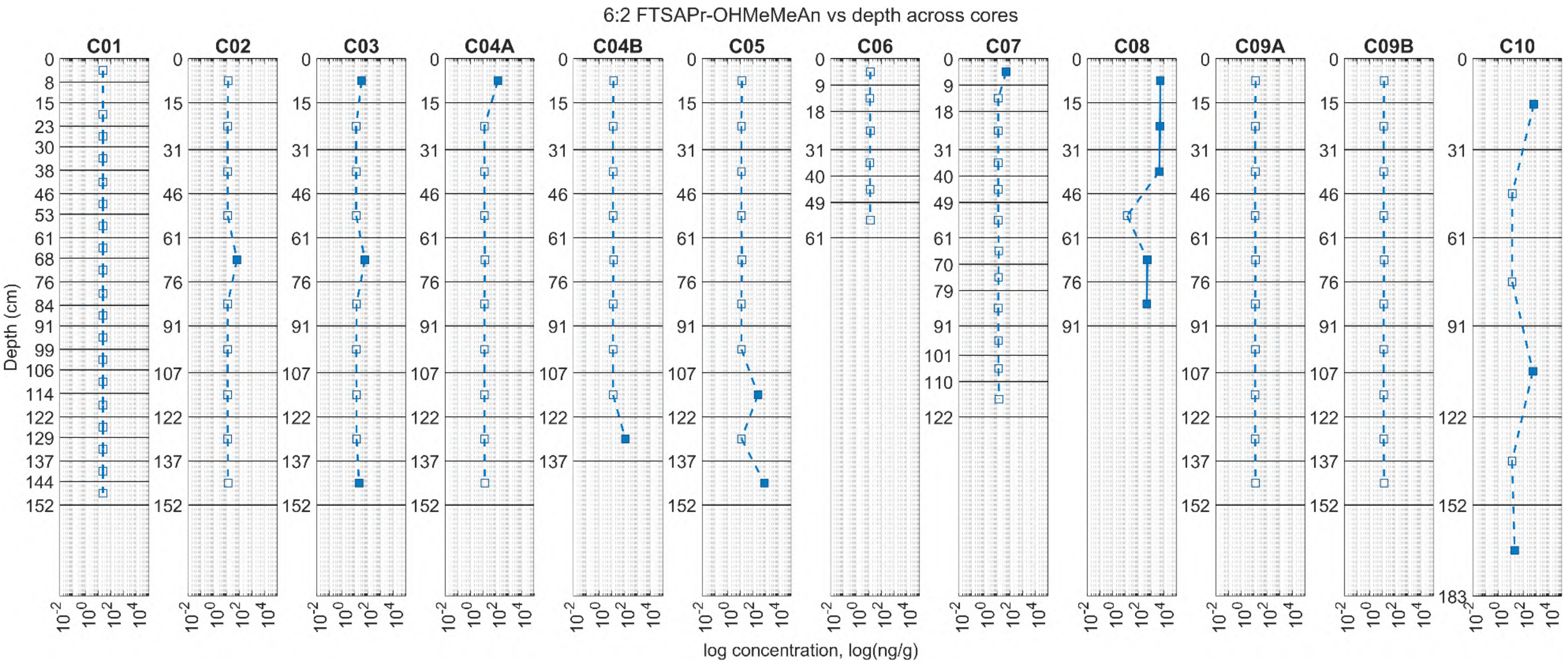

Figure S115. Vertical distribution profile of **EtOH-Am-OHPr-FHxSA** across the twelve studied cores

Note that the concentration is shown on a log-base-10 scale. For any given plot and compound, open markers with dashed-line connectors represent sampled depth intervals where the compounds' concentration was below the reporting limit – the location of the open marker along the x-axis is representative of those reporting limits.

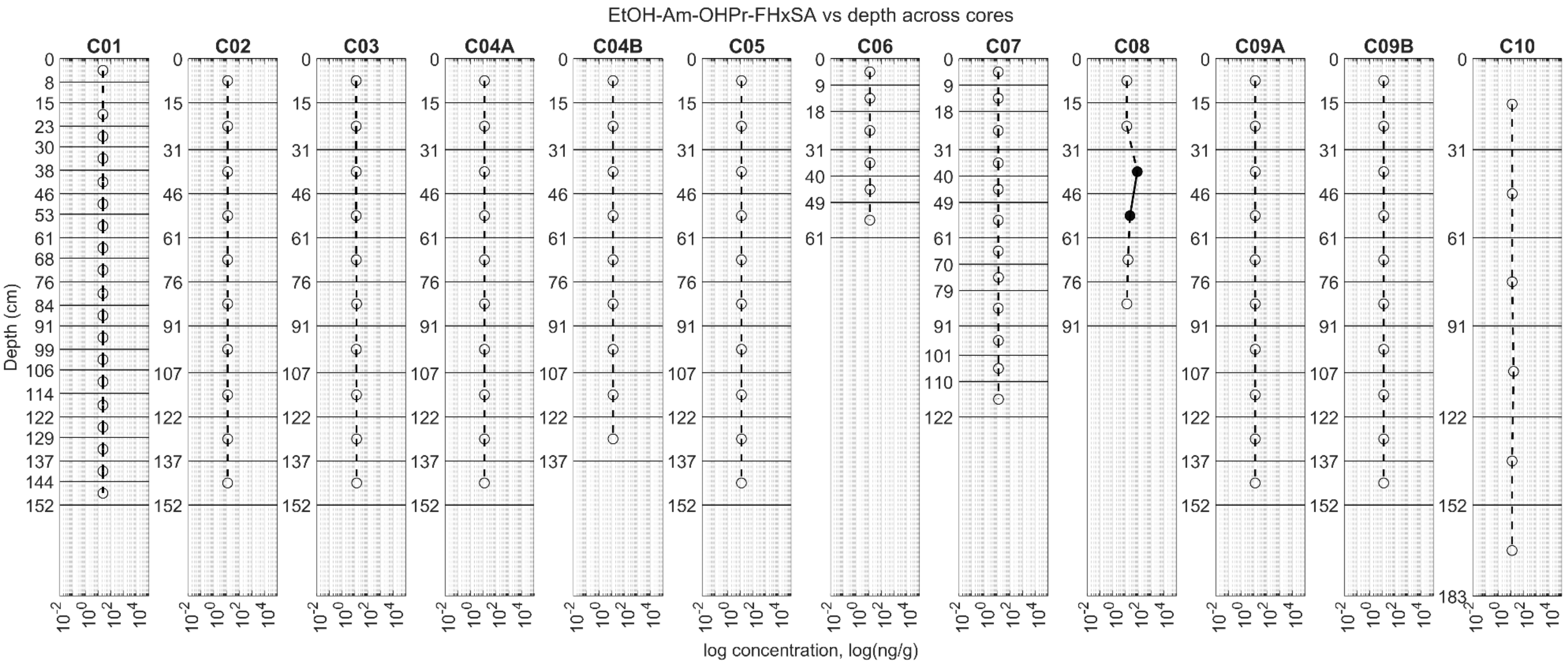

Figure S116. Vertical distribution profile of **EtOH-AmPr-FBSA** across the twelve studied cores

Note that the concentration is shown on a log-base-10 scale. For any given plot and compound, open markers with dashed-line connectors represent sampled depth intervals where the compounds' concentration was below the reporting limit – the location of the open marker along the x-axis is representative of those reporting limits.

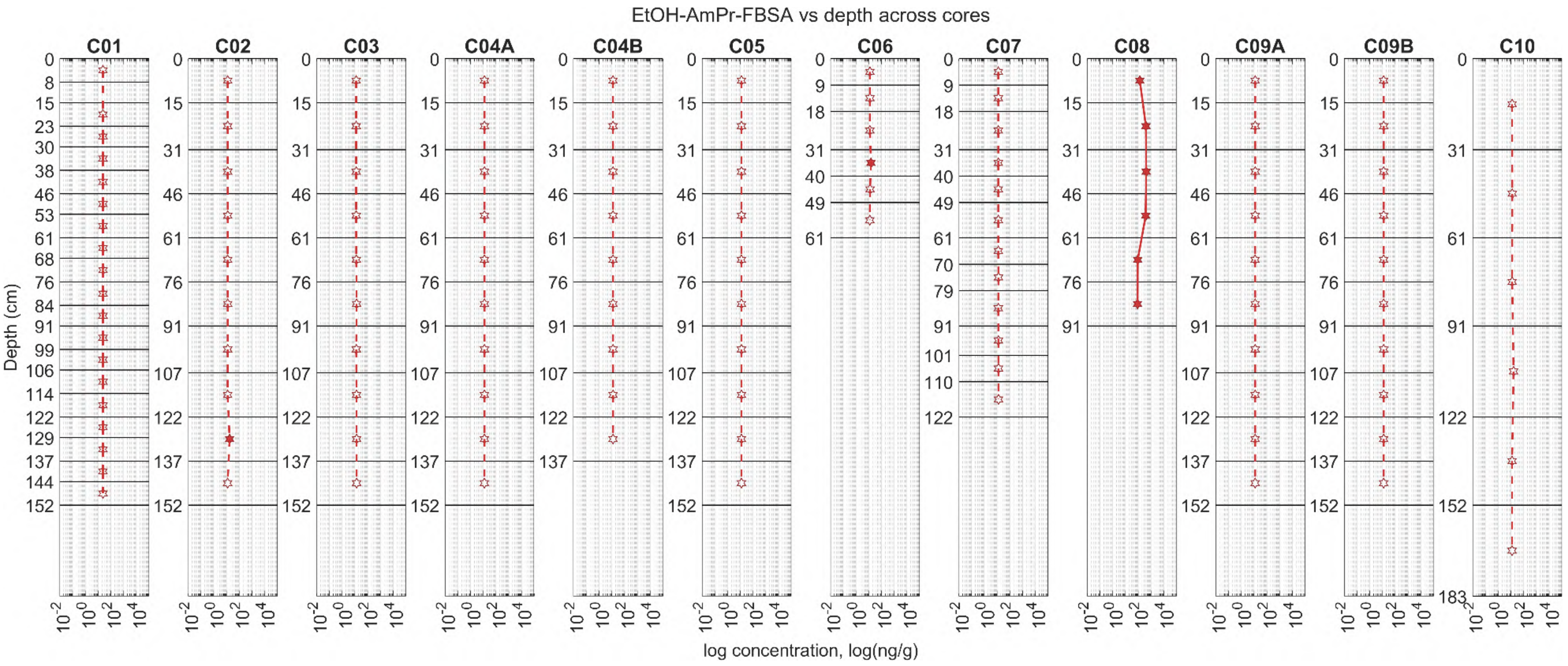

Figure S117. Vertical distribution profile of **EtOH-AmPr-FPeSA** across the twelve studied cores

Note that the concentration is shown on a log-base-10 scale. For any given plot and compound, open markers with dashed-line connectors represent sampled depth intervals where the compounds' concentration was below the reporting limit – the location of the open marker along the x-axis is representative of those reporting limits.

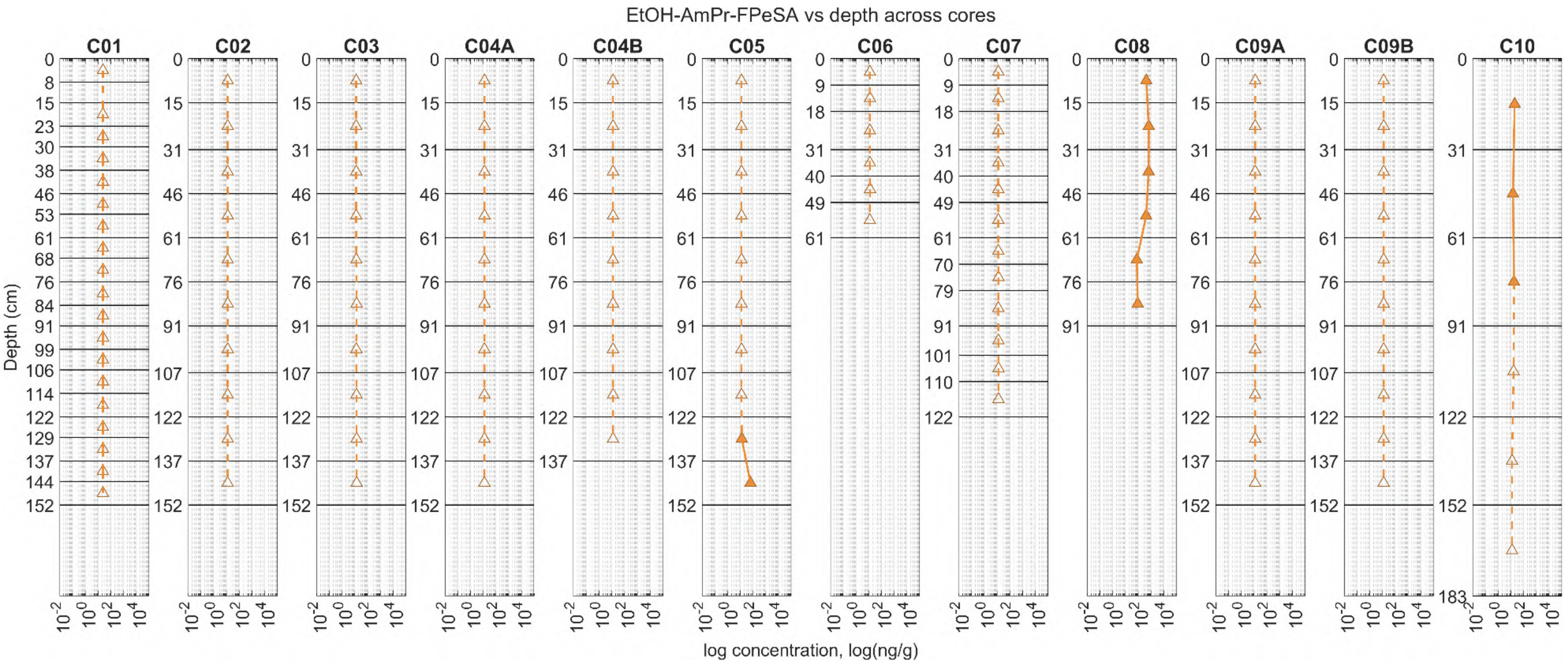

Figure S118. Vertical distribution profile of **EtOH-AmPr-FHxSA** across the twelve studied cores

Note that the concentration is shown on a log-base-10 scale. For any given plot and compound, open markers with dashed-line connectors represent sampled depth intervals where the compounds' concentration was below the reporting limit – the location of the open marker along the x-axis is representative of those reporting limits.

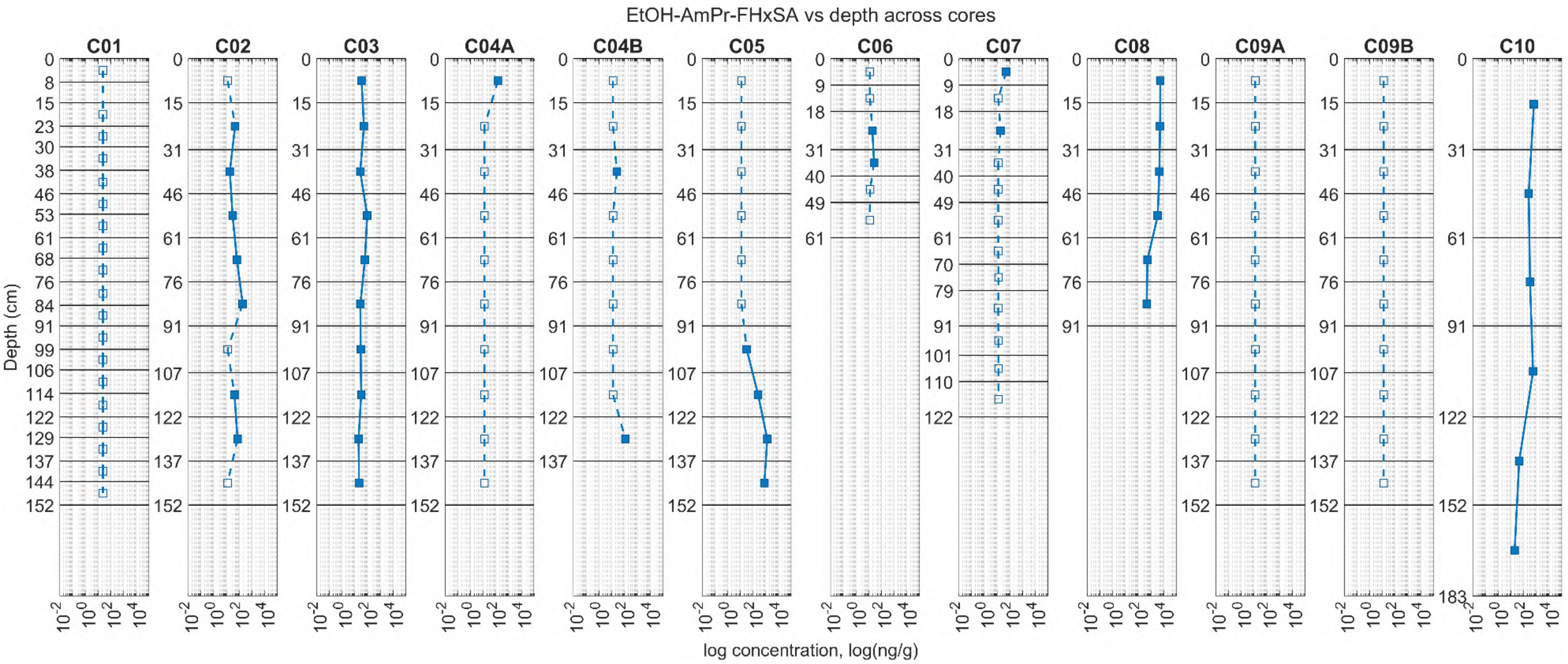

Figure S119. Vertical distribution profile of **EtOH-AmPr-FHpSA** across the twelve studied cores

Note that the concentration is shown on a log-base-10 scale. For any given plot and compound, open markers with dashed-line connectors represent sampled depth intervals where the compounds' concentration was below the reporting limit – the location of the open marker along the x-axis is representative of those reporting limits.

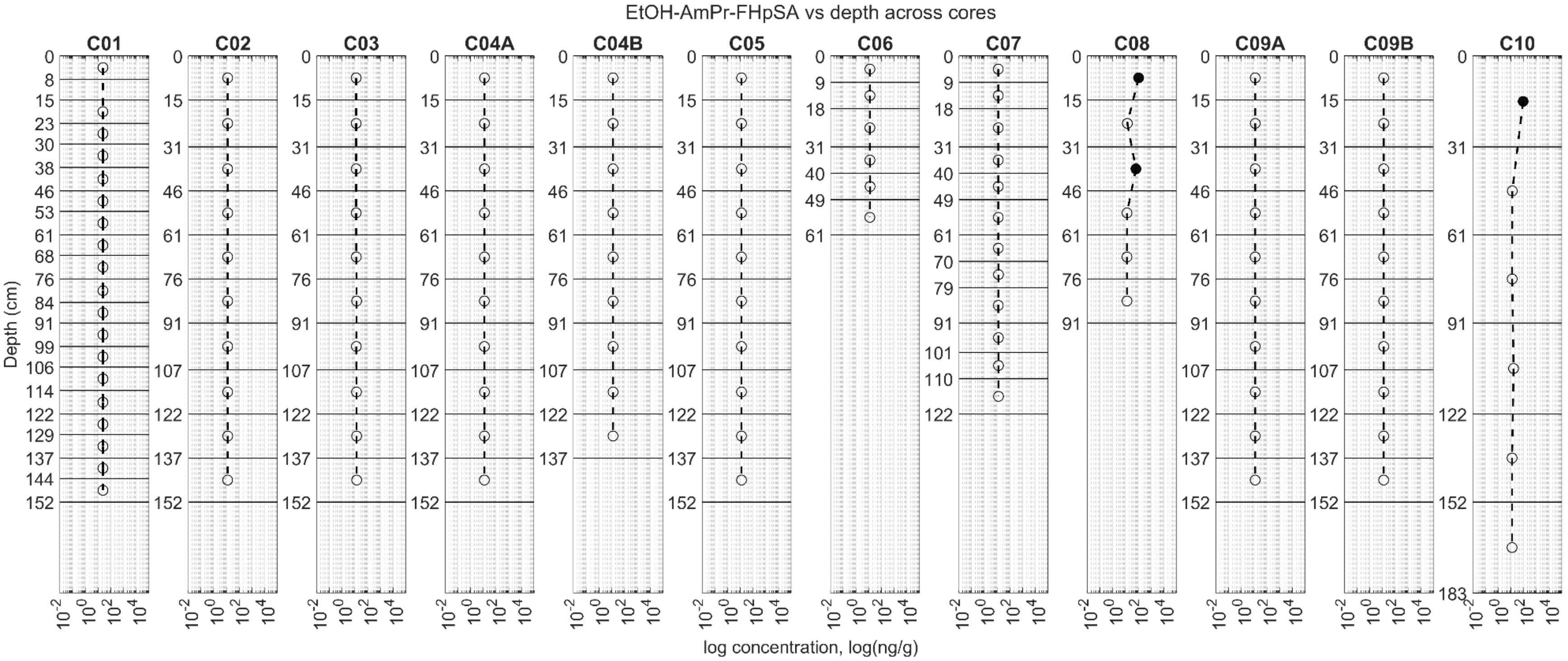

Figure S120. Vertical distribution profile of **EtOH-AmPr-FOSA** across the twelve studied cores

Note that the concentration is shown on a log-base-10 scale. For any given plot and compound, open markers with dashed-line connectors represent sampled depth intervals where the compounds' concentration was below the reporting limit – the location of the open marker along the x-axis is representative of those reporting limits.

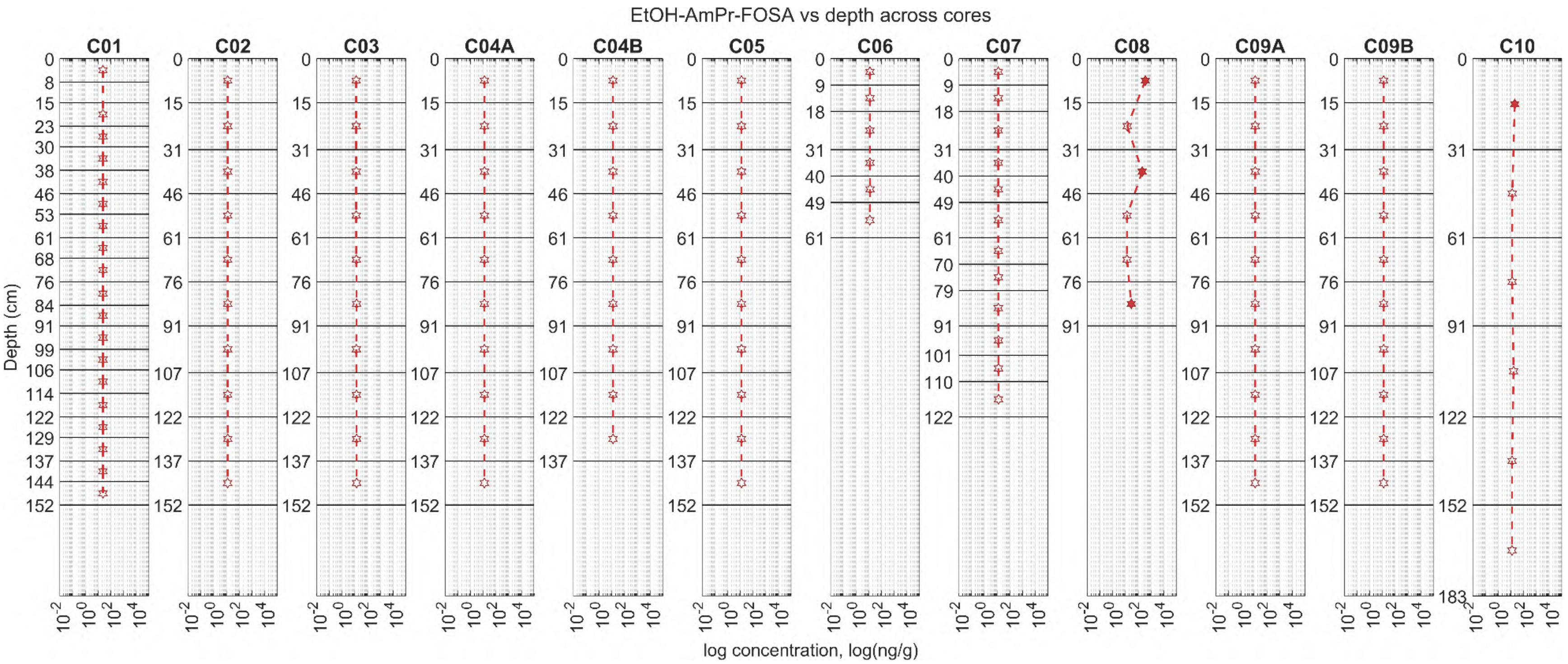

Figure S121. Vertical distribution profile of **EtOH-AmPr-FBSA-OHPrS** across the twelve studied cores

Note that the concentration is shown on a log-base-10 scale. For any given plot and compound, open markers with dashed-line connectors represent sampled depth intervals where the compounds' concentration was below the reporting limit – the location of the open marker along the x-axis is representative of those reporting limits.

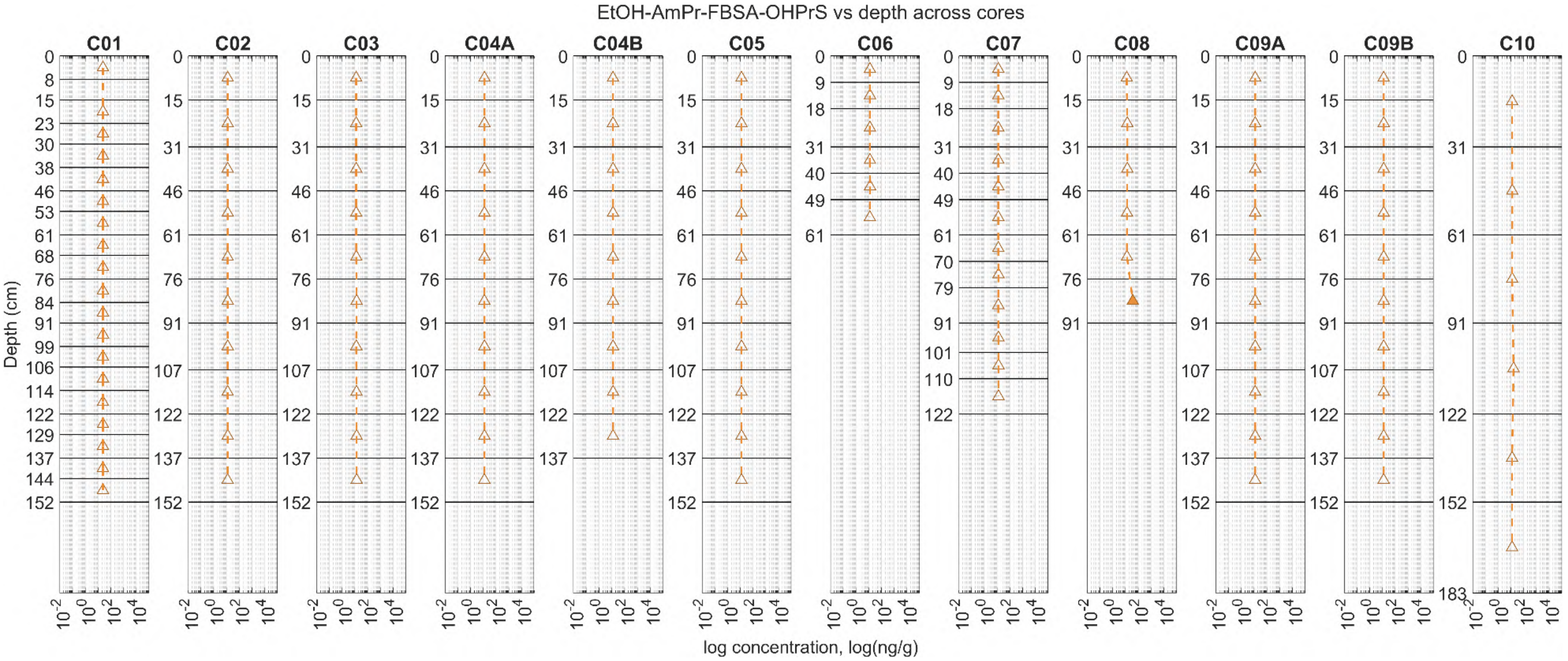

Figure S122. Vertical distribution profile of **EtOH-AmPr-FPeSA-OHPrS** across the twelve studied cores

Note that the concentration is shown on a log-base-10 scale. For any given plot and compound, open markers with dashed-line connectors represent sampled depth intervals where the compounds' concentration was below the reporting limit – the location of the open marker along the x-axis is representative of those reporting limits.

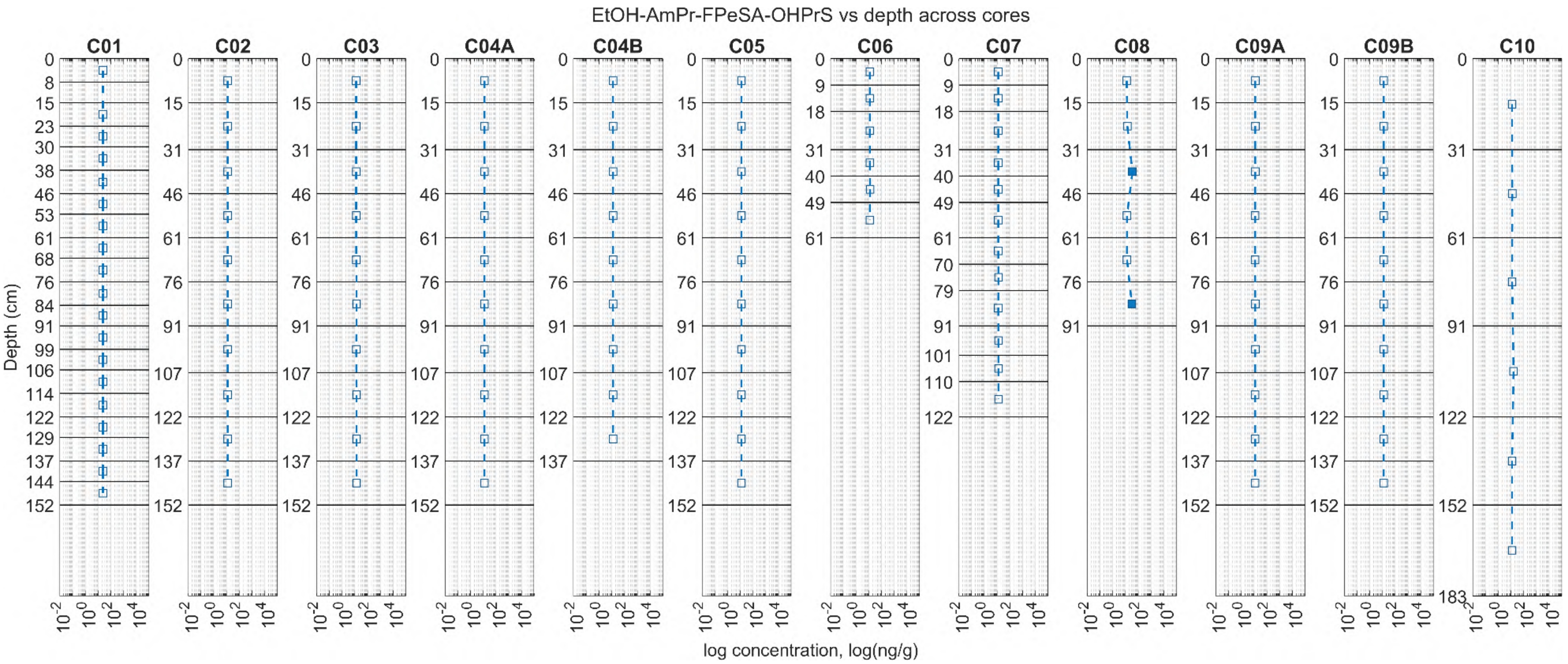

Figure S123. Vertical distribution profile of **EtOH-AmPr-FHxSA-OHPrS** across the twelve studied cores

Note that the concentration is shown on a log-base-10 scale. For any given plot and compound, open markers with dashed-line connectors represent sampled depth intervals where the compounds' concentration was below the reporting limit – the location of the open marker along the x-axis is representative of those reporting limits.

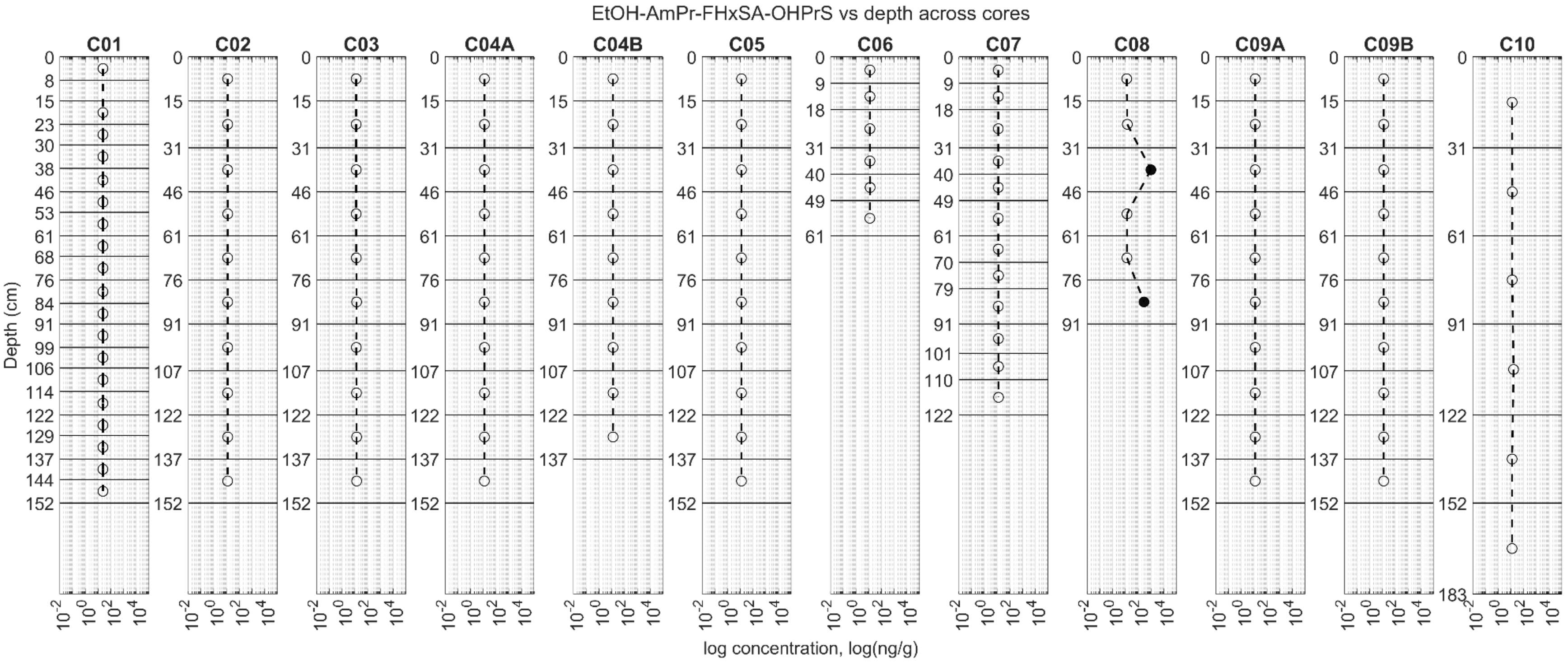

Figure S124. Vertical distribution profile of **SPrAmPr-N-Me-FHxSA** across the twelve studied cores

Note that the concentration is shown on a log-base-10 scale. For any given plot and compound, open markers with dashed-line connectors represent sampled depth intervals where the compounds' concentration was below the reporting limit – the location of the open marker along the x-axis is representative of those reporting limits.

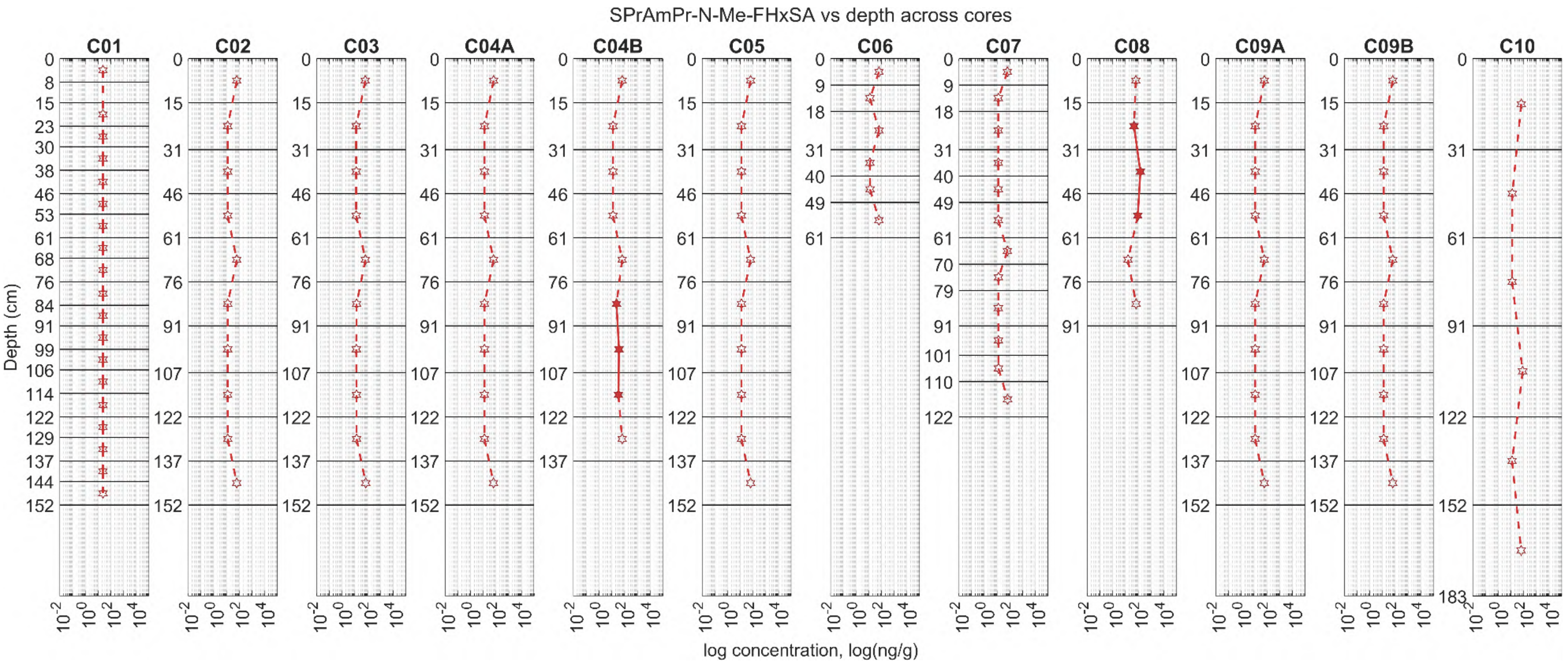

Figure S125. Vertical distribution profile of **EtOH-AmPr-FHxSAPrS** across the twelve studied cores

Note that the concentration is shown on a log-base-10 scale. For any given plot and compound, open markers with dashed-line connectors represent sampled depth intervals where the compounds' concentration was below the reporting limit – the location of the open marker along the x-axis is representative of those reporting limits.

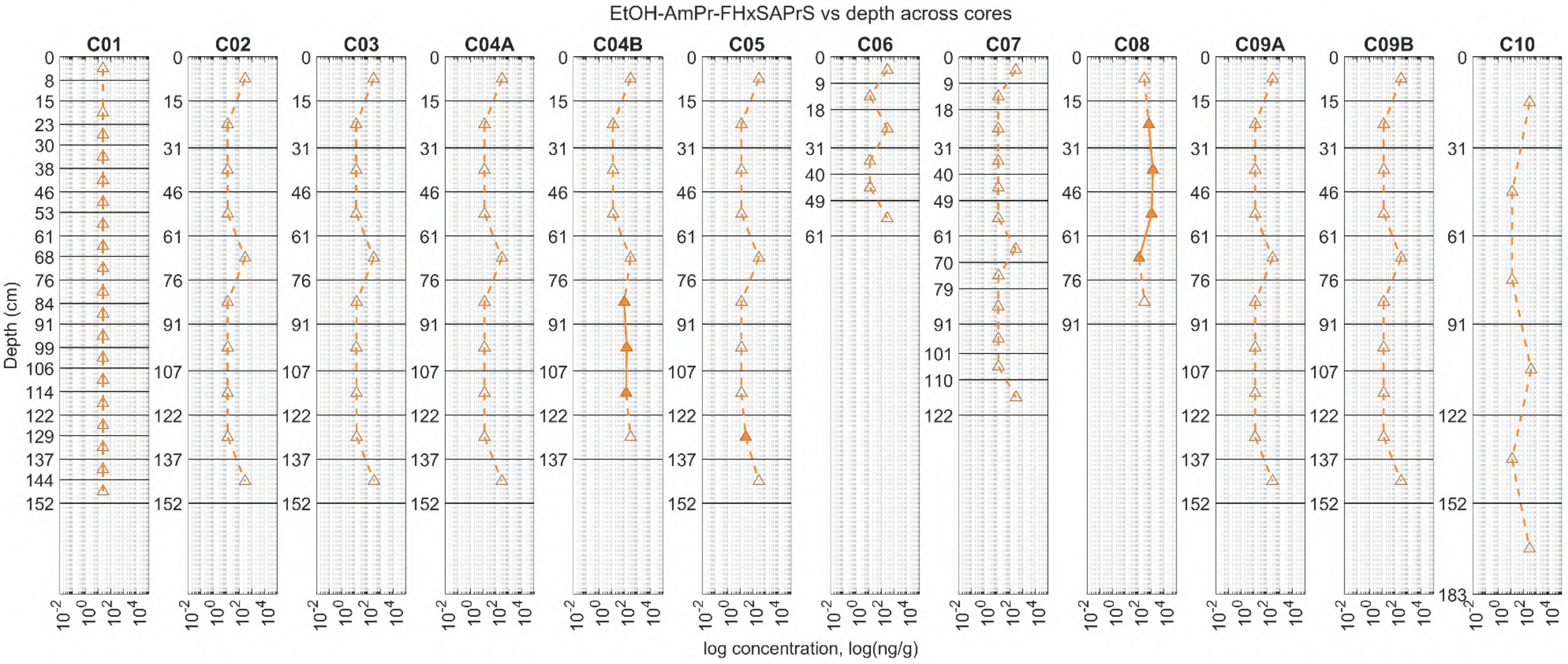

Figure S126. Vertical distribution profile of **6:2 FTSA-PrB (6:2 FTAB)** across the twelve studied cores

Note that the concentration is shown on a log-base-10 scale. For any given plot and compound, open markers with dashed-line connectors represent sampled depth intervals where the compounds' concentration was below the reporting limit – the location of the open marker along the x-axis is representative of those reporting limits.

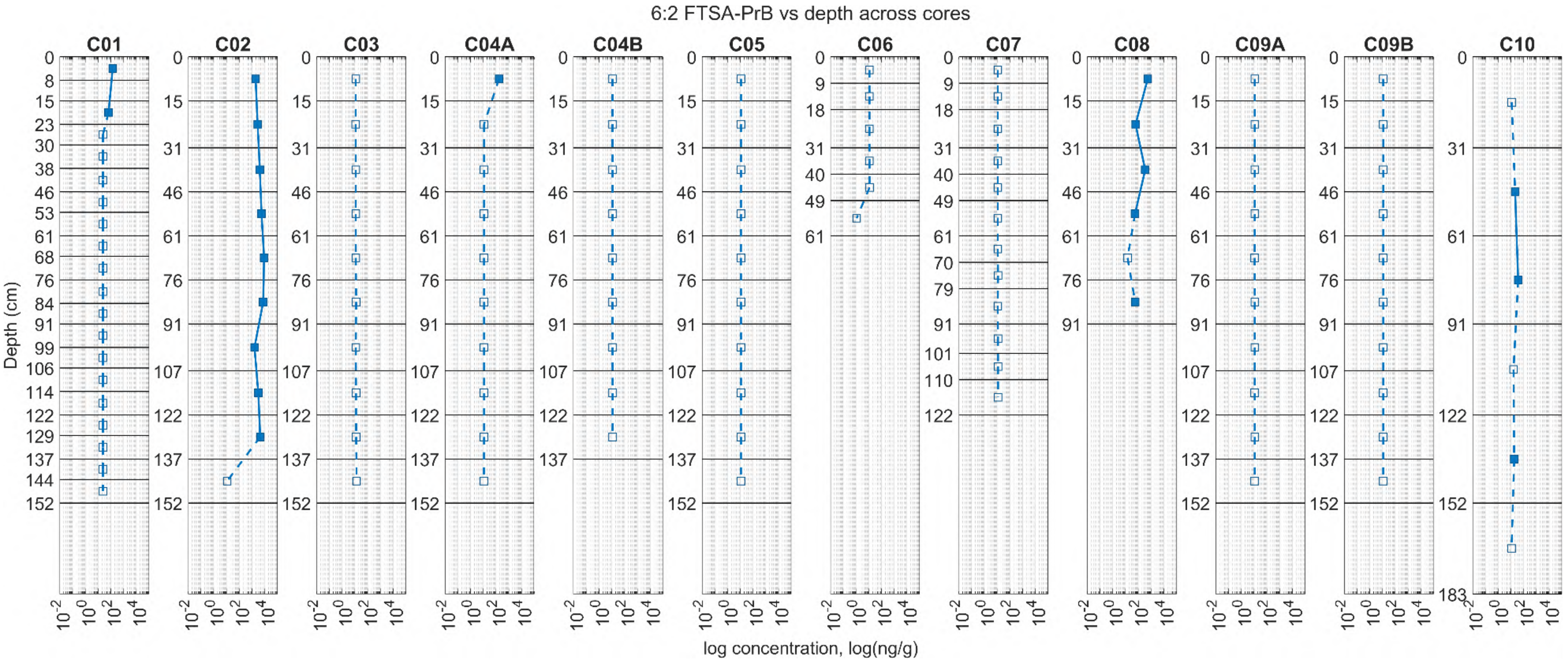

Figure S127. Vertical distribution profile of **8:2 FTSA-PrB (8:2 FTAB)** across the twelve studied cores

Note that the concentration is shown on a log-base-10 scale. For any given plot and compound, open markers with dashed-line connectors represent sampled depth intervals where the compounds' concentration was below the reporting limit – the location of the open marker along the x-axis is representative of those reporting limits.

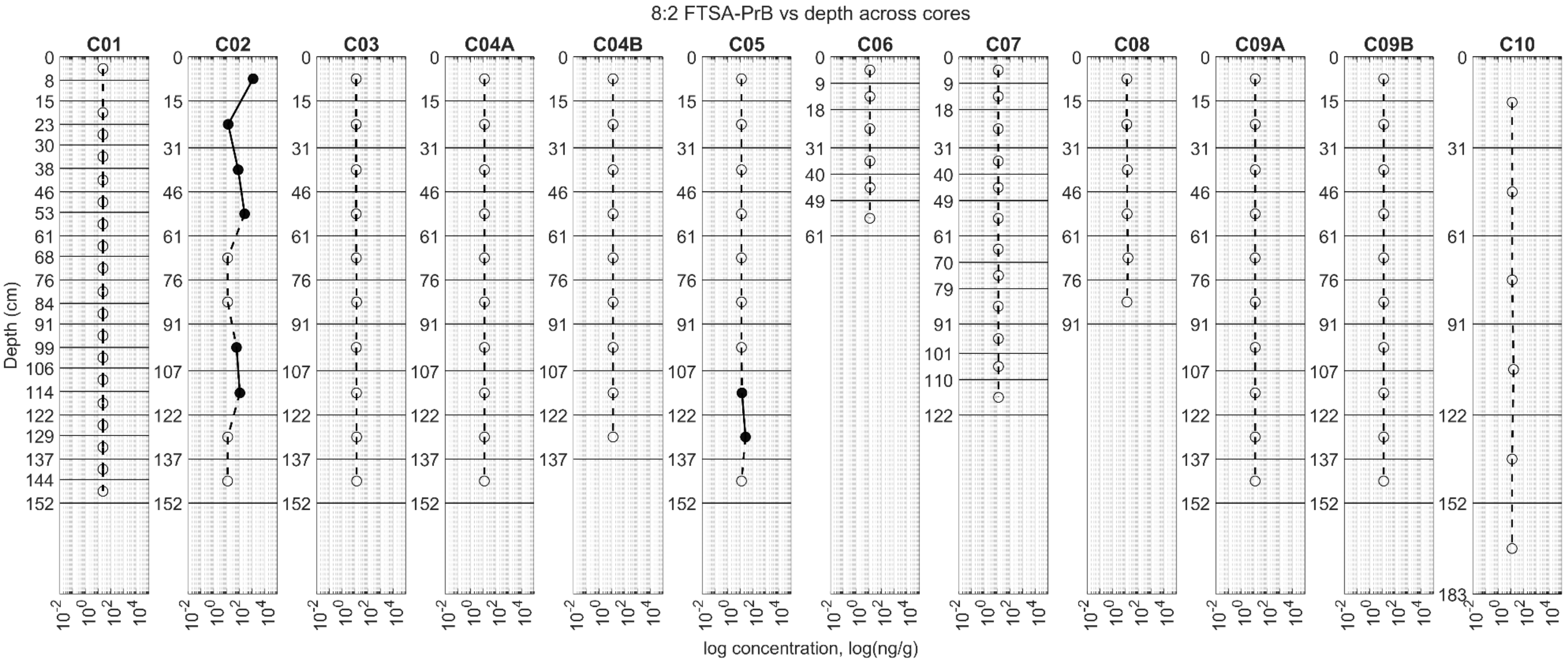

Figure S128. Vertical distribution profile of **TAmPr-N-MeFBSA** across the twelve studied cores

Note that the concentration is shown on a log-base-10 scale. For any given plot and compound, open markers with dashed-line connectors represent sampled depth intervals where the compounds' concentration was below the reporting limit – the location of the open marker along the x-axis is representative of those reporting limits.

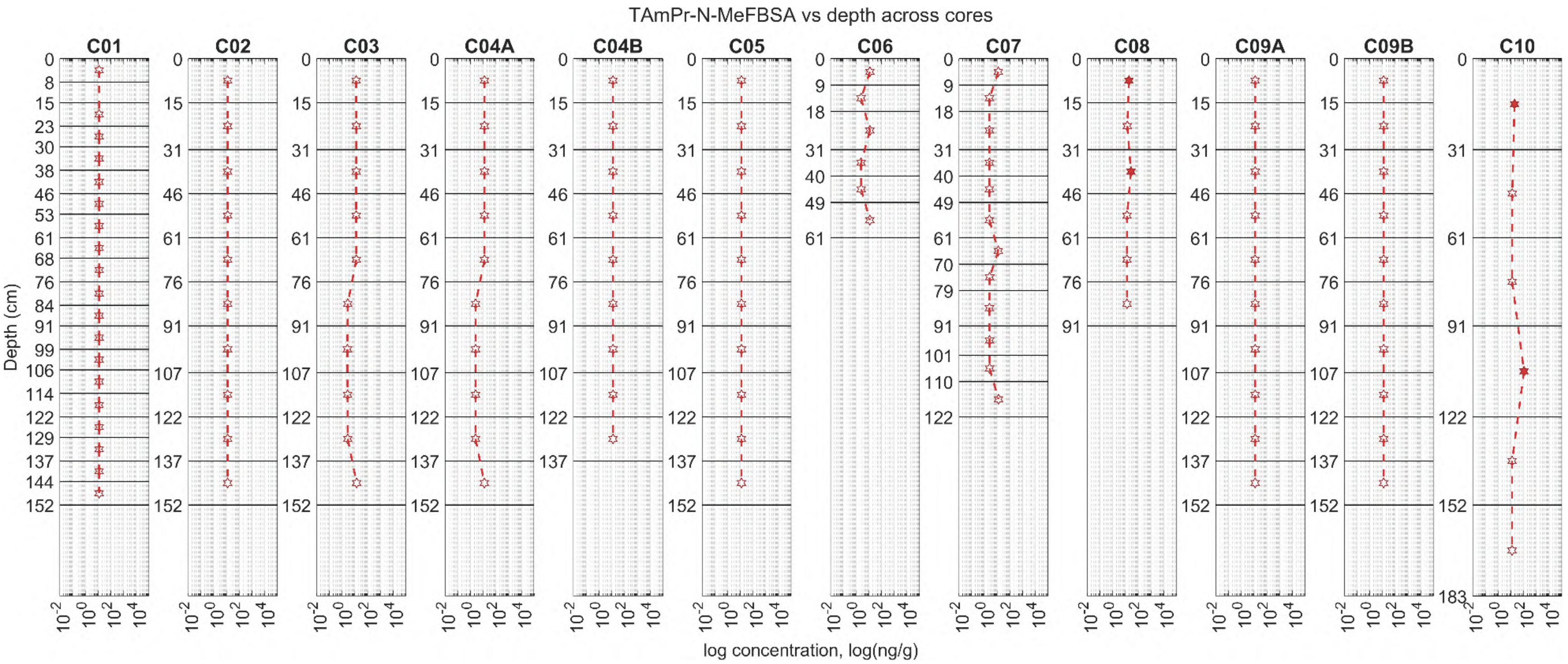

Figure S129. Vertical distribution profile of **TAmPr-N-MeFPeSA** across the twelve studied cores

Note that the concentration is shown on a log-base-10 scale. For any given plot and compound, open markers with dashed-line connectors represent sampled depth intervals where the compounds' concentration was below the reporting limit – the location of the open marker along the x-axis is representative of those reporting limits.

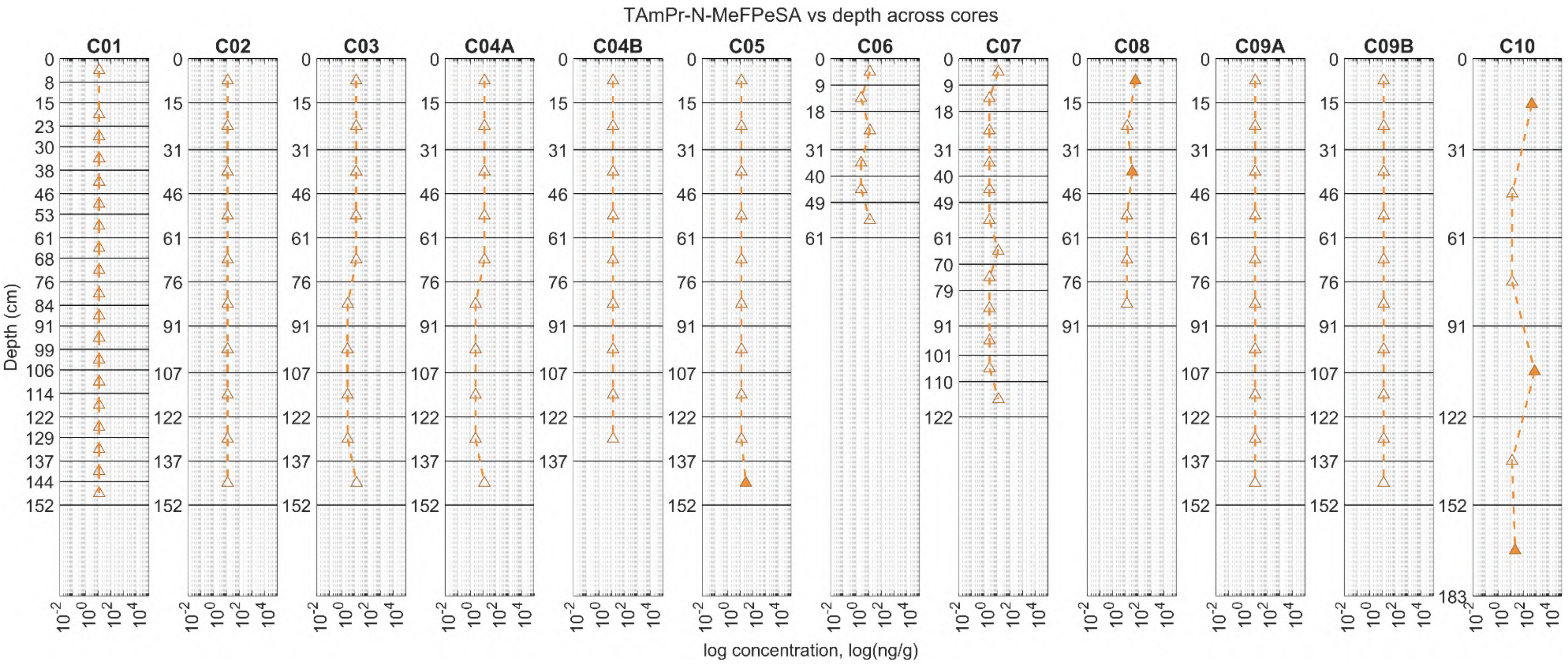

Figure S130. Vertical distribution profile of **TAmPr-N-MeFHxSA** across the twelve studied cores

Note that the concentration is shown on a log-base-10 scale. For any given plot and compound, open markers with dashed-line connectors represent sampled depth intervals where the compounds' concentration was below the reporting limit – the location of the open marker along the x-axis is representative of those reporting limits.

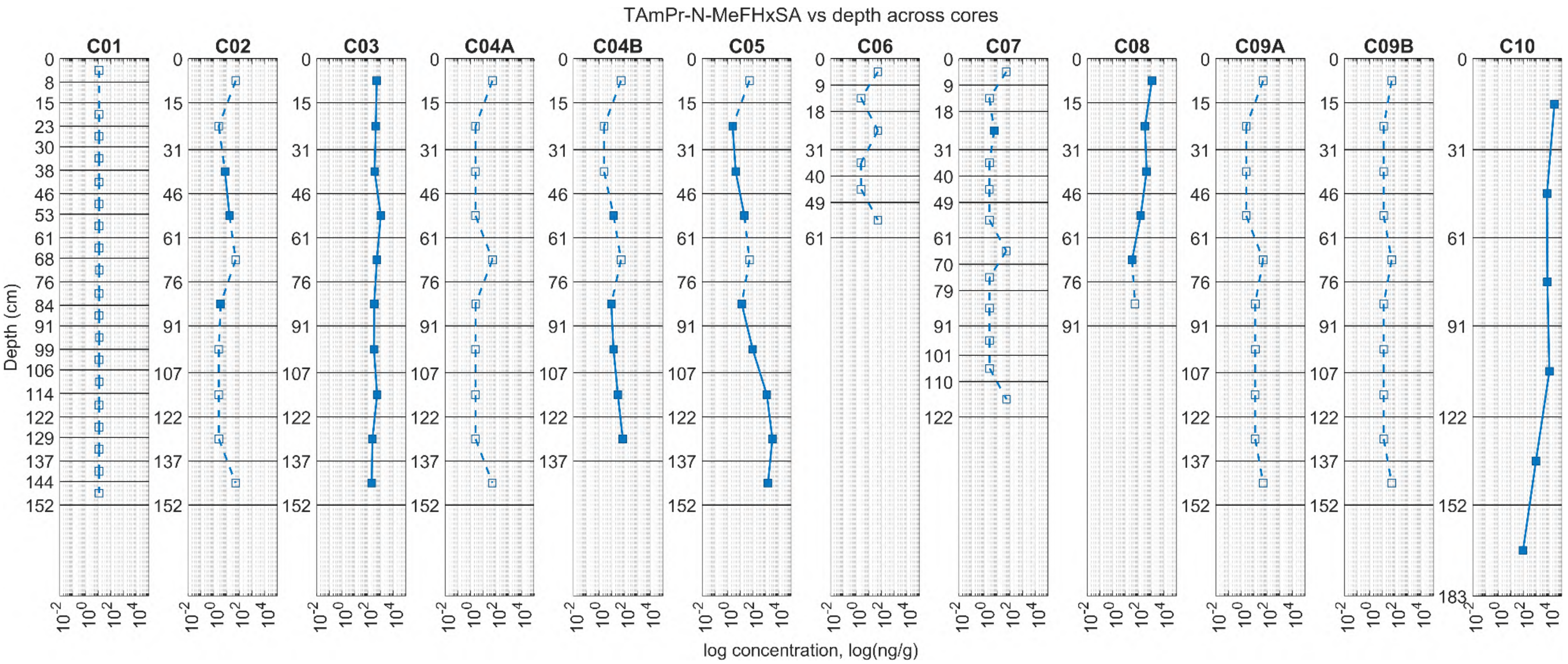

Figure S131. Vertical distribution profile of **TAmPr-N-MeFOSA** across the twelve studied cores

Note that the concentration is shown on a log-base-10 scale. For any given plot and compound, open markers with dashed-line connectors represent sampled depth intervals where the compounds' concentration was below the reporting limit – the location of the open marker along the x-axis is representative of those reporting limits.

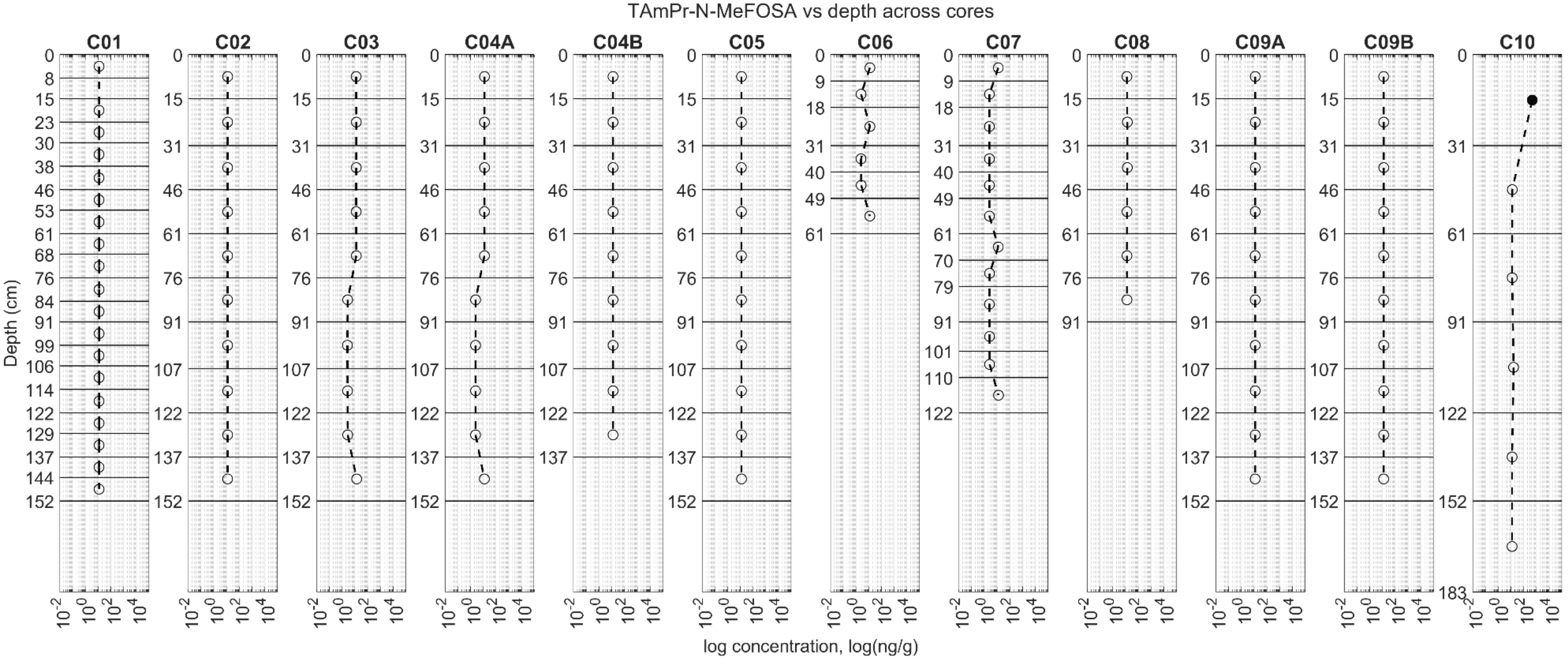

Figure S132. Vertical distribution profile of **TAmPr-FBSA** across the twelve studied cores

Note that the concentration is shown on a log-base-10 scale. For any given plot and compound, open markers with dashed-line connectors represent sampled depth intervals where the compounds' concentration was below the reporting limit – the location of the open marker along the x-axis is representative of those reporting limits.

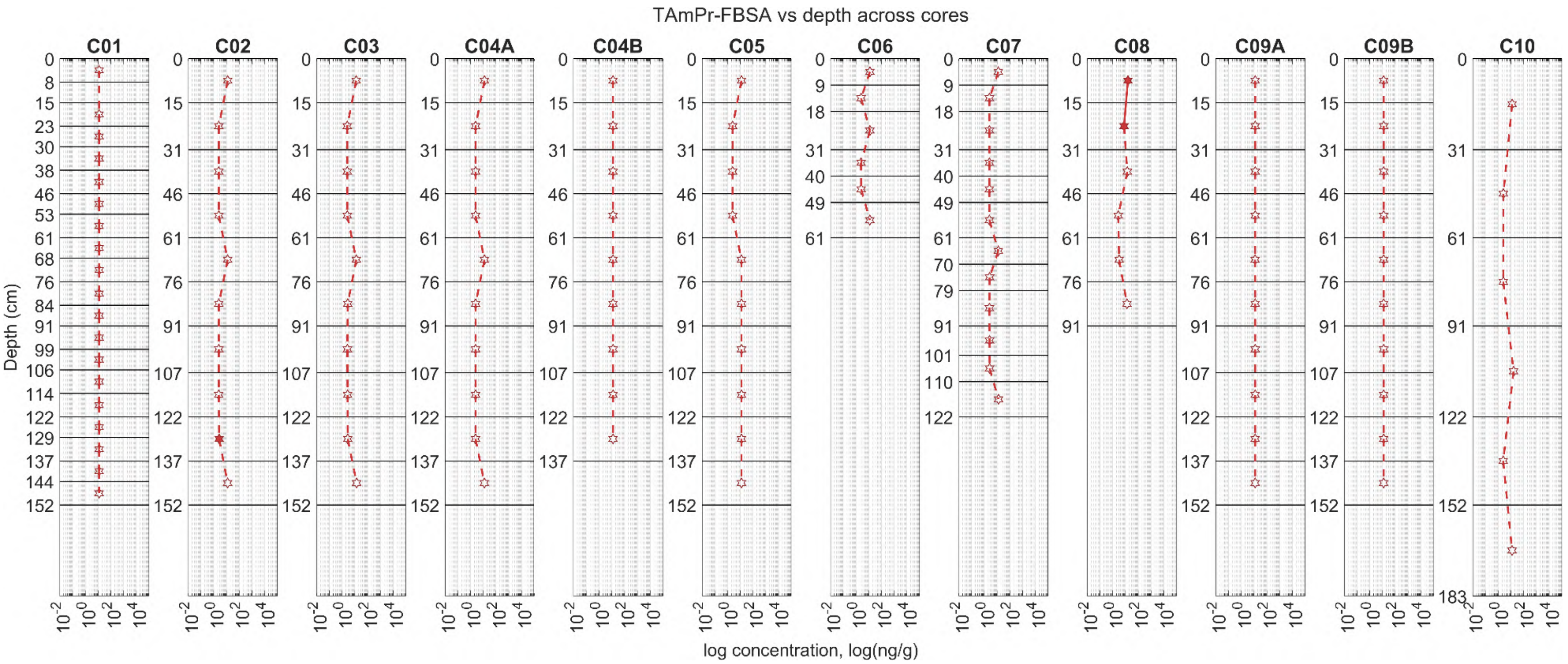

Figure S133. Vertical distribution profile of **TAmPr-FPeSA** across the twelve studied cores

Note that the concentration is shown on a log-base-10 scale. For any given plot and compound, open markers with dashed-line connectors represent sampled depth intervals where the compounds' concentration was below the reporting limit – the location of the open marker along the x-axis is representative of those reporting limits.

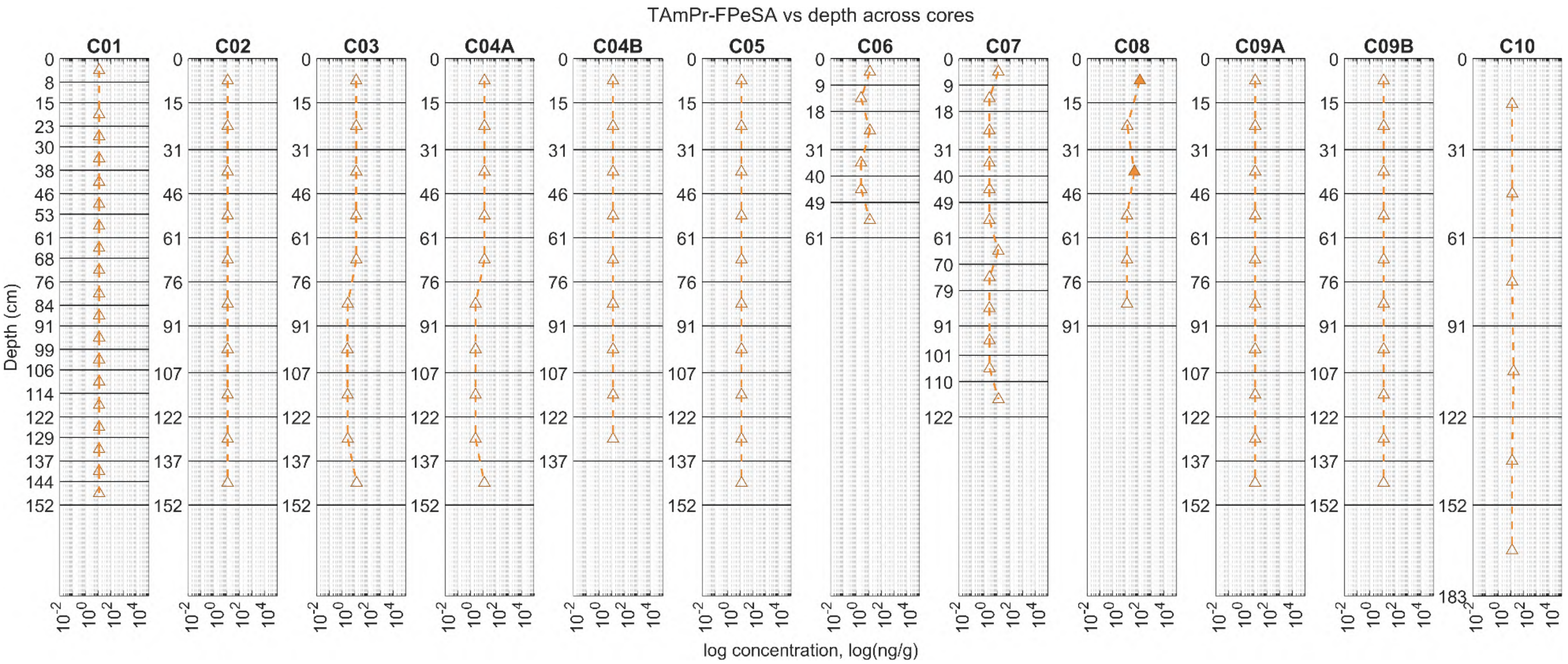

Figure S134. Vertical distribution profile of **TAmPr-FHxSA** across the twelve studied cores

Note that the concentration is shown on a log-base-10 scale. For any given plot and compound, open markers with dashed-line connectors represent sampled depth intervals where the compounds' concentration was below the reporting limit – the location of the open marker along the x-axis is representative of those reporting limits.

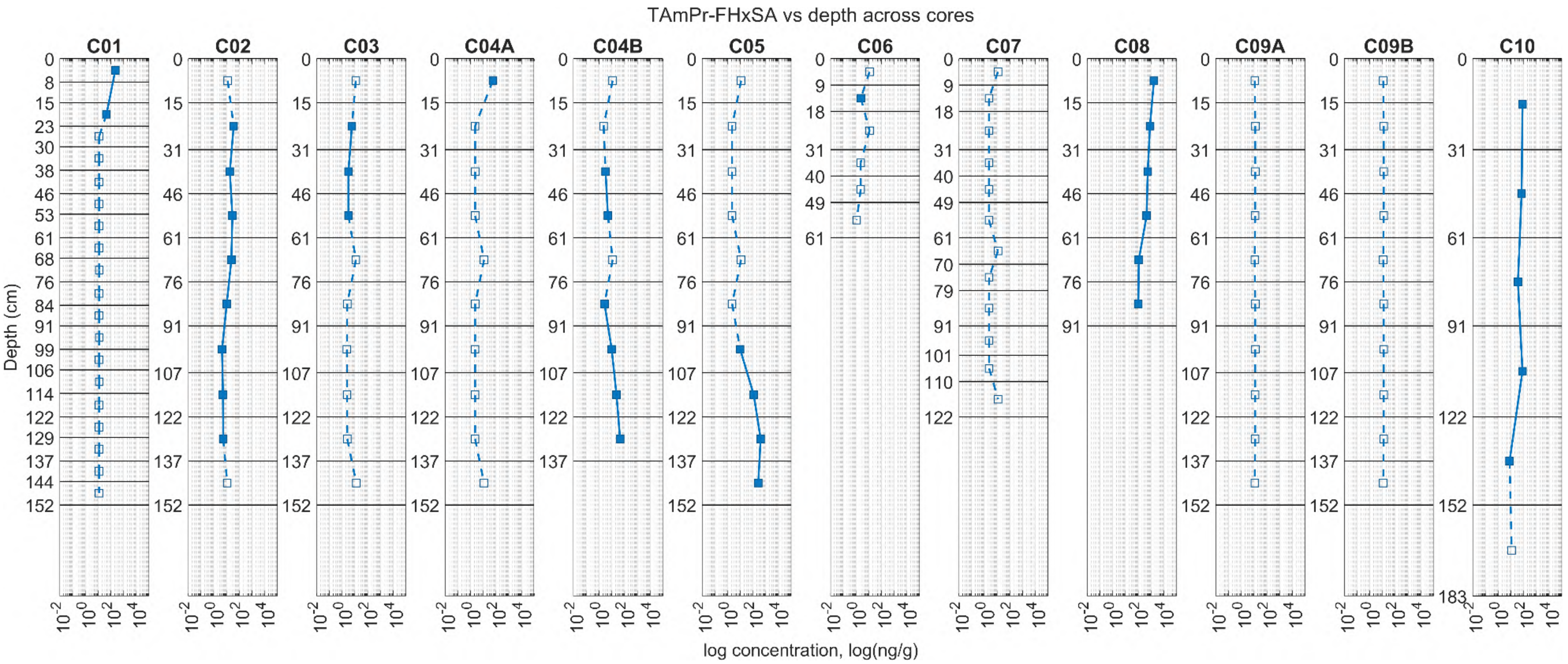

Figure S135. Vertical distribution profile of **TAmPr-FHpSA** across the twelve studied cores

Note that the concentration is shown on a log-base-10 scale. For any given plot and compound, open markers with dashed-line connectors represent sampled depth intervals where the compounds' concentration was below the reporting limit – the location of the open marker along the x-axis is representative of those reporting limits.

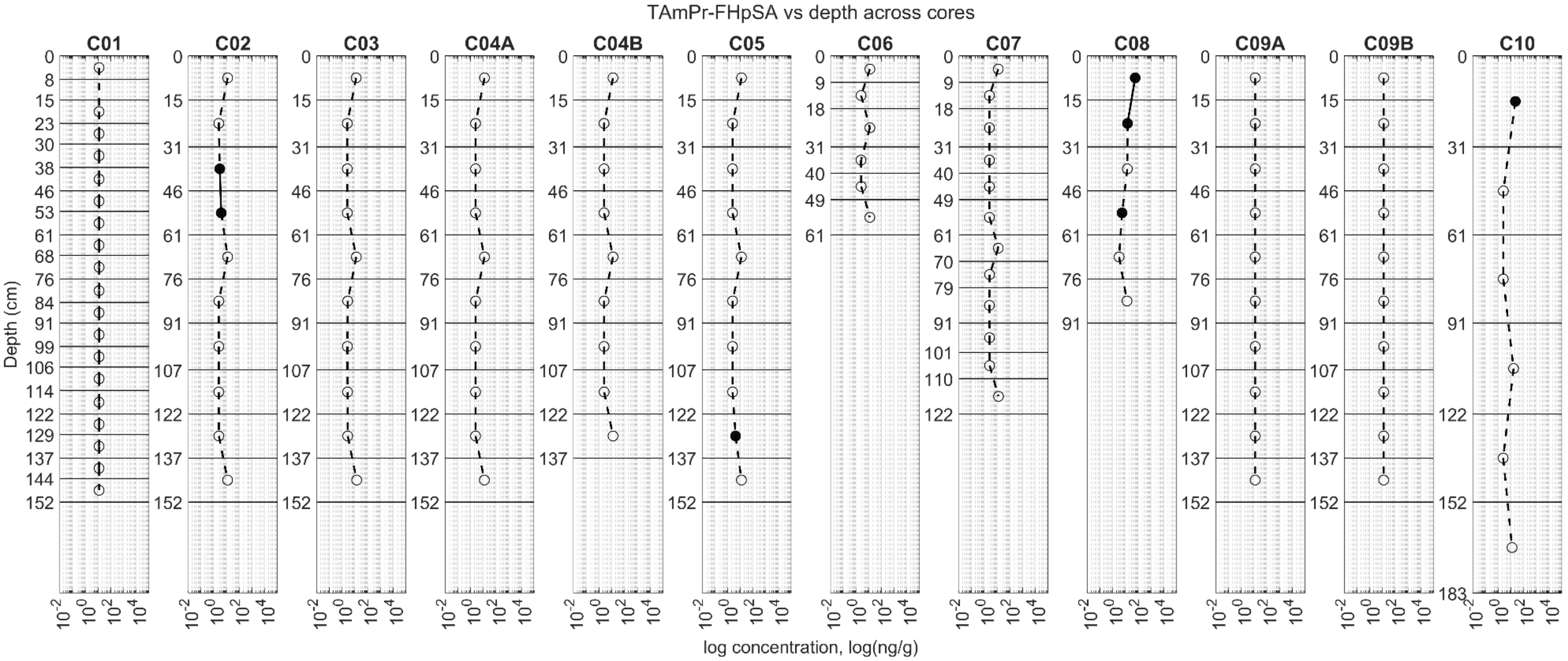

Figure S136. Vertical distribution profile of **TAmPr-FOSA** across the twelve studied cores

Note that the concentration is shown on a log-base-10 scale. For any given plot and compound, open markers with dashed-line connectors represent sampled depth intervals where the compounds' concentration was below the reporting limit – the location of the open marker along the x-axis is representative of those reporting limits.

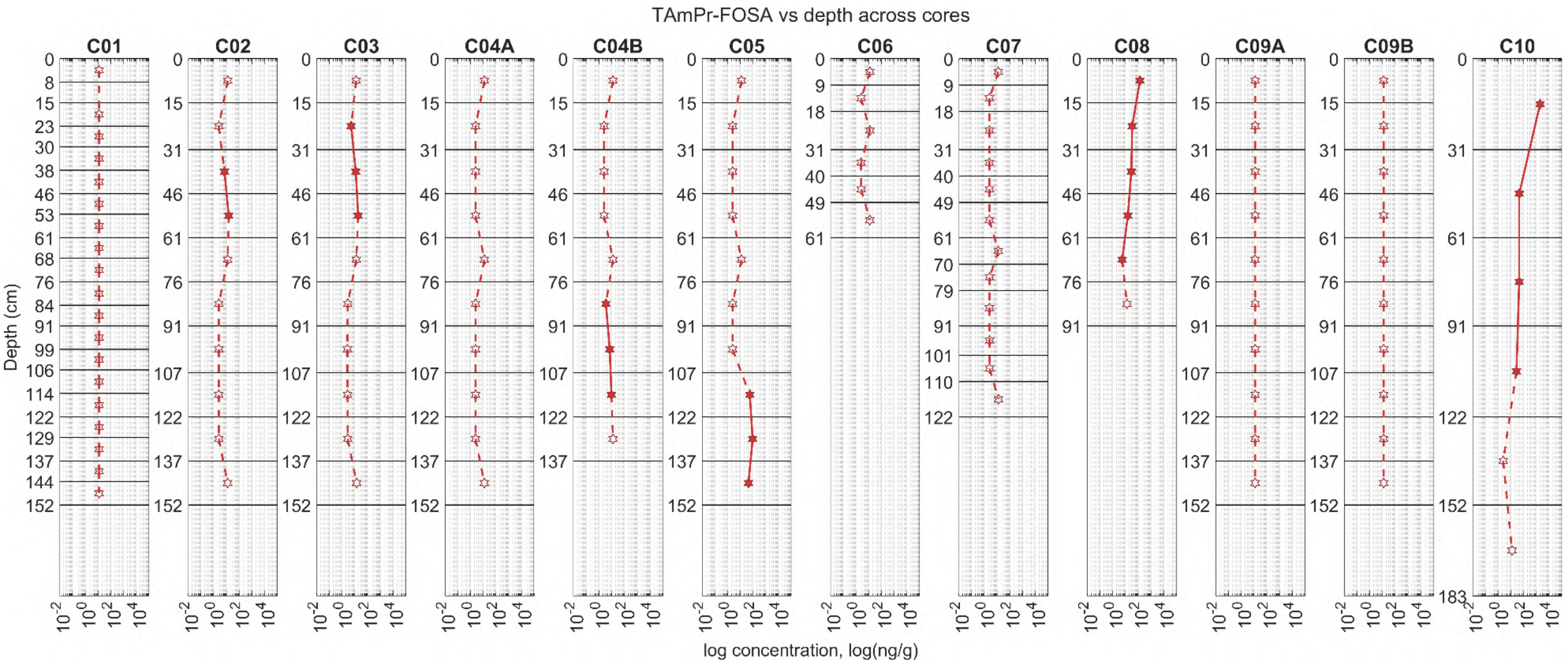

Figure S137. Vertical distribution profile of **EtAmPr-FEt-N-EtSA** across the twelve studied cores

Note that the concentration is shown on a log-base-10 scale. For any given plot and compound, open markers with dashed-line connectors represent sampled depth intervals where the compounds' concentration was below the reporting limit – the location of the open marker along the x-axis is representative of those reporting limits.

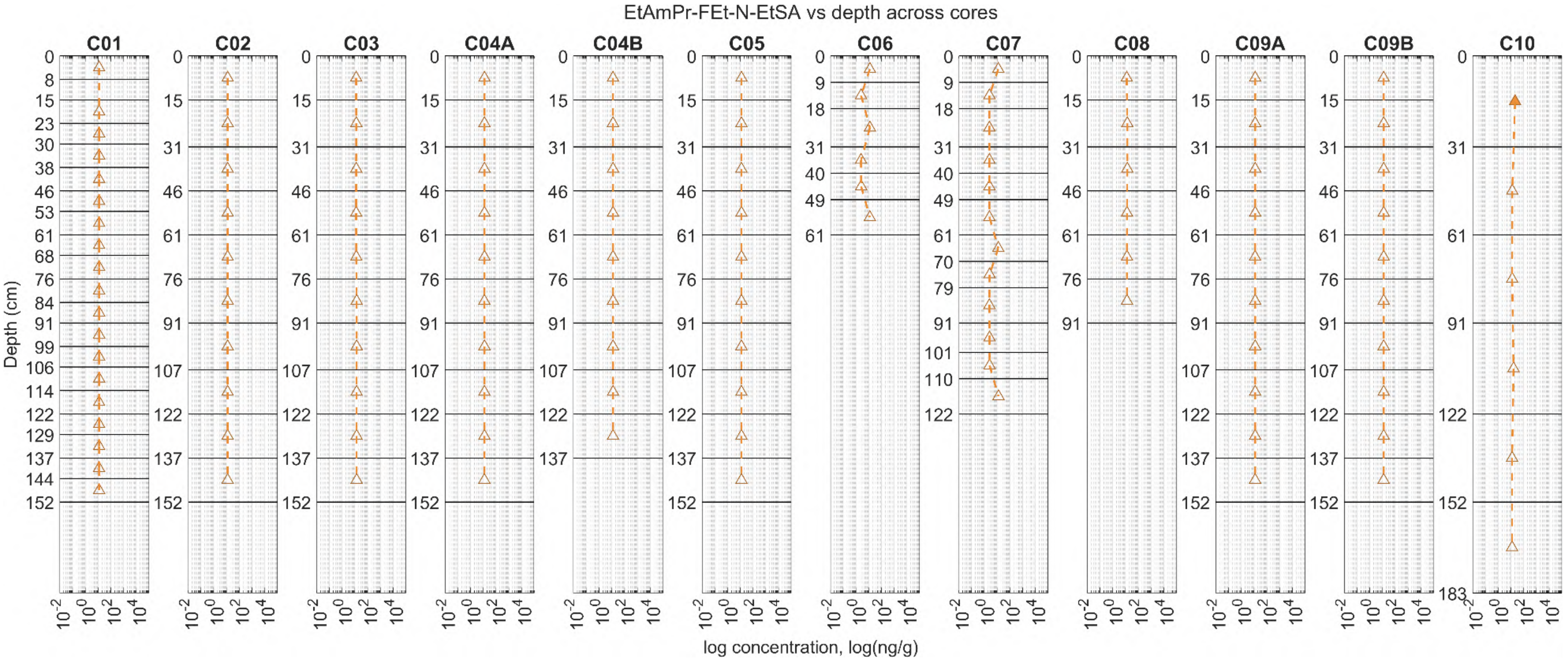

Figure S138. Vertical distribution profile of **EtAmPr-FPr-N-EtSA** across the twelve studied cores

Note that the concentration is shown on a log-base-10 scale. For any given plot and compound, open markers with dashed-line connectors represent sampled depth intervals where the compounds' concentration was below the reporting limit – the location of the open marker along the x-axis is representative of those reporting limits.

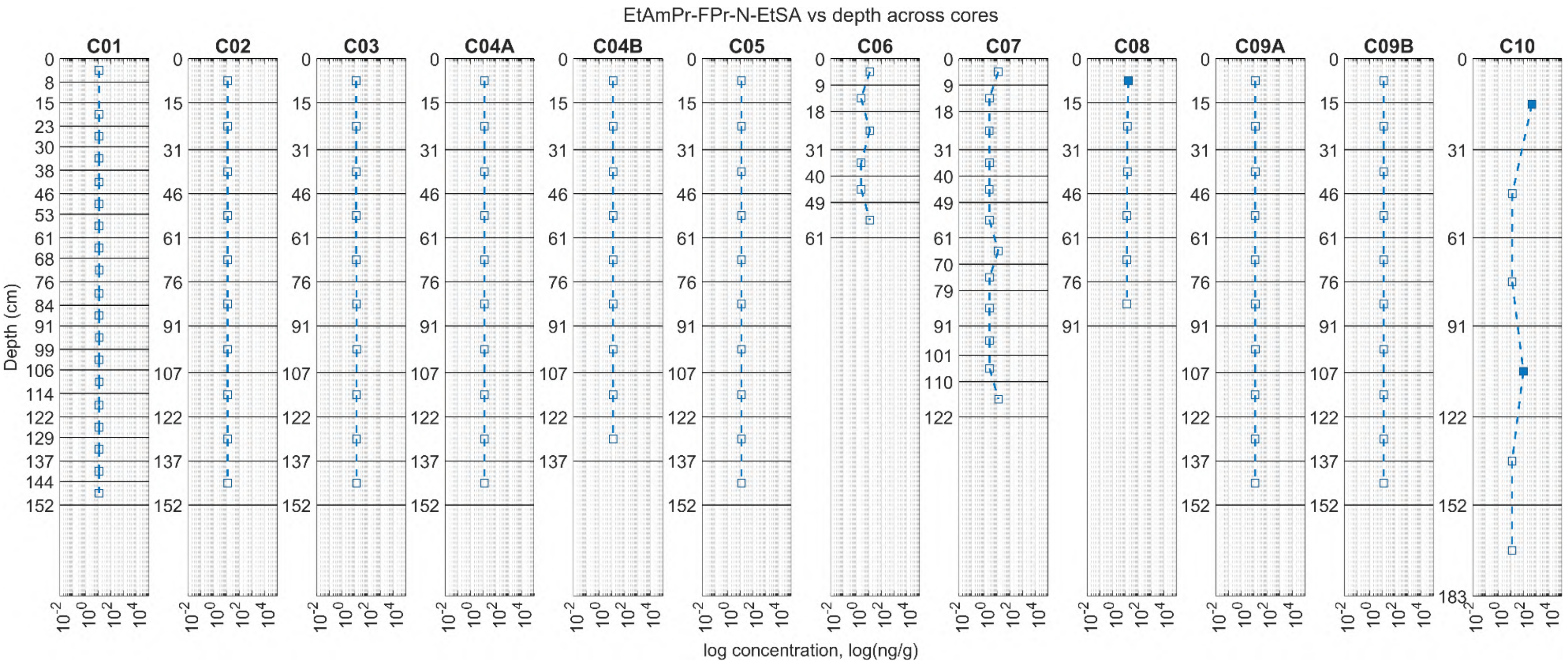

Figure S139. Vertical distribution profile of **EtAmPr-FB-N-EtSA** across the twelve studied cores

Note that the concentration is shown on a log-base-10 scale. For any given plot and compound, open markers with dashed-line connectors represent sampled depth intervals where the compounds' concentration was below the reporting limit – the location of the open marker along the x-axis is representative of those reporting limits.

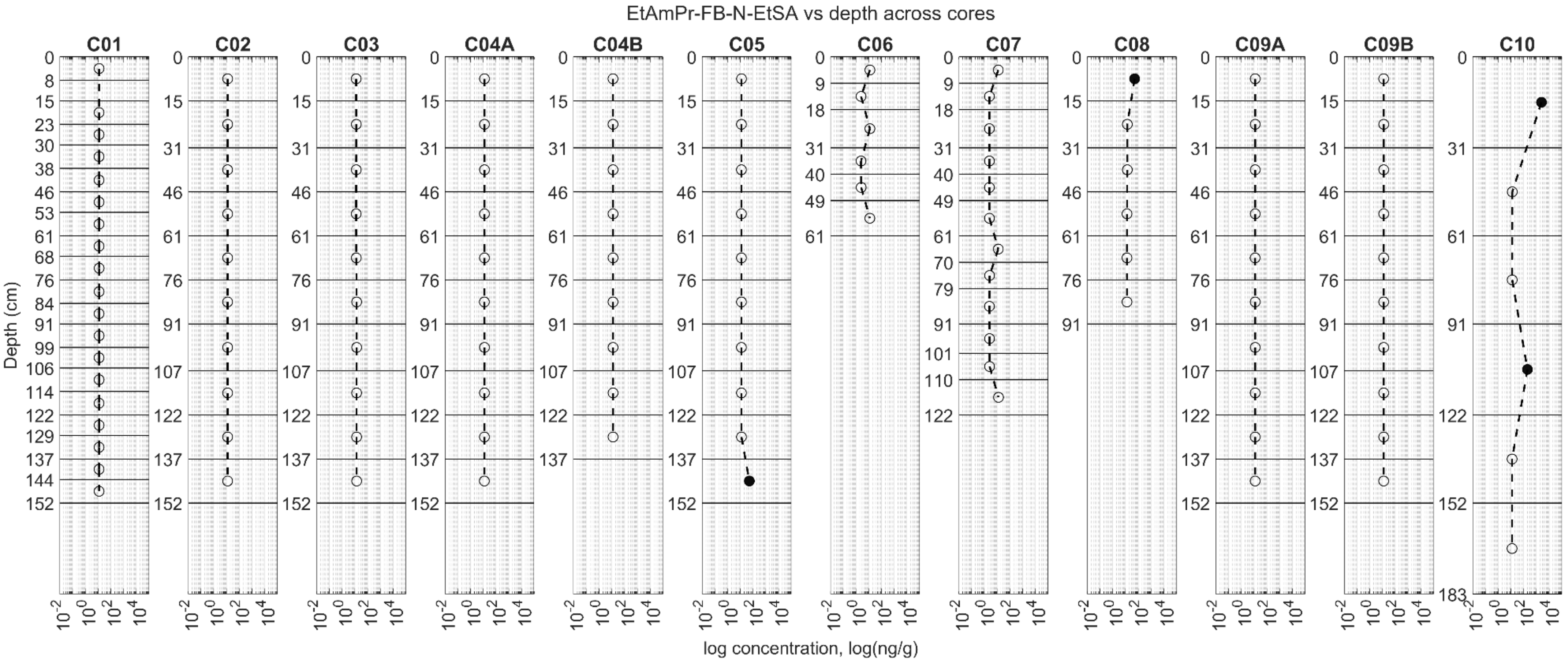

Figure S140. Vertical distribution profile of **EtAmPr-FPe-N-EtSA** across the twelve studied cores

Note that the concentration is shown on a log-base-10 scale. For any given plot and compound, open markers with dashed-line connectors represent sampled depth intervals where the compounds' concentration was below the reporting limit – the location of the open marker along the x-axis is representative of those reporting limits.

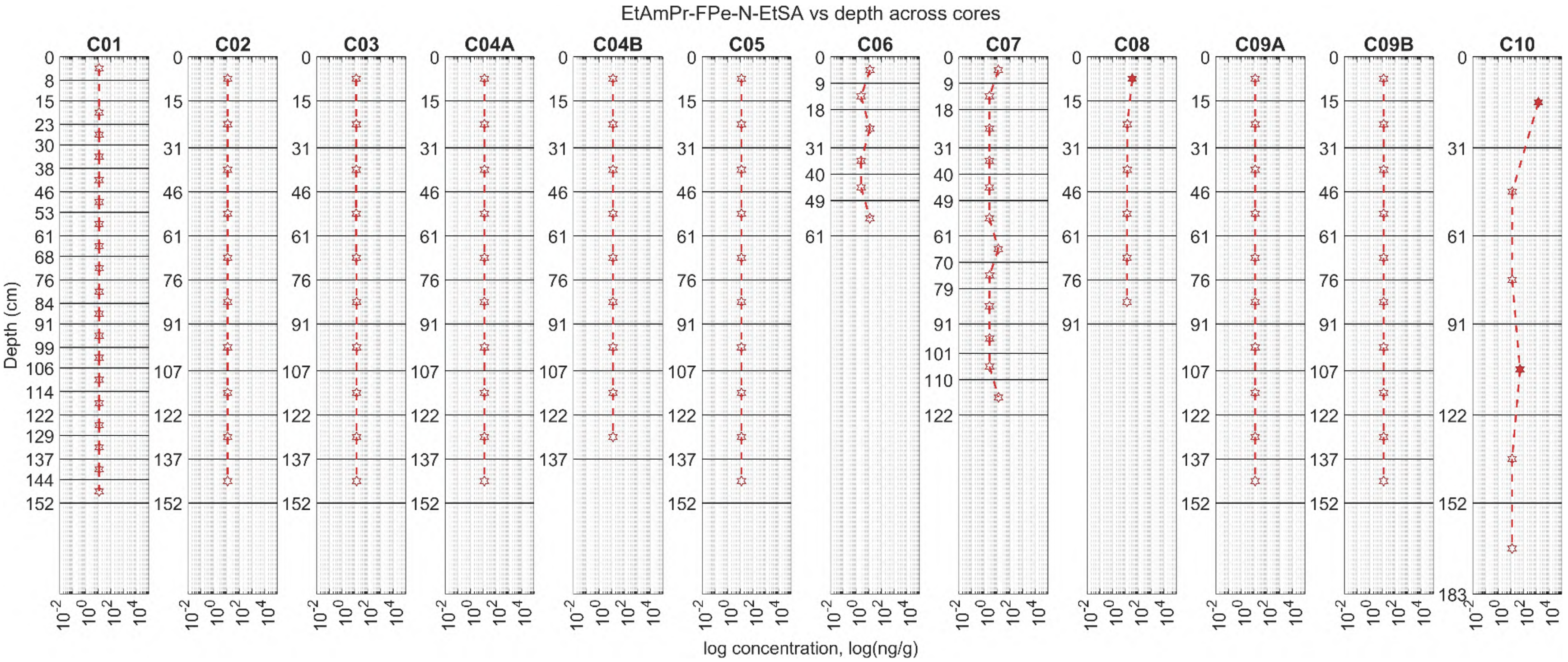

Figure S141. Vertical distribution profile of **EtAmPr-FHx-N-EtSA** across the twelve studied cores

Note that the concentration is shown on a log-base-10 scale. For any given plot and compound, open markers with dashed-line connectors represent sampled depth intervals where the compounds' concentration was below the reporting limit – the location of the open marker along the x-axis is representative of those reporting limits.

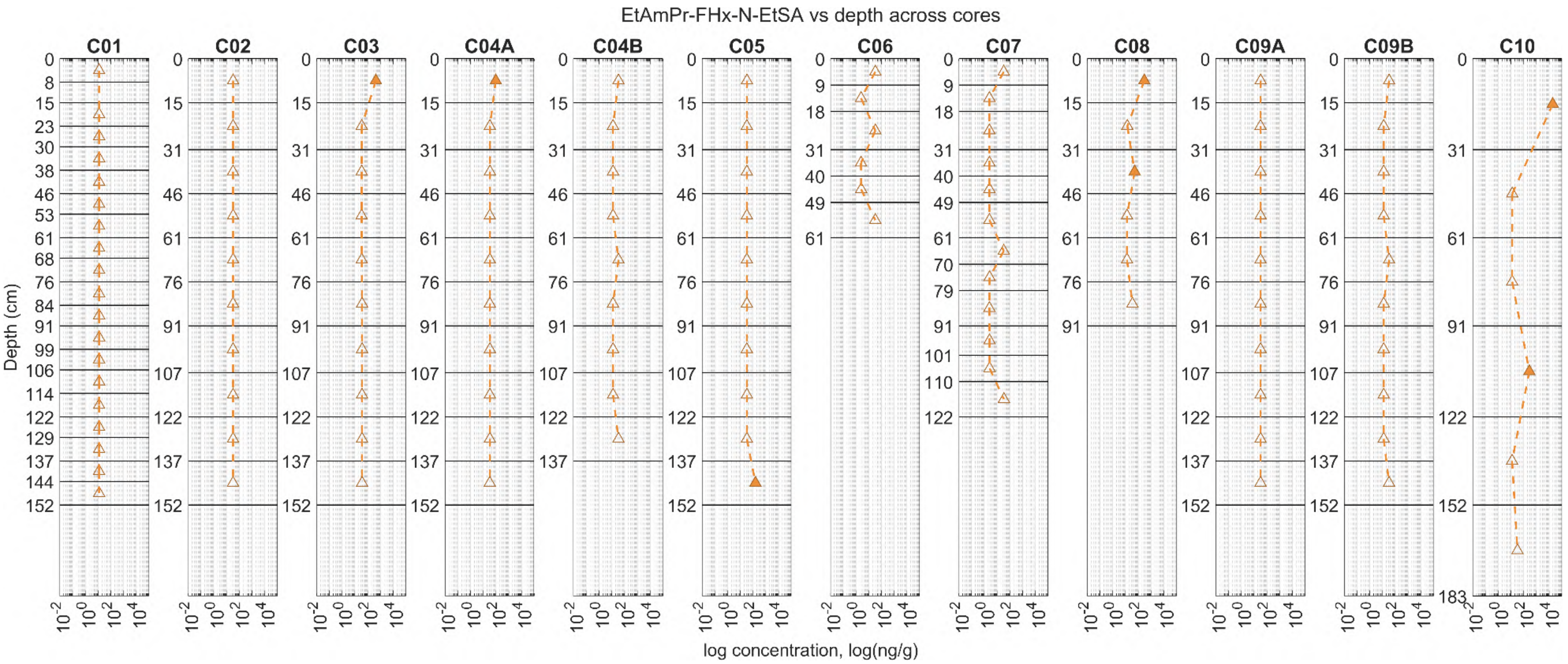

Figure S142. Vertical distribution profile of **EtAmPr-FHp-N-EtSA** across the twelve studied cores

Note that the concentration is shown on a log-base-10 scale. For any given plot and compound, open markers with dashed-line connectors represent sampled depth intervals where the compounds' concentration was below the reporting limit – the location of the open marker along the x-axis is representative of those reporting limits.

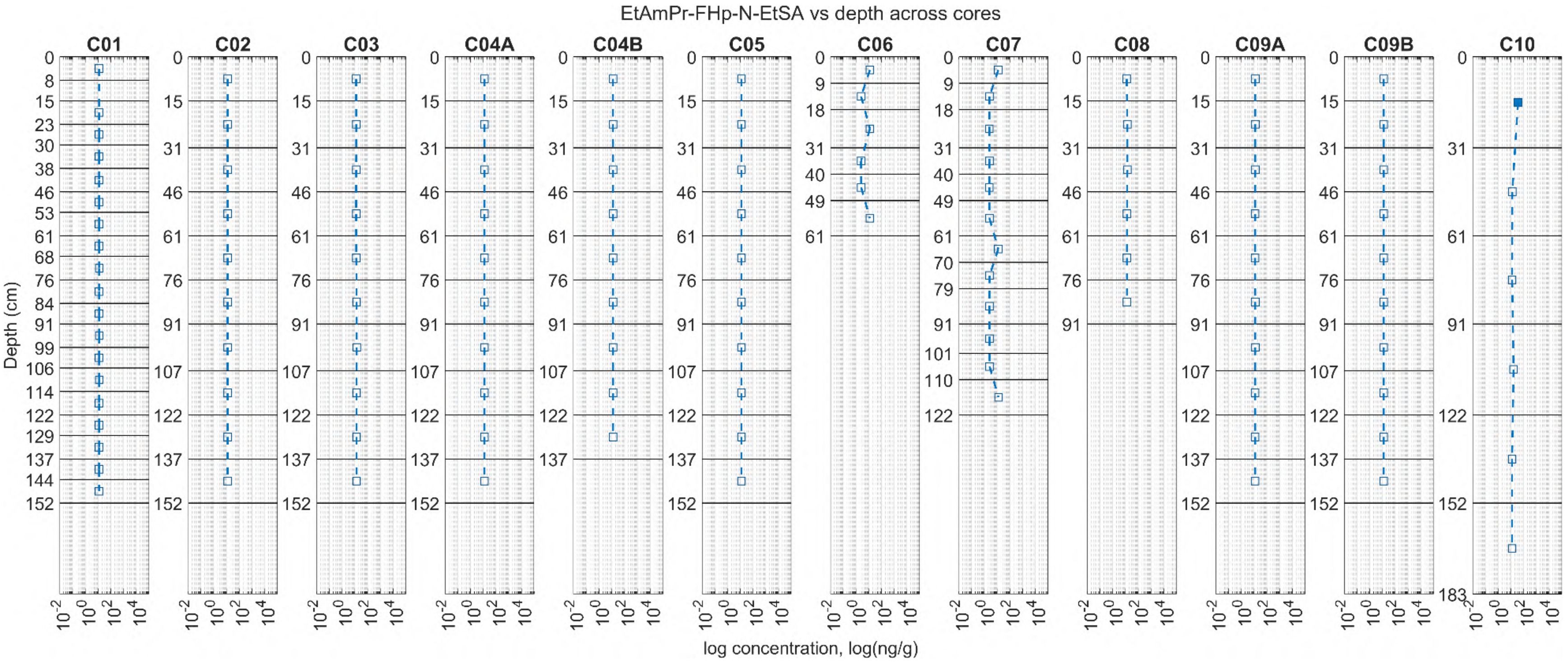

Figure S143. Vertical distribution profile of **EtAmPr-FO-N-EtSA** across the twelve studied cores

Note that the concentration is shown on a log-base-10 scale. For any given plot and compound, open markers with dashed-line connectors represent sampled depth intervals where the compounds' concentration was below the reporting limit – the location of the open marker along the x-axis is representative of those reporting limits.

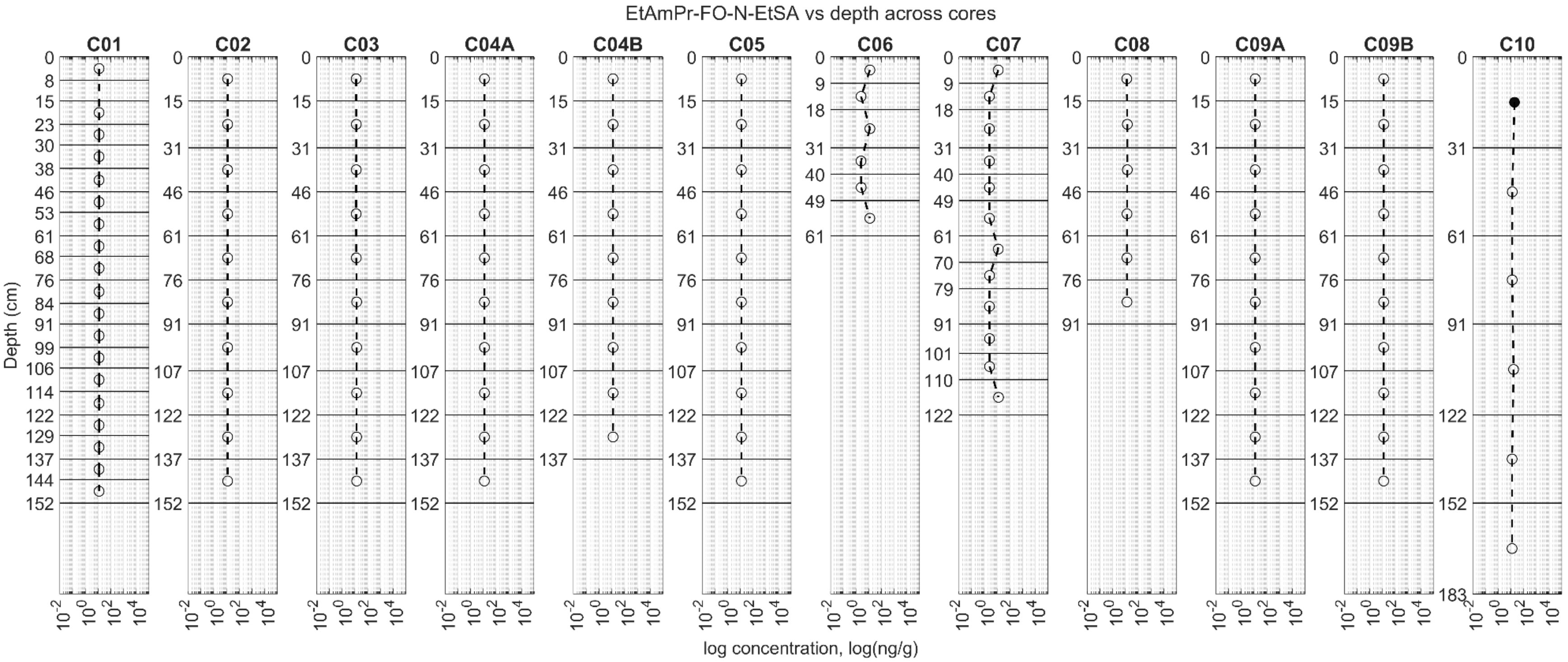

Figure S144. Vertical distribution profile of **7:3 FTCA** across the twelve studied cores

Note that the concentration is shown on a log-base-10 scale. For any given plot and compound, open markers with dashed-line connectors represent sampled depth intervals where the compounds' concentration was below the reporting limit – the location of the open marker along the x-axis is representative of those reporting limits.

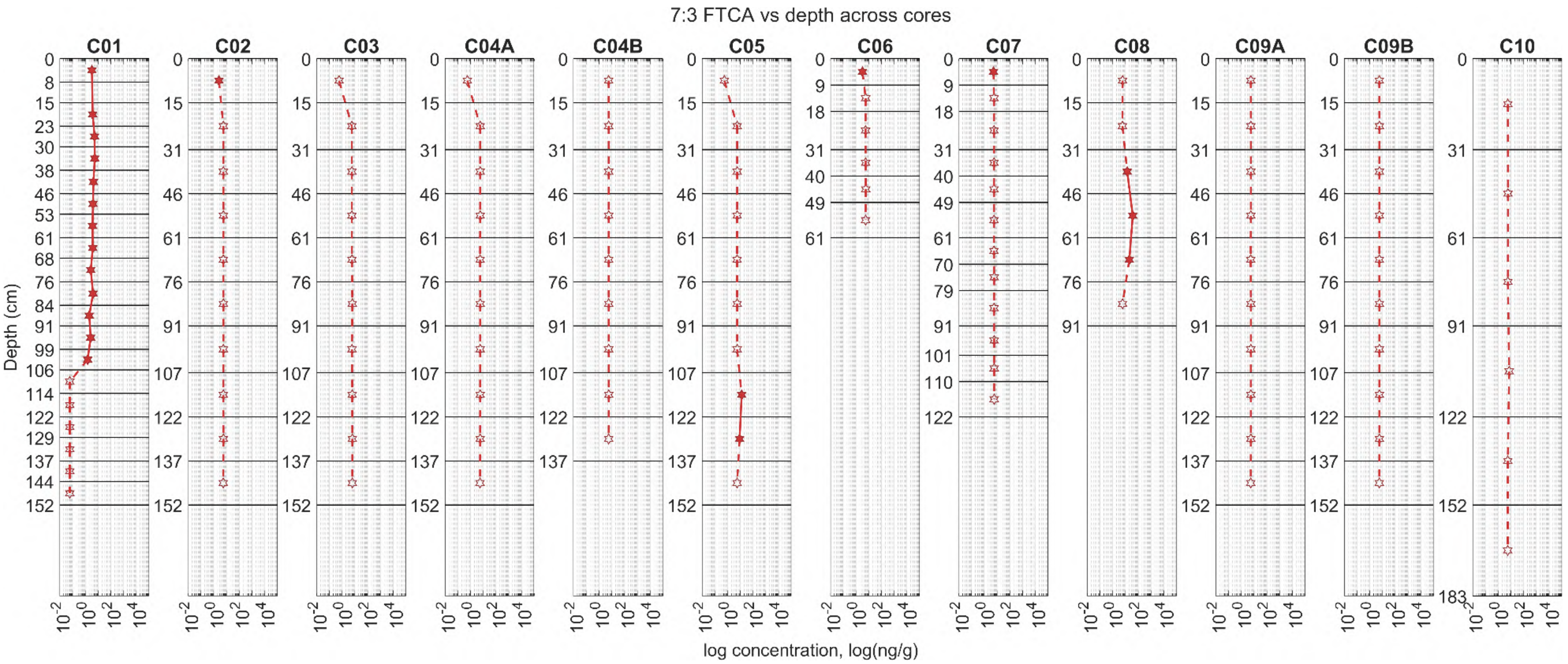

Figure S145. Vertical distribution profile of **PFBA** across the twelve studied cores

Note that the concentration is shown on a log-base-10 scale. For any given plot and compound, open markers with dashed-line connectors represent sampled depth intervals where the compounds' concentration was below the reporting limit – the location of the open marker along the x-axis is representative of those reporting limits.

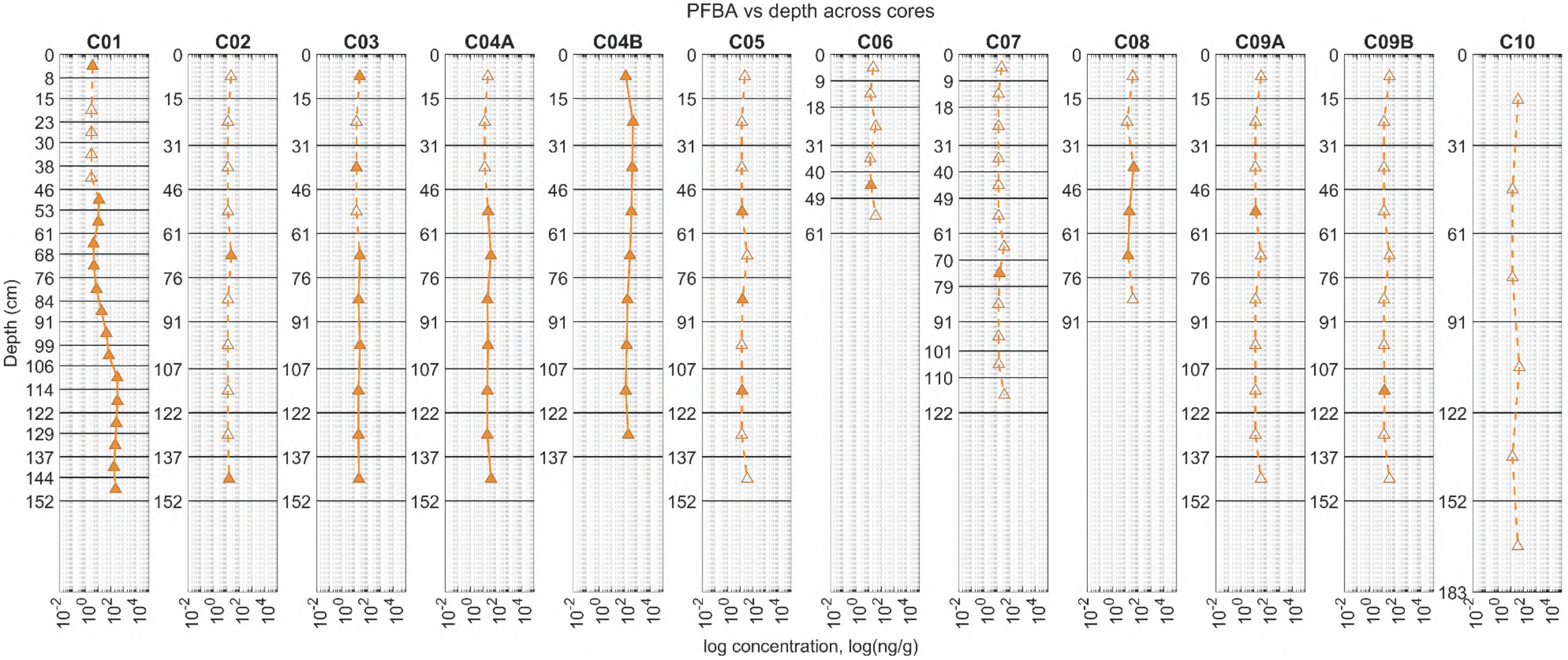

Figure S146. Vertical distribution profile of **PFPeA** across the twelve studied cores

Note that the concentration is shown on a log-base-10 scale. For any given plot and compound, open markers with dashed-line connectors represent sampled depth intervals where the compounds' concentration was below the reporting limit – the location of the open marker along the x-axis is representative of those reporting limits.

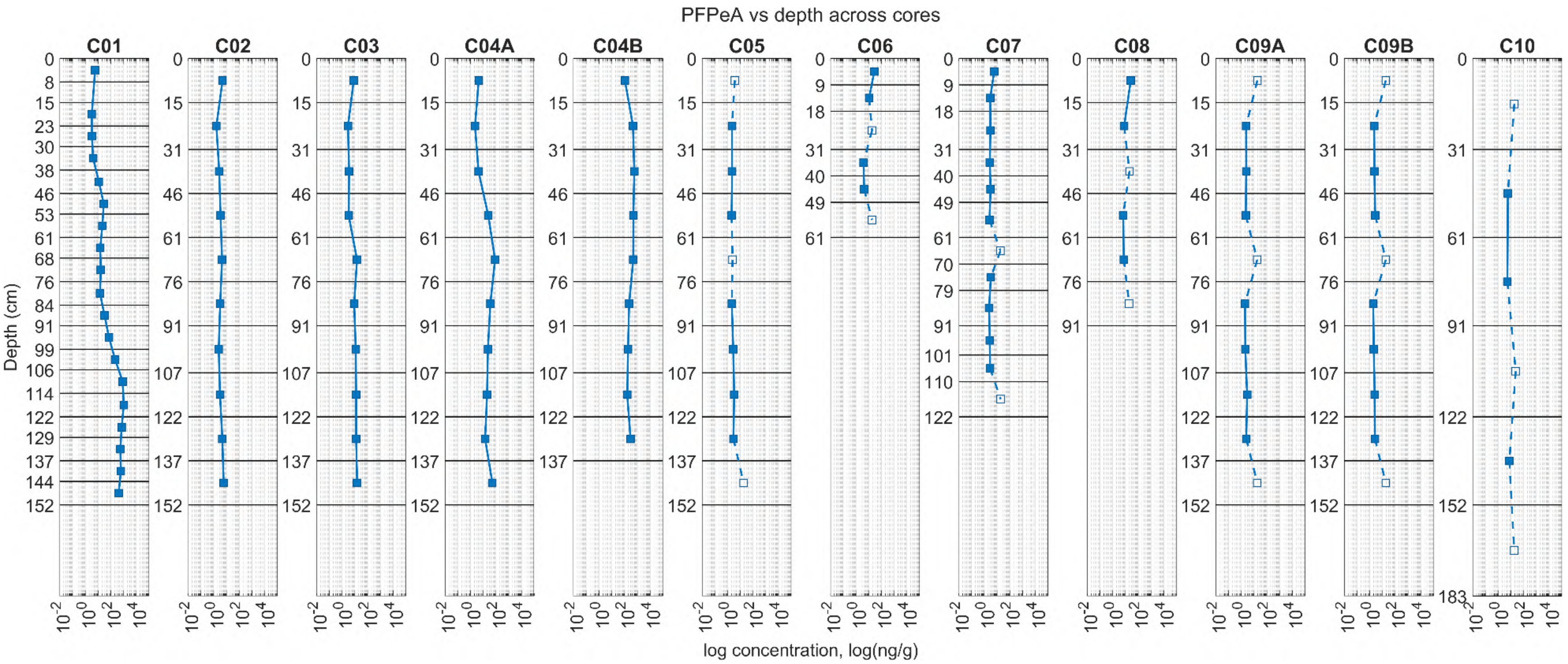

Figure S147. Vertical distribution profile of **PFHxA** across the twelve studied cores

Note that the concentration is shown on a log-base-10 scale. For any given plot and compound, open markers with dashed-line connectors represent sampled depth intervals where the compounds' concentration was below the reporting limit – the location of the open marker along the x-axis is representative of those reporting limits.

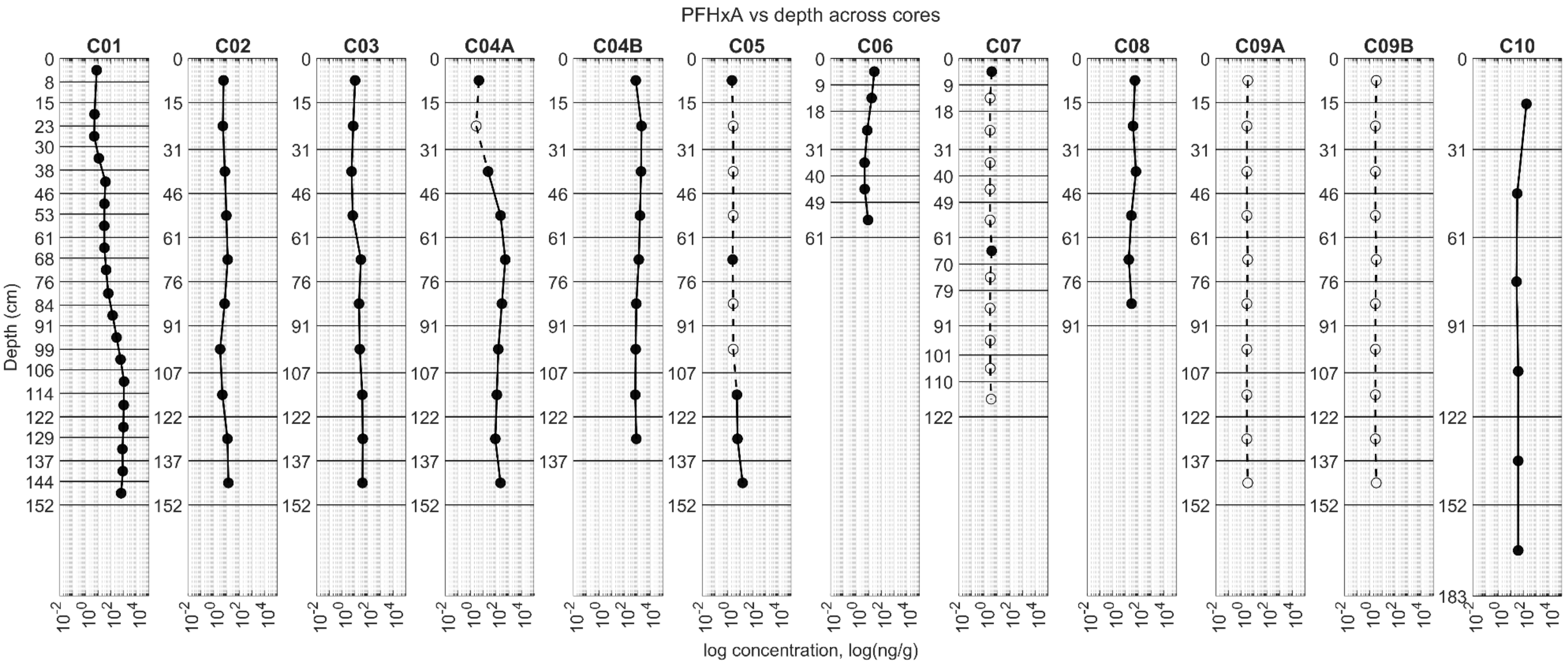

Figure S148. Vertical distribution profile of **PFHpA** across the twelve studied cores

Note that the concentration is shown on a log-base-10 scale. For any given plot and compound, open markers with dashed-line connectors represent sampled depth intervals where the compounds' concentration was below the reporting limit – the location of the open marker along the x-axis is representative of those reporting limits.

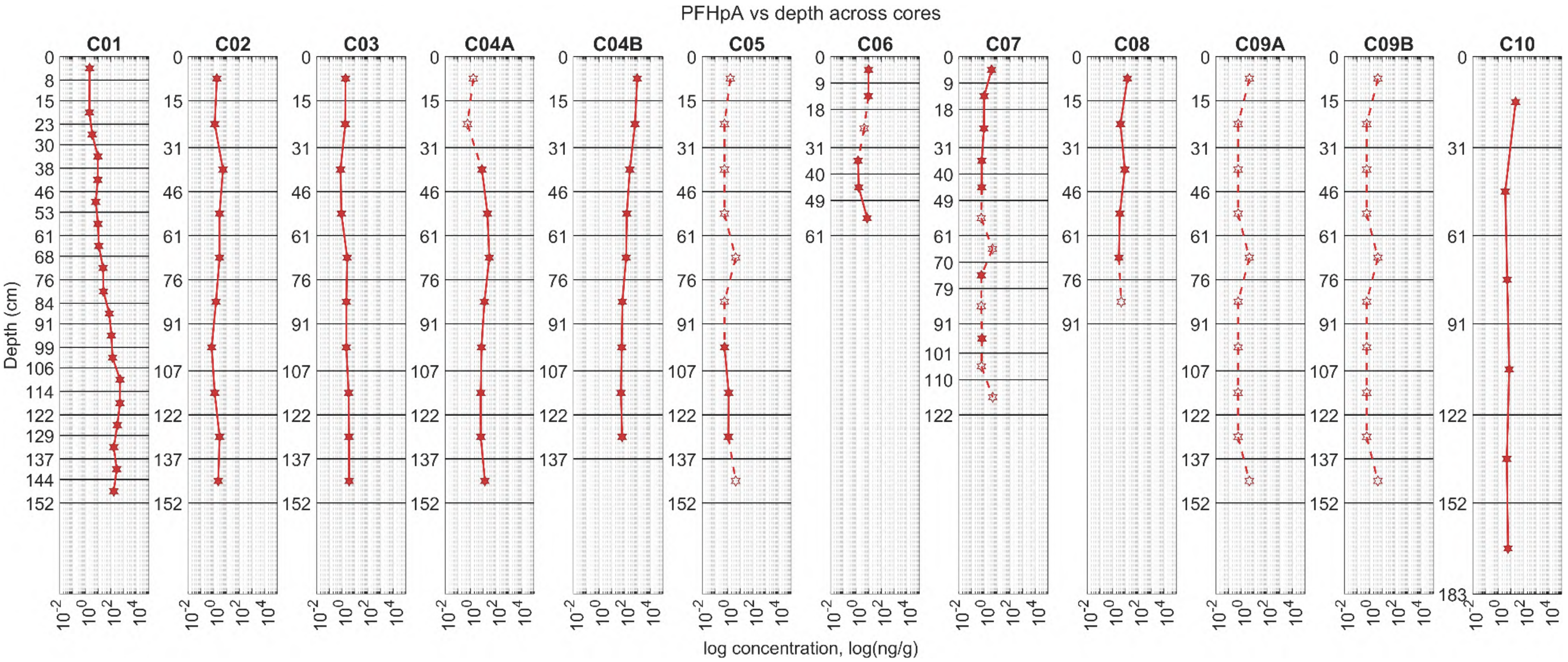

Figure S149. Vertical distribution profile of **PFOA** across the twelve studied cores

Note that the concentration is shown on a log-base-10 scale. For any given plot and compound, open markers with dashed-line connectors represent sampled depth intervals where the compounds' concentration was below the reporting limit – the location of the open marker along the x-axis is representative of those reporting limits.

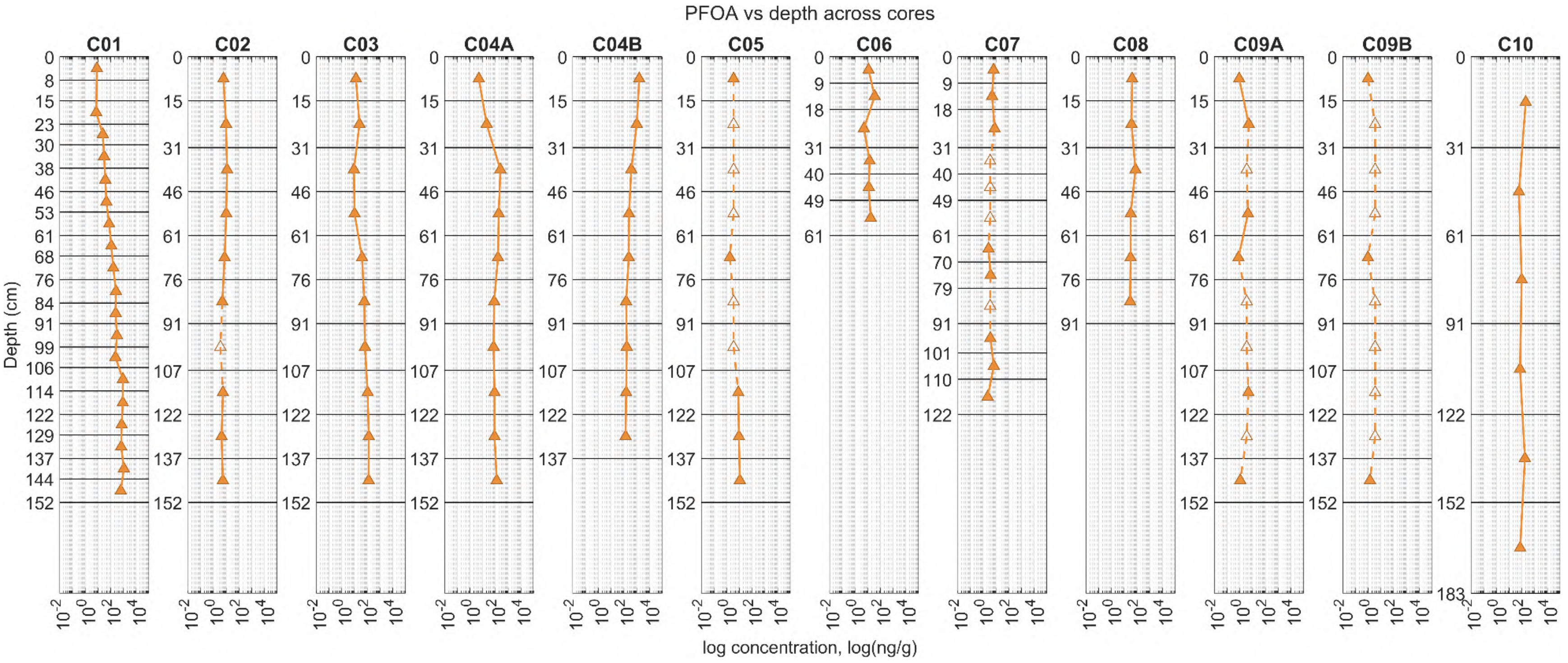

Figure S150. Vertical distribution profile of **PFNA** across the twelve studied cores

Note that the concentration is shown on a log-base-10 scale. For any given plot and compound, open markers with dashed-line connectors represent sampled depth intervals where the compounds' concentration was below the reporting limit – the location of the open marker along the x-axis is representative of those reporting limits.

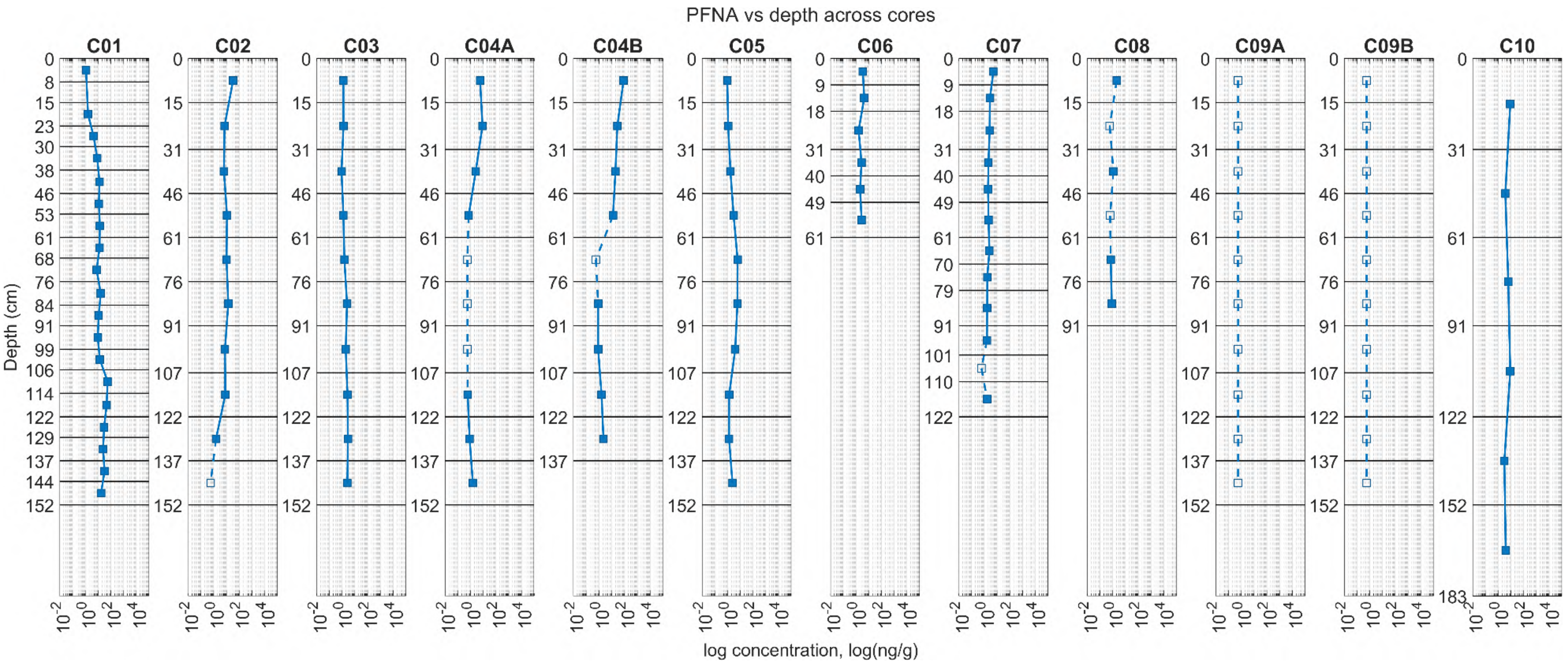

Figure S151. Vertical distribution profile of **PFDA** across the twelve studied cores

Note that the concentration is shown on a log-base-10 scale. For any given plot and compound, open markers with dashed-line connectors represent sampled depth intervals where the compounds' concentration was below the reporting limit – the location of the open marker along the x-axis is representative of those reporting limits.

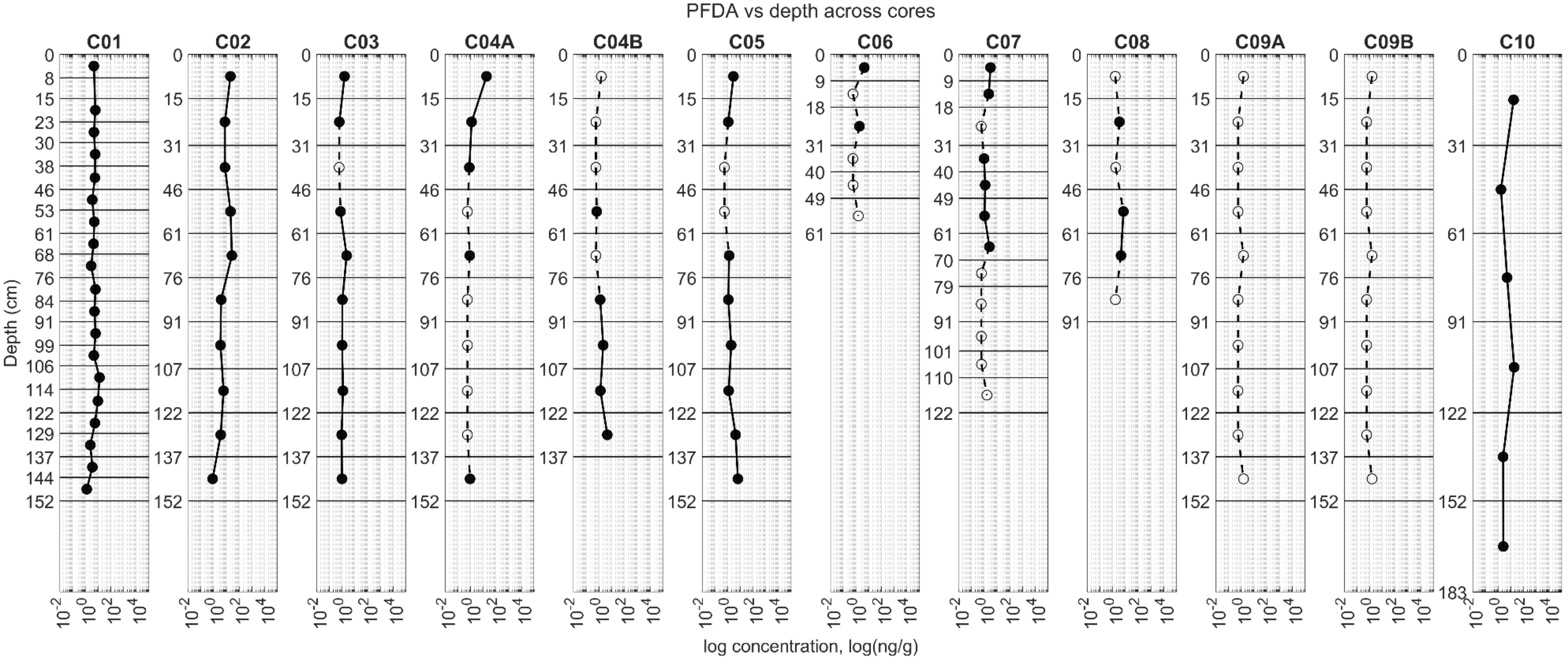

Figure S152. Vertical distribution profile of **PFUdA** across the twelve studied cores

Note that the concentration is shown on a log-base-10 scale. For any given plot and compound, open markers with dashed-line connectors represent sampled depth intervals where the compounds' concentration was below the reporting limit – the location of the open marker along the x-axis is representative of those reporting limits.

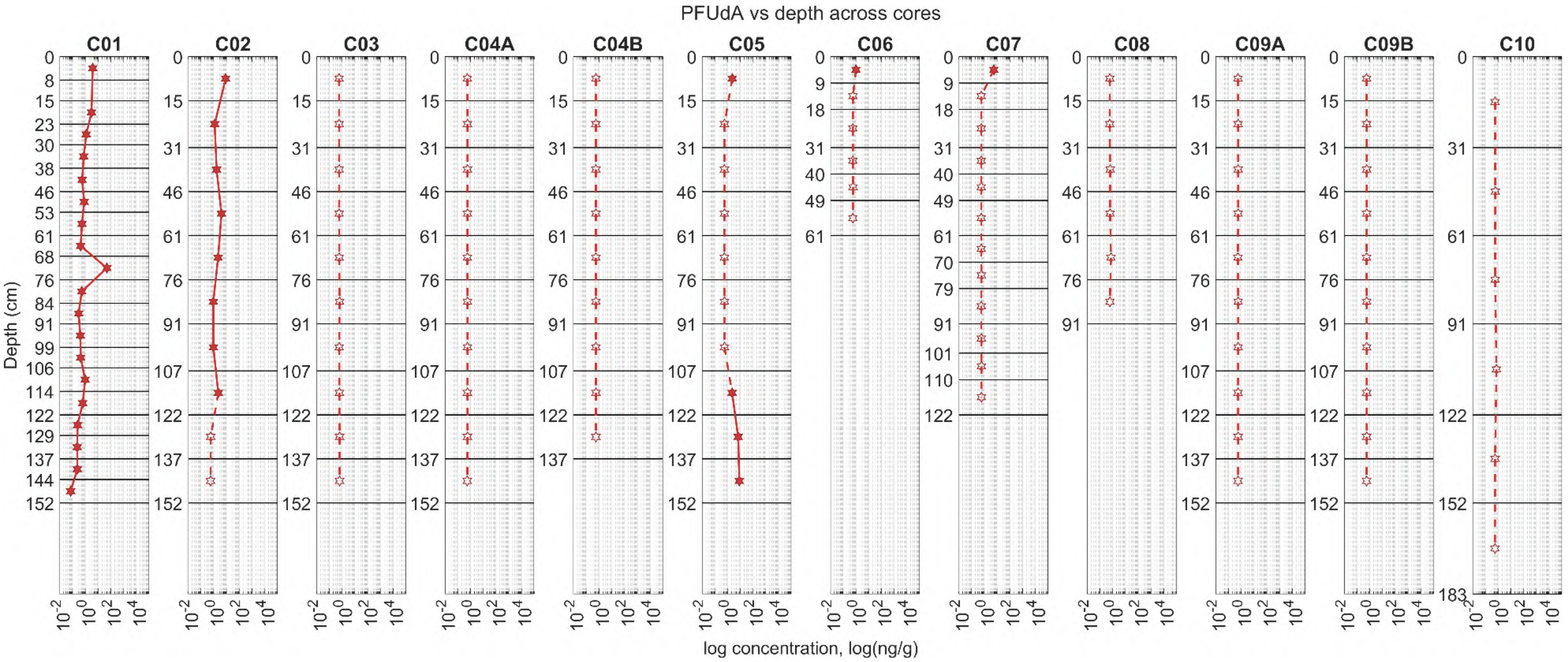

Figure S153. Vertical distribution profile of **PFDoA** across the twelve studied cores

Note that the concentration is shown on a log-base-10 scale. For any given plot and compound, open markers with dashed-line connectors represent sampled depth intervals where the compounds' concentration was below the reporting limit – the location of the open marker along the x-axis is representative of those reporting limits.

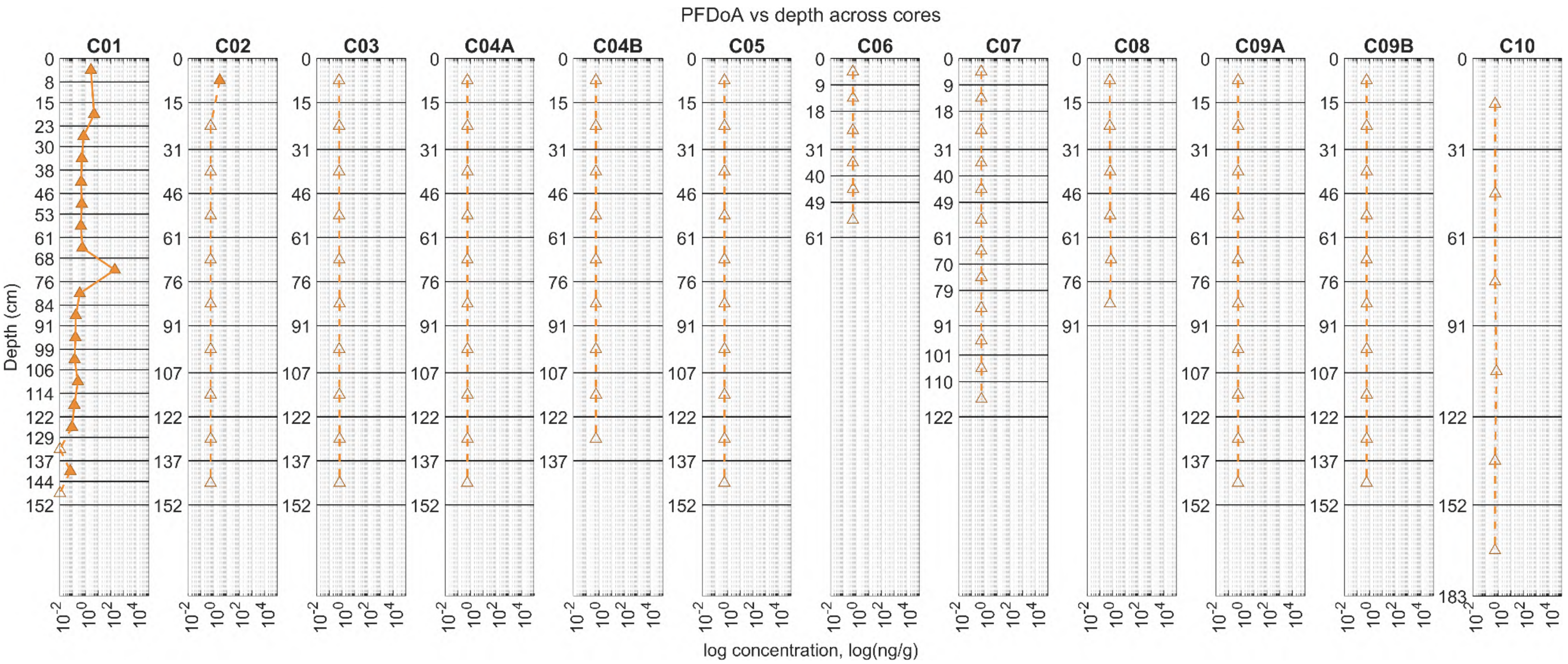

Figure S154. Vertical distribution profile of **6:2 UFTCA** across the twelve studied cores

Note that the concentration is shown on a log-base-10 scale. For any given plot and compound, open markers with dashed-line connectors represent sampled depth intervals where the compounds' concentration was below the reporting limit – the location of the open marker along the x-axis is representative of those reporting limits.

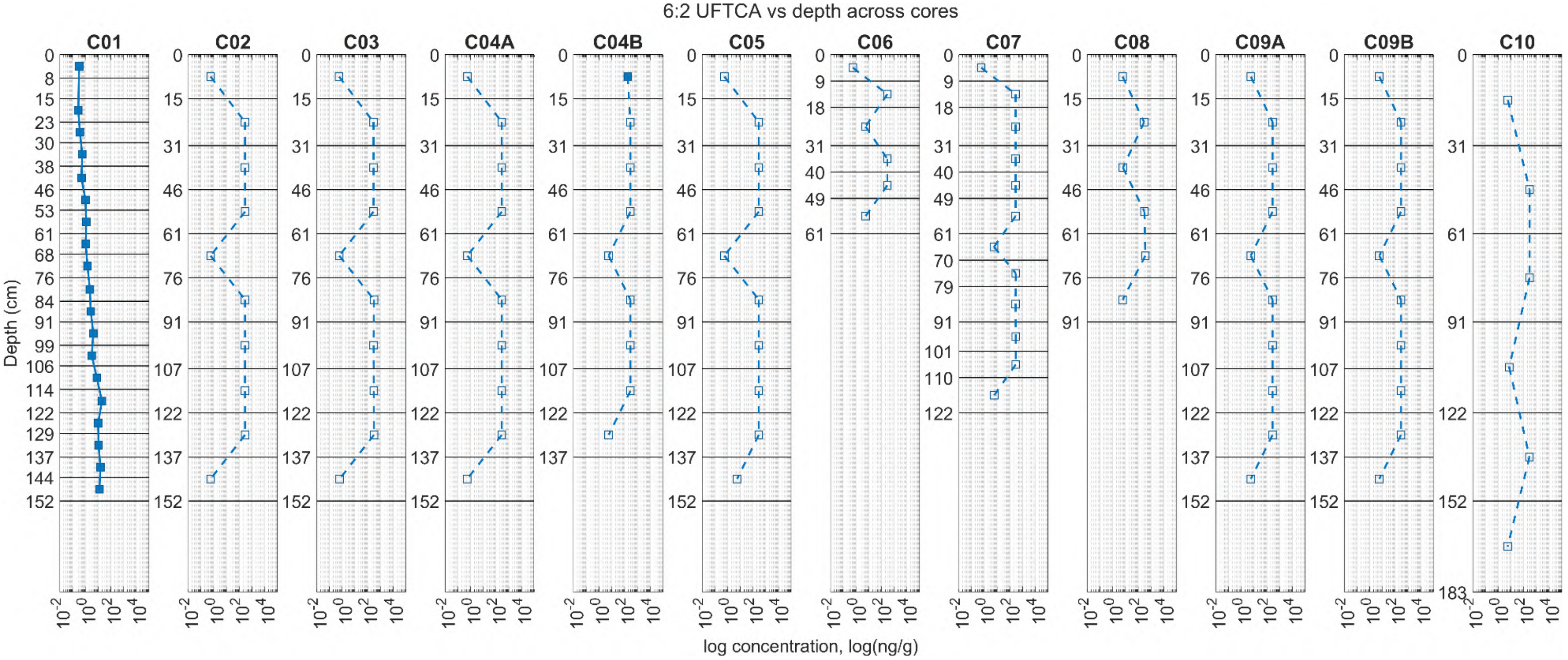

Figure S155. Vertical distribution profile of **8:2 UFTCA** across the twelve studied cores

Note that the concentration is shown on a log-base-10 scale. For any given plot and compound, open markers with dashed-line connectors represent sampled depth intervals where the compounds' concentration was below the reporting limit – the location of the open marker along the x-axis is representative of those reporting limits.

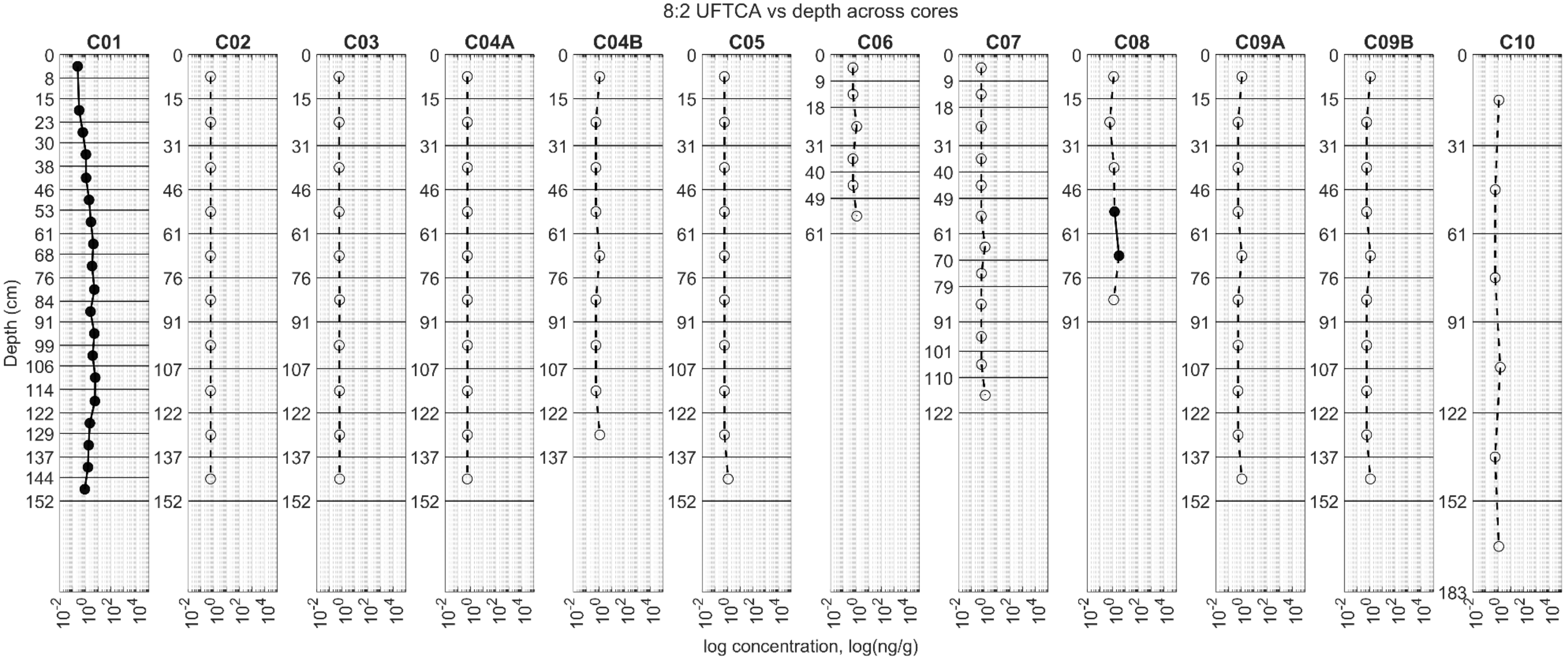

Figure S156. Vertical distribution profile of **PFPrS** across the twelve studied cores

Note that the concentration is shown on a log-base-10 scale. For any given plot and compound, open markers with dashed-line connectors represent sampled depth intervals where the compounds' concentration was below the reporting limit – the location of the open marker along the x-axis is representative of those reporting limits.

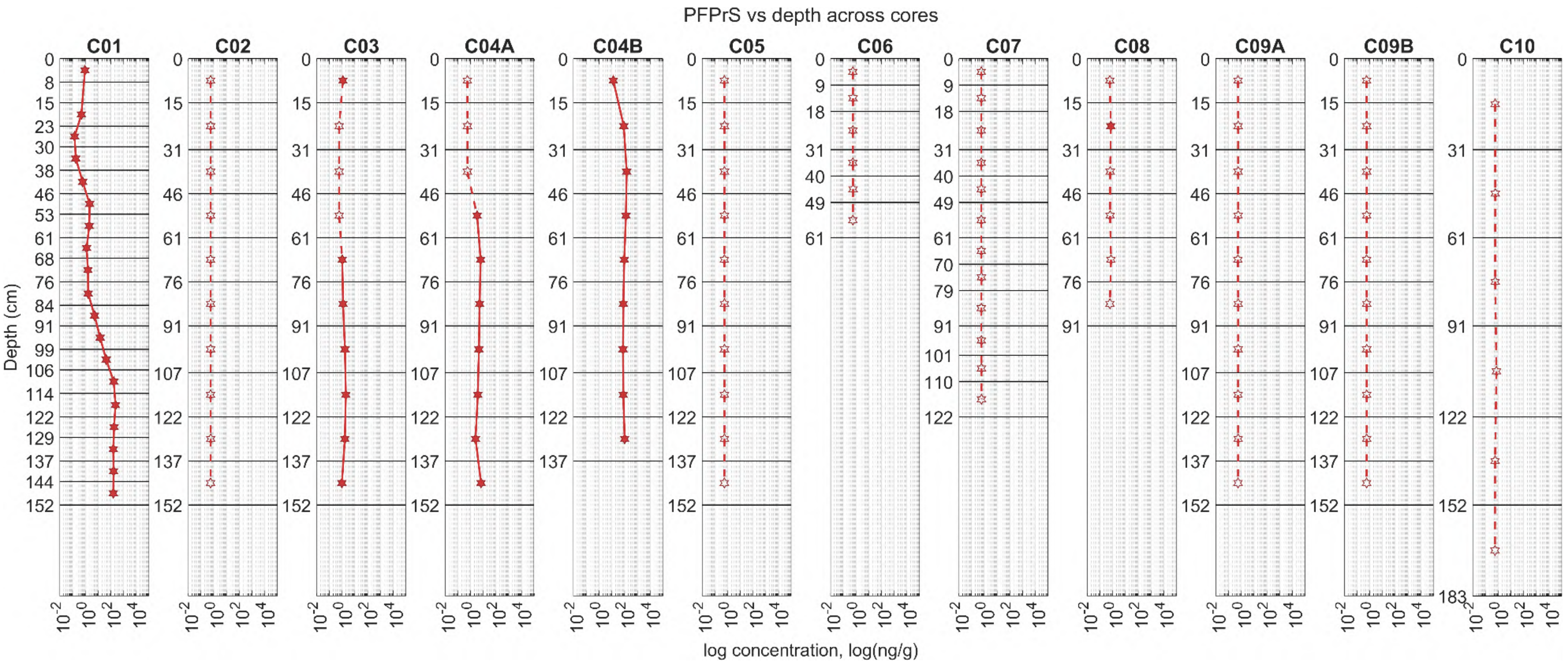

Figure S157. Vertical distribution profile of **PFBS** across the twelve studied cores

Note that the concentration is shown on a log-base-10 scale. For any given plot and compound, open markers with dashed-line connectors represent sampled depth intervals where the compounds' concentration was below the reporting limit – the location of the open marker along the x-axis is representative of those reporting limits.

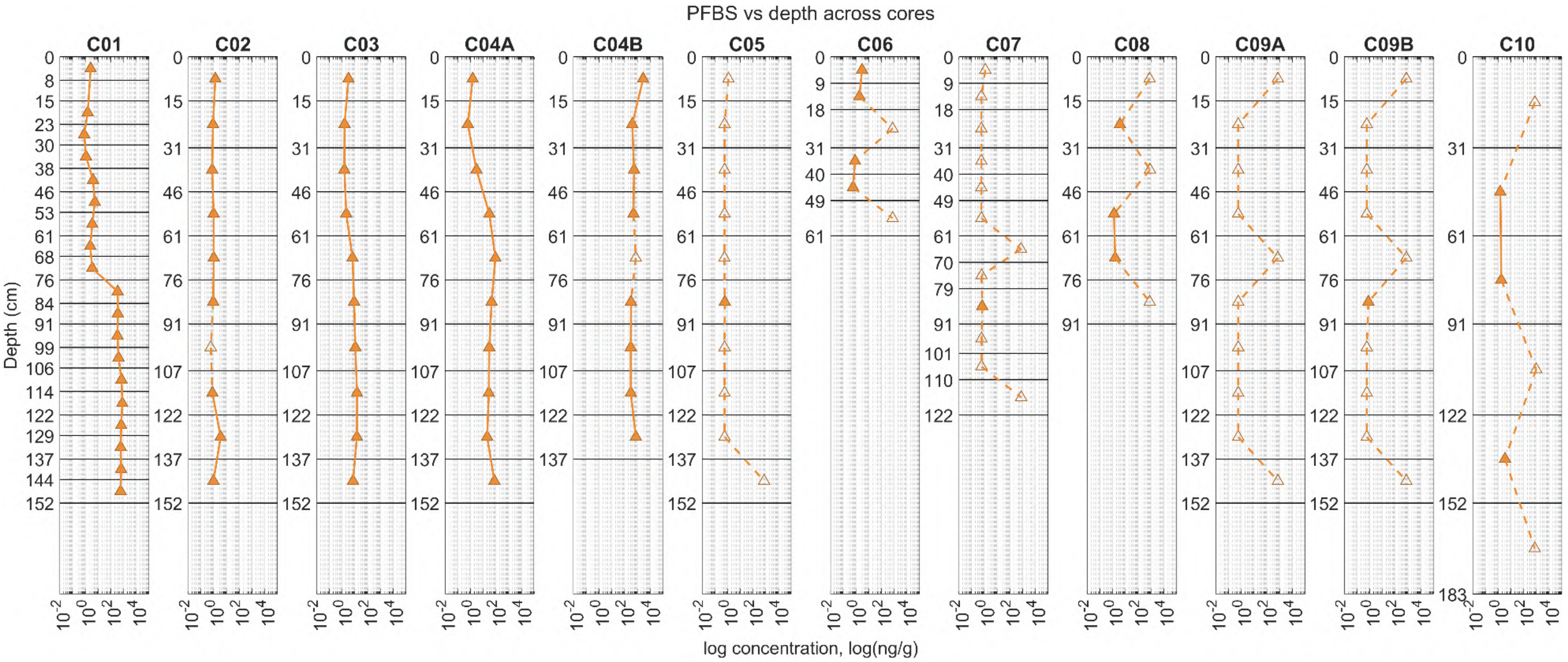

Figure S158. Vertical distribution profile of **PFPeS** across the twelve studied cores

Note that the concentration is shown on a log-base-10 scale. For any given plot and compound, open markers with dashed-line connectors represent sampled depth intervals where the compounds' concentration was below the reporting limit – the location of the open marker along the x-axis is representative of those reporting limits.

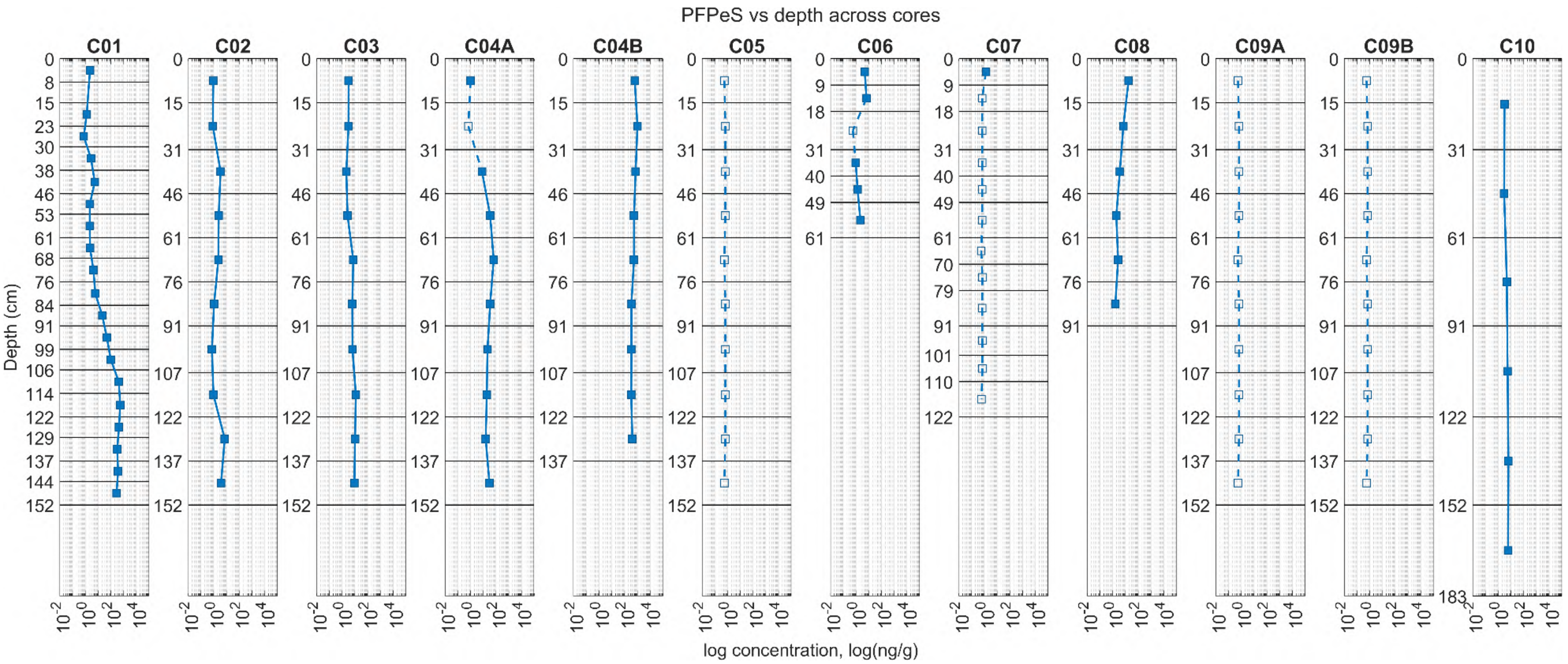

Figure S159. Vertical distribution profile of **PFHxS** across the twelve studied cores

Note that the concentration is shown on a log-base-10 scale. For any given plot and compound, open markers with dashed-line connectors represent sampled depth intervals where the compounds' concentration was below the reporting limit – the location of the open marker along the x-axis is representative of those reporting limits.

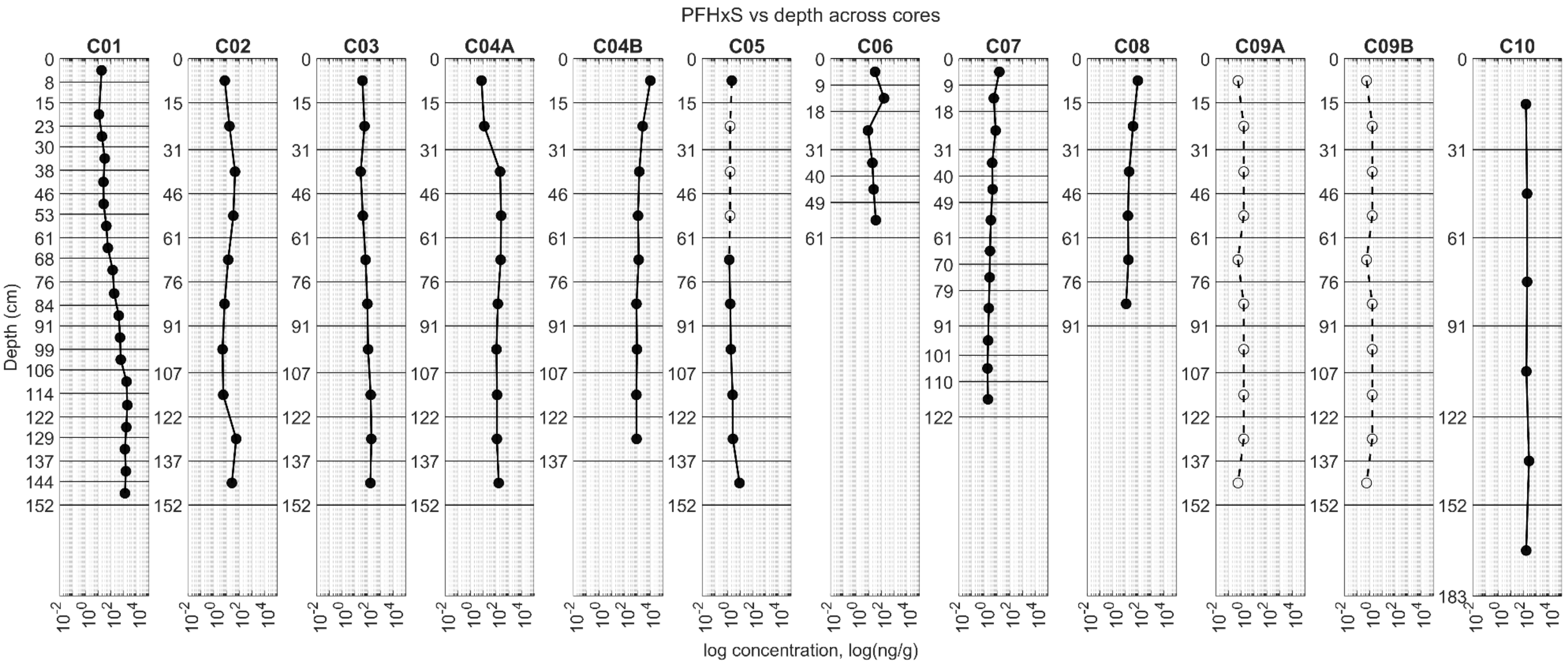

Figure S160. Vertical distribution profile of **PFHpS** across the twelve studied cores

Note that the concentration is shown on a log-base-10 scale. For any given plot and compound, open markers with dashed-line connectors represent sampled depth intervals where the compounds' concentration was below the reporting limit – the location of the open marker along the x-axis is representative of those reporting limits.

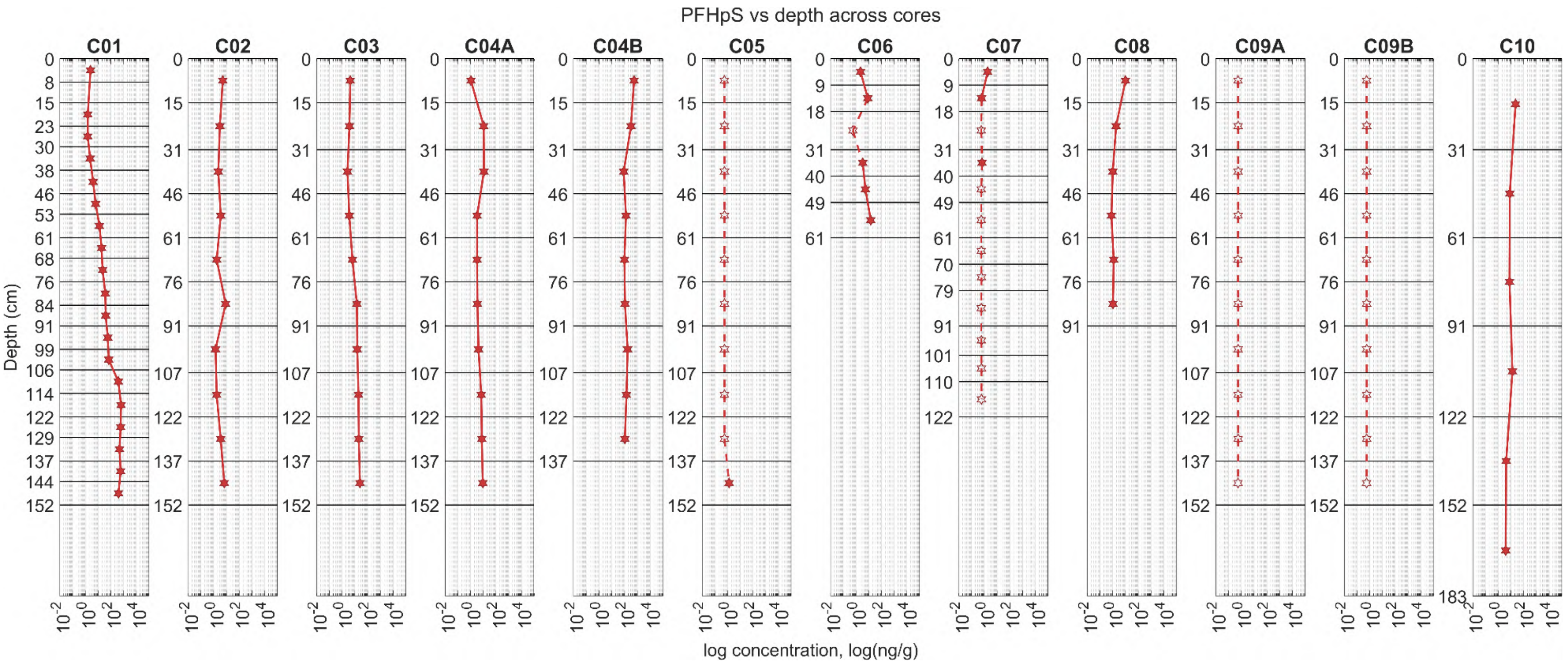

Figure S161. Vertical distribution profile of **PFOS** across the twelve studied cores

Note that the concentration is shown on a log-base-10 scale. For any given plot and compound, open markers with dashed-line connectors represent sampled depth intervals where the compounds' concentration was below the reporting limit – the location of the open marker along the x-axis is representative of those reporting limits.

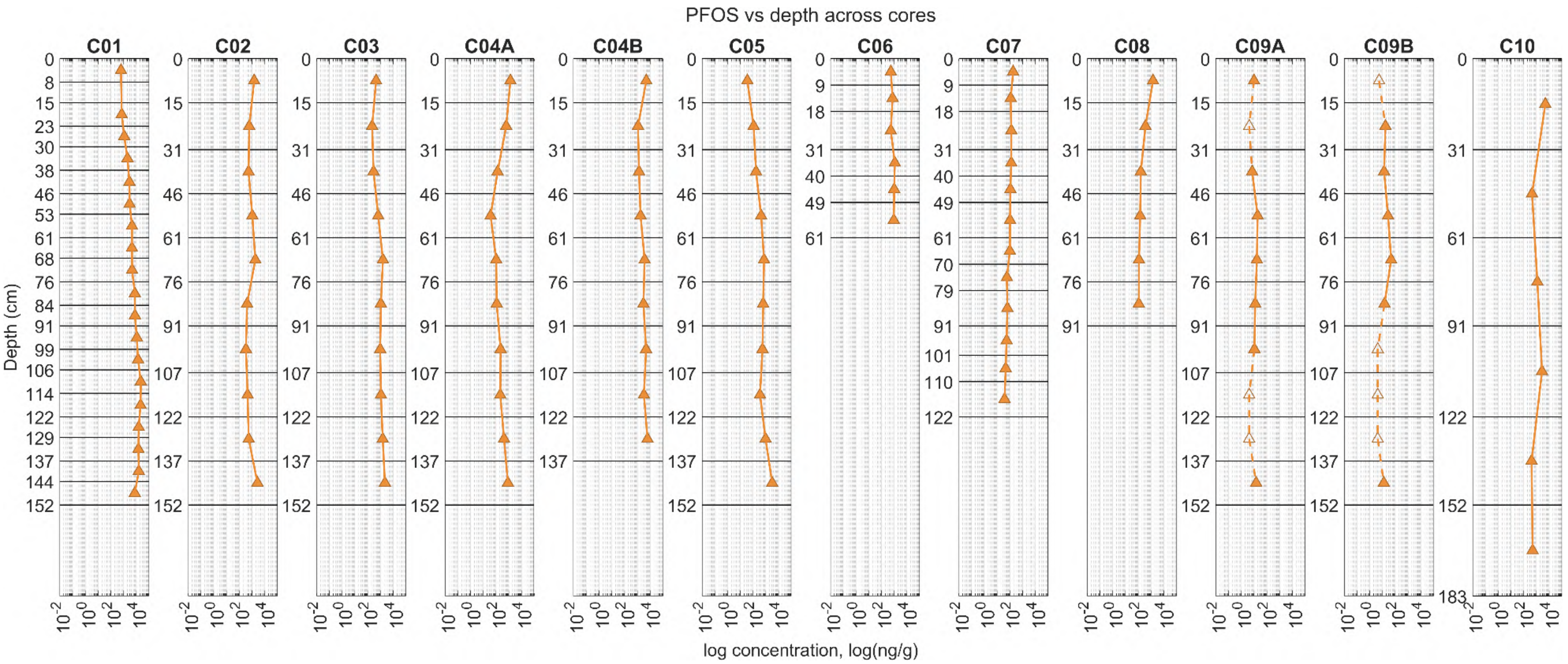

Figure S162. Vertical distribution profile of **PFNS** across the twelve studied cores

Note that the concentration is shown on a log-base-10 scale. For any given plot and compound, open markers with dashed-line connectors represent sampled depth intervals where the compounds' concentration was below the reporting limit – the location of the open marker along the x-axis is representative of those reporting limits.

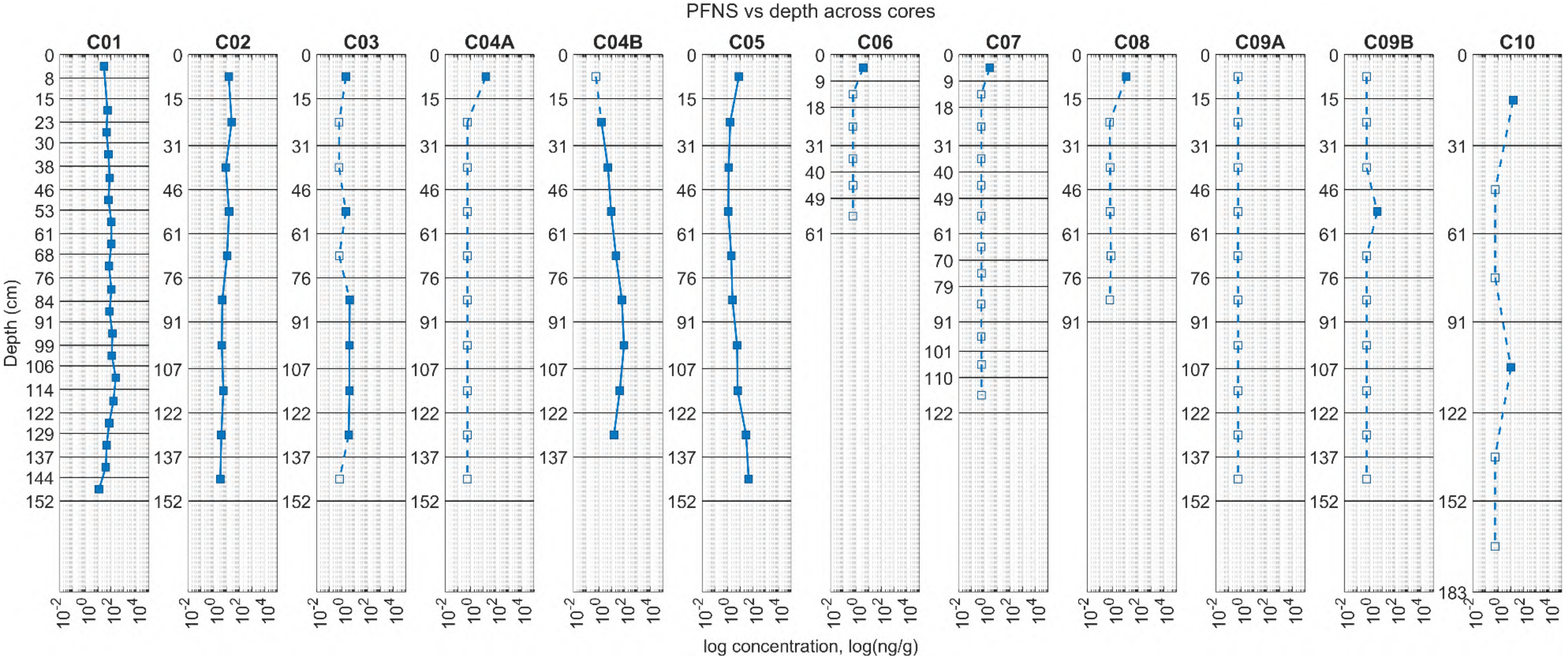

Figure S163. Vertical distribution profile of **PFDS** across the twelve studied cores

Note that the concentration is shown on a log-base-10 scale. For any given plot and compound, open markers with dashed-line connectors represent sampled depth intervals where the compounds' concentration was below the reporting limit – the location of the open marker along the x-axis is representative of those reporting limits.

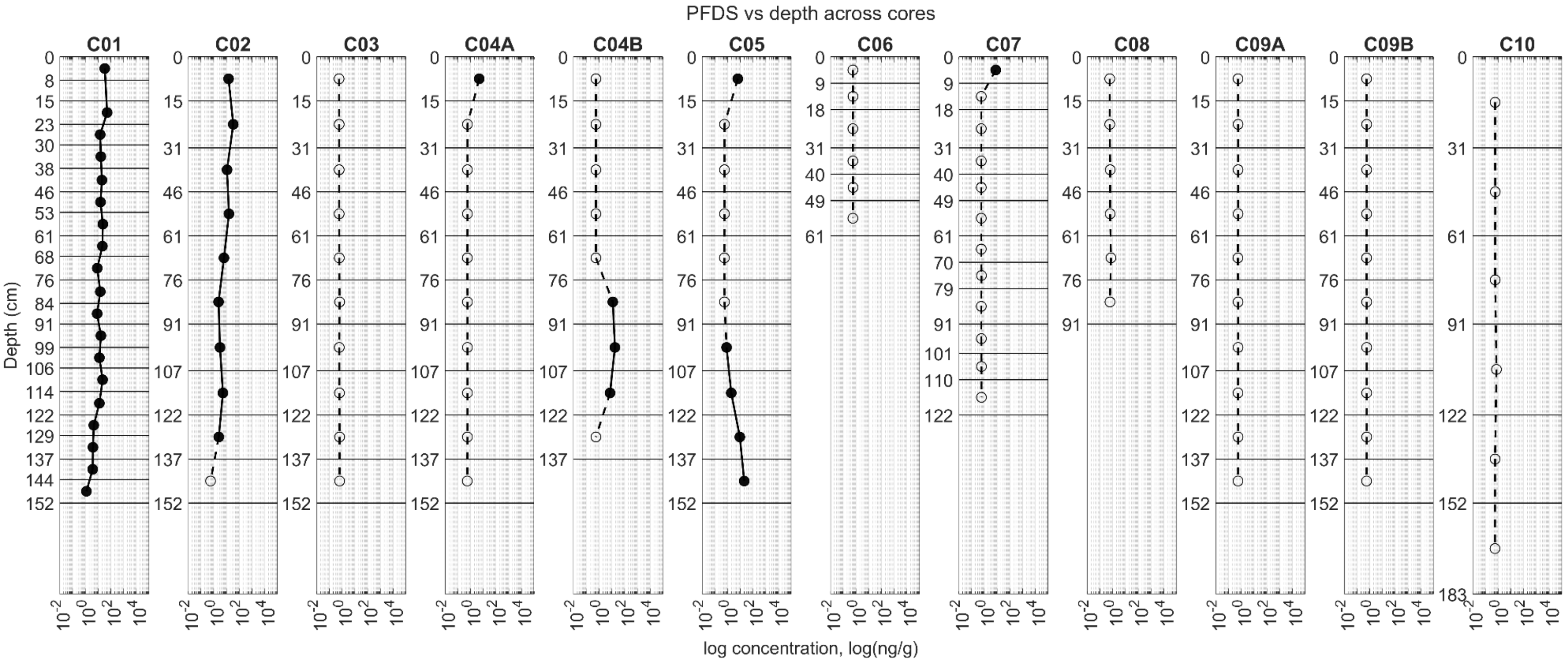

Figure S163. Vertical distribution profile of **PFDoS** across the twelve studied cores

Note that the concentration is shown on a log-base-10 scale. For any given plot and compound, open markers with dashed-line connectors represent sampled depth intervals where the compounds' concentration was below the reporting limit – the location of the open marker along the x-axis is representative of those reporting limits.

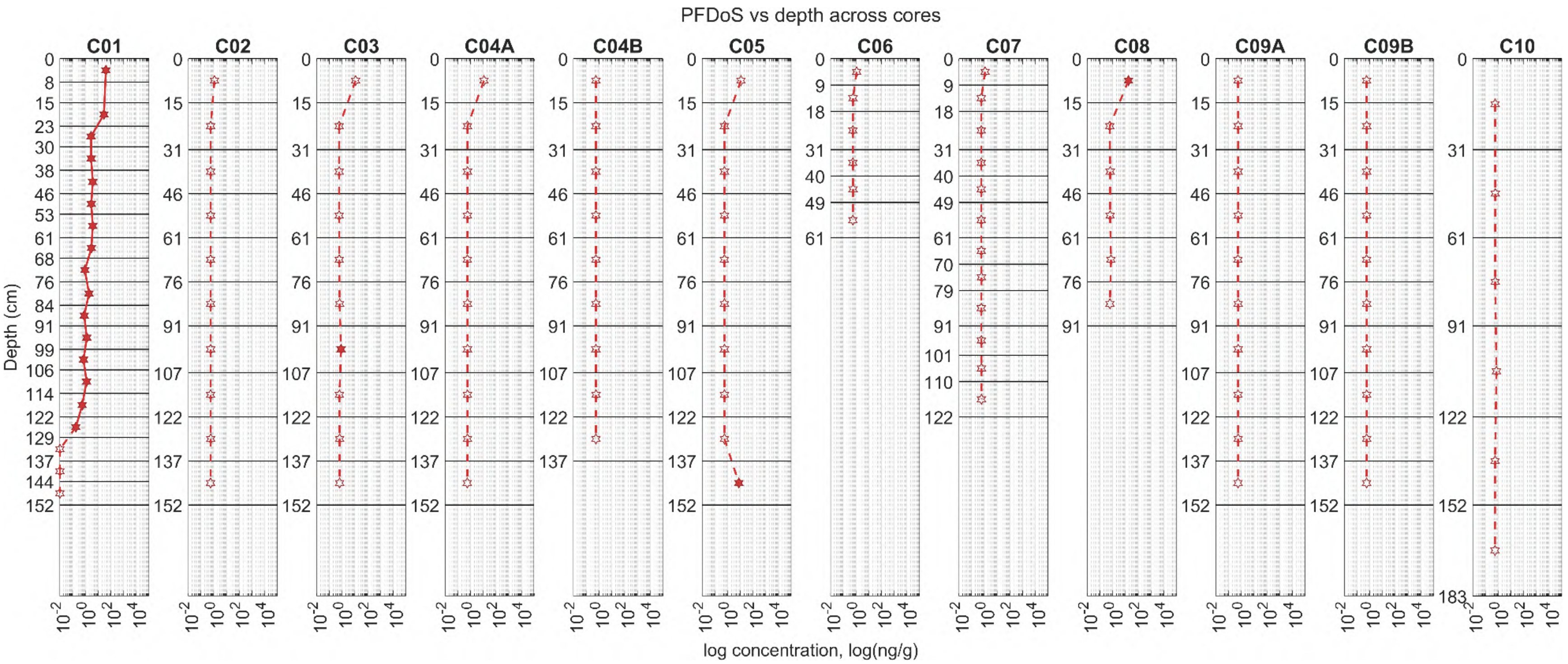

Figure S164. Spearman's rank correlation matrices and corresponding p-values of PFASs within the **SPr-FASA** class

The correlation analysis was conducted using data from all sampled intervals across all cores. Below the diagonal are Spearman's rank correlation coefficients, which range from 1 (red) to -1 (blue). Above the diagonal are corresponding p-values, where p-values above 0.05 are shaded grey and those below 0.05 are shaded orange. Orange cells above the diagonal indicate strong statistical significance.

Compound	SPr-FPrSA	SPr-FBSA	SPr-FPeSA	SPr-FHxSA
SPr-FPrSA		6.83E-12	4.16E-04	3.29E-07
SPr-FBSA	0.58		3.46E-29	3.10E-23
SPr-FPeSA	0.32	0.81		3.35E-24
SPr-FHxSA	0.45	0.76	0.77	

Figure S165. Spearman's rank correlation matrices and corresponding p-values of PFASs within the **SPrAmPr-FASA** class

The correlation analysis was conducted using data from all sampled intervals across all cores. Below the diagonal are Spearman's rank correlation coefficients, which range from 1 (red) to -1 (blue). Above the diagonal are corresponding p-values, where p-values above 0.05 are shaded grey and those below 0.05 are shaded orange. Orange cells above the diagonal indicate strong statistical significance.

Compound	SPrAmPr-FPrSA	SPrAmPr-FBSA	SPrAmPr-FPeSA	SPrAmPr-FHxSA
SPrAmPr-FPrSA		6.99E-30	8.18E-09	1.27E-05
SPrAmPr-FBSA	0.82		5.17E-29	1.82E-08
SPrAmPr-FPeSA	0.50	0.81		6.97E-06
SPrAmPr-FHxSA	0.39	0.49	0.40	

Figure S166. Spearman's rank correlation matrices and corresponding p-values of PFASs within the **H-PFSA** class

The correlation analysis was conducted using data from all sampled intervals across all cores. Below the diagonal are Spearman's rank correlation coefficients, which range from 1 (red) to -1 (blue). Above the diagonal are corresponding p-values, where p-values above 0.05 are shaded grey and those below 0.05 are shaded orange. Orange cells above the diagonal indicate strong statistical significance.

Compound	H-PFPeS	H-PFHxS	H-PFOS
H-PFPeS		6.46E-01	9.41E-04
H-PFHxS	-0.04		5.06E-10
H-PFOS	0.30	0.53	

Figure S167. Spearman's rank correlation matrices and corresponding p-values of PFASs within the **S-OHPrAmPr-FASA** class

The correlation analysis was conducted using data from all sampled intervals across all cores. Below the diagonal are Spearman's rank correlation coefficients, which range from 1 (red) to -1 (blue). Above the diagonal are corresponding p-values, where p-values above 0.05 are shaded grey and those below 0.05 are shaded orange. Orange cells above the diagonal indicate strong statistical significance.

Compound	S-OHPrAmPr-FBSA	S-OHPrAmPr-FPeSA	S-OHPrAmPr-FHxSA
S-OHPrAmPr-FBSA		1.58E-18	7.50E-06
S-OHPrAmPr-FPeSA	0.70		4.63E-12
S-OHPrAmPr-FHxSA	0.40	0.58	

Figure S168. Spearman's rank correlation matrices and corresponding p-values of PFASs within the **diOHPrAm-MeOHPr-FASA** class

The correlation analysis was conducted using data from all sampled intervals across all cores. Below the diagonal are Spearman's rank correlation coefficients, which range from 1 (red) to -1 (blue). Above the diagonal are corresponding p-values, where p-values above 0.05 are shaded grey and those below 0.05 are shaded orange. Orange cells above the diagonal indicate strong statistical significance.

Compound	diOHPrAm-MeOHPr-FBSA	diOHPrAm-MeOHPr-FPeSA	diOHPrAm-MeOHPr-FHxSA
diOHPrAm-MeOHPr-FBSA		6.27E-32	5.42E-10
diOHPrAm-MeOHPr-FPeSA	0.84		7.85E-13
diOHPrAm-MeOHPr-FHxSA	0.53	0.60	

Figure S169. Spearman's rank correlation matrices and corresponding p-values of PFASs within the **diOHPrAm-MeOHPr-FASAPrS** class

The correlation analysis was conducted using data from all sampled intervals across all cores. Below the diagonal are Spearman's rank correlation coefficients, which range from 1 (red) to -1 (blue). Above the diagonal are corresponding p-values, where p-values above 0.05 are shaded grey and those below 0.05 are shaded orange. Orange cells above the diagonal indicate strong statistical significance.

Compound	diOHPrAm-MeOHPr-FBSAPrS	diOHPrAm-MeOHPr-FPeSAPrS	diOHPrAm-MeOHPr-FHxSAPrS
diOHPrAm-MeOHPr-FBSAPrS		9.31E-16	3.88E-06
diOHPrAm-MeOHPr-FPeSAPrS	0.65		2.70E-14
diOHPrAm-MeOHPr-FHxSAPrS	0.41	0.63	

Figure S170. Spearman's rank correlation matrices and corresponding p-values of PFASs within the **Cl-PFSA** class

The correlation analysis was conducted using data from all sampled intervals across all cores. Below the diagonal are Spearman's rank correlation coefficients, which range from 1 (red) to -1 (blue). Above the diagonal are corresponding p-values, where p-values above 0.05 are shaded grey and those below 0.05 are shaded orange. Orange cells above the diagonal indicate strong statistical significance.

Compound	Cl-PFBS	Cl-PFHxS	Cl-PFOS
Cl-PFBS		9.69E-18	3.70E-06
Cl-PFHxS	0.69		3.42E-16
Cl-PFOS	0.41	0.66	

Figure S171. Spearman's rank correlation matrices and corresponding p-values of PFASs within the **PFSAi** class

The correlation analysis was conducted using data from all sampled intervals across all cores. Below the diagonal are Spearman's rank correlation coefficients, which range from 1 (red) to -1 (blue). Above the diagonal are corresponding p-values, where p-values above 0.05 are shaded grey and those below 0.05 are shaded orange. Orange cells above the diagonal indicate strong statistical significance.

Compound	PFBSi	PFPeSi	PFHxSi	PFOSi
PFBSi		2.01E-25	3.84E-08	2.56E-01
PFPeSi	0.78		3.20E-10	2.23E-01
PFHxSi	0.48	0.54		2.24E-03
PFOSi	0.11	0.11	0.28	

Figure S172. Spearman's rank correlation matrices and corresponding p-values of PFASs within the **X:2 FTSO2PrAd-DiMeEtS** class

The correlation analysis was conducted using data from all sampled intervals across all cores. Below the diagonal are Spearman's rank correlation coefficients, which range from 1 (red) to -1 (blue). Above the diagonal are corresponding p-values, where p-values above 0.05 are shaded grey and those below 0.05 are shaded orange. Orange cells above the diagonal indicate strong statistical significance.

Compound	4:2 FTSO2PrAd-DiMeEtS	6:2 FTSO2PrAd-DiMeEtS	8:2 FTSO2PrAd-DiMeEtS
4:2 FTSO2PrAd-DiMeEtS		3.64E-03	2.02E-01
6:2 FTSO2PrAd-DiMeEtS	0.27		6.24E-16
8:2 FTSO2PrAd-DiMeEtS	-0.12	0.66	

Figure S173. Spearman's rank correlation matrices and corresponding p-values of PFASs within the **X:2 FTS** class

The correlation analysis was conducted using data from all sampled intervals across all cores. Below the diagonal are Spearman's rank correlation coefficients, which range from 1 (red) to -1 (blue). Above the diagonal are corresponding p-values, where p-values above 0.05 are shaded grey and those below 0.05 are shaded orange. Orange cells above the diagonal indicate strong statistical significance.

Compound	4:2 FTS	6:2 FTS	8:2 FTS	10:2 FTS
4:2 FTS		4.31E-18	1.25E-10	2.98E-02
6:2 FTS	0.69		1.17E-25	1.53E-03
8:2 FTS	0.55	0.78		2.30E-10
10:2 FTS	0.20	0.29	0.54	

Figure S174. Spearman's rank correlation matrices and corresponding p-values of PFASs within the **K-PFSA** class

The correlation analysis was conducted using data from all sampled intervals across all cores. Below the diagonal are Spearman's rank correlation coefficients, which range from 1 (red) to -1 (blue). Above the diagonal are corresponding p-values, where p-values above 0.05 are shaded grey and those below 0.05 are shaded orange. Orange cells above the diagonal indicate strong statistical significance.

Compound	K-PFPeS	K-PFHxS	K-PFHpS	K-PFOS
K-PFPeS		9.98E-39	5.23E-24	7.90E-22
K-PFHxS	0.88		3.22E-27	1.59E-31
K-PFHpS	0.77	0.80		6.31E-17
K-PFOS	0.74	0.83	0.67	

Figure S175. Spearman's rank correlation matrices and corresponding p-values of PFASs within the **FASA** class

The correlation analysis was conducted using data from all sampled intervals across all cores. Below the diagonal are Spearman's rank correlation coefficients, which range from 1 (red) to -1 (blue). Above the diagonal are corresponding p-values, where p-values above 0.05 are shaded grey and those below 0.05 are shaded orange. Orange cells above the diagonal indicate strong statistical significance.

Compound	FPrSA	FBSA	FPeSA	FHxSA	FHpSA	FOSA
FPrSA		2.16E-19	1.66E-18	5.20E-16	4.14E-15	7.58E-09
FBSA	0.71		2.61E-76	3.22E-41	3.66E-23	2.06E-11
FPeSA	0.70	0.97		3.37E-56	1.21E-31	3.10E-13
FHxSA	0.66	0.89	0.94		3.06E-45	8.60E-21
FHpSA	0.64	0.76	0.83	0.91		2.33E-27
FOSA	0.50	0.57	0.61	0.73	0.80	

Figure S176. Spearman's rank correlation matrices and corresponding p-values of PFASs within the **FASAA** class

The correlation analysis was conducted using data from all sampled intervals across all cores. Below the diagonal are Spearman's rank correlation coefficients, which range from 1 (red) to -1 (blue). Above the diagonal are corresponding p-values, where p-values above 0.05 are shaded grey and those below 0.05 are shaded orange. Orange cells above the diagonal indicate strong statistical significance.

Compound	FBSAA	FPeSAA	FHxSAA	FOSAA
FBSAA		4.80E-02	1.26E-03	6.05E-01
FPeSAA	0.18		5.49E-14	6.92E-06
FHxSAA	0.29	0.62		2.49E-06
FOSAA	-0.05	0.40	0.42	

Figure S177. Spearman's rank correlation matrices and corresponding p-values of PFASs within the **AmPr-FASA-PrA** class

The correlation analysis was conducted using data from all sampled intervals across all cores. Below the diagonal are Spearman's rank correlation coefficients, which range from 1 (red) to -1 (blue). Above the diagonal are corresponding p-values, where p-values above 0.05 are shaded grey and those below 0.05 are shaded orange. Orange cells above the diagonal indicate strong statistical significance.

Compound	AmPr-FEtSA-PrA	AmPr-FPrSA-PrA	AmPr-FBSA-PrA	AmPr-FHxSA-PrA
AmPr-FEtSA-PrA		0.00E+00	2.18E-19	8.34E-01
AmPr-FPrSA-PrA	1.00		2.18E-19	8.34E-01
AmPr-FBSA-PrA	0.71	0.71		1.16E-03
AmPr-FHxSA-PrA	-0.02	-0.02	0.30	

Figure S178. Spearman's rank correlation matrices and corresponding p-values of PFASs within the **AmPr-FASA** class

The correlation analysis was conducted using data from all sampled intervals across all cores. Below the diagonal are Spearman's rank correlation coefficients, which range from 1 (red) to -1 (blue). Above the diagonal are corresponding p-values, where p-values above 0.05 are shaded grey and those below 0.05 are shaded orange. Orange cells above the diagonal indicate strong statistical significance.

Compound	AmPr-FPrSA	AmPr-FBSA	AmPr-FPeSA	AmPr-FHxSA	AmPr-FHpSA	AmPr-FOSA
AmPr-FPrSA		2.76E-12	2.47E-08	1.32E-03	8.18E-09	4.29E-14
AmPr-FBSA	0.59		1.09E-17	2.07E-05	7.82E-12	1.32E-20
AmPr-FPeSA	0.49	0.69		2.87E-12	4.59E-08	5.43E-13
AmPr-FHxSA	0.29	0.38	0.59		1.61E-03	2.10E-07
AmPr-FHpSA	0.50	0.58	0.48	0.29		1.12E-14
AmPr-FOSA	0.62	0.73	0.60	0.46	0.64	

Figure S179. Spearman's rank correlation matrices and corresponding p-values of PFASs within the **UPFSA** class

The correlation analysis was conducted using data from all sampled intervals across all cores. Below the diagonal are Spearman's rank correlation coefficients, which range from 1 (red) to -1 (blue). Above the diagonal are corresponding p-values, where p-values above 0.05 are shaded grey and those below 0.05 are shaded orange. Orange cells above the diagonal indicate strong statistical significance.

Compound	UPFHxS	UPFHpS	UPFOS	UPFNS	UPFDS
UPFHxS		8.97E-13	1.40E-16	1.99E-20	1.70E-22
UPFHpS	0.60		1.16E-11	1.19E-11	4.35E-06
UPFOS	0.67	0.57		1.78E-22	3.71E-13
UPFNS	0.72	0.57	0.75		7.00E-41
UPFDS	0.75	0.41	0.61	0.89	

Figure S180. Spearman's rank correlation matrices and corresponding p-values of PFASs within the **PFA-OS** class

The correlation analysis was conducted using data from all sampled intervals across all cores. Below the diagonal are Spearman's rank correlation coefficients, which range from 1 (red) to -1 (blue). Above the diagonal are corresponding p-values, where p-values above 0.05 are shaded grey and those below 0.05 are shaded orange. Orange cells above the diagonal indicate strong statistical significance.

Compound	PFPe-OS	PFHx-OS	PFHp-OS	PFO-OS	PFN-OS
PFPe-OS		9.76E-30	2.24E-14	1.46E-16	6.34E-12
PFHx-OS	0.82		1.75E-31	1.68E-31	4.03E-25
PFHp-OS	0.63	0.83		1.47E-32	3.44E-27
PFO-OS	0.67	0.83	0.84		4.46E-64
PFN-OS	0.58	0.78	0.80	0.96	

Figure S181. Spearman's rank correlation matrices and corresponding p-values of PFASs within the **EtFASA** class

The correlation analysis was conducted using data from all sampled intervals across all cores. Below the diagonal are Spearman's rank correlation coefficients, which range from 1 (red) to -1 (blue). Above the diagonal are corresponding p-values, where p-values above 0.05 are shaded grey and those below 0.05 are shaded orange. Orange cells above the diagonal indicate strong statistical significance.

Compound	EtFHxSA	EtFDSA	EtFDoSA
EtFHxSA		2.21E-14	2.27E-07
EtFDSA	0.63		2.18E-19
EtFDoSA	0.45	0.71	

Figure S182. Spearman's rank correlation matrices and corresponding p-values of PFASs within the **MeFASA** class

The correlation analysis was conducted using data from all sampled intervals across all cores. Below the diagonal are Spearman's rank correlation coefficients, which range from 1 (red) to -1 (blue). Above the diagonal are corresponding p-values, where p-values above 0.05 are shaded grey and those below 0.05 are shaded orange. Orange cells above the diagonal indicate strong statistical significance.

Compound	MeFBSA	MeFPeSA	MeFHxSA	MeFOSA
MeFBSA		2.35E-30	4.54E-02	5.73E-01
MeFPeSA	0.82		5.86E-01	6.45E-01
MeFHxSA	0.18	0.05		3.25E-01
MeFOSA	-0.05	-0.04	-0.09	

Figure S183. Spearman's rank correlation matrices and corresponding p-values of PFASs within the **CMeAmPr-FASAPrA** class

The correlation analysis was conducted using data from all sampled intervals across all cores. Below the diagonal are Spearman's rank correlation coefficients, which range from 1 (red) to -1 (blue). Above the diagonal are corresponding p-values, where p-values above 0.05 are shaded grey and those below 0.05 are shaded orange. Orange cells above the diagonal indicate strong statistical significance.

Compound	CMeAmPr-FPrSAPrA	CMeAmPr-FBSAPrA	CMeAmPr-FHxSAPrA
CMeAmPr-FPrSAPrA		2.74E-04	1.58E-18
CMeAmPr-FBSAPrA	0.33		1.09E-02
CMeAmPr-FHxSAPrA	0.70	0.23	

Figure S184. Spearman's rank correlation matrices and corresponding p-values of PFASs within the **diOHBAmPr-FASA** class

The correlation analysis was conducted using data from all sampled intervals across all cores. Below the diagonal are Spearman's rank correlation coefficients, which range from 1 (red) to -1 (blue). Above the diagonal are corresponding p-values, where p-values above 0.05 are shaded grey and those below 0.05 are shaded orange. Orange cells above the diagonal indicate strong statistical significance.

Compound	diOHBAmPr-FBSA	diOHBAmPr-FPeSA	diOHBAmPr-FHxSA	diOHBAmPr-FHpSA	diOHBAmPr-FOSA
diOHBAmPr-FBSA		1.15E-22	8.57E-08	5.68E-06	4.83E-04
diOHBAmPr-FPeSA	0.75		7.01E-08	1.87E-05	9.36E-05
diOHBAmPr-FHxSA	0.47	0.47		6.92E-03	1.99E-04
diOHBAmPr-FHpSA	0.40	0.38	0.25		1.47E-02
diOHBAmPr-FOSA	0.32	0.35	0.34	0.22	

Figure S185. Spearman's rank correlation matrices and corresponding p-values of PFASs within the **EtOH-AmPr-FASA** class

The correlation analysis was conducted using data from all sampled intervals across all cores. Below the diagonal are Spearman's rank correlation coefficients, which range from 1 (red) to -1 (blue). Above the diagonal are corresponding p-values, where p-values above 0.05 are shaded grey and those below 0.05 are shaded orange. Orange cells above the diagonal indicate strong statistical significance.

Compound	EtOH-AmPr-FBSA	EtOH-AmPr-FPeSA	EtOH-AmPr-FHxSA	EtOH-AmPr-FHpSA	EtOH-AmPr-FOSA
EtOH-AmPr-FBSA		1.69E-15	8.74E-08	7.75E-06	1.51E-09
EtOH-AmPr-FPeSA	0.65		1.55E-12	1.81E-09	7.00E-13
EtOH-AmPr-FHxSA	0.47	0.59		5.36E-04	8.39E-05
EtOH-AmPr-FHpSA	0.40	0.52	0.31		1.80E-36
EtOH-AmPr-FOSA	0.52	0.60	0.35	0.86	

Figure S186. Spearman's rank correlation matrices and corresponding p-values of PFASs within the **TAmPr-N-MeFASA** class

The correlation analysis was conducted using data from all sampled intervals across all cores. Below the diagonal are Spearman's rank correlation coefficients, which range from 1 (red) to -1 (blue). Above the diagonal are corresponding p-values, where p-values above 0.05 are shaded grey and those below 0.05 are shaded orange. Orange cells above the diagonal indicate strong statistical significance.

Compound	TAmPr-N-MeFBSA	TAmPr-N-MeFPeSA	TAmPr-N-MeFHxSA	TAmPr-N-MeFOSA
TAmPr-N-MeFBSA		2.59E-30	8.85E-05	1.91E-08
TAmPr-N-MeFPeSA	0.82		3.25E-06	4.03E-06
TAmPr-N-MeFHxSA	0.35	0.41		3.79E-02
TAmPr-N-MeFOSA	0.49	0.41	0.19	

Figure S187. Spearman's rank correlation matrices and corresponding p-values of PFASs within the **TAmPr-FASA** class

The correlation analysis was conducted using data from all sampled intervals across all cores. Below the diagonal are Spearman's rank correlation coefficients, which range from 1 (red) to -1 (blue). Above the diagonal are corresponding p-values, where p-values above 0.05 are shaded grey and those below 0.05 are shaded orange. Orange cells above the diagonal indicate strong statistical significance.

Compound	TAmPr-FBSA	TAmPr-FPeSA	TAmPr-FHxSA	TAmPr-FHpSA	TAmPr-FOSA
TAmPr-FBSA		4.94E-06	1.64E-03	8.85E-07	8.24E-03
TAmPr-FPeSA	0.41		2.89E-03	4.25E-03	4.37E-04
TAmPr-FHxSA	0.29	0.27		1.98E-07	1.61E-20
TAmPr-FHpSA	0.43	0.26	0.46		4.63E-12
TAmPr-FOSA	0.24	0.32	0.73	0.58	

Figure S188. Spearman's rank correlation matrices and corresponding p-values of PFASs within the **EtAmPr-FA-N-EtSA** class

The correlation analysis was conducted using data from all sampled intervals across all cores. Below the diagonal are Spearman's rank correlation coefficients, which range from 1 (red) to -1 (blue). Above the diagonal are corresponding p-values, where p-values above 0.05 are shaded grey and those below 0.05 are shaded orange. Orange cells above the diagonal indicate strong statistical significance.

Compound	EtAmPr-FEt-N-EtSA	EtAmPr-FPr-N-EtSA	EtAmPr-FB-N-EtSA	EtAmPr-FPe-N-EtSA	EtAmPr-FHx-N-EtSA	EtAmPr-FHp-N-EtSA	EtAmPr-FO-N-EtSA
EtAmPr-FEt-N-EtSA		4.63E-12	4.89E-09	4.63E-12	1.43E-05	0.00E+00	0.00E+00
EtAmPr-FPr-N-EtSA	0.58		2.24E-37	0.00E+00	5.48E-16	4.63E-12	4.63E-12
EtAmPr-FB-N-EtSA	0.51	0.87		2.24E-37	4.62E-23	4.89E-09	4.89E-09
EtAmPr-FPe-N-EtSA	0.58	1.00	0.87		5.48E-16	4.63E-12	4.63E-12
EtAmPr-FHx-N-EtSA	0.39	0.66	0.76	0.66		1.43E-05	1.43E-05
EtAmPr-FHp-N-EtSA	1.00	0.58	0.51	0.58	0.39		0.00E+00
EtAmPr-FO-N-EtSA	1.00	0.58	0.51	0.58	0.39	1.00	

Figure S189. Spearman's rank correlation matrices and corresponding p-values of PFASs within the **PFCA** class

The correlation analysis was conducted using data from all sampled intervals across all cores. Below the diagonal are Spearman's rank correlation coefficients, which range from 1 (red) to -1 (blue). Above the diagonal are corresponding p-values, where p-values above 0.05 are shaded grey and those below 0.05 are shaded orange. Orange cells above the diagonal indicate strong statistical significance.

Compound	PFBA	PFPeA	PFHxA	PFHpA	PFOA	PFNA	PFDA	PFUdA	PFDoA
PFBA		6.26E-22	5.04E-20	2.37E-16	4.03E-17	2.19E-02	5.57E-01	8.59E-01	6.51E-02
PFPeA	0.74		9.44E-26	1.58E-30	1.40E-23	1.60E-05	3.96E-03	2.09E-02	6.67E-05
PFHxA	0.72	0.78		8.40E-50	1.60E-54	6.06E-07	6.52E-05	3.64E-02	1.56E-03
PFHpA	0.66	0.83	0.92		1.39E-45	3.08E-10	2.13E-05	2.69E-03	1.37E-05
PFOA	0.68	0.76	0.94	0.91		1.59E-08	2.78E-04	5.06E-02	2.60E-04
PFNA	0.21	0.39	0.44	0.54	0.49		1.31E-13	1.42E-09	1.52E-07
PFDA	0.05	0.26	0.36	0.38	0.33	0.61		1.58E-17	6.43E-09
PFUdA	0.02	0.21	0.19	0.27	0.18	0.52	0.68		1.47E-14
PFDoA	0.17	0.36	0.29	0.39	0.33	0.46	0.50	0.63	

Figure S190. Spearman's rank correlation matrices and corresponding p-values of PFASs within the **PFSA** class

The correlation analysis was conducted using data from all sampled intervals across all cores. Below the diagonal are Spearman's rank correlation coefficients, which range from 1 (red) to -1 (blue). Above the diagonal are corresponding p-values, where p-values above 0.05 are shaded grey and those below 0.05 are shaded orange. Orange cells above the diagonal indicate strong statistical significance.

Compound	PFPrS	PFBS	PFPeS	PFHxS	PFHpS	PFOS	PFNS	PFDS	PFDoS
PFPrS		9.53E-32	1.69E-27	2.03E-23	1.02E-22	1.90E-14	1.22E-08	2.68E-04	1.07E-05
PFBS	0.83		1.78E-35	2.97E-31	3.58E-29	2.36E-16	1.30E-06	2.90E-04	9.94E-04
PFPeS	0.80	0.86		4.53E-61	6.92E-45	7.24E-19	4.36E-06	2.29E-02	1.75E-02
PFHxS	0.76	0.83	0.95		4.41E-53	4.99E-25	1.00E-06	5.77E-03	6.00E-03
PFHpS	0.75	0.81	0.91	0.93		5.55E-32	1.26E-09	4.09E-04	5.17E-04
PFOS	0.63	0.66	0.70	0.78	0.84		2.17E-23	1.04E-10	2.77E-08
PFNS	0.49	0.43	0.41	0.43	0.52	0.76		1.68E-31	2.78E-14
PFDS	0.33	0.33	0.21	0.25	0.32	0.55	0.83		2.56E-14
PFDoS	0.39	0.30	0.22	0.25	0.31	0.48	0.63	0.63	

Figure S191. Spearman's rank correlation matrices and corresponding p-values of **PFOA** and its FT precursors

The correlation analysis was conducted using data from all sampled intervals across all cores, not just those featured in Figure 5. Below the diagonal are Spearman's rank correlation coefficients, which range from 1 (red) to -1 (blue). Above the diagonal are corresponding p-values, where p-values above 0.01 are underlined and shaded grey (statistically insignificant) and those below 0.01 are shaded orange (strong statistical significance). Cells of correlation coefficients with insignificant p-values are also shaded grey.

	8:2 FTSO2PrA	8:2 FTSO2-PrAd-DiMeEtS	8:2 FTS	8:2 FTTh-PrAd-DiMeEtS	8:2 FTSi	8:2 FTSO-PrAd-DiMeEtS	8:2 FTSA-PrB	PFOA	8:2 UFTCA
8:2 FTSO2PrA		**4.8E-14**	**1.8E-06**	**7.5E-06**	**1.3E-03**	**1.5E-18**	5.0E-01	**2.5E-06**	**1.5E-07**
8:2 FTSO2-PrAd-DiMeEtS	**0.62**		**5.9E-17**	**2.6E-03**	4.8E-02	**8.5E-13**	3.1E-01	**3.0E-08**	**3.2E-13**
8:2 FTS	**0.42**	**0.67**		1.6E-02	4.4E-02	**9.3E-08**	2.0E-01	**2.3E-14**	**2.1E-08**
8:2 FTTh-PrAd-DiMeEtS	**0.40**	**0.27**	0.22		**9.5E-03**	3.7E-01	**5.4E-05**	7.4E-01	4.2E-01
8:2 FTSi	**0.29**	0.18	0.19	**0.24**		**6.8E-06**	5.4E-01	6.7E-01	2.9E-01
8:2 FTSO-PrAd-DiMeEtS	**0.70**	**0.60**	**0.47**	0.08	**0.40**		1.9E-01	**9.3E-06**	**3.8E-20**
8:2 FTSA-PrB	0.06	0.09	0.12	**0.36**	-0.06	-0.12		2.4E-01	1.8E-01
PFOA	**0.42**	**0.48**	**0.63**	-0.03	0.04	**0.40**	-0.11		**6.2E-07**
8:2 UFTCA	**0.46**	**0.61**	**0.49**	-0.07	**-0.10**	**0.72**	-0.12	**0.44**	

Figure S192. Side-by-side comparison of PFAS contributions per core when using only EPA Method 1633 analyte list vs extended 1633 + NTA analyte list

Contributions of PFAA and PFAA-precursor ("Precursor") PFASs were calculated in two ways: 1) normalizing to the summation of contributions from all detected EPA Method 1633 analytes and 2) normalizing to the summation of contributions from all detected analytes using the combined target (which encompasses the 1633 analyte list) and nontarget analysis approach described in the manuscript. The total PFAS mass per unit core representing these two categories are listed in the 3rd row of the figure header.

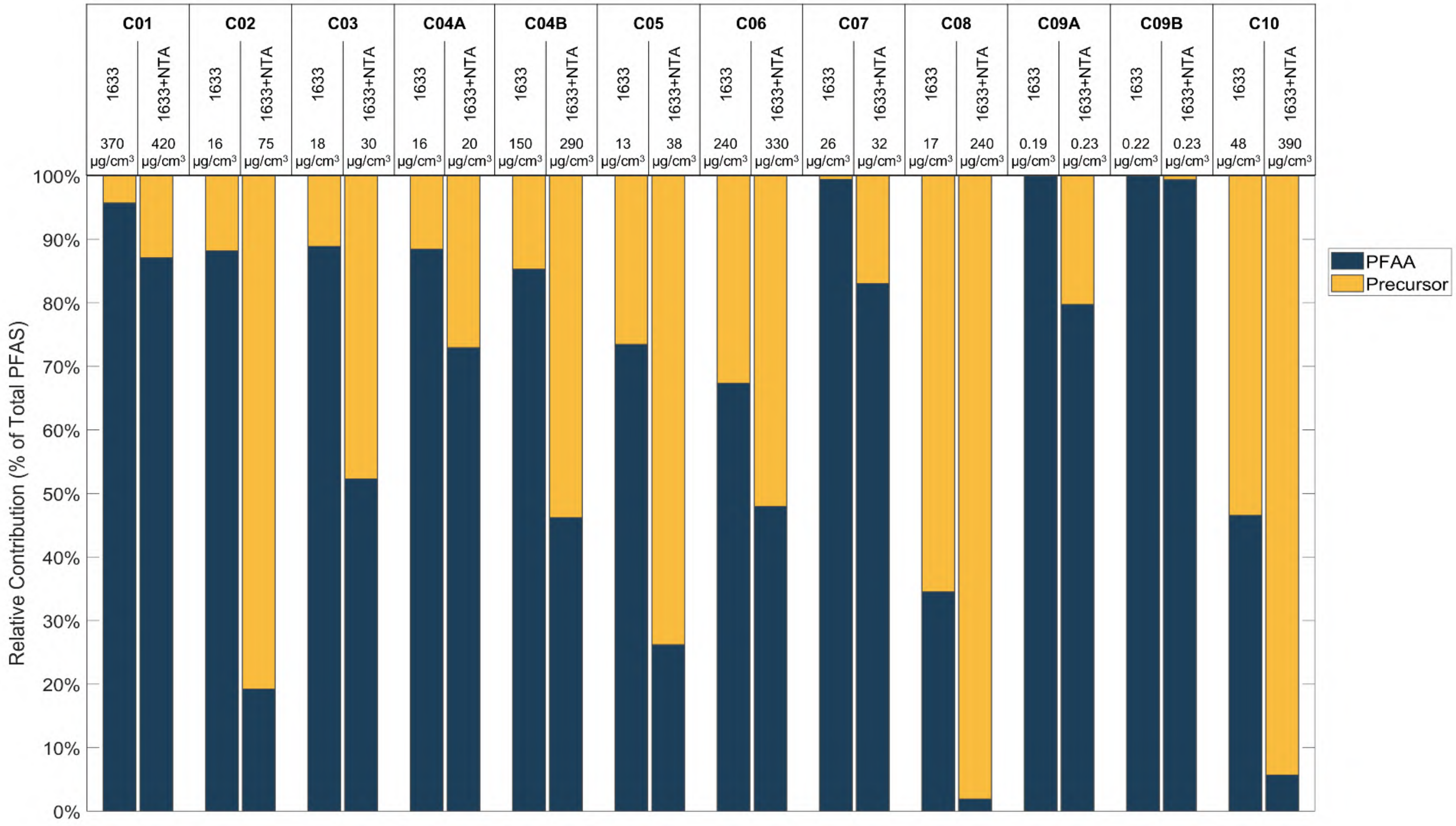

Scheme S1. Transformation scheme of the PFHxA precursors features in Figure 4 based on those proposed in literature

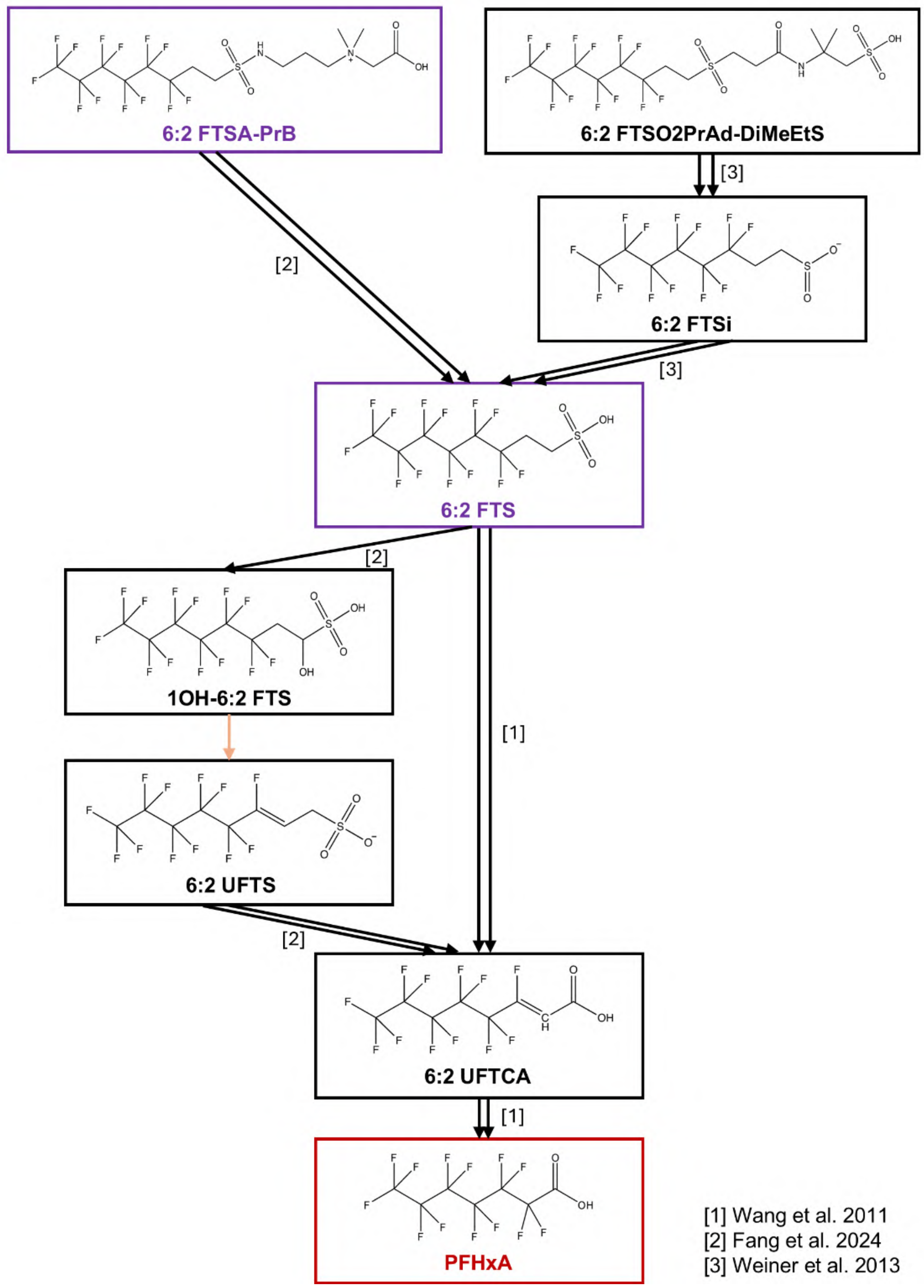

Compounds in purple boxes indicate that they have been identified within AFFF formulations. Compounds in black boxes are proposed intermediate products. Compound in red box is a proposed end product not found within AFFFF formulations. Double arrows indicate potential multi-step reactions that may have other, unidentified intermediates.[8–10] Yellow arrows represent potential reaction pathways that haven't been reported.

Scheme S2. Transformation scheme of the PFHxS precursors featured in Figure 5 based on those proposed in literature

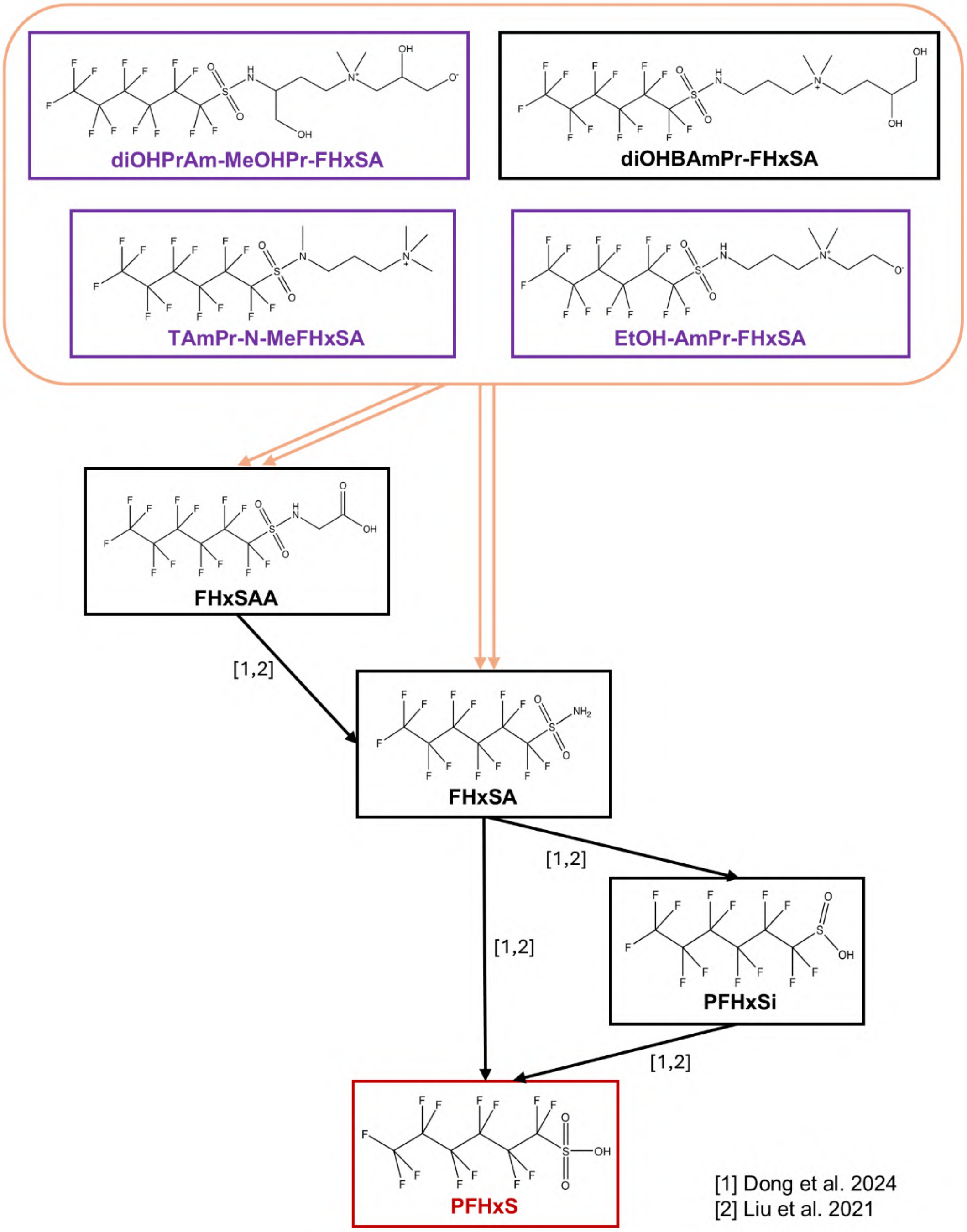

Compounds in purple boxes indicate that they have been identified within AFFF formulations. Compounds in black boxes are proposed intermediate products. Compound in red box is a proposed end product that is also found within AFFFF formulations. Double arrows indicate potential multi-step reactions that may have other, unidentified intermediates.[11,12] Yellow arrows represent potential reaction pathways that haven't been reported.

References

(1) Nickerson, A.; Maizel, A. C.; Kulkarni, P. R.; Adamson, D. T.; Kornuc, J. J.; Higgins, C. P. Enhanced Extraction of AFFF-Associated PFASs from Source Zone Soils. *Environ. Sci. Technol.* **2020**, *54* (8), 4952–4962. https://doi.org/10.1021/acs.est.0c00792.

(2) Gonda, N.; Zhang, C.; Tepedelen, D.; Smith, A.; Schaefer, C.; Higgins, C. P. Quantitative Assessment of Poly- and Perfluoroalkyl Substances (PFASs) in Aqueous Film Forming Foam (AFFF)–Impacted Soils: A Comparison of Analytical Protocols. *Anal. Bioanal. Chem.* **2024**, *416* (29), 6879–6892. https://doi.org/10.1007/s00216-024-05585-2.

(3) Pritchard, J. C.; Hire, M.; McDonough, J.; Higgins, C. P.; Schaefer, C. E. PFAS Conceptual Site Model of an AFFF-Impacted Firefighting Training Area Informed by High Resolution Soil, Porewater, and Groundwater Sampling. *J. Contam. Hydrol.* **2025**, 104728. https://doi.org/10.1016/j.jconhyd.2025.104728.

(4) Benjamin Place. Suspect List of Possible Per- and Polyfluoroalkyl Substances (PFAS), 2021, 3 files, 1.09 MB. https://doi.org/10.18434/MDS2-2387.

(5) Barzen-Hanson, K. A.; Roberts, S. C.; Choyke, S.; Oetjen, K.; McAlees, A.; Riddell, N.; McCrindle, R.; Ferguson, P. L.; Higgins, C. P.; Field, J. A. Discovery of 40 Classes of Per- and Polyfluoroalkyl Substances in Historical Aqueous Film-Forming Foams (AFFFs) and AFFF-Impacted Groundwater. *Environ. Sci. Technol.* **2017**, *51* (4), 2047–2057. https://doi.org/10.1021/acs.est.6b05843.

(6) D'Agostino, L. A.; Mabury, S. A. Identification of Novel Fluorinated Surfactants in Aqueous Film Forming Foams and Commercial Surfactant Concentrates. *Environ. Sci. Technol.* **2014**, *48* (1), 121–129. https://doi.org/10.1021/es403729e.

(7) Hao, S.; Choi, Y. J.; Deeb, R. A.; Strathmann, T. J.; Higgins, C. P. Application of Hydrothermal Alkaline Treatment for Destruction of Per- and Polyfluoroalkyl Substances in Contaminated Groundwater and Soil. *Environ. Sci. Technol.* **2022**, *56* (10), 6647–6657. https://doi.org/10.1021/acs.est.2c00654.

(8) Wang, N.; Liu, J.; Buck, R. C.; Korzeniowski, S. H.; Wolstenholme, B. W.; Folsom, P. W.; Sulecki, L. M. 6:2 Fluorotelomer Sulfonate Aerobic Biotransformation in Activated Sludge of Waste Water Treatment Plants. *Chemosphere* **2011**, *82* (6), 853–858. https://doi.org/10.1016/j.chemosphere.2010.11.003.

(9) Fang, B.; Zhang, Y.; Chen, H.; Qiao, B.; Yu, H.; Zhao, M.; Gao, M.; Li, X.; Yao, Y.; Zhu, L.; Sun, H. Stability and Biotransformation of 6:2 Fluorotelomer Sulfonic Acid, Sulfonamide Amine Oxide, and Sulfonamide Alkylbetaine in Aerobic Sludge. *Environ. Sci. Technol.* **2024**, *58* (5), 2446–2457. https://doi.org/10.1021/acs.est.3c05506.

(10) Weiner, B.; Yeung, L. W. Y.; Marchington, E. B.; D'Agostino, L. A.; Mabury, S. A. Organic Fluorine Content in Aqueous Film Forming Foams (AFFFs) and Biodegradation of the Foam Component 6 : 2 Fluorotelomermercaptoalkylamido Sulfonate (6 : 2 FTSAS). *Environ. Chem.* **2013**, *10* (6), 486–493. https://doi.org/10.1071/EN13128.

(11) Dong, S.; Yan, P.-F.; Manz, K. E.; Abriola, L. M.; Pennell, K. D.; Cápiro, N. L. Fate and Transformation of 15 Classes of Per- and Polyfluoroalkyl Substances in Aqueous Film-Forming Foam (AFFF)-Amended Soil Microcosms. *Environ. Sci. Technol.* **2024**, *58* (51), 22777–22789. https://doi.org/10.1021/acs.est.4c08665.

(12) Liu, M.; Munoz, G.; Vo Duy, S.; Sauvé, S.; Liu, J. Stability of Nitrogen-Containing Polyfluoroalkyl Substances in Aerobic Soils. *Environ. Sci. Technol.* **2021**, *55* (8), 4698–4708. https://doi.org/10.1021/acs.est.0c05811.
